# Integrating single-cell regulatory atlas and multi-omics data for differential treatment response and multimodal predictive modeling in CDK 4/6 inhibitor-treated breast cancer

**DOI:** 10.3389/fonc.2025.1585574

**Published:** 2025-07-17

**Authors:** Li Yan, Sijie Chen, Ran Ran, Shidi Zhao, Jing Huang, Jin Yang

**Affiliations:** ^1^ Department of Medical Oncology, The First Affiliated Hospital of Xi’an Jiaotong University, Xi’an, China; ^2^ Cancer Center, The First Affiliated Hospital of Xi’an Jiaotong University, Xi’an, China; ^3^ Precision Medicine Center, The First Affiliated Hospital of Xi’an Jiaotong University, Xi’an, China; ^4^ Phase I Clinical Trial Ward, The First Affiliated Hospital of Xi’an Jiaotong University, Xi’an, China

**Keywords:** breast cancer, TPRI, CDK 4/6 inhibitors, prognostic model, transcriptional regulation, TCGA, single-cell sequencing

## Abstract

**Introduction:**

CDK4/6 inhibitors are cornerstone therapies for advanced HR+/HER2- breast cancer, yet treatment response heterogeneity remains a major clinical challenge. This study integrates single-cell regulatory landscapes with multi-omics data to decode resistance mechanisms and develop predictive biomarkers for CDK4/6 inhibitor response stratification.

**Methods:**

Single-cell RNA-seq data (GSE158724, n=14 samples) and bulk multi-omics profiles (TCGA-BRCA, n=1,059; GSE186901, n=90) were analyzed. Gene regulatory networks were reconstructed using SCENIC to identify resistance-specific regulons. The Tumor Prognostic Regulon Index (TPRI) was derived from five prognostic transcription factors and validated in independent cohorts. Experimental validation including qPCR of core TFs was performed in patient-derived samples. Multimodal predictive models integrating TPRI, differentially expressed genes, and miRNAs were developed using logistic regression, with performance assessed via ROC/AUC analysis.

**Results:**

We identified 86 resistance-associated regulons and established TPRI based on five prognostic TFs (ATF1, TEAD4, NFIL3, FOXO1, ETV3). TPRI significantly stratified patients into high/low-risk groups with differential overall survival and treatment response (Fisher’s exact test P=0.0237). qPCR confirmed elevated expression of these TFs in resistant tumors (*P*<0.01). High-risk patients exhibited increased stemness indices (mRNAsi, *P*<2.2e-16) and mTOR pathway activation. The multimodal model (TPRI + top 30 DEGs + top 30 miRNAs) achieved superior prognostic accuracy (95%CI:0.6575-0.75).

**Discussion:**

This study establishes TPRI as a novel biomarker for CDK4/6 inhibitor response prediction, validated through multi-omics integration and qPCR confirmation. The model provides actionable risk stratification, where high-risk patients may benefit from combinatorial mTOR-targeted therapies. Limitations include sample size constraints for methylation integration. Future studies should validate these findings in prospective clinical trials.

## 1 Introduction

Tumor immune microenvironment (TME) is a complex ecosystem, immune cells, blood vessels, cytokines, stromal components, and other immune-related cellular and molecular environments within tumor tissues influence tumor growth, metastasis, and response to therapy. The transcriptional regulatory network (GRN) between transcription factors (TFs) and their target genes plays a significant role in determining cellular identity and maintaining cellular status. In breast cancer (BC), dysregulation of the transcriptional program is relevant to the ongoing evolution of cancer cells in TME, which directly affects the patient’s response to therapy.

BC is one of the most prevalent malignant tumors in women. The Lancet estimates that global BC cases are predicted to increase from 2.3 million to more than 3 million (from 2020 to 2040), with an annual mortality rate of 1 million by 2040 (1). Recent years, cyclin-dependent kinase 4 and 6 inhibitors (CDK4/6i), including palbociclib and ribociclib, have been used for the treatment of hormone receptor-positive (HR+), human epidermal growth factor receptor-negative (HER2-) advanced breast cancer (ABC) (2, 3). These drugs selectively target CDK4/6 to prevent Rb phosphorylation in the G1 phase, thus blocking the G1/S transition in the cell cycle (4, 5). Several phase III clinical trials have approved its efficacy, which showed that the combination CDK4/6i and endocrine therapy (ET) greatly enhances progression-free survival (PFS) in contrast to simply ET, and could also postpone the need for following chemotherapy CT (6–12). Moreover, follow-up analyses showed that the combination of CDK4/6i with ET offers an overall survival (OS) benefit, with median OS (mOS) values reported as 53.7 months with ribociclib plus fulvestrant in the MONALEESA-3 trial, and 34.9 months with palbociclib plus fulvestrant in the PALOMA-3 trial (9, 13–15). However, despite the remarkable success, significant differences in response to the efficacy of CDK4/6i among patients. Both of the intrinsic resistance and the acquired resistance affects. Although the factors influencing CDK4/6i resistance are being recognized, treatment has yet to consider the invaluable biological information that provides guidance to clinical treatment, thus combining biomarkers with customized methods to optimize clinical outcomes should be noticed (16).

Omics technologies hold immense promise in cancer research, offering a unique ability to explore cancer biology across various pathological and molecular dimensions (17). For example, single-cell RNA sequencing (scRNA-seq) is a highly effective method for unraveling the complexity of solid tumors, allowing for an in-depth characterization of cellular diversity and the various heterogeneous phenotypic states (18). Regulon is a collection of transcription factors (TFs) and their regulated target genes that is key to regulating multiplication, drug resistance, and stemness characteristics of tumor cells, thereby affecting tumorigenesis, progression, and treatment response. Through the application of these technologies, groundbreaking insights and enhancement of our understanding of biological properties of tumors and mechanisms of drug resistance can be uncovered. However, the characteristics of CDK4/6 inhibitor-treated BC cells are not yet well understood, and current precision therapy for BC faces challenges in achieving personalized treatment and more precise risk stratification. Given that bulk RNA-seq data (e.g., TCGA/GEO cohorts) provide robust statistical power for differential analysis due to their large sample size and comprehensive molecular profiles, while single-cell data are inherently limited by technical noise and small cohort sizes, our study adopts a complementary multi-omics strategy: leveraging bulk data for population-level biomarker screening and survival validation, while utilizing single-cell resolution to dissect regulatory dynamics within resistant cell subpopulations.

In this paper, we studied the prognostic and predictive roles of biomarkers in patients by integrating single-cell transcriptomics data and multi-omics data, and constructed a multilevel predictive model for CDK4/6 inhibitor-resistant HR+/HER2- ABC treatment response through combining the prognostic regulator index (TPRI), differential gene and miRNA, provided a basis for precise treatment.

## 2 Materials

10x single-cell expression profiles from the GSE158724 dataset were coming from the GEO (https://www.ncbi.nlm.nih.gov/geo/) database. This dataset is based on single-cell sequencing from ET and CDK4/6i therapy for BC, and the only cell type it contains are tumor cells. 95 samples were collected, including 14 iCell8 sequencing samples from 10 patients and 81 10x sequencing samples from 36 patients, for a total of 46 patients, numbered P01 through P46. Longitudinal samples were collected from three standardized treatment timepoints: baseline (day 0, S), interim assessment (day 14, M), and treatment completion (day 180, E). Patients were divided into three groups, the first group was ET alone (letrozole plus placebo), the second group was intermittent high-dose combination therapy (letrozole plus a CDK4/6i (600 mg/d, three weeks on and one week off)), and the third group was continuous low-dose combination therapy (letrozole plus CDK4/6 inhibitor (400 mg/d)). Samples from the end-of-treatment phase (E), as well as samples from patients with CDK4/6 inhibitors, were selected for analysis in this program. Patients P11 through P46 were sequenced in 10x single cells, and the sample information is shown below. Patient samples with E samples and treated with Ribociclib were selected for analysis in this project. Thus 14 samples were analyzed, P12_E, P13_E, P21_E, P22_E, P25_E, P27_E, P30_E, P31_E, P33_E, P34_E, P35_E, P37_E, P38_E, P40_E, of which 7 responded to treatment and 7 did not (**Table 1**).

**Table 1:** The sample information of P11 through P46 in 10x single cells: the second column in the table is whether or not there was a response to treatment, and the third column is whether or not a CDK4/6 i was used in the treatment (i.e. Ribociclib).

Patient	Response	Ribociclib given or endocrine alone
P11	No Phase E samples	
P12	Non-responder	1
P13	Non-response	1
P14	Response	0
P15	Non-response	0
P16	Responder	0
P17	No Phase E samples	
P18	No Phase E samples	
P19	No Phase E samples	
P20	Non-response	0
P21	Responder	1
P22	Response	1
P23	No Phase E samples	
P24	No Phase E samples	
P25	Response	1
P26	No Phase E samples	
P27	Response	1

We downloaded the FPKM expression profiles, OS, and clinical information of GDC TCGA-BRCA from UCSCXena (https://xena.ucsc.edu/), and retained 1059 tumor samples with both expression and survival information for the training set of TPRI modeling, miRNA Expression Quantification data for differential miRNA analysis and Illumina Human Methylation 450 data of DNA methylation for differential methylation CpG site analysis. Finally we download Breast Invasive Carcinoma (TCGA, PanCancer Atlas) data from cbioportal, extracting mutation data, CNA data, and clinical data including TMB.

The RNA-seq dataset GSE186901 was downloaded from the GEO database with CDK4/6i (Palbociclib) treatment response information and patient clinical data. There are 90 samples, which are WTS (RNA-Seq) data of 71 patients before and after Palbociclib treatment. Based on the Progressive Disease (PD) event in the clinical data, patients with a PD event of 0 were classified as responding to treatment and patients with a PD event of 1 were classified as not responding to treatment (refractory). The 90 samples included baseline samples from patients with a PD event of 0, and baseline and PD samples from patients with a PD event of 1. Seventeen baseline samples were selected from patients who responded to treatment and 47 baseline samples were selected from patients who did not respond to treatment.

We download the RPKM data for GSE130437 and GSE222367 from the GEO database, which is used for validation of prognostic genes. For GSE130437, there are a total of 12 datasets, divided into two cell lines: the MCF7 cell line and the MDAMB231 cell line. The MCF7 cell line includes 3 parental cell line replicates as controls and 3 palbociclib-resistant replicates; the MDAMB231 cell line includes 3 parental cell line replicates as controls and 3 palbociclib-resistant replicates. As for GSE222367, the MCF7 cell line includes 6 parental cell line replicates as controls, 12 palbociclib-resistant replicates, and 9 Abema-resistant replicates; the T47D cell line includes 3 parental cell line replicates as controls and 12 palbociclib-resistant replicates.

## 3 Methods

### 3.1 Analysis of scRNA-seq data

Fourteen samples from the GSE158724 dataset were processed using the R package *Seurat* (v5.1.0) (19). To ensure data quality, we applied widely adopted quality control thresholds (20): (1) genes were retained only if expressed in at least 3 cells, to eliminate sparsely expressed noise genes; (2) cells with fewer than 200 detected genes were excluded to remove low-complexity or empty droplets; (3) cells with >15% mitochondrial gene content were filtered out to reduce the influence of potential apoptotic cells.

Transcriptomic data processing proceeded through four key stages: (1) Expression normalization: Cellular transcript counts were standardized by log-transformed library size scaling, ensuring comparability across heterogeneous cellular libraries; (2) Feature selection: Highly variable genes (n=2000) were identified through mean-variance relationship modeling, prioritizing transcripts with dispersion exceeding technical noise thresholds; (3) Inter-sample integration: Batch effects were corrected via reciprocal pairwise canonical correlation analysis (CCA) coupled with mutual nearest neighbor anchoring, followed by multi-dimensional scaling-based harmonization; (4) Dimensionality reduction: Principal component analysis (PCA) was performed on the integrated feature space comprising 2,000 highly variable genes (HVGs), a standard parameter optimized to balance biological signal retention and technical noise suppression in single-cell transcriptomic analysis. The top 30 principal components (cumulative variance >80%) were retained for downstream uniform manifold approximation and projection (UMAP) visualization and graph-based clustering (20, 21). All computational workflows were implemented using Seurat (v4.3.0) with default parameters.

### 3.2 Identification and characterization of regulons

Given the unique advantages of single-cell transcriptome data in resolving cellular heterogeneity, dynamic regulatory states and cell subpopulation-specific regulatory features, in this study, we adopted the pySCENIC multimodal analysis framework (22), constructed an initial co-expression network to identify potential transcription factor (TF)-target gene relationships through the GRNBoost2 algorithm, combined with RcisTarget’s cis-regulatory element analysis to screen gene sets with regulatory features (regulon), and AUCell was used to quantify the regulon activity score (RAS) at the single-cell level, and ultimately screen drug-resistant tumor cells (treatment-unresponsive subpopulation) specific regulatory networks by Mann-Whitney U test.

### 3.3 Construction of TPRI

The Tumor Prognostic Regulon Index (TPRI) integrates cross-scale regulatory features derived from single-cell and bulk omics. Specifically, single-cell data capture therapy-responsive transcriptional circuits at cellular resolution, whereas bulk data enable quantification and validation of these regulatory patterns across population cohorts (TCGA, n=1,059). TPRI thus reflects the aggregate activity of resistance-associated regulons at the tissue level, bridging single-cell mechanisms to clinical prognosis.

Based on the TCGA training set, the target gene set of each regulon specific to drug-resistant tumor cells was enriched using the R package GSVA to obtain the TCGA sample enrichment score of each regulon; the GSVA enrichment score of each TF was univariate regression analyzed using the R package coxph for survival, and the five independent prognostic TFs were identified. The regression coefficients of the independent prognostic TFs of each patient were multiplied by the GSVA enrichment scores of the corresponding target genes of the TFs, and then the scores of the five TFs were summed up to obtain the tumor prognostic regulator index (TPRI) of each patient (23). The TPRI score for each sample was calculated as follows:


$$\begin{array}{c} TPRI=\sum_{i=1}^{5}\beta_{i}\cdot S_{i}\; \end{array}$$

where:

*β_i_*: Regression coefficient of the i-th prognostic TF derived from Cox analysis.

*S_i_*: GSVA enrichment score of the target genes regulated by the i-th TF.

### 3.4 Calculation of the stemness index of mRNA expression

The mRNA stemness index (mRNAsi) of tumor malignant cell subpopulations was computed through a one-class logistic regression framework with elastic net regularization (L1/L2 norm penalties), trained on human stem cell transcriptomic reference data from the Progenitor Cell Biology Consortium (PCBC; https://www.synapse.org). This penalized regression model, optimized for sparse feature selection and overfitting prevention, was subsequently applied to quantify stemness characteristics in malignant tumor cells (24).

### 3.5 Analysis of differential gene/methylated CpG site/miRNA

The FPKM expression profiles of TCGA-BRCA were analyzed by R package limma for differential gene expression according to high and low risk groups, and 220 differential genes were screened by adj.pval<0.05, |log2FC|>1, and gene ontology (GO) enrichment analysis was performed by enrichGO of R package clusterProfiler, and 355 enriched pathways were obtained; the Illumina Human Methylation 450 data of TCGA-BRCA were analyzed by limma according to high and low risk groups, and 355 enriched pathways were acquired. We obtained 355 enriched pathways, and used limma to analyze the Illumina Human Methylation 450 data of TCGA-BRCA according to the high and low risk groups, and identified 7243 differentially methylated sites with adj. pval<0.05 and |log2FC|>1. The miRNA expression profiles of TCGA-BRCA were differentially analyzed according to high and low risk groups using limma, and 49 specific miRNAs were identified as highly expressed in the high-risk group and 38 miRNAs were identified as highly expressed in the low-risk group using adj.pval<0.05.

### 3.6 qRT-PCR analysis

This study included CDK4/6 inhibitor-resistant breast cancer cell lines. Total RNA was extracted from these cells using an RNA isolation kit (5201050, Simgen, China). An aliquot of 2 μL of the extracted RNA was used for RNA quantification analysis. Total RNA was then reverse transcribed into cDNA using a reverse transcription kit (RR037A, Takara, Japan) according to the manufacturer’s instructions. Subsequently, real-time quantitative PCR (qRT-PCR) was performed using TB Green Premix Ex Taq II (RR820A, Takara, Japan). Data were normalized to GAPDH as the control. The primer sequences used are as follows: ETV3 (left primer: CCTCCAGGAATGCCATTGGT; right primer: ACATCCCTGGCCTAGCAAAC); FOXO1 (left primer: TGTCAACCTATGGCAGCCAG; right primer: TTGGGTCAGGCGGTTCATAC); NFIL3 (left primer: CATGTCGGAGGAAACGGGAA; right primer: GTCGACGCTTCTCACGAGAT); TEAD4 (left primer: AGGATCTCTTCGAACGGGGA; right primer: ATACTGGCTGGAGACCCCAT);

ATF1 (left primer: TGACACAAGGGCGTCTGTAC; right primer: ATGTGAGCTCCCTGAACTGC). The relative expression of genes was determined using the 2-ΔΔCT method.

Expression levels of ATF1, TEAD4, NFIL3, FOXO1, and ETV3 - were compared between resistant and control replicates across multiple datasets. For the GSE130437 dataset, expression comparison was performed in MCF7 and MDA-MB231 cell lines using 3 resistant replicates versus 3 control replicates. For the GSE222367 dataset, expression comparison was performed in the MCF7 cell line using 12 resistant replicates versus 6 control replicates. For the GSE222367 dataset, expression comparison was performed in the T47D cell line using 12 resistant replicates versus 3 control replicates. RPKM data were processed by applying a log2(RPKM + 1) transformation, and box plots were generated to visualize the results.

Biological informatics analysis and data visualization were performed using R software (version 4.3.3). Statistical analysis was performed using GraphPad Prism (version 9.0). Group comparisons were made using Student’s t-test for two groups, and one-way ANOVA for three or more groups (with Holm-Sidak’s multiple comparison). Statistical significance was set at *P* < 0.05.

## 4 Result

### 4.1 Cell-specific transcriptional GRN identifies differential treatment response in tumor cells

**Figure 1:**
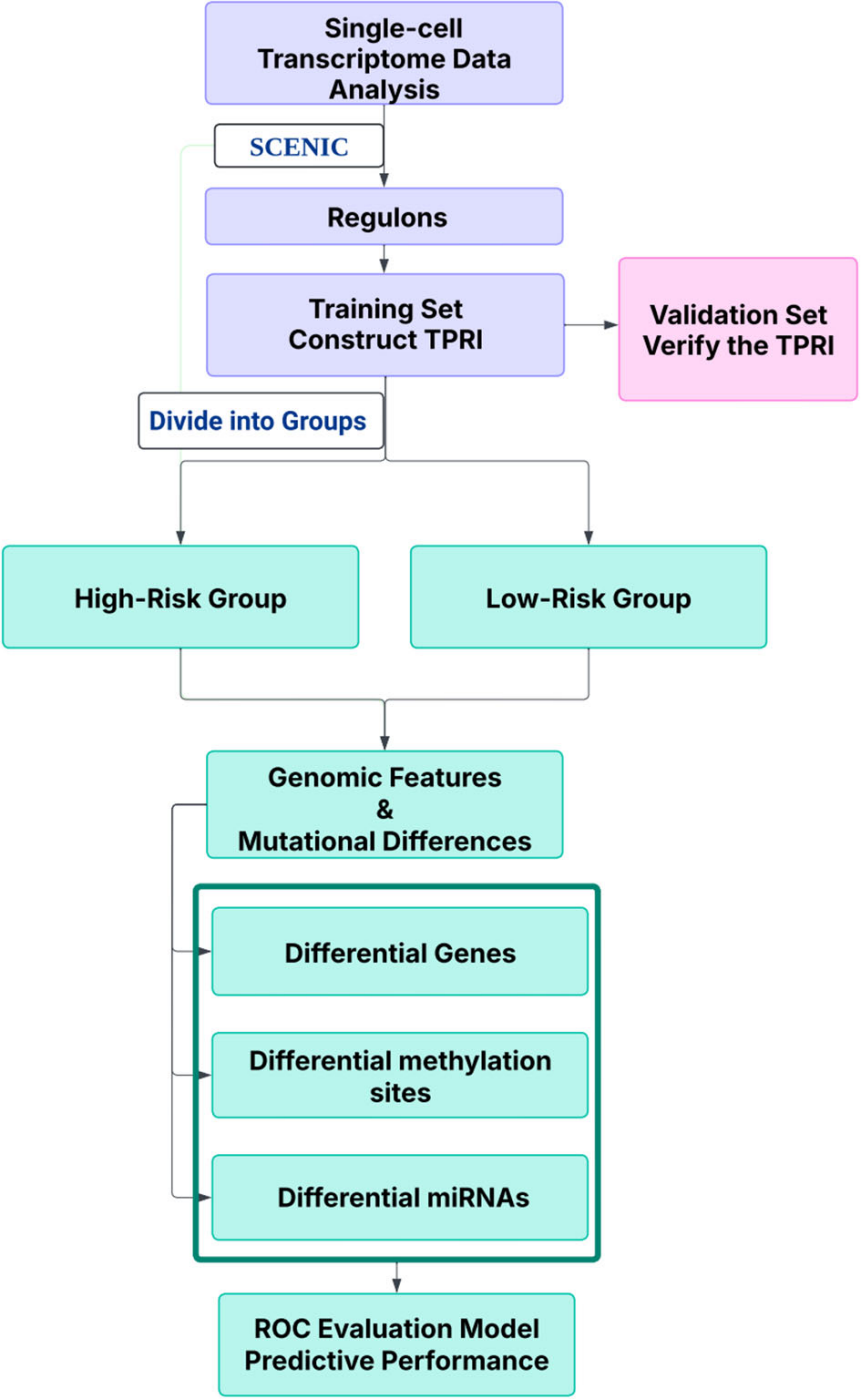
The workflow of this study.

The workflow is outlined in **Figure 1**. Fourteen samples from the GSE158724 dataset were processed using the R package *Seurat* (v5.1.0), and then using pySCENIC, 276 regulons were identified. AUCell regulon activity was differentially tested for each cell according to CDK4/6 inhibitor treatment response/non-response using wilcox.test from the R package, with alternative set to greater to account for highly expressed regulons in the non-response group, 86 regulons were screened out using adj.pval<0.05.

**Figure 2:**
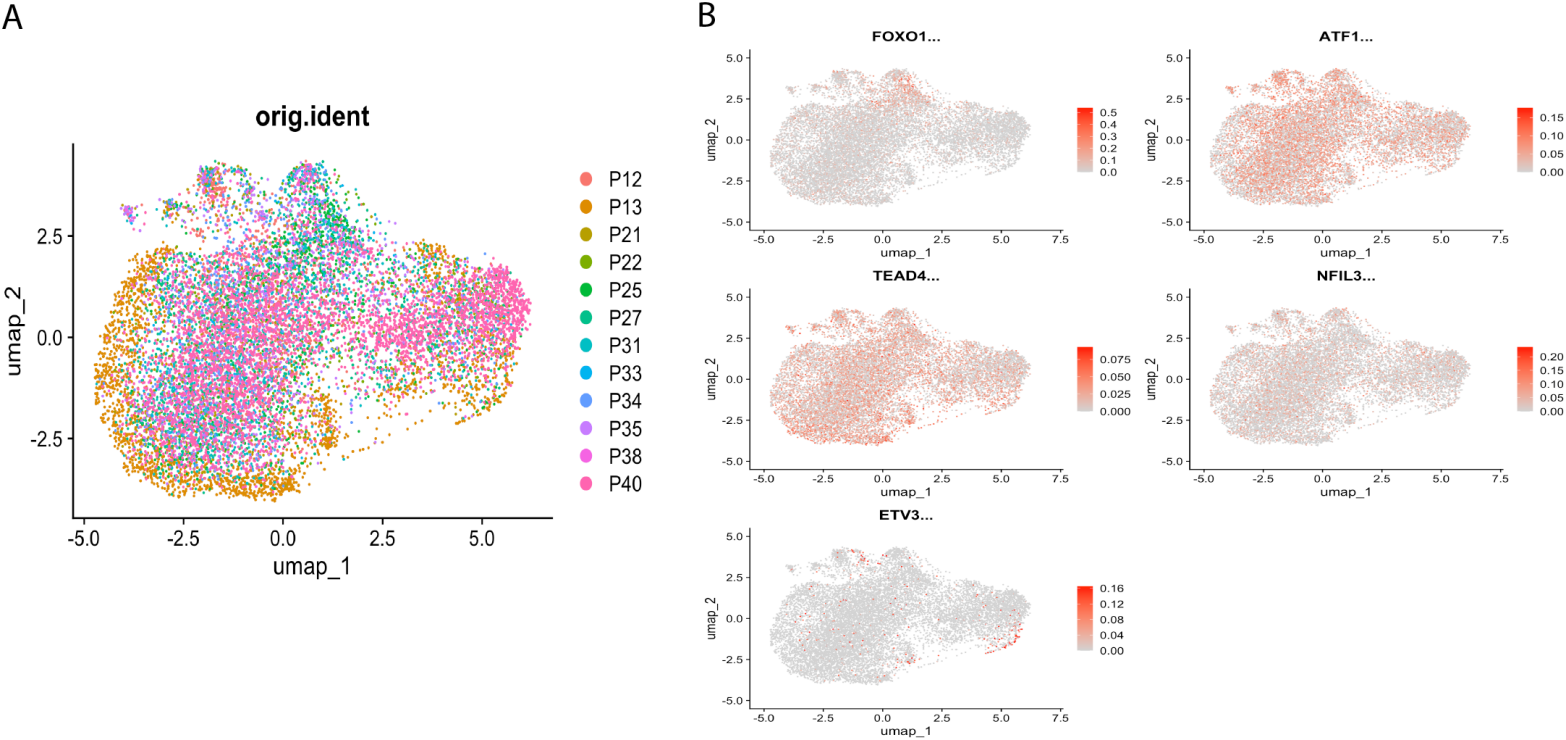
Distribution of regulator expression activity in cells. **(A)** Single-cell UMAP clustering, shown according to the distribution of samples. **(B)** UMAP showing the distribution of regulator expression activity in cells.

Highly variable genes were identified via mean-variance relationship modeling. Inter-sample batch effects were mitigated through canonical correlation analysis (CCA) with mutual nearest neighbor alignment, followed by multi-dimensional scaling integration using *Seurat*. Based on the highest 2000 highly variable genes, principal component analysis was performed, and the top 30 principal components were chosen for UMAP clustering (**Figure 2A**), and UMAP was used to show the distribution of regulon expression activity in the cells (**Figure 2B**).

### 4.2 Prognostic regulon signature identification and TPRI construction

**Figure 3:**
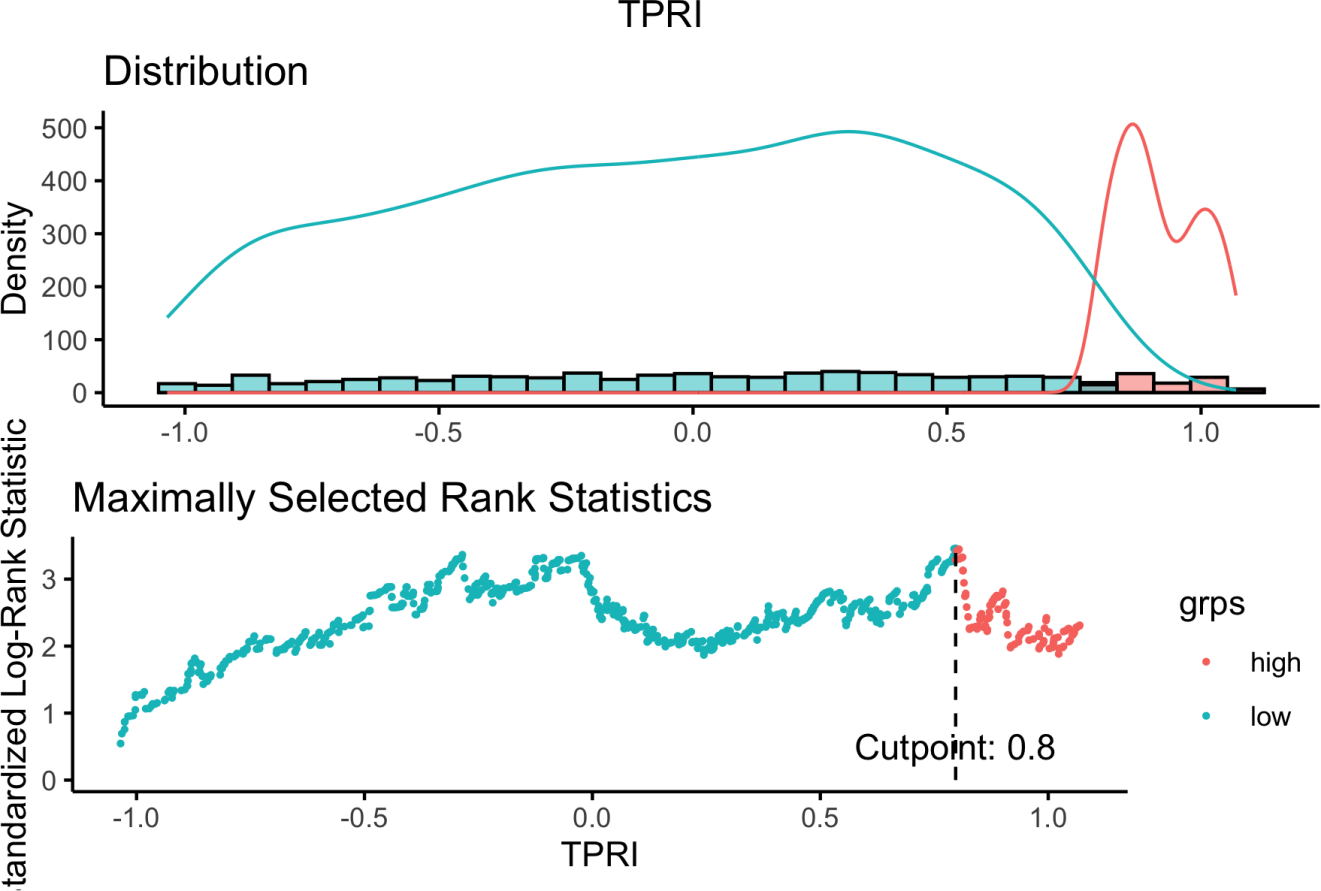
Selection of optimal cutoff value.

Based on the 86 key regulons specific to drug-resistant tumor cells, since each TF has multiple corresponding target genes, the target genes of the same TF were combined to obtain the 86 TFs and their corresponding target gene sets. The GSVA enrichment analysis was performed on the target gene set of each regulon based on the TCGA-BRCA expression profile training set to obtain the enrichment score of each regulon. Based on the enrichment scores of the regulons and the prognostic information of OS and OS. time, five independent prognostic TFs were identified by univariate cox regression analysis with a p-value of <0.05, which were identified as “ATF1”, “TEAD4”, “NFIL3”, “FOXO1” and “ETV3” respectively. The cox proportional risk regression coefficients of the five independent prognostic TFs were multiplied by the GSVA enrichment scores of the target gene sets of the TFs, and then the scores of the five TFs of each TCGA sample were summed to get the TPRI of each sample. Using the surv_cutpoint of the R package survminer, the optimal clinically useful TPRI cutoff value of 0.7968428 (**Figure 3**) was identified using the maximum choice rank statistic, and patients were categorized into high- and low-risk subgroups based on the optimal cutoff value.

### 4.3 TPRI differentiate different therapeutic responses to CDK4/6 i with good prognostic efficacy

Based on the TCGA-BRCA data, analyzed by high/low risk grouping using limma, 1464 differential genes were obtained according to adj. pval<0.05, |log2FC|>0.5, and 2 overlapped with 5 independent prognostic TFs as “FOXO1” and “NFIL3”.

The RNA-seq dataset GSE186901 with CDK4/6 inhibitor treatment response information was selected from public databases as the validation set, and GSVA analysis was performed on the dataset according to the 5 independent prognostic TFs and the GSVA enrichment scores of the 5 TFs were obtained. The cox proportional regression risk coefficients of the five TFs obtained from the TCGA data were multiplied by the GSVA enrichment scores of the TFs obtained from the GSE186901 dataset to calculate the TPRI of each patient, and the optimal TPRI cutoff value of clinically usefulness was identified by the maximum choice rank statistic to be -0.1971562, then the patients in the validation set were categorized into high-risk and low-risk subgroups according to it. Now the patients have two sets of information, one is the PD event of whether they responded to drug treatment or not, and the other is the high/low risk subgroup. To verify whether there is a significant difference in the distribution of patients responding/not responding to the treatment between the high/low risk groups, Fisher’s Exact Test was used, and the p-value was obtained as 0.02368, it can be proved that the grouping of the TPRI model has a significant effect on the prediction of drug resistance.

**Figure 4:**
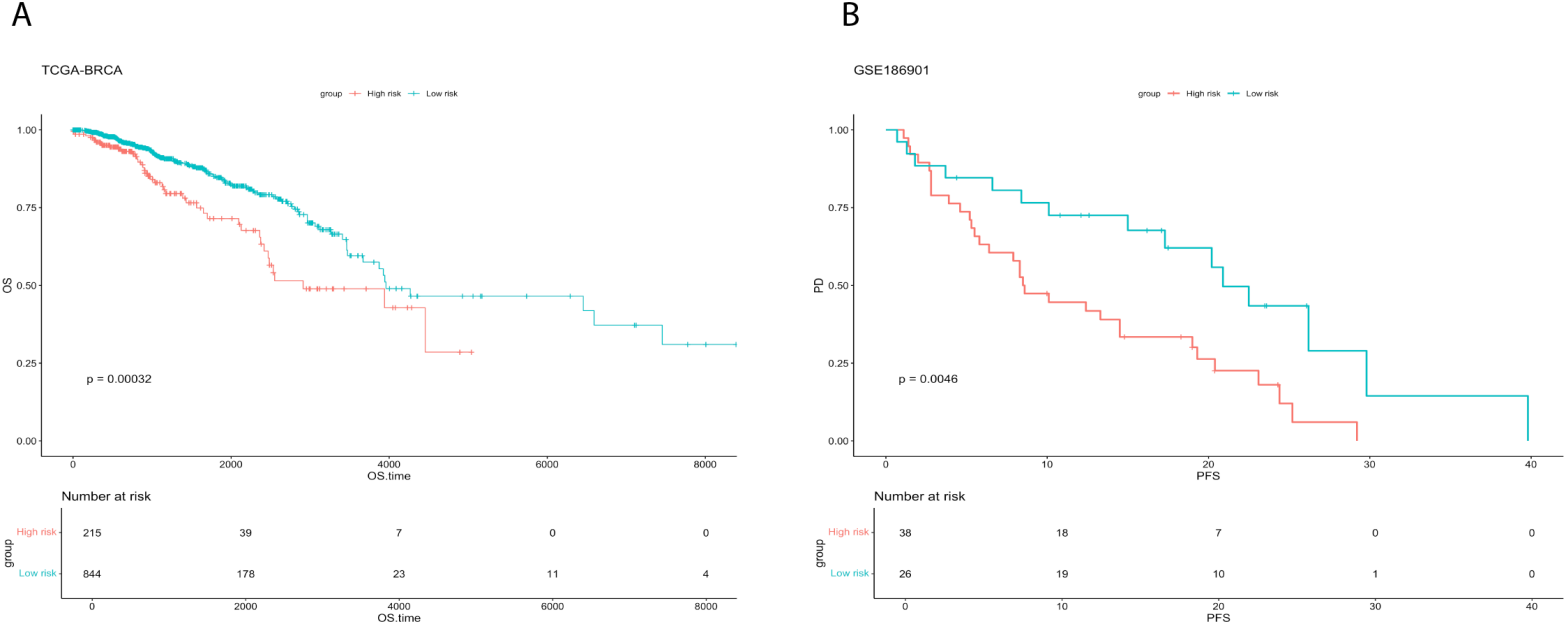
Survival analysis for training and validation sets. **(A)** Comparison of survival curves between high and low risk groups of TCGA-BRCA. **(B)** Comparison of survival curves between high and low risk groups of GSE186901.

Survival analysis demonstrated consistent prognostic stratification by TPRI in both training (TCGA) and independent validation (GSE186901) cohorts (log-rank p<0.05, **Figures 4A, B**). Three key design principles ensure model generalizability: (1) Single-cell data were solely used for regulon discovery, while TPRI training relied entirely on TCGA bulk data (n=1,059), minimizing population bias from limited single-cell cohorts (n=46 patients); (2) TF selection through univariate Cox regression (p<0.05) remained independent of molecular subtypes; (3) Validation in GSE186901 confirmed TPRI’s applicability across diverse genetic backgrounds.

### 4.4 Integrating genomes to explore the genomic features related to CDK4/6 i treatment response in TPRI subgroups

**Figure 5:**
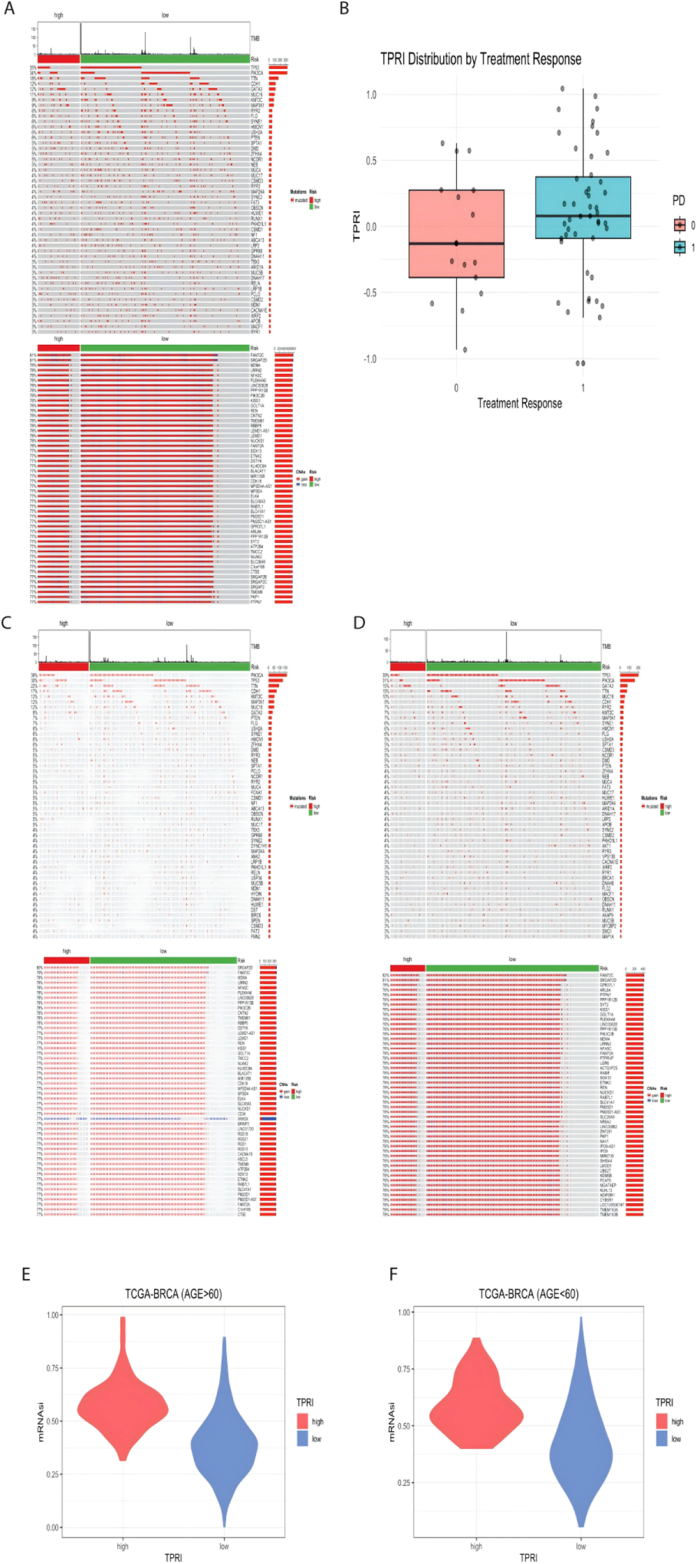
Visualization of genomic features associated with CDK4/6 therapeutic response. **(A)** Genomic mutation map of TCGA-BRCA, from top to bottom, the first part is the distribution of TMB, the second part is the mutation status, and the third part is the distribution of CNA in the genome. **(B)** GSE186901 according to the PD grouping, 0 is the response to the treatment, and 1 is the non-response to the treatment, the distribution of the TPRI value of both groups. **(C)** Genomic mutation map of patients aged 60 years or above in TCGA data. **(D)** Genomic mutation map of patients under 60 years old; E: high and low risk of patients over 60 years old. **(F)** mRNAsi distribution of high- and low-risk groups of patients under 60 years old.

The genomic differences between the high and low risk groups of the TCGA dataset are shown below, mutations, and copy number variations (**Figure 5A**) did not differ significantly between the two groups.

Based on the public dataset GSE186901, using the t-test between patients who responded to treatment and those who did not, although the p value of the difference in TPRIs was 0.1451, which was greater than 0.05, indicating that there was no significant difference in the distribution of TPRIs. However, according to **Figure 5B**, the distribution of TPRI scores of the patients with a PD of 0 (who responded to treatment, i.e., low risk) was higher than the distribution of TPRI scores of the patients with a PD of 1 (who did not respond to treatment, i.e., low risk).

The TCGA data were further subdivided into subgroups based on age, with those older than or equal to 60 years in a group of 438 patients and those younger than 60 years in a group of 524 patients. The genomic analysis of the age-grouped patients was performed according to high and low risk, and it was seen that there was little genomic change between high and low age (**Figures 5C, D**).

The mRNAsi values of each patient were calculated (**Figures 5E, F**), and the wilcox test was performed according to the distribution of the mRNAsi values in the high/low risk groups. The p-value of the difference in mRNAsi values for patients over 60 years old was < 2.2e-16, and the p-value of the difference in mRNAsi values for patients under 60 years old was 7.032e-16, which proved that the mRNAsi values of low-risk patients were significantly lower than those of high-risk patients, suggesting that the stemness of the cells of patients with higher drug resistance is also higher.

### 4.5 Contribution of TPRI in different transcriptomes, epigenomes and miRNAs characterization

**Figure 6:**
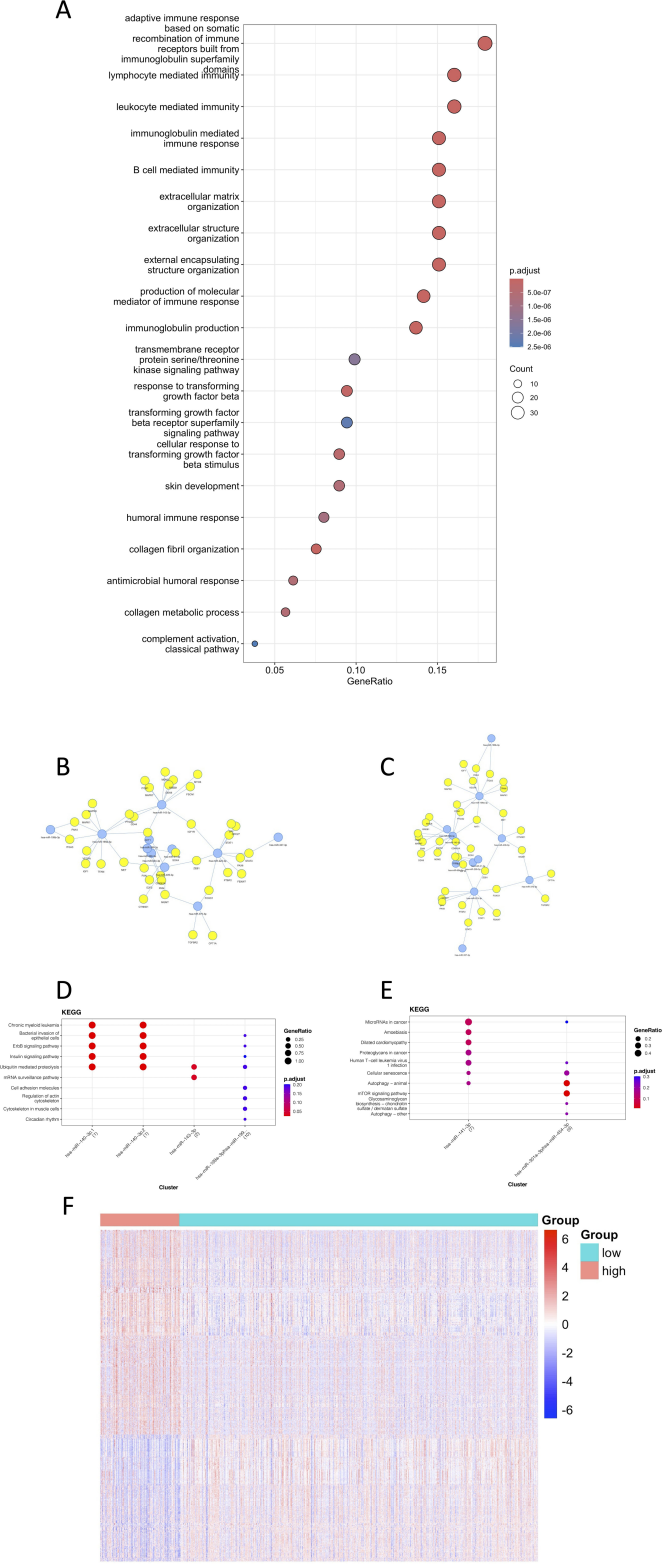
Visualization of the contribution of TPRI to the characterization of different transcriptomes, epigenomes and miRNAs. **(A)** The first 20 GO enrichment results of differential genes. **(B)** Network diagram of miRNAs and their target genes enriched in the high-risk group. **(C)** Network diagram of miRNAs and their target genes enriched in the low-risk group. **(D)** KEGG pathway enrichment of miRNA targets in the low-risk group. **(E)** KEGG pathway enrichment of miRNA targets in the high-risk group. **(F)** Heat map of differentially methylated sites.

The gene variability between high and low risk groups based on the TCGA data was calculated by limma, and 220 genes were screened according to the criteria of adj p value<0.05, |log2FC| > 1. GO enrichment analysis was performed on these 220 genes, and 355 pathways were obtained (**Figure 6A**), which were mainly enriched in pathways of immune and inflammation-related processes (e.g. B/T cell-mediated immunity, immunoglobulin/complement system, and various chemokines/miRNAs), as well as in cell adhesion, cytoskeletal/extracellular matrix remodeling, and signal transduction regulation. Suggesting that different genes in the high and low risk groups are involved in immune defense, which is potentially related to drug resistance (**Supplementary Table S1**).

The miRNAs were grouped according to their risk, and the miRNA differences between the groups were calculated by limma as well. 87 differential miRNAs were screened out according to the criteria of adj p value<0.05, |log2FC|>0.5, 49 miRNAs with higher expression in the high-risk group, and 38 miRNAs with higher expression in the low-risk group. The miRNAs with the highest |logFC| were selected, and the network diagrams of miRNAs and target genes were plotted using mienturnet (25) (**Figures 6B, C**). It can be seen from the enriched pathways in the target genes of miRNAs that the high-risk group had enrichment in the mTOR signaling and the cancer related pathways, which was highly correlated with the occurrence of cancer (**Supplementary Table S2**).

The beta value of the methylation data was converted to M value, and the differentially methylated CpG sites between the high- and low-risk groups were calculated by limma, and 7243 differentially methylated sites were removed according to the criteria of adj p value<0.05, |log2FC| > 1. The heat map of differentially methylated sites (**Figure 6F**) showed that the methylation level was higher in the high-risk group. Studies have showed that methylation alteration is not only associated with tumorigenesis, but also may encourage the evolution of tumor cells and the acquisition of drug resistance, which is consistent with the results that higher drug resistance in the high-risk group. Methylation also tends to lead to transcriptional repression of related genes (including tumor suppressor genes), which may also explain a worse prognosis (**Supplementary Tables S3-4**).

### 4.6 Multimodal data to identify the prognostic risk stratification of patients in different treatment response groups

A multivariable logistic regression framework was implemented to predict overall survival (OS) outcomes, integrating the Tumor Prognostic Regulon Index (TPRI) with differentially expressed genes and miRNAs. A total of 3 models were constructed: (1) Baseline model: TPRI as the sole predictor; (2) Transcriptomic expansion model: TPRI combined with the 30 most significant differentially expressed genes (adjusted p-value <0.05, |log2FC|>1); and (3) Multi-omics integration model: TPRI augmented with both the top 30 miRNAs exhibiting strongest differential expression signals (FDR<0.05). Methylation data were excluded from model construction due to substantial sample size discrepancy (n=646 methylation vs. n=1,059 transcriptomic profiles), which would introduce cohort-specific bias during multimodal integration.

**Figure 7:**
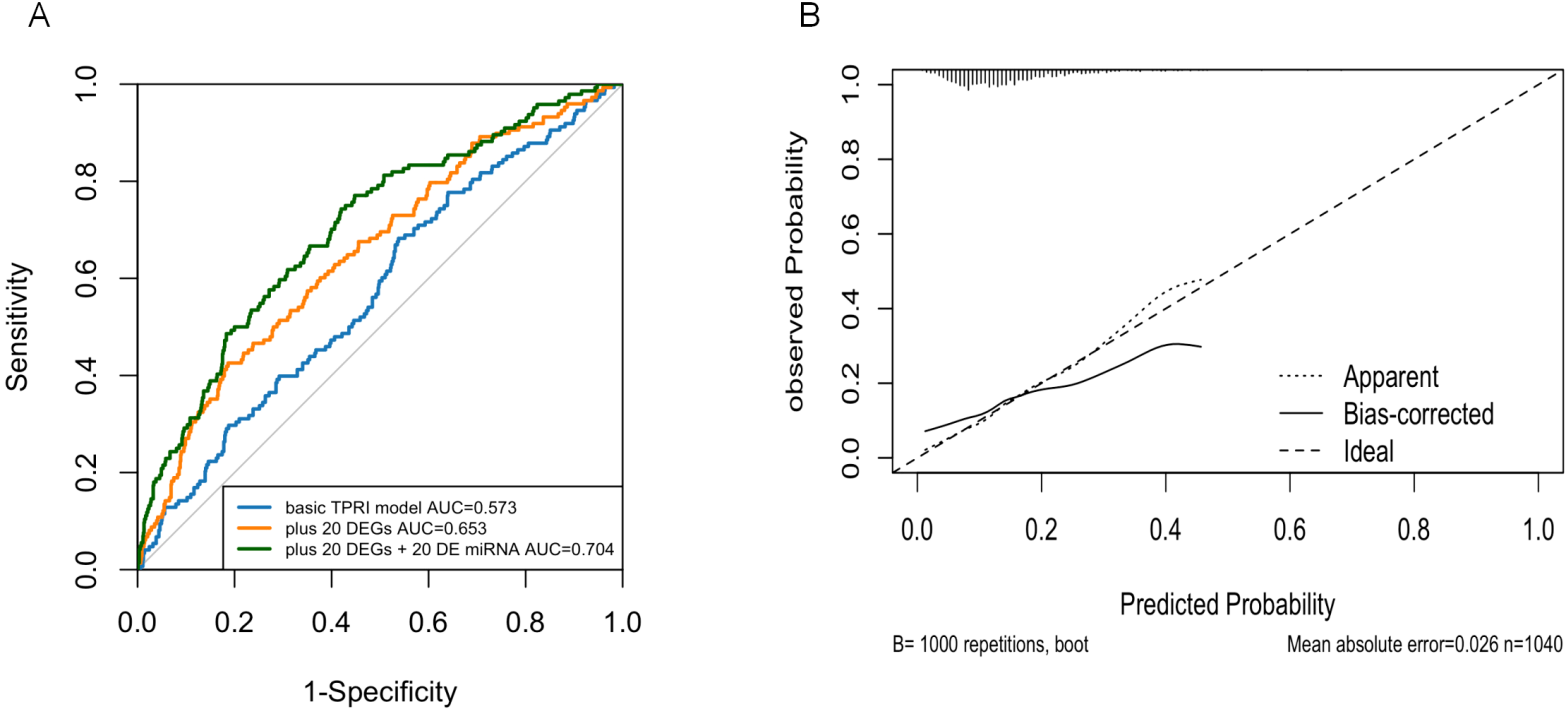
Logistic regression model. **(A)** ROC curves showing the predictive power of the three models. **(B)** Calibration plots showing the consistency of the predicted probabilities of the evaluation models with the actual frequency of observations.

The performance of the prediction model was evaluated using ROC curves and area under the curve (AUC) to assess the ability of the model to distinguish prognostic risks. The results were shown in **Figure 7A**, the third model with TPRI+30 differential genes+30 miRNAs as variables had the largest AUC and the best prediction effect, and the 95% confidence interval (CI) of the third model was 0.6575-0.75, which indicated that the model had a good discriminatory ability.

Nomogram calibration plots using R-package rms showed that the probabilities predicted by the models were generally good (**Figure 7B**).

### 4.7 Validation of the expression of prognostic genes

**Figure 8:**
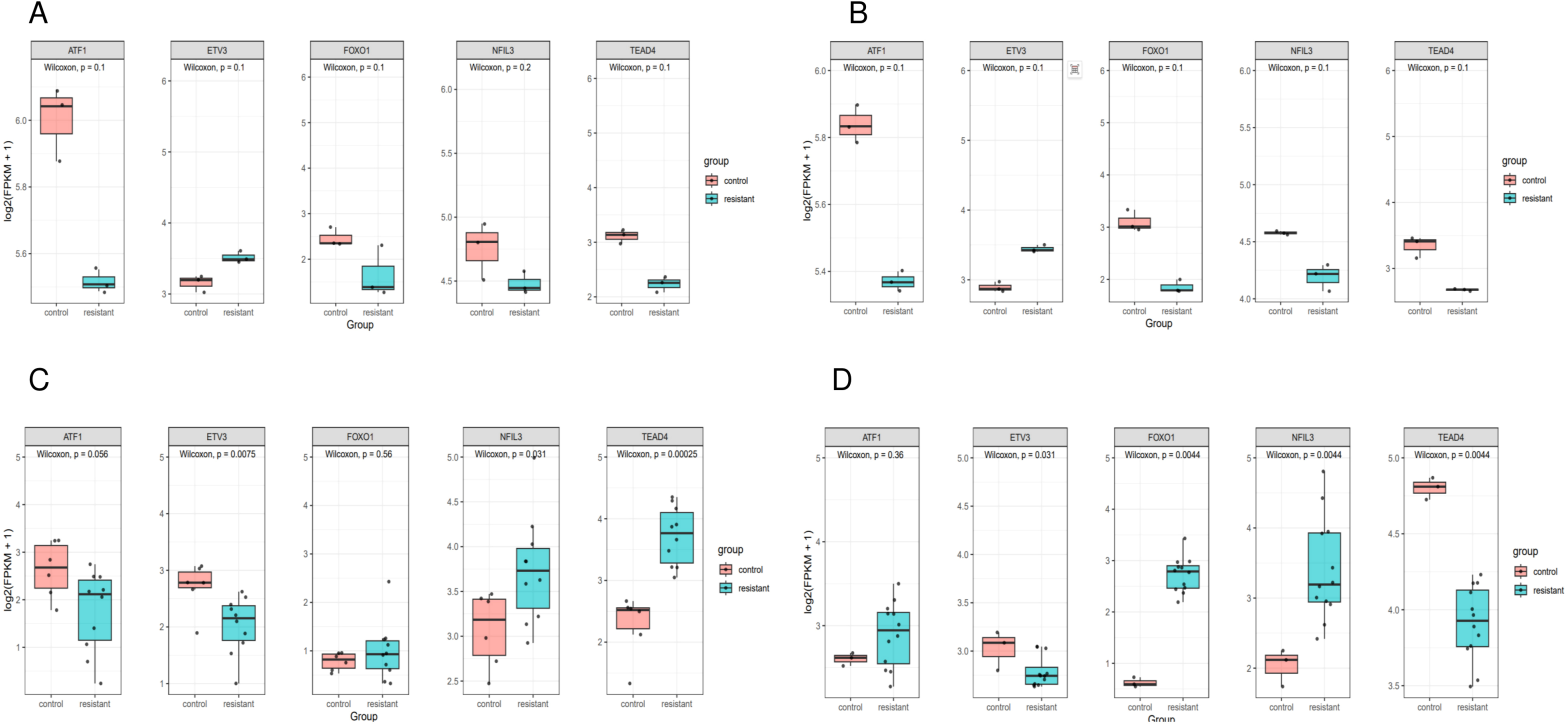
Expression of genes in single cells. Box plot of prognostic gene expression in control and resistant. **(A)** Comparison of the expression in resistant and control MCF7 cell lines from GSE130437 dataset. **(B)** Comparison of the expression in resistant and control MDAMB231 cell lines from GSE130437 dataset. **(C)** Comparison of the expression in resistant and control MCF7 cell lines from GSE222367 dataset. **(D)** Comparison of the expression in resistant and control T47D cell lines from GSE222367 dataset.

**Figure 9:**
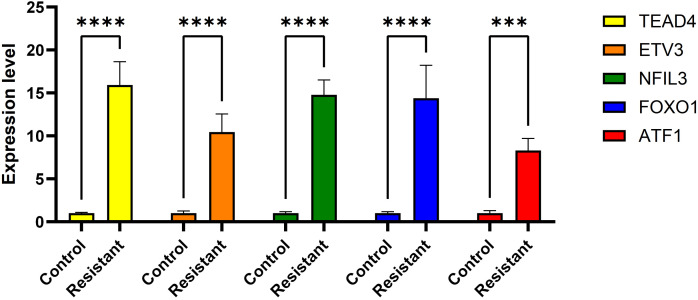
Expression of genes was verified using qRT-PCR. ***P < 0.001, ****P <0.0001.

ATF1 showed no significant changes across all four comparative analyses. ETV3 was significantly downregulated in the resistant group in two datasets (**Figures 8C, D**). FOXO1 was significantly upregulated in the resistant group in one dataset (**Figure 8D**). NFIL3 was significantly upregulated in the resistant group in two datasets (**Figures 8C, D**). TEAD4 was significantly upregulated in the resistant group in one dataset (**Figure 8C**) and significantly downregulated in another dataset (**Figure 8D**). However, qRT-PCR results demonstrated significantly increased expression of ‘ATF1’, ‘TEAD4’, ‘NFIL3’, ‘FOXO1’, and ‘ETV3’ in the resistant group (**Figure 9**).

## 5 Discussion

Dysregulation of regulatory programs is an important factor in tumor development. Single-cell histology studies have increasingly shown that intra-tumor heterogeneity is a significant marker of it. We developed TPRI based on BC single-cell sequencing data and multi-omics data by constructing GRNs with the SCENIC algorithm for accurate prediction of differential response to CDK4/6 inhibitor therapy in BC patients. Patients were divided into high- and low-risk groups according to TPRI, and there was a significant difference in the OS rate between them, suggesting that TPRI has a good differentiation of tumor prognosis. Moreover, it was found that patients in the high-risk group had a higher cell stemness index, while TPRI was used as a model for predicting differential response (resistance/non-resistance) to CDK4/6 inhibitor therapy, the high-risk group corresponded to a higher likelihood of resistance, and the high cell stemness index of the group was in line with its tendency to have a stronger self-renewal as well as drug-resistance potential. The differential genes between the groups were mainly enriched in the immune pathway, suggesting that the TPRI high-risk group may have specific alterations in the immune microenvironment or immune escape, which also provides a reference for the subsequent immunotherapy strategy. The predictive effect of the TPRI index model was verified by the validation set, and there were significant differences in treatment response and survival rates between the high- and low-risk groups of patients in the validation set, which proved that the model had a good differentiation and predictive effect on prognosis and treatment response. Combining the TPRI with differential genes and miRNA for logistic regression modeling, the AUC of the model was 0.694, indicating that the model has a good differentiation of prognosis.

In this study, we present five independent prognostically relevant regulons. These gene signature have been reported to be significant in BC tumorigenesis. Take ATF1 for example, in the process of neural signals in TME steering cancer stemness’ establishing the hierarchical structures of malignant cells, ATF1 enhances cancer stemness by simultaneously activating both nuclear pluripotency factors MYC/NANOG and mitochondrial biogenesis regulators NRF1/TFAM, thus driving nuclear reconfiguration and mitochondrial renewal across various cancer types including BC (26). This is consistent with the result that the TPRI high-risk group had higher stemness. Moreover, ATF1 also promotes CXCR4 expression in BC cells by binding to the CRE/AP-1 element on the CXCR4 promoter, which enhances migration, invasion and metastasis of BC cells (27).Studies also showed that it promote ICD development through involving in angiogenesis (28). ATF1 also promotes FRA-1 expression by binding to the ATF site on the FRA-1 promoter, thereby enhancing the response of cancer cells to mitogens (29). ATF1 is involved in the regulation of PKC/MAPK and PKC/Src pathways through ATP stimulation of P2Y(2) and P2Y(4) receptors, which promotes the phosphorylation of ATF1 in breast cancer cells and drives BC progression (30). TEAD4 (TEA Domain Transcription Factor 4) is widely known as the DNA-binding protein in the YAP transcription complex, which is regulated by the Hippo pathway. It acts primarily as a nuclear protein but is also localized to mitochondria, and enhances metastasis, cancer stem cells and drug resistance through cytoplasmic translocation (31). Through the regulation of YAP1, TEAD4 binds to the TIAM1 enhancer region, thereby activates the expression of TIAM1 and subsequently increases the activity of RAC1 and induces the formation of invadopodia formation and promotes tumor metastasis (32). The novel transcriptional target of TEAD4, RBM8A, also interacts with EIF4A3 to increase the expression of IGF1R and IRS-2 and activate the PI3K/AKT signaling pathway, which further promotes the malignant phenotype of BC cells (33). In a major study it was found that, the upregulation of the TEAD coactivator VGLL1 mediates transcriptional reprogramming and indirectly grants resistance to estrogen receptor (ER) degraders in BC (34). The bZIP transcriptional blocker NFIL3 (nuclear factor interleukin 3-regulated) is overexpressed in different cancers and reduces histone acetylation by combining with neighboring DNA and recruiting histone deacetylase-2 (HDAC2), which prevents the entry of FOXO (Forkhead O) (FOXO1, FOXO3, and FOXO4) transcription factors into TRAIL promoters and into chromatin at the tumor promoter. chromatin at the TRAIL promoter to support tumor cell survival (35). SOX2 is a transcriptional factor for cancer stemness, whose transcriptional expression is promoted by the accumulated FOXO1, which in turn, stimulates FOXO1 transcription and shapes a positive regulatory loop (36). Overexpression of the ETS transcription factors ETV3 and ELF3 is relevant to the most common genomic copy number increase in BC (1q21 and 1q32) at these loci, and the expression of the oncogene MYC correlates with the expression of ETV3 and ELK4 (37).

We categorized all samples into low-risk and high-risk groups based on TPRI. And we observed that the differential genes were mainly enriched in pathways of immune and inflammation-related processes (e.g., B/T cell-mediated immunity, immunoglobulin/complement system, and various types of chemokine/cytokine signaling), and were also significantly enriched in cell adhesion, cytoskeleton/extracellular matrix remodeling, and signal transduction regulation. This suggests that the differential genes are involved in immune defense, which is potentially relevant to drug resistance. In addition, according to the pathway of miRNA target gene enrichment, the high-risk group was enriched in the mTOR signaling pathway. mTOR is an atypical serine-threonine kinase, present between mTORC1 and raptor and PRAS40, and between mTORC2 and rictor, mSIN1 and protor-1/2 (38), is one of the most frequently activated pathways in BC (39). Activation of the PI3K/AKT/mTOR signaling pathway is a contributing factor to disease progression in HR+/HER2- ABC patients with CDK4/6i, and blockade of this signaling pathway is an important area to explore for post-progression therapy. Our study also confirms this. A real-world study in the United States (40) retrospectively analyzed the efficacy of 41 HR+/HER2- BC patients at the University of Pittsburgh Medical Centers in Roswell and Pittsburgh, USA, who were treated with everolimus follow-up after progression on perphenazine therapy, and the results showed that the median PFS was 4.2 months, and the median OS reached approximately 18 months. After progression of first-line CDK4/6 inhibitors, endocrine combination with other targeted therapies, such as CDK4/6 inhibitor reuse, PI3K inhibitors, mTOR inhibitors, and AKT inhibitors, may be hopefully a new option for patients. Although relevant researches are still being tested, our result supports it. Although age is often recognized as a risk factor for cancers, in this study, age differences did not cause significant gene expression changes or genomic variation at the genomic level. This may imply that BC development and progression are mainly driven by other factors (e.g., tumor molecular characteristics) rather than by age alone. And it indirectly suggests that TPRI may be a key factor contributing to the prognostic differences between different age groups.

Finally, TPRI, 30 differential genes combined with differential miRNAs (the top 30 miRNAs with the smallest adj.pvalue) were used as variables to establish a prediction model, which predicts the patients’ sensitivity to drugs and quantifies the prognostic risk, and provides a basis for personalized treatment based on the multi-level information of patients’ gene expression, miRNA expression and stemness characteristics. For example, for high-risk patients, a more aggressive treatment regimen may be required, while low-risk patients can be treated relatively conservatively. We also found that some core TFs play a regulatory role for tumor resistance, and the development of antitumor drugs specifically targeting these TFs will be necessary in the future. Overall, the model provides a more detailed prognostic risk stratification of BC patients, which helps clinical judgment of patients’ treatment options, possible therapeutic efficacy, and provides direction for future research.

We admit the limitations of this paper. The construction of this model relies on samples from the TCGA dataset, which, despite the large sample size, is still limited for different clinical subtypes, genomic backgrounds, and treatment responses. In this study, the model did not include methylation data due to the sample size of it was inconsistent with other data, which may result in the model failing to take full advantage of the influence of epigenetics, and the lack of this information may affect the completeness of the model and the prediction accuracy. Moreover, in real clinical applications, the treatment response of different patients is affected by a variety of factors (e.g., immune system, tumor microenvironment, etc.), and the predictive effect of the model may differ from the real clinical situation. A large number of clinical studies are still needed to validate it. Overall, although this study presents an effective prognostic model for breast cancer, there are limitations in terms of limited sample size, single data source, complexity of the model and lack of clinical validation. Future studies can further improve the model by increasing the sample size, including methylation data and more clinical treatment information, and conducting multicenter and large-scale clinical validation.

## 6 Conclusion

To summarize, this study integrates scRNA-seq and multi-omics data with machine learning to develop a multimodal predictive model for CDK4/6i-resistant BC patients. By combining the TPRI, differentially expressed genes, and miRNAs, we enhanced risk stratification and predictive accuracy for patient outcomes. And the integrated multi-omic analysis revealed that the two subgroups based on TPRI had different survival outcomes, transcriptome, epigenome, and miRNAs signatures. They provide an in-depth comprehension of the heterogeneity of TPRI and the potential for treatment methods to be more personalized. Further studies should highlight on the potential of multi-omics data and machine learning in advancing precision medicine, and the exploration of additional markers and validation with larger datasets.

## Data Availability

The datasets presented in this study can be found in online repositories. The names of the repository/repositories and accession number(s) can be found in the article/**Supplementary Material**.
