# Supplementary material for: Integrating single-cell regulatory atlas and multi-omics data for differential treatment response and multimodal predictive modeling in CDK 4/6 inhibitor-treated breast cancer
Source: Front Oncol. 2025 Jul 17;15:1585574. doi: 10.3389/fonc.2025.1585574 (PMC12310732; doi:10.3389/fonc.2025.1585574)
Supplement: Supplementary file 1 [file Table1.docx]

Supplementary Material

# Supplementary Data

| **Table S1. GO enrichment of differential gene** | | | | | | | | | | | | | | | | | | | | | | | | | | | | | | | | |
| --- | --- | --- | --- | --- | --- | --- | --- | --- | --- | --- | --- | --- | --- | --- | --- | --- | --- | --- | --- | --- | --- | --- | --- | --- | --- | --- | --- | --- | --- | --- | --- | --- |
| **ONTOLOGY** | **ID** | | | **Description** | | | | **GeneRatio** | | **BgRatio** | | | **RichFactor** | | | **FoldEnrichment** | | | | **zScore** | | **pvalue** | | | **p.adjust** | | | | **qvalue** | | **geneID** | **Count** |
| BP | GO:0016064 | | | immunoglobulin mediated immune response | | | | 32/212 | | 208/18888 | | | 0.153846153846154 | | | 13.7068214804064 | | | | 19.6330443684202 | | 3.14605817491602e-27 | | | 7.84168577502925e-24 | | | | 6.209312196182e-24 | | SERPING1/C1R/C1S/C3/CD74/IGHA2/IGHA1/IGHV3-7/IGHV3-74/IGHV3-72/IGHV3-15/IGHM/IGLC2/IGHV5-51/IGHV3-49/IGKC/IGHV3-23/IGHV2-5/IGHV4-59/IGHV3-30/IGHV6-1/IGHV4-39/IGHV3-11/IGHV4-34/IGHV3-48/IGLC3/IGHV1-24/IGHV4-4/IGHV3-21/IGHV1-18/IGHV1-3/IGHG1 | 32 |
| BP | GO:0019724 | | | B cell mediated immunity | | | | 32/212 | | 212/18888 | | | 0.150943396226415 | | | 13.4482022071912 | | | | 19.4195928449559 | | 5.86075170032081e-27 | | | 7.84168577502925e-24 | | | | 6.209312196182e-24 | | SERPING1/C1R/C1S/C3/CD74/IGHA2/IGHA1/IGHV3-7/IGHV3-74/IGHV3-72/IGHV3-15/IGHM/IGLC2/IGHV5-51/IGHV3-49/IGKC/IGHV3-23/IGHV2-5/IGHV4-59/IGHV3-30/IGHV6-1/IGHV4-39/IGHV3-11/IGHV4-34/IGHV3-48/IGLC3/IGHV1-24/IGHV4-4/IGHV3-21/IGHV1-18/IGHV1-3/IGHG1 | 32 |
| BP | GO:0002460 | | | adaptive immune response based on somatic recombination of immune receptors built from immunoglobulin superfamily domains | | | | 38/212 | | 385/18888 | | | 0.0987012987012987 | | | 8.79372702768929 | | | | 16.4612180999602 | | 5.30786044218964e-25 | | | 4.73461151443316e-22 | | | | 3.74902563864131e-22 | | SERPING1/C1R/C1S/C3/ANXA1/CD74/HLA-DRA/IL33/IL7R/IGHA2/IGHA1/CCL19/IGHV3-7/IGHV3-74/IGHV3-72/IGHV3-15/IGHM/IGLC2/IGHV5-51/IGHV3-49/IGKC/IGHV3-23/IGHV2-5/IGHV4-59/IGHV3-30/IGHV6-1/IGHV4-39/IGHV3-11/IGHV4-34/IGHV3-48/IGLC3/IGHV1-24/IGHV4-4/IGHV3-21/IGHV1-18/CXCL13/IGHV1-3/IGHG1 | 38 |
| BP | GO:0002377 | | | immunoglobulin production | | | | 29/212 | | 194/18888 | | | 0.149484536082474 | | | 13.318226026065 | | | | 18.3740715171938 | | 2.27568449790833e-24 | | | 1.52243292910067e-21 | | | | 1.20551391954986e-21 | | IL33/IGLV7-43/IGKV1-17/IGLV2-11/IGLV2-8/IGLV2-18/IGKV2-24/IGKV2-30/IGKV1-9/IGLV1-40/IGKV1-27/IGKV1-6/IGLV7-46/IGLV6-57/IGLV4-69/IGKV1-5/IGKV4-1/IGKV3-15/IGLV2-14/IGKV1-16/IGLV1-44/IGLV2-23/IGLV1-47/IGKV1-12/IGLV3-21/IGKV3-20/IGLV8-61/IGLV3-25/IGLV3-19 | 29 |
| BP | GO:0002449 | | | lymphocyte mediated immunity | | | | 34/212 | | 372/18888 | | | 0.0913978494623656 | | | 8.14303104077906 | | | | 14.8247681254897 | | 2.47274425452293e-21 | | | 1.32341272502067e-18 | | | | 1.04792298617993e-18 | | SERPING1/C1R/C1S/C3/CD74/HLA-DRA/IL7R/IGHA2/IGHA1/IGHV3-7/IGHV3-74/IGHV3-72/IGHV3-15/IGHM/IGLC2/IGHV5-51/IGHV3-49/IGKC/IGHV3-23/IGHV2-5/IGHV4-59/IGHV3-30/IGHV6-1/IGHV4-39/IGHV3-11/IGHV4-34/IGHV3-48/IGLC3/IGHV1-24/IGHV4-4/IGHV3-21/IGHV1-18/IGHV1-3/IGHG1 | 34 |
| BP | GO:0030198 | | | extracellular matrix organization | | | | 32/212 | | 332/18888 | | | 0.0963855421686747 | | | 8.58740622868834 | | | | 14.8603036824101 | | 8.16827876848868e-21 | | | 3.28066944623624e-18 | | | | 2.59774510080778e-18 | | FBLN5/CRISPLD2/MMP2/CCDC80/FBLN1/CCN1/DPT/COL16A1/MFAP4/LOX/EMILIN1/CAV1/COL6A1/ADAMTS2/COL8A2/CTSK/COL14A1/SFRP2/COL1A1/COL5A1/AEBP1/COL1A2/COL3A1/MMP3/LUM/ELN/COL5A2/FMOD/COL17A1/POSTN/MMP7/COMP | 32 |
| BP | GO:0043062 | | | extracellular structure organization | | | | 32/212 | | 333/18888 | | | 0.0960960960960961 | | | 8.56161822199558 | | | | 14.8324834327763 | | 8.95194991675062e-21 | | | 3.28066944623624e-18 | | | | 2.59774510080778e-18 | | FBLN5/CRISPLD2/MMP2/CCDC80/FBLN1/CCN1/DPT/COL16A1/MFAP4/LOX/EMILIN1/CAV1/COL6A1/ADAMTS2/COL8A2/CTSK/COL14A1/SFRP2/COL1A1/COL5A1/AEBP1/COL1A2/COL3A1/MMP3/LUM/ELN/COL5A2/FMOD/COL17A1/POSTN/MMP7/COMP | 32 |
| BP | GO:0045229 | | | external encapsulating structure organization | | | | 32/212 | | 334/18888 | | | 0.0958083832335329 | | | 8.53598463450458 | | | | 14.8047797075754 | | 9.80768145362104e-21 | | | 3.28066944623624e-18 | | | | 2.59774510080778e-18 | | FBLN5/CRISPLD2/MMP2/CCDC80/FBLN1/CCN1/DPT/COL16A1/MFAP4/LOX/EMILIN1/CAV1/COL6A1/ADAMTS2/COL8A2/CTSK/COL14A1/SFRP2/COL1A1/COL5A1/AEBP1/COL1A2/COL3A1/MMP3/LUM/ELN/COL5A2/FMOD/COL17A1/POSTN/MMP7/COMP | 32 |
| BP | GO:0002440 | | | production of molecular mediator of immune response | | | | 30/212 | | 310/18888 | | | 0.0967741935483871 | | | 8.62203286670724 | | | | 14.4164945719268 | | 1.31436473520147e-19 | | | 3.90804447933237e-17 | | | | 3.09452188533399e-17 | | CD74/IL33/IGLV7-43/IGKV1-17/IGLV2-11/IGLV2-8/IGLV2-18/IGKV2-24/IGKV2-30/IGKV1-9/IGLV1-40/IGKV1-27/IGKV1-6/IGLV7-46/IGLV6-57/IGLV4-69/IGKV1-5/IGKV4-1/IGKV3-15/IGLV2-14/IGKV1-16/IGLV1-44/IGLV2-23/IGLV1-47/IGKV1-12/IGLV3-21/IGKV3-20/IGLV8-61/IGLV3-25/IGLV3-19 | 30 |
| BP | GO:0002443 | | | leukocyte mediated immunity | | | | 34/212 | | 469/18888 | | | 0.0724946695095949 | | | 6.458864706 | | | | 12.7544934817452 | | 3.65679533920089e-18 | | | 9.78558432770158e-16 | | | | 7.7485568608541e-16 | | SERPING1/C1R/C1S/C3/CD74/HLA-DRA/IL7R/IGHA2/IGHA1/IGHV3-7/IGHV3-74/IGHV3-72/IGHV3-15/IGHM/IGLC2/IGHV5-51/IGHV3-49/IGKC/IGHV3-23/IGHV2-5/IGHV4-59/IGHV3-30/IGHV6-1/IGHV4-39/IGHV3-11/IGHV4-34/IGHV3-48/IGLC3/IGHV1-24/IGHV4-4/IGHV3-21/IGHV1-18/IGHV1-3/IGHG1 | 34 |
| BP | GO:0030199 | | | collagen fibril organization | | | | 16/212 | | 66/18888 | | | 0.242424242424242 | | | 21.598627787307 | | | | 17.8601400998225 | | 1.87029244076562e-17 | | | 4.54991142862618e-15 | | | | 3.60277385958009e-15 | | DPT/LOX/EMILIN1/COL6A1/ADAMTS2/COL14A1/SFRP2/COL1A1/COL5A1/AEBP1/COL1A2/COL3A1/LUM/COL5A2/FMOD/COMP | 16 |
| BP | GO:0071559 | | | response to transforming growth factor beta | | | | 20/212 | | 309/18888 | | | 0.0647249190938511 | | | 5.76662392379557 | | | | 9.00091061179195 | | 3.30275993200091e-10 | | | 7.36515464836203e-08 | | | | 5.83197872203318e-08 | | LOX/CAV1/LTBP2/ZFP36L2/CTSK/HTRA1/COL1A1/ACTA2/MXRA5/COL1A2/COL3A1/FBN1/FMOD/HTRA3/CILP/FOS/SFRP1/ASPN/CLDN1/POSTN | 20 |
| BP | GO:0071560 | | | cellular response to transforming growth factor beta stimulus | | | | 19/212 | | 302/18888 | | | 0.0629139072847682 | | | 5.60527302261652 | | | | 8.59554415104186 | | 1.4985351087038e-09 | | | 3.08467688530104e-07 | | | | 2.44255155774959e-07 | | LOX/CAV1/LTBP2/ZFP36L2/CTSK/HTRA1/COL1A1/ACTA2/COL1A2/COL3A1/FBN1/FMOD/HTRA3/CILP/FOS/SFRP1/ASPN/CLDN1/POSTN | 19 |
| BP | GO:0032963 | | | collagen metabolic process | | | | 12/212 | | 107/18888 | | | 0.11214953271028 | | | 9.99188855581026 | | | | 9.93781565001138 | | 2.84816631968994e-09 | | | 5.4440664796359e-07 | | | | 4.31079609138033e-07 | | MMP2/MRC2/MFAP4/EMILIN1/COL6A1/ADAMTS2/CTSK/COL1A1/COL5A1/COL1A2/MMP3/MMP7 | 12 |
| BP | GO:0019730 | | | antimicrobial humoral response | | | | 13/212 | | 132/18888 | | | 0.0984848484848485 | | | 8.77444253859348 | | | | 9.54978511894511 | | 3.09340618476496e-09 | | | 5.51863663362068e-07 | | | | 4.36984326311007e-07 | | RARRES2/IGHA2/IGHA1/LTF/JCHAIN/SLPI/IGHM/CXCL9/IGKV3-20/CXCL13/CXCL14/S100A9/IGHG1 | 13 |
| BP | GO:0043588 | | | skin development | | | | 19/212 | | 318/18888 | | | 0.059748427672956 | | | 5.32324670701317 | | | | 8.28371436724516 | | 3.51764975271542e-09 | | | 5.88326921141654e-07 | | | | 4.65857167908956e-07 | | ANXA1/COL6A1/ADAMTS2/COL1A1/COL5A1/COL1A2/COL3A1/ZFP36/NGFR/COL5A2/KRT7/CLDN1/KRT5/KRT14/KRT17/COMP/TFAP2B/KRT6B/KRT81 | 19 |
| BP | GO:0006959 | | | humoral immune response | | | | 17/212 | | 258/18888 | | | 0.0658914728682171 | | | 5.870557262 | | | | 8.39246554872557 | | 5.66878485236079e-09 | | | 8.92333427348087e-07 | | | | 7.06579808532649e-07 | | SERPING1/C1R/C1S/C3/RARRES2/IGHA2/IGHA1/LTF/JCHAIN/SLPI/IGHM/CXCL9/IGKV3-20/CXCL13/CXCL14/S100A9/IGHG1 | 17 |
| BP | GO:0007178 | | | transmembrane receptor protein serine/threonine kinase signaling pathway | | | | 21/212 | | 417/18888 | | | 0.0503597122302158 | | | 4.48676530473734 | | | | 7.67101844814233 | | 1.06190156198408e-08 | | | 1.578693655483e-06 | | | | 1.25006306682688e-06 | | FSTL1/CCN1/LOX/EMILIN1/LTBP2/EGR1/HTRA1/SFRP2/COL1A2/COL3A1/FBN1/SFRP4/FMOD/HTRA3/CILP/FOS/CHRDL1/SFRP1/ASPN/COMP/TFAP2B | 21 |
| BP | GO:0141091 | | | transforming growth factor beta receptor superfamily signaling pathway | | | | 20/212 | | 388/18888 | | | 0.0515463917525773 | | | 4.59249173312585 | | | | 7.61786485925708 | | 1.64450240985727e-08 | | | 2.31615181514635e-06 | | | | 1.83400739669955e-06 | | FSTL1/CCN1/LOX/LTBP2/EGR1/HTRA1/SFRP2/COL1A2/COL3A1/FBN1/SFRP4/FMOD/HTRA3/CILP/FOS/CHRDL1/SFRP1/ASPN/COMP/TFAP2B | 20 |
| BP | GO:0006958 | | | complement activation, classical pathway | | | | 8/212 | | 42/18888 | | | 0.19047619047619 | | | 16.9703504043127 | | | | 11.0391843225537 | | 1.87698724742185e-08 | | | 2.51140893705044e-06 | | | | 1.98861859424221e-06 | | SERPING1/C1R/C1S/C3/IGHA2/IGHA1/IGHM/IGHG1 | 8 |
| BP | GO:0061448 | | | connective tissue development | | | | 17/212 | | 288/18888 | | | 0.0590277777777778 | | | 5.25904088050314 | | | | 7.75995259951298 | | 2.89879318415213e-08 | | | 3.69389074323385e-06 | | | | 2.92494770912192e-06 | | CCN1/CCN2/LOX/PRRX1/BGN/COL6A1/EGR1/CTSK/SFRP2/COL1A1/COL5A1/ACTA2/COL3A1/OGN/ID4/COMP/CHI3L1 | 17 |
| BP | GO:0045785 | | | positive regulation of cell adhesion | | | | 22/212 | | 485/18888 | | | 0.045360824742268 | | | 4.04139272515075 | | | | 7.22947145082602 | | 3.04968986168321e-08 | | | 3.70953184993831e-06 | | | | 2.9373328667791e-06 | | FBLN2/CCDC80/CCN1/ANXA1/COL16A1/EMILIN1/CAV1/HLA-DPB1/SFRP2/CXCL12/CD74/CX3CL1/HLA-DRA/SAA1/CCL21/IL7R/CCL19/SFRP1/HLA-DRB5/CCL5/VTCN1/CXCL13 | 22 |
| BP | GO:0001503 | | | ossification | | | | 21/212 | | 446/18888 | | | 0.047085201793722 | | | 4.19502495981047 | | | | 7.27520380097748 | | 3.39099915048447e-08 | | | 3.94535379421584e-06 | | | | 3.12406466358134e-06 | | MMP2/TMEM119/CCN1/CCN2/MRC2/LOX/COL6A1/CTSK/SFRP2/COL1A1/COL1A2/TNC/COL5A2/VCAN/CHRDL1/ID4/SFRP1/LTF/ASPN/PTN/COMP | 21 |
| BP | GO:0048251 | | | elastic fiber assembly | | | | 5/212 | | 10/18888 | | | 0.5 | | | 44.5471698113208 | | | | 14.6753825830412 | | 4.09099178412931e-08 | | | 4.56145583930418e-06 | | | | 3.61191511467206e-06 | | FBLN5/MFAP4/LOX/EMILIN1/COL3A1 | 5 |
| BP | GO:0071356 | | | cellular response to tumor necrosis factor | | | | 15/212 | | 241/18888 | | | 0.0622406639004149 | | | 5.54529084788225 | | | | 7.56611960485086 | | 9.75217683908653e-08 | | | 1.04387300885582e-05 | | | | 8.26573977982366e-06 | | ZFP36L2/CTSK/COL1A1/ZFP36/CX3CL1/GSTP1/CCL21/FOS/CCL19/SFRP1/CLDN1/POSTN/CCL5/CHI3L1/FABP4 | 15 |
| BP | GO:0085029 | | | extracellular matrix assembly | | | | 8/212 | | 55/18888 | | | 0.145454545454545 | | | 12.9591766723842 | | | | 9.46303314594278 | | 1.70861403506543e-07 | | | 1.75855813762889e-05 | | | | 1.39248585124968e-05 | | FBLN5/MFAP4/LOX/EMILIN1/COL6A1/COL1A2/COL3A1/ELN | 8 |
| BP | GO:0002455 | | | humoral immune response mediated by circulating immunoglobulin | | | | 8/212 | | 56/18888 | | | 0.142857142857143 | | | 12.7277628032345 | | | | 9.36415195311425 | | 1.97430834687344e-07 | | | 1.95675893934568e-05 | | | | 1.549427954096e-05 | | SERPING1/C1R/C1S/C3/IGHA2/IGHA1/IGHM/IGHG1 | 8 |
| BP | GO:0071621 | | | granulocyte chemotaxis | | | | 11/212 | | 128/18888 | | | 0.0859375 | | | 7.65654481132075 | | | | 8.05090896247889 | | 2.12258535010584e-07 | | | 2.02858514174401e-05 | | | | 1.60630237209137e-05 | | ANXA1/RARRES2/CD74/CX3CL1/SAA1/CCL21/CCL19/CCL5/CXCL9/CXCL13/S100A9 | 11 |
| BP | GO:0034612 | | | response to tumor necrosis factor | | | | 15/212 | | 263/18888 | | | 0.0570342205323194 | | | 5.0814262142191 | | | | 7.10148495127328 | | 3.02397060012087e-07 | | | 2.7903949399736e-05 | | | | 2.20952915355474e-05 | | ZFP36L2/CTSK/COL1A1/ZFP36/CX3CL1/GSTP1/CCL21/FOS/CCL19/SFRP1/CLDN1/POSTN/CCL5/CHI3L1/FABP4 | 15 |
| BP | GO:0030509 | | | BMP signaling pathway | | | | 12/212 | | 168/18888 | | | 0.0714285714285714 | | | 6.36388140161725 | | | | 7.44026447505362 | | 4.45836084870812e-07 | | | 3.97685787704765e-05 | | | | 3.14901066261384e-05 | | FSTL1/CCN1/EGR1/HTRA1/SFRP2/FBN1/SFRP4/HTRA3/CHRDL1/SFRP1/COMP/TFAP2B | 12 |
| BP | GO:0030595 | | | leukocyte chemotaxis | | | | 14/212 | | 237/18888 | | | 0.0590717299578059 | | | 5.26295677095773 | | | | 7.03625432484381 | | 4.97391877781549e-07 | | | 4.2936150482046e-05 | | | | 3.3998297112878e-05 | | MMP2/ANXA1/RARRES2/CXCL12/CD74/CX3CL1/SAA1/CCL21/CCL19/PTN/CCL5/CXCL9/CXCL13/S100A9 | 14 |
| BP | GO:0097529 | | | myeloid leukocyte migration | | | | 14/212 | | 239/18888 | | | 0.0585774058577406 | | | 5.21891529170285 | | | | 6.99325669217108 | | 5.50426595425389e-07 | | | 4.60294240424482e-05 | | | | 3.64476558089246e-05 | | MMP2/ANXA1/EMILIN1/RARRES2/CXCL12/CD74/CX3CL1/SAA1/CCL21/CCL19/CCL5/CXCL9/CXCL13/S100A9 | 14 |
| BP | GO:0050851 | | | antigen receptor-mediated signaling pathway | | | | 13/212 | | 208/18888 | | | 0.0625 | | | 5.56839622641509 | | | | 7.05853355569945 | | 6.7975327313969e-07 | | | 5.51218108764185e-05 | | | | 4.36473154331801e-05 | | HLA-DPB1/IGHA2/IGHA1/TRAC/TRBC2/CD3D/TRBC1/IGHM/CD79A/VTCN1/IGKC/IGLC3/IGHG1 | 13 |
| BP | GO:0060326 | | | cell chemotaxis | | | | 16/212 | | 322/18888 | | | 0.0496894409937888 | | | 4.42704793155983 | | | | 6.60840162632666 | | 7.69129695017524e-07 | | | 6.05350312902027e-05 | | | | 4.79336865656432e-05 | | MMP2/ANXA1/LOX/RARRES2/CXCL12/CD74/CX3CL1/SAA1/CCL21/CCL19/PTN/CCL5/CXCL9/CXCL13/CXCL14/S100A9 | 16 |
| BP | GO:0006956 | | | complement activation | | | | 8/212 | | 67/18888 | | | 0.119402985074627 | | | 10.638130104196 | | | | 8.42008210759285 | | 8.15587334948892e-07 | | | 6.23574773806639e-05 | | | | 4.93767610602141e-05 | | SERPING1/C1R/C1S/C3/IGHA2/IGHA1/IGHM/IGHG1 | 8 |
| BP | GO:0071772 | | | response to BMP | | | | 12/212 | | 180/18888 | | | 0.0666666666666667 | | | 5.93962264150943 | | | | 7.09453331669574 | | 9.3229871149012e-07 | | | 6.74278743769611e-05 | | | | 5.33916730079548e-05 | | FSTL1/CCN1/EGR1/HTRA1/SFRP2/FBN1/SFRP4/HTRA3/CHRDL1/SFRP1/COMP/TFAP2B | 12 |
| BP | GO:0071773 | | | cellular response to BMP stimulus | | | | 12/212 | | 180/18888 | | | 0.0666666666666667 | | | 5.93962264150943 | | | | 7.09453331669574 | | 9.3229871149012e-07 | | | 6.74278743769611e-05 | | | | 5.33916730079548e-05 | | FSTL1/CCN1/EGR1/HTRA1/SFRP2/FBN1/SFRP4/HTRA3/CHRDL1/SFRP1/COMP/TFAP2B | 12 |
| BP | GO:0019731 | | | antibacterial humoral response | | | | 8/212 | | 69/18888 | | | 0.115942028985507 | | | 10.3297785069729 | | | | 8.27189657576288 | | 1.0256633479355e-06 | | | 7.22282926072473e-05 | | | | 5.71928066314172e-05 | | IGHA2/IGHA1/LTF/JCHAIN/SLPI/IGHM/IGKV3-20/IGHG1 | 8 |
| BP | GO:0030510 | | | regulation of BMP signaling pathway | | | | 10/212 | | 121/18888 | | | 0.0826446280991736 | | | 7.36316856385467 | | | | 7.48128456436555 | | 1.09871332348098e-06 | | | 7.53886372726947e-05 | | | | 5.96952744984401e-05 | | FSTL1/CCN1/HTRA1/SFRP2/FBN1/SFRP4/HTRA3/CHRDL1/SFRP1/TFAP2B | 10 |
| BP | GO:0090287 | | | regulation of cellular response to growth factor stimulus | | | | 17/212 | | 373/18888 | | | 0.0455764075067024 | | | 4.06059992918205 | | | | 6.36072485215671 | | 1.13864324536802e-06 | | | 7.61752331151204e-05 | | | | 6.03181277085742e-05 | | FSTL1/CCN1/LOX/EMILIN1/DCN/HTRA1/SFRP2/FBN1/NGFR/SFRP4/HTRA3/CILP/CHRDL1/SFRP1/ASPN/TFAP2B/CXCL13 | 17 |
| BP | GO:0097530 | | | granulocyte migration | | | | 11/212 | | 154/18888 | | | 0.0714285714285714 | | | 6.36388140161725 | | | | 7.12084868443024 | | 1.35862401943466e-06 | | | 8.768582817974e-05 | | | | 6.94326064533934e-05 | | ANXA1/RARRES2/CD74/CX3CL1/SAA1/CCL21/CCL19/CCL5/CXCL9/CXCL13/S100A9 | 11 |
| BP | GO:0071774 | | | response to fibroblast growth factor | | | | 10/212 | | 124/18888 | | | 0.0806451612903226 | | | 7.1850273889227 | | | | 7.36202428268739 | | 1.3762349714309e-06 | | | 8.768582817974e-05 | | | | 6.94326064533934e-05 | | CCN2/ZFP36L2/COL1A1/ZFP36/NGFR/TNC/SFRP1/POSTN/CCL5/CXCL13 | 10 |
| BP | GO:1990868 | | | response to chemokine | | | | 9/212 | | 101/18888 | | | 0.0891089108910891 | | | 7.93909957033439 | | | | 7.44976505474255 | | 2.04016386598577e-06 | | | 0.000124079056940407 | | | | 9.82499967040513e-05 | | LOX/CXCL12/CX3CL1/CCL21/ACKR1/CCL19/CCL5/CXCL9/CXCL13 | 9 |
| BP | GO:1990869 | | | cellular response to chemokine | | | | 9/212 | | 101/18888 | | | 0.0891089108910891 | | | 7.93909957033439 | | | | 7.44976505474255 | | 2.04016386598577e-06 | | | 0.000124079056940407 | | | | 9.82499967040513e-05 | | LOX/CXCL12/CX3CL1/CCL21/ACKR1/CCL19/CCL5/CXCL9/CXCL13 | 9 |
| BP | GO:0002685 | | | regulation of leukocyte migration | | | | 13/212 | | 230/18888 | | | 0.0565217391304348 | | | 5.0357670221493 | | | | 6.56092312103999 | | 2.10070709284417e-06 | | | 0.000124922048454467 | | | | 9.891750591568e-05 | | ANXA1/EMILIN1/RARRES2/CXCL12/CD74/CX3CL1/IL33/CCL21/CCL19/PTN/CCL5/APOD/CXCL13 | 13 |
| BP | GO:0050900 | | | leukocyte migration | | | | 17/212 | | 396/18888 | | | 0.0429292929292929 | | | 3.82475700400229 | | | | 6.05263148592631 | | 2.56574642031782e-06 | | | 0.00014608377491001 | | | | 0.000115674077135493 | | MMP2/ANXA1/EMILIN1/RARRES2/CXCL12/CD74/CX3CL1/SAA1/IL33/CCL21/CCL19/PTN/CCL5/CXCL9/APOD/CXCL13/S100A9 | 17 |
| BP | GO:2000146 | | | negative regulation of cell motility | | | | 17/212 | | 396/18888 | | | 0.0429292929292929 | | | 3.82475700400229 | | | | 6.05263148592631 | | 2.56574642031782e-06 | | | 0.00014608377491001 | | | | 0.000115674077135493 | | SERPINF1/FBLN1/DPYSL3/EMILIN1/DCN/PODN/SFRP2/CXCL12/COL3A1/CD74/CX3CL1/NGFR/IL33/CCL21/SFRP1/APOD/CXCL13 | 17 |
| BP | GO:0031589 | | | cell-substrate adhesion | | | | 16/212 | | 359/18888 | | | 0.0445682451253482 | | | 3.97077836758291 | | | | 6.05478845920463 | | 3.16776514767481e-06 | | | 0.000176602906982871 | | | | 0.000139840158821697 | | FBLN5/FBLN2/CCDC80/FBLN1/CCN1/CCN2/COL16A1/EMILIN1/COL1A1/COL3A1/CX3CL1/CCL21/SFRP1/COL17A1/POSTN/APOD | 16 |
| BP | GO:0030593 | | | neutrophil chemotaxis | | | | 9/212 | | 107/18888 | | | 0.0841121495327103 | | | 7.4939164168577 | | | | 7.17706228194005 | | 3.30531436785401e-06 | | | 0.000178689275628513 | | | | 0.000141492216127841 | | CD74/CX3CL1/SAA1/CCL21/CCL19/CCL5/CXCL9/CXCL13/S100A9 | 9 |
| BP | GO:2000107 | | | negative regulation of leukocyte apoptotic process | | | | 7/212 | | 57/18888 | | | 0.12280701754386 | | | 10.9414101290963 | | | | 8.00859284818556 | | 3.33873833386609e-06 | | | 0.000178689275628513 | | | | 0.000141492216127841 | | CXCL12/CD74/CCL21/IL7R/CCL19/CCL5/PIP | 7 |
| BP | GO:0090288 | | | negative regulation of cellular response to growth factor stimulus | | | | 10/212 | | 137/18888 | | | 0.072992700729927 | | | 6.50323646880595 | | | | 6.88769217056196 | | 3.40796155624253e-06 | | | 0.000178817747539314 | | | | 0.000141593944534907 | | EMILIN1/DCN/HTRA1/SFRP2/FBN1/NGFR/HTRA3/CHRDL1/SFRP1/CXCL13 | 10 |
| BP | GO:0051216 | | | cartilage development | | | | 12/212 | | 209/18888 | | | 0.0574162679425837 | | | 5.11546447594114 | | | | 6.37415584089645 | | 4.4429739159889e-06 | | | 0.000228642273061275 | | | | 0.000181046690139386 | | CCN1/CCN2/PRRX1/BGN/COL6A1/CTSK/SFRP2/COL1A1/COL3A1/OGN/COMP/CHI3L1 | 12 |
| BP | GO:0002429 | | | immune response-activating cell surface receptor signaling pathway | | | | 15/212 | | 332/18888 | | | 0.0451807228915663 | | | 4.02534666969766 | | | | 5.9252884188919 | | 5.50303814945812e-06 | | | 0.000277851511093395 | | | | 0.000220012230285187 | | C3/HLA-DPB1/IGHA2/IGHA1/TRAC/TRBC2/LTF/CD3D/TRBC1/IGHM/CD79A/VTCN1/IGKC/IGLC3/IGHG1 | 15 |
| BP | GO:0009612 | | | response to mechanical stimulus | | | | 12/212 | | 216/18888 | | | 0.0555555555555556 | | | 4.9496855345912 | | | | 6.22015867555691 | | 6.22599942000085e-06 | | | 0.000308532860146709 | | | | 0.0002443067608667 | | MMP2/COL6A1/COL1A1/CXCL12/TNC/IL33/FOS/PTN/FOSB/KRT5/POSTN/CHI3L1 | 12 |
| BP | GO:0044344 | | | cellular response to fibroblast growth factor stimulus | | | | 9/212 | | 116/18888 | | | 0.0775862068965517 | | | 6.91249186727391 | | | | 6.80537037515919 | | 6.4424550124715e-06 | | | 0.000313454720243159 | | | | 0.000248204056269955 | | CCN2/ZFP36L2/COL1A1/ZFP36/NGFR/SFRP1/POSTN/CCL5/CXCL13 | 9 |
| BP | GO:0030336 | | | negative regulation of cell migration | | | | 16/212 | | 381/18888 | | | 0.041994750656168 | | | 3.74149457732878 | | | | 5.75956063198288 | | 6.73849857806781e-06 | | | 0.00032200396776624 | | | | 0.000254973639805461 | | SERPINF1/DPYSL3/EMILIN1/DCN/PODN/SFRP2/CXCL12/COL3A1/CD74/CX3CL1/NGFR/IL33/CCL21/SFRP1/APOD/CXCL13 | 16 |
| BP | GO:0010810 | | | regulation of cell-substrate adhesion | | | | 12/212 | | 221/18888 | | | 0.0542986425339367 | | | 4.83770169896696 | | | | 6.11417092498144 | | 7.85824789193983e-06 | | | 0.000364119922996852 | | | | 0.00028832247855899 | | FBLN2/CCDC80/FBLN1/CCN1/COL16A1/EMILIN1/COL1A1/CX3CL1/CCL21/SFRP1/POSTN/APOD | 12 |
| BP | GO:0040013 | | | negative regulation of locomotion | | | | 17/212 | | 431/18888 | | | 0.0394431554524362 | | | 3.514161888 | | | | 5.62546990399758 | | 7.89198637287646e-06 | | | 0.000364119922996852 | | | | 0.00028832247855899 | | SERPINF1/FBLN1/DPYSL3/EMILIN1/DCN/PODN/SFRP2/CXCL12/COL3A1/CD74/CX3CL1/NGFR/IL33/CCL21/SFRP1/APOD/CXCL13 | 17 |
| BP | GO:0060740 | | | prostate gland epithelium morphogenesis | | | | 5/212 | | 26/18888 | | | 0.192307692307692 | | | 17.133526850508 | | | | 8.77060612495834 | | 9.22422490369489e-06 | | | 0.000418373319360805 | | | | 0.00033128215398997 | | MMP2/TNC/ID4/SFRP1/ESR1 | 5 |
| BP | GO:0002690 | | | positive regulation of leukocyte chemotaxis | | | | 8/212 | | 93/18888 | | | 0.0860215053763441 | | | 7.66402921485088 | | | | 6.86380109354169 | | 9.92324811021304e-06 | | | 0.000435321507261149 | | | | 0.000344702302775821 | | RARRES2/CXCL12/CD74/CCL21/CCL19/PTN/CCL5/CXCL13 | 8 |
| BP | GO:0070098 | | | chemokine-mediated signaling pathway | | | | 8/212 | | 93/18888 | | | 0.0860215053763441 | | | 7.66402921485088 | | | | 6.86380109354169 | | 9.92324811021304e-06 | | | 0.000435321507261149 | | | | 0.000344702302775821 | | CXCL12/CX3CL1/CCL21/ACKR1/CCL19/CCL5/CXCL9/CXCL13 | 8 |
| BP | GO:0030574 | | | collagen catabolic process | | | | 6/212 | | 45/18888 | | | 0.133333333333333 | | | 11.8792452830189 | | | | 7.78462128319804 | | 1.05312441468098e-05 | | | 0.000454542086078436 | | | | 0.000359921807598101 | | MMP2/MRC2/ADAMTS2/CTSK/MMP3/MMP7 | 6 |
| BP | GO:0030282 | | | bone mineralization | | | | 9/212 | | 127/18888 | | | 0.0708661417322835 | | | 6.31377209924231 | | | | 6.40153730806954 | | 1.34670402240725e-05 | | | 0.000565815209553873 | | | | 0.000448031632771594 | | TMEM119/CCN1/LOX/COL6A1/COL1A2/LTF/ASPN/PTN/COMP | 9 |
| BP | GO:0060512 | | | prostate gland morphogenesis | | | | 5/212 | | 28/18888 | | | 0.178571428571429 | | | 15.9097035040431 | | | | 8.41171726194916 | | 1.35322023211689e-05 | | | 0.000565815209553873 | | | | 0.000448031632771594 | | MMP2/TNC/ID4/SFRP1/ESR1 | 5 |
| BP | GO:0002768 | | | immune response-regulating cell surface receptor signaling pathway | | | | 15/212 | | 360/18888 | | | 0.0416666666666667 | | | 3.7122641509434 | | | | 5.53574977561633 | | 1.44344310297222e-05 | | | 0.00058669117933872 | | | | 0.000464561932188201 | | C3/HLA-DPB1/IGHA2/IGHA1/TRAC/TRBC2/LTF/CD3D/TRBC1/IGHM/CD79A/VTCN1/IGKC/IGLC3/IGHG1 | 15 |
| BP | GO:0002757 | | | immune response-activating signaling pathway | | | | 18/212 | | 500/18888 | | | 0.036 | | | 3.20739622641509 | | | | 5.32973404788783 | | 1.46842748822762e-05 | | | 0.00058669117933872 | | | | 0.000464561932188201 | | C3/CAV1/HLA-DPB1/IGHA2/IGHA1/TRAC/TRBC2/LTF/CD3D/TRBC1/IGHM/CD79A/VTCN1/IGKC/ESR1/IGLC3/S100A9/IGHG1 | 18 |
| BP | GO:0090092 | | | regulation of transmembrane receptor protein serine/threonine kinase signaling pathway | | | | 14/212 | | 317/18888 | | | 0.0441640378548896 | | | 3.93476578775073 | | | | 5.61426517733577 | | 1.46892036680472e-05 | | | 0.00058669117933872 | | | | 0.000464561932188201 | | FSTL1/CCN1/LOX/EMILIN1/HTRA1/SFRP2/FBN1/SFRP4/HTRA3/CILP/CHRDL1/SFRP1/ASPN/TFAP2B | 14 |
| BP | GO:1990266 | | | neutrophil migration | | | | 9/212 | | 129/18888 | | | 0.0697674418604651 | | | 6.21588415971918 | | | | 6.33323260524331 | | 1.52741962031334e-05 | | | 0.000601084544699779 | | | | 0.00047595908602024 | | CD74/CX3CL1/SAA1/CCL21/CCL19/CCL5/CXCL9/CXCL13/S100A9 | 9 |
| BP | GO:0030850 | | | prostate gland development | | | | 6/212 | | 49/18888 | | | 0.122448979591837 | | | 10.909510974201 | | | | 7.39995072596997 | | 1.74178626514282e-05 | | | 0.000675510151524955 | | | | 0.000534891800416857 | | SERPINF1/MMP2/TNC/ID4/SFRP1/ESR1 | 6 |
| BP | GO:0022409 | | | positive regulation of cell-cell adhesion | | | | 14/212 | | 325/18888 | | | 0.0430769230769231 | | | 3.83791001451379 | | | | 5.49824017345272 | | 1.93890268335553e-05 | | | 0.000741214797237056 | | | | 0.000586918962645816 | | ANXA1/EMILIN1/CAV1/HLA-DPB1/CD74/CX3CL1/HLA-DRA/CCL21/IL7R/CCL19/HLA-DRB5/CCL5/VTCN1/CXCL13 | 14 |
| BP | GO:0006935 | | | chemotaxis | | | | 17/212 | | 466/18888 | | | 0.036480686695279 | | | 3.25022269009636 | | | | 5.24031697084215 | | 2.15980343126569e-05 | | | 0.000814032955220701 | | | | 0.000644578844646082 | | MMP2/CCN1/ANXA1/LOX/RARRES2/CXCL12/CD74/CX3CL1/SAA1/CCL21/CCL19/PTN/CCL5/CXCL9/CXCL13/CXCL14/S100A9 | 17 |
| BP | GO:0042330 | | | taxis | | | | 17/212 | | 468/18888 | | | 0.0363247863247863 | | | 3.2363328495404 | | | | 5.21941758947439 | | 2.28049769089494e-05 | | | 0.000841605618487754 | | | | 0.000666411812609491 | | MMP2/CCN1/ANXA1/LOX/RARRES2/CXCL12/CD74/CX3CL1/SAA1/CCL21/CCL19/PTN/CCL5/CXCL9/CXCL13/CXCL14/S100A9 | 17 |
| BP | GO:0042742 | | | defense response to bacterium | | | | 14/212 | | 330/18888 | | | 0.0424242424242424 | | | 3.77975986277873 | | | | 5.42757898870843 | | 2.29585987106151e-05 | | | 0.000841605618487754 | | | | 0.000666411812609491 | | EMILIN1/RARRES2/IL7R/IGHA2/IGHA1/LTF/PLA2G2A/JCHAIN/SLPI/IGHM/IGKV3-20/CXCL13/S100A9/IGHG1 | 14 |
| BP | GO:0070371 | | | ERK1 and ERK2 cascade | | | | 14/212 | | 332/18888 | | | 0.0421686746987952 | | | 3.75699022505115 | | | | 5.39969928574377 | | 2.4540997765072e-05 | | | 0.000887455540801793 | | | | 0.000702717332874679 | | FBLN1/CCN1/CCN2/EMILIN1/ZFP36L2/ACTA2/CD74/CX3CL1/GSTP1/CCL21/CCL19/PLA2G2A/CCL5/CHI3L1 | 14 |
| BP | GO:0002468 | | | dendritic cell antigen processing and presentation | | | | 4/212 | | 16/18888 | | | 0.25 | | | 22.2735849056604 | | | | 9.06983103121363 | | 2.52584813553272e-05 | | | 0.000901222614758076 | | | | 0.000713618567975771 | | CD74/HLA-DRA/CCL21/CCL19 | 4 |
| BP | GO:0050853 | | | B cell receptor signaling pathway | | | | 7/212 | | 78/18888 | | | 0.0897435897435897 | | | 7.99564586357039 | | | | 6.59611483517904 | | 2.73401463653005e-05 | | | 0.000962660943072949 | | | | 0.000762267515697367 | | IGHA2/IGHA1/IGHM/CD79A/IGKC/IGLC3/IGHG1 | 7 |
| BP | GO:0048545 | | | response to steroid hormone | | | | 14/212 | | 338/18888 | | | 0.0414201183431953 | | | 3.69029809087864 | | | | 5.31733821059666 | | 2.98791181114042e-05 | | | 0.00103839636449504 | | | | 0.000822237385622101 | | SERPINF1/ANXA1/LOX/CAV1/FIBIN/ZFP36L2/COL1A1/ZFP36/FOS/SFRP1/FOSB/CLDN1/ESR1/SCGB2A2 | 14 |
| BP | GO:0060348 | | | bone development | | | | 11/212 | | 215/18888 | | | 0.0511627906976744 | | | 4.55831505046073 | | | | 5.59067268972967 | | 3.26968023636365e-05 | | | 0.00112175183493707 | | | | 0.000888241068259113 | | TMEM119/LOX/BGN/COL6A1/COL1A1/COL3A1/FBN1/SFRP4/OGN/LTF/COMP | 11 |
| BP | GO:0008544 | | | epidermis development | | | | 15/212 | | 387/18888 | | | 0.0387596899224806 | | | 3.45326897762176 | | | | 5.19529886683382 | | 3.34383855456716e-05 | | | 0.00113267240152174 | | | | 0.000896888342484169 | | CCN2/ANXA1/COL6A1/LAMB3/ZFP36/NGFR/SFRP4/KRT7/COL17A1/CLDN1/KRT5/KRT14/KRT17/KRT6B/KRT81 | 15 |
| BP | GO:0001649 | | | osteoblast differentiation | | | | 12/212 | | 257/18888 | | | 0.046692607003891 | | | 4.1600469862712 | | | | 5.4343737885523 | | 3.52888509827165e-05 | | | 0.00117580432961188 | | | | 0.000931041662933174 | | TMEM119/CCN1/MRC2/LOX/COL6A1/SFRP2/COL1A1/TNC/VCAN/ID4/SFRP1/LTF | 12 |
| BP | GO:1901654 | | | response to ketone | | | | 11/212 | | 217/18888 | | | 0.0506912442396313 | | | 4.51630293017999 | | | | 5.55059893501241 | | 3.5590489797669e-05 | | | 0.00117580432961188 | | | | 0.000931041662933174 | | SERPINF1/CAV1/FIBIN/TNC/CCL21/FOS/CCL19/SFRP1/FOSB/CLDN1/POSTN | 11 |
| BP | GO:0031214 | | | biomineral tissue development | | | | 10/212 | | 180/18888 | | | 0.0555555555555556 | | | 4.9496855345912 | | | | 5.67273608719751 | | 3.73411772635641e-05 | | | 0.00121859744338168 | | | | 0.000964926698736257 | | TMEM119/CCN1/LOX/COL6A1/COL1A1/COL1A2/LTF/ASPN/PTN/COMP | 10 |
| BP | GO:0070372 | | | regulation of ERK1 and ERK2 cascade | | | | 13/212 | | 308/18888 | | | 0.0422077922077922 | | | 3.76047537368292 | | | | 5.20407885436752 | | 4.75650592093694e-05 | | | 0.00152657320795046 | | | | 0.00120879233246963 | | FBLN1/CCN1/CCN2/EMILIN1/ACTA2/CD74/CX3CL1/GSTP1/CCL21/CCL19/PLA2G2A/CCL5/CHI3L1 | 13 |
| BP | GO:0002687 | | | positive regulation of leukocyte migration | | | | 9/212 | | 149/18888 | | | 0.0604026845637584 | | | 5.38153729264278 | | | | 5.72076264293215 | | 4.79193383661581e-05 | | | 0.00152657320795046 | | | | 0.00120879233246963 | | RARRES2/CXCL12/CD74/CX3CL1/CCL21/CCL19/PTN/CCL5/CXCL13 | 9 |
| BP | GO:0007162 | | | negative regulation of cell adhesion | | | | 13/212 | | 310/18888 | | | 0.0419354838709677 | | | 3.73621424223981 | | | | 5.17534081270896 | | 5.08215551512894e-05 | | | 0.00159998213629236 | | | | 0.001266920006434 | | MMP2/FBLN1/ANXA1/COL1A1/CXCL12/CD74/CX3CL1/TNC/CCL21/PLA2G2A/POSTN/VTCN1/APOD | 13 |
| BP | GO:0071230 | | | cellular response to amino acid stimulus | | | | 7/212 | | 86/18888 | | | 0.0813953488372093 | | | 7.2518648530057 | | | | 6.19104941912878 | | 5.1551401093853e-05 | | | 0.00160408778287385 | | | | 0.00127017099635161 | | MMP2/COL16A1/COL6A1/COL1A1/COL1A2/COL3A1/COL5A2 | 7 |
| BP | GO:0060263 | | | regulation of respiratory burst | | | | 4/212 | | 19/18888 | | | 0.210526315789474 | | | 18.7567030784508 | | | | 8.2503463161815 | | 5.23887439063158e-05 | | | 0.00161140550222185 | | | | 0.00127596541419738 | | IGHA2/IGHA1/JCHAIN/S100A9 | 4 |
| BP | GO:1905517 | | | macrophage migration | | | | 6/212 | | 60/18888 | | | 0.1 | | | 8.90943396226415 | | | | 6.5377215992003 | | 5.62802178958925e-05 | | | 0.00171143026237964 | | | | 0.00135516840459847 | | MMP2/EMILIN1/RARRES2/CX3CL1/SAA1/CCL5 | 6 |
| BP | GO:0033627 | | | cell adhesion mediated by integrin | | | | 7/212 | | 88/18888 | | | 0.0795454545454545 | | | 7.08704974271012 | | | | 6.09785018519593 | | 5.97659139169462e-05 | | | 0.00179700658024436 | | | | 0.00142293062938868 | | COL16A1/EMILIN1/SFRP2/FBN1/CCL21/CCL5/CXCL13 | 7 |
| BP | GO:2000106 | | | regulation of leukocyte apoptotic process | | | | 7/212 | | 89/18888 | | | 0.0786516853932584 | | | 7.00741997032012 | | | | 6.05233713019922 | | 6.42574690764776e-05 | | | 0.00191058874720727 | | | | 0.00151286883334444 | | CXCL12/CD74/CCL21/IL7R/CCL19/CCL5/PIP | 7 |
| BP | GO:0002688 | | | regulation of leukocyte chemotaxis | | | | 8/212 | | 123/18888 | | | 0.0650406504065041 | | | 5.79475379659457 | | | | 5.68397384775485 | | 7.53078751339516e-05 | | | 0.00221454806437862 | | | | 0.00175355410809305 | | RARRES2/CXCL12/CD74/CCL21/CCL19/PTN/CCL5/CXCL13 | 8 |
| BP | GO:0010811 | | | positive regulation of cell-substrate adhesion | | | | 8/212 | | 124/18888 | | | 0.0645161290322581 | | | 5.74802191113816 | | | | 5.65155987298212 | | 7.97360556599673e-05 | | | 0.00231927918419644 | | | | 0.00183648375335829 | | FBLN2/CCDC80/CCN1/COL16A1/EMILIN1/CX3CL1/CCL21/SFRP1 | 8 |
| BP | GO:0048247 | | | lymphocyte chemotaxis | | | | 6/212 | | 64/18888 | | | 0.09375 | | | 8.35259433962264 | | | | 6.27743353975236 | | 8.1202564290228e-05 | | | 0.00233653830151237 | | | | 0.00185015010657871 | | CX3CL1/SAA1/CCL21/CCL19/CCL5/CXCL13 | 6 |
| BP | GO:0072676 | | | lymphocyte migration | | | | 8/212 | | 125/18888 | | | 0.064 | | | 5.70203773584906 | | | | 5.61949728097423 | | 8.43781470760601e-05 | | | 0.00240208427208018 | | | | 0.00190205162445811 | | CXCL12/CX3CL1/SAA1/CCL21/CCL19/CCL5/APOD/CXCL13 | 8 |
| BP | GO:0031099 | | | regeneration | | | | 10/212 | | 200/18888 | | | 0.05 | | | 4.45471698113208 | | | | 5.23303389601341 | | 9.05314531704103e-05 | | | 0.00255012809141071 | | | | 0.00201927773110289 | | NNMT/MMP2/PRRX1/COL6A1/CXCL12/TNC/PTN/CLDN1/POSTN/APOD | 10 |
| BP | GO:0007160 | | | cell-matrix adhesion | | | | 11/212 | | 241/18888 | | | 0.045643153526971 | | | 4.06654662178032 | | | | 5.10459270720849 | | 9.16793934619859e-05 | | | 0.00255556309275286 | | | | 0.0020235813491116 | | FBLN5/CCN2/COL16A1/EMILIN1/COL3A1/CX3CL1/CCL21/SFRP1/COL17A1/POSTN/APOD | 11 |
| BP | GO:0033630 | | | positive regulation of cell adhesion mediated by integrin | | | | 4/212 | | 22/18888 | | | 0.181818181818182 | | | 16.1989708404803 | | | | 7.59964131595583 | | 9.62938704668319e-05 | | | 0.00263195705165768 | | | | 0.00208407267130317 | | SFRP2/CCL21/CCL5/CXCL13 | 4 |
| BP | GO:0001655 | | | urogenital system development | | | | 6/212 | | 66/18888 | | | 0.0909090909090909 | | | 8.09948542024014 | | | | 6.1556432569877 | | 9.65919168993772e-05 | | | 0.00263195705165768 | | | | 0.00208407267130317 | | SERPINF1/MMP2/TNC/ID4/SFRP1/ESR1 | 6 |
| BP | GO:0071229 | | | cellular response to acid chemical | | | | 7/212 | | 95/18888 | | | 0.0736842105263158 | | | 6.56484607745779 | | | | 5.7932782769361 | | 9.73706084133448e-05 | | | 0.00263195705165768 | | | | 0.00208407267130317 | | MMP2/COL16A1/COL6A1/COL1A1/COL1A2/COL3A1/COL5A2 | 7 |
| BP | GO:0000302 | | | response to reactive oxygen species | | | | 10/212 | | 203/18888 | | | 0.0492610837438424 | | | 4.38888372525328 | | | | 5.17208464755955 | | 0.000102407181195609 | | | 0.00274041616879451 | | | | 0.00216995427101854 | | FBLN5/MMP2/COL6A1/SOD3/COL1A1/MMP3/GSTP1/FOS/CRYAB/APOD | 10 |
| BP | GO:0007179 | | | transforming growth factor beta receptor signaling pathway | | | | 11/212 | | 245/18888 | | | 0.0448979591836735 | | | 4.0001540238737 | | | | 5.03588938105569 | | 0.000106089401331743 | | | 0.00281084394023509 | | | | 0.0022257213640521 | | LOX/LTBP2/HTRA1/COL1A2/COL3A1/FBN1/FMOD/HTRA3/CILP/FOS/ASPN | 11 |
| BP | GO:0071674 | | | mononuclear cell migration | | | | 10/212 | | 206/18888 | | | 0.0485436893203883 | | | 4.32496794284668 | | | | 5.11230642273892 | | 0.000115576896637853 | | | 0.00303219387649897 | | | | 0.00240099373510834 | | ANXA1/RARRES2/CXCL12/CX3CL1/SAA1/CCL21/CCL19/CCL5/APOD/CXCL13 | 10 |
| BP | GO:0002548 | | | monocyte chemotaxis | | | | 6/212 | | 70/18888 | | | 0.0857142857142857 | | | 7.6366576819407 | | | | 5.92678527567478 | | 0.000134298822526003 | | | 0.00348916164154937 | | | | 0.00276283627741282 | | ANXA1/CXCL12/CX3CL1/CCL21/CCL19/CCL5 | 6 |
| BP | GO:0051384 | | | response to glucocorticoid | | | | 8/212 | | 134/18888 | | | 0.0597014925373134 | | | 5.319065052 | | | | 5.34567653997947 | | 0.00013717194191462 | | | 0.00352953958234156 | | | | 0.00279480889751143 | | SERPINF1/ANXA1/FIBIN/ZFP36L2/ZFP36/FOS/FOSB/CLDN1 | 8 |
| BP | GO:0050870 | | | positive regulation of T cell activation | | | | 11/212 | | 253/18888 | | | 0.0434782608695652 | | | 3.87366694011485 | | | | 4.90274699011935 | | 0.000140780448889875 | | | 0.00358789029742196 | | | | 0.00284101296857463 | | ANXA1/CAV1/HLA-DPB1/CD74/HLA-DRA/CCL21/IL7R/CCL19/HLA-DRB5/CCL5/VTCN1 | 11 |
| BP | GO:0030514 | | | negative regulation of BMP signaling pathway | | | | 6/212 | | 72/18888 | | | 0.0833333333333333 | | | 7.42452830188679 | | | | 5.81903984085255 | | 0.000157083799588551 | | | 0.00391104890442024 | | | | 0.00309690089080243 | | HTRA1/SFRP2/FBN1/HTRA3/CHRDL1/SFRP1 | 6 |
| BP | GO:0014012 | | | peripheral nervous system axon regeneration | | | | 3/212 | | 10/18888 | | | 0.3 | | | 26.7283018867925 | | | | 8.67042967514512 | | 0.00015784502304835 | | | 0.00391104890442024 | | | | 0.00309690089080243 | | MMP2/TNC/APOD | 3 |
| BP | GO:0060346 | | | bone trabecula formation | | | | 3/212 | | 10/18888 | | | 0.3 | | | 26.7283018867925 | | | | 8.67042967514512 | | 0.00015784502304835 | | | 0.00391104890442024 | | | | 0.00309690089080243 | | MMP2/COL1A1/SFRP1 | 3 |
| BP | GO:0022407 | | | regulation of cell-cell adhesion | | | | 16/212 | | 496/18888 | | | 0.032258064516129 | | | 2.87401095556908 | | | | 4.50615340558113 | | 0.000159314506651486 | | | 0.0039112442183429 | | | | 0.00309705554697674 | | ANXA1/EMILIN1/CAV1/HLA-DPB1/CXCL12/CD74/CX3CL1/HLA-DRA/CCL21/IL7R/CCL19/HLA-DRB5/PLA2G2A/CCL5/VTCN1/CXCL13 | 16 |
| BP | GO:0070555 | | | response to interleukin-1 | | | | 8/212 | | 138/18888 | | | 0.0579710144927536 | | | 5.16488925348646 | | | | 5.23178457332487 | | 0.000168118349626402 | | | 0.00408795864332176 | | | | 0.00323698400952195 | | MMP2/EGR1/CX3CL1/CCL21/CCL19/SFRP1/CCL5/CHI3L1 | 8 |
| BP | GO:0045109 | | | intermediate filament organization | | | | 6/212 | | 73/18888 | | | 0.0821917808219178 | | | 7.32282243473766 | | | | 5.76670575676267 | | 0.000169567791258862 | | | 0.00408795864332176 | | | | 0.00323698400952195 | | KRT7/KRT5/KRT14/KRT17/KRT6B/KRT81 | 6 |
| BP | GO:0032642 | | | regulation of chemokine production | | | | 7/212 | | 104/18888 | | | 0.0673076923076923 | | | 5.99673439767779 | | | | 5.44397826868225 | | 0.000171943032770048 | | | 0.00410821031868436 | | | | 0.00325301997148596 | | EGR1/CD74/GSTP1/IL33/ACKR1/POSTN/APOD | 7 |
| BP | GO:0032602 | | | chemokine production | | | | 7/212 | | 105/18888 | | | 0.0666666666666667 | | | 5.93962264150943 | | | | 5.4077104644778 | | 0.00018249482036464 | | | 0.00432173574598033 | | | | 0.00342209663152325 | | EGR1/CD74/GSTP1/IL33/ACKR1/POSTN/APOD | 7 |
| BP | GO:0033622 | | | integrin activation | | | | 4/212 | | 26/18888 | | | 0.153846153846154 | | | 13.7068214804064 | | | | 6.90775968190089 | | 0.000189998723370028 | | | 0.00442118768467996 | | | | 0.00350084604250679 | | COL16A1/CXCL12/CX3CL1/CXCL13 | 4 |
| BP | GO:0060343 | | | trabecula formation | | | | 4/212 | | 26/18888 | | | 0.153846153846154 | | | 13.7068214804064 | | | | 6.90775968190089 | | 0.000189998723370028 | | | 0.00442118768467996 | | | | 0.00350084604250679 | | MMP2/COL1A1/SFRP1/SLC40A1 | 4 |
| BP | GO:0050921 | | | positive regulation of chemotaxis | | | | 8/212 | | 142/18888 | | | 0.0563380281690141 | | | 5.01939941536009 | | | | 5.12222343580564 | | 0.000204595023776184 | | | 0.00471979554849197 | | | | 0.00373729385536713 | | RARRES2/CXCL12/CD74/CCL21/CCL19/PTN/CCL5/CXCL13 | 8 |
| BP | GO:2001235 | | | positive regulation of apoptotic signaling pathway | | | | 8/212 | | 143/18888 | | | 0.0559440559440559 | | | 4.98429872014778 | | | | 5.09547501308017 | | 0.000214662547406249 | | | 0.004881250680319 | | | | 0.00386513949314851 | | MMP2/SRPX/CAV1/CTSK/NGFR/SFRP1/G0S2/S100A9 | 8 |
| BP | GO:0002604 | | | regulation of dendritic cell antigen processing and presentation | | | | 3/212 | | 11/18888 | | | 0.272727272727273 | | | 24.2984562607204 | | | | 8.23501678291193 | | 0.000215241995619448 | | | 0.004881250680319 | | | | 0.00386513949314851 | | CD74/CCL21/CCL19 | 3 |
| BP | GO:2000404 | | | regulation of T cell migration | | | | 5/212 | | 50/18888 | | | 0.1 | | | 8.90943396226415 | | | | 5.96651172292117 | | 0.000238766303173494 | | | 0.00536923216211991 | | | | 0.00425153974602603 | | CXCL12/CCL21/CCL5/APOD/CXCL13 | 5 |
| BP | GO:0048144 | | | fibroblast proliferation | | | | 7/212 | | 110/18888 | | | 0.0636363636363636 | | | 5.6696397941681 | | | | 5.23314230327856 | | 0.000243394823800137 | | | 0.00538284750817493 | | | | 0.00426232083783972 | | CAV1/COL3A1/CD74/NGFR/GSTP1/SFRP1/ESR1 | 7 |
| BP | GO:0071347 | | | cellular response to interleukin-1 | | | | 7/212 | | 110/18888 | | | 0.0636363636363636 | | | 5.6696397941681 | | | | 5.23314230327856 | | 0.000243394823800137 | | | 0.00538284750817493 | | | | 0.00426232083783972 | | MMP2/EGR1/CX3CL1/CCL21/CCL19/SFRP1/CCL5 | 7 |
| BP | GO:0035987 | | | endodermal cell differentiation | | | | 5/212 | | 51/18888 | | | 0.0980392156862745 | | | 8.73473917869034 | | | | 5.89294490414891 | | 0.000262325967601157 | | | 0.0057071893439081 | | | | 0.00451914568062584 | | MMP2/COL6A1/LAMB3/COL5A1/COL5A2 | 5 |
| BP | GO:0071622 | | | regulation of granulocyte chemotaxis | | | | 5/212 | | 51/18888 | | | 0.0980392156862745 | | | 8.73473917869034 | | | | 5.89294490414891 | | 0.000262325967601157 | | | 0.0057071893439081 | | | | 0.00451914568062584 | | RARRES2/CD74/CCL21/CCL19/CCL5 | 5 |
| BP | GO:0048705 | | | skeletal system morphogenesis | | | | 10/212 | | 229/18888 | | | 0.0436681222707424 | | | 3.89058251627255 | | | | 4.68885085066012 | | 0.000272717359737969 | | | 0.00588541656982907 | | | | 0.00466027203015731 | | MMP2/TMEM119/CCN2/PRRX1/COL1A1/COL3A1/SFRP4/SFRP1/LTF/COMP | 10 |
| BP | GO:0097048 | | | dendritic cell apoptotic process | | | | 3/212 | | 12/18888 | | | 0.25 | | | 22.2735849056604 | | | | 7.85387179453593 | | 0.000284617152474231 | | | 0.00604472619064319 | | | | 0.00478641877970448 | | CXCL12/CCL21/CCL19 | 3 |
| BP | GO:2000668 | | | regulation of dendritic cell apoptotic process | | | | 3/212 | | 12/18888 | | | 0.25 | | | 22.2735849056604 | | | | 7.85387179453593 | | 0.000284617152474231 | | | 0.00604472619064319 | | | | 0.00478641877970448 | | CXCL12/CCL21/CCL19 | 3 |
| BP | GO:0007159 | | | leukocyte cell-cell adhesion | | | | 14/212 | | 419/18888 | | | 0.0334128878281623 | | | 2.97689917593552 | | | | 4.35990804060639 | | 0.00028803929445872 | | | 0.0060692374170987 | | | | 0.0048058275983871 | | ANXA1/CAV1/HLA-DPB1/CXCL12/CD74/HLA-DRA/CCL21/IL7R/CCL19/HLA-DRB5/PLA2G2A/CCL5/VTCN1/S100A9 | 14 |
| BP | GO:0003094 | | | glomerular filtration | | | | 4/212 | | 29/18888 | | | 0.137931034482759 | | | 12.2888744307092 | | | | 6.48183242444159 | | 0.000294001244719844 | | | 0.00609881651837443 | | | | 0.00482924933187308 | | IGHA2/IGHA1/JCHAIN/IGKV3-20 | 4 |
| BP | GO:0003401 | | | axis elongation | | | | 4/212 | | 29/18888 | | | 0.137931034482759 | | | 12.2888744307092 | | | | 6.48183242444159 | | 0.000294001244719844 | | | 0.00609881651837443 | | | | 0.00482924933187308 | | SFRP2/TNC/SFRP1/ESR1 | 4 |
| BP | GO:1903039 | | | positive regulation of leukocyte cell-cell adhesion | | | | 11/212 | | 277/18888 | | | 0.0397111913357401 | | | 3.53804236768613 | | | | 4.53378984609714 | | 0.000308020871925726 | | | 0.00634049117902495 | | | | 0.00502061550758289 | | ANXA1/CAV1/HLA-DPB1/CD74/HLA-DRA/CCL21/IL7R/CCL19/HLA-DRB5/CCL5/VTCN1 | 11 |
| BP | GO:0050727 | | | regulation of inflammatory response | | | | 14/212 | | 425/18888 | | | 0.0329411764705882 | | | 2.93487236403996 | | | | 4.29836350046371 | | 0.000332441020366606 | | | 0.00678123649641173 | | | | 0.00536961256678342 | | C3/ANXA1/ZFP36/MMP3/CX3CL1/TNC/SAA1/GSTP1/IL33/PLA2G2A/CCL5/FABP4/ESR1/S100A9 | 14 |
| BP | GO:0071295 | | | cellular response to vitamin | | | | 4/212 | | 30/18888 | | | 0.133333333333333 | | | 11.8792452830189 | | | | 6.3535882726256 | | 0.000336268096676817 | | | 0.00678123649641173 | | | | 0.00536961256678342 | | COL1A1/TNC/SFRP1/POSTN | 4 |
| BP | GO:0071887 | | | leukocyte apoptotic process | | | | 7/212 | | 116/18888 | | | 0.0603448275862069 | | | 5.37638256343526 | | | | 5.03728460286198 | | 0.000337034549335859 | | | 0.00678123649641173 | | | | 0.00536961256678342 | | CXCL12/CD74/CCL21/IL7R/CCL19/CCL5/PIP | 7 |
| BP | GO:0033690 | | | positive regulation of osteoblast proliferation | | | | 3/212 | | 13/18888 | | | 0.230769230769231 | | | 20.5602322206096 | | | | 7.51639665244657 | | 0.00036694557288162 | | | 0.00727367668912011 | | | | 0.00575954337786122 | | TMEM119/CCN1/LTF | 3 |
| BP | GO:0061430 | | | bone trabecula morphogenesis | | | | 3/212 | | 13/18888 | | | 0.230769230769231 | | | 20.5602322206096 | | | | 7.51639665244657 | | 0.00036694557288162 | | | 0.00727367668912011 | | | | 0.00575954337786122 | | MMP2/COL1A1/SFRP1 | 3 |
| BP | GO:0042060 | | | wound healing | | | | 14/212 | | 430/18888 | | | 0.0325581395348837 | | | 2.90074594120228 | | | | 4.24789200186752 | | 0.000373806637033679 | | | 0.00731529081006285 | | | | 0.00579249484724118 | | SERPING1/FBLN1/CCN1/ANXA1/EMILIN1/CAV1/MYL9/COL5A1/COL3A1/CX3CL1/SAA1/F13A1/CLDN1/COMP | 14 |
| BP | GO:0002686 | | | negative regulation of leukocyte migration | | | | 5/212 | | 55/18888 | | | 0.0909090909090909 | | | 8.09948542024014 | | | | 5.61766647414836 | | 0.00037463224408165 | | | 0.00731529081006285 | | | | 0.00579249484724118 | | EMILIN1/CXCL12/IL33/CCL21/APOD | 5 |
| BP | GO:0050867 | | | positive regulation of cell activation | | | | 13/212 | | 380/18888 | | | 0.0342105263157895 | | | 3.04796425024826 | | | | 4.29676807045867 | | 0.000378937468719765 | | | 0.00731529081006285 | | | | 0.00579249484724118 | | ANXA1/CAV1/HLA-DPB1/ACTA2/CD74/HLA-DRA/IL33/CCL21/IL7R/CCL19/HLA-DRB5/CCL5/VTCN1 | 13 |
| BP | GO:0019886 | | | antigen processing and presentation of exogenous peptide antigen via MHC class II | | | | 4/212 | | 31/18888 | | | 0.129032258064516 | | | 11.4960438222763 | | | | 6.23128578382412 | | 0.000382713271079521 | | | 0.00731529081006285 | | | | 0.00579249484724118 | | HLA-DPB1/CD74/HLA-DRA/HLA-DRB5 | 4 |
| BP | GO:0033688 | | | regulation of osteoblast proliferation | | | | 4/212 | | 31/18888 | | | 0.129032258064516 | | | 11.4960438222763 | | | | 6.23128578382412 | | 0.000382713271079521 | | | 0.00731529081006285 | | | | 0.00579249484724118 | | TMEM119/CCN1/SFRP1/LTF | 4 |
| BP | GO:0031960 | | | response to corticosteroid | | | | 8/212 | | 156/18888 | | | 0.0512820512820513 | | | 4.5689404934688 | | | | 4.76888686396103 | | 0.000386905862580755 | | | 0.00734297934940497 | | | | 0.00581441956980262 | | SERPINF1/ANXA1/FIBIN/ZFP36L2/ZFP36/FOS/FOSB/CLDN1 | 8 |
| BP | GO:1903037 | | | regulation of leukocyte cell-cell adhesion | | | | 13/212 | | 382/18888 | | | 0.0340314136125654 | | | 3.03200632223649 | | | | 4.2747226798993 | | 0.00039828047768277 | | | 0.00750562364985276 | | | | 0.00594320683154496 | | ANXA1/CAV1/HLA-DPB1/CXCL12/CD74/HLA-DRA/CCL21/IL7R/CCL19/HLA-DRB5/PLA2G2A/CCL5/VTCN1 | 13 |
| BP | GO:0090101 | | | negative regulation of transmembrane receptor protein serine/threonine kinase signaling pathway | | | | 9/212 | | 198/18888 | | | 0.0454545454545455 | | | 4.04974271012007 | | | | 4.59619699336765 | | 0.000411134128855358 | | | 0.00769367083088768 | | | | 0.00609210895388911 | | EMILIN1/HTRA1/SFRP2/FBN1/HTRA3/CILP/CHRDL1/SFRP1/ASPN | 9 |
| BP | GO:0034694 | | | response to prostaglandin | | | | 4/212 | | 32/18888 | | | 0.125 | | | 11.1367924528302 | | | | 6.1144619529699 | | 0.000433567515771747 | | | 0.00800156325658755 | | | | 0.00633590859708549 | | TNC/CCL21/CCL19/SFRP1 | 4 |
| BP | GO:0097205 | | | renal filtration | | | | 4/212 | | 32/18888 | | | 0.125 | | | 11.1367924528302 | | | | 6.1144619529699 | | 0.000433567515771747 | | | 0.00800156325658755 | | | | 0.00633590859708549 | | IGHA2/IGHA1/JCHAIN/IGKV3-20 | 4 |
| BP | GO:0043200 | | | response to amino acid | | | | 7/212 | | 124/18888 | | | 0.0564516129032258 | | | 5.02951917224589 | | | | 4.79632766812949 | | 0.000504634608496882 | | | 0.0092493302214908 | | | | 0.00732393271019628 | | MMP2/COL16A1/COL6A1/COL1A1/COL1A2/COL3A1/COL5A2 | 7 |
| BP | GO:0022612 | | | gland morphogenesis | | | | 7/212 | | 125/18888 | | | 0.056 | | | 4.98928301886792 | | | | 4.76767017555995 | | 0.000529565076547271 | | | 0.00957743301274591 | | | | 0.00758373560485308 | | MMP2/CAV1/TNC/ID4/SFRP1/PTN/ESR1 | 7 |
| BP | GO:0048145 | | | regulation of fibroblast proliferation | | | | 6/212 | | 90/18888 | | | 0.0666666666666667 | | | 5.93962264150943 | | | | 5.00456912993936 | | 0.000529693604591328 | | | 0.00957743301274591 | | | | 0.00758373560485308 | | CAV1/CD74/NGFR/GSTP1/SFRP1/ESR1 | 6 |
| BP | GO:2001233 | | | regulation of apoptotic signaling pathway | | | | 13/212 | | 396/18888 | | | 0.0328282828282828 | | | 2.92481417953116 | | | | 4.12431614155873 | | 0.00055869576533628 | | | 0.0100277200812569 | | | | 0.00794028814553152 | | MMP2/SRPX/CAV1/CTSK/SFRP2/CXCL12/CD74/CX3CL1/NGFR/GSTP1/SFRP1/G0S2/S100A9 | 13 |
| BP | GO:0001706 | | | endoderm formation | | | | 5/212 | | 60/18888 | | | 0.0833333333333333 | | | 7.42452830188679 | | | | 5.31033922835806 | | 0.000562091932805882 | | | 0.0100277200812569 | | | | 0.00794028814553152 | | MMP2/COL6A1/LAMB3/COL5A1/COL5A2 | 5 |
| BP | GO:0010820 | | | positive regulation of T cell chemotaxis | | | | 3/212 | | 15/18888 | | | 0.2 | | | 17.8188679245283 | | | | 6.94271611875167 | | 0.000574178885786698 | | | 0.01017551455871 | | | | 0.00805731681483878 | | CCL21/CCL5/CXCL13 | 3 |
| BP | GO:0032496 | | | response to lipopolysaccharide | | | | 12/212 | | 348/18888 | | | 0.0344827586206897 | | | 3.07221860767729 | | | | 4.15697884367991 | | 0.000591049175325988 | | | 0.0104055762708707 | | | | 0.0082394874648976 | | COL6A1/ZFP36/MMP3/CX3CL1/GSTP1/FOS/LTF/CLDN1/SLPI/CXCL9/CXCL13/S100A9 | 12 |
| BP | GO:0031102 | | | neuron projection regeneration | | | | 5/212 | | 61/18888 | | | 0.0819672131147541 | | | 7.30281472316734 | | | | 5.25310866975901 | | 0.00060675717275692 | | | 0.0106123019235132 | | | | 0.00840317983322793 | | MMP2/PRRX1/TNC/PTN/APOD | 5 |
| BP | GO:0002495 | | | antigen processing and presentation of peptide antigen via MHC class II | | | | 4/212 | | 35/18888 | | | 0.114285714285714 | | | 10.1822102425876 | | | | 5.79293304422376 | | 0.000614919921558012 | | | 0.0106852318836964 | | | | 0.0084609282439937 | | HLA-DPB1/CD74/HLA-DRA/HLA-DRB5 | 4 |
| BP | GO:0070661 | | | leukocyte proliferation | | | | 12/212 | | 352/18888 | | | 0.0340909090909091 | | | 3.03730703259005 | | | | 4.11080895263009 | | 0.00065346531192408 | | | 0.0112817624174764 | | | | 0.00893328131003853 | | ANXA1/HLA-DPB1/CD74/CX3CL1/GSTP1/IL33/IL7R/CCL19/PLA2G2A/CCL5/CD79A/VTCN1 | 12 |
| BP | GO:0045104 | | | intermediate filament cytoskeleton organization | | | | 6/212 | | 94/18888 | | | 0.0638297872340425 | | | 5.68687274187073 | | | | 4.85338747730796 | | 0.000667512155520507 | | | 0.011450400821621 | | | | 0.00906681490595669 | | KRT7/KRT5/KRT14/KRT17/KRT6B/KRT81 | 6 |
| BP | GO:0033687 | | | osteoblast proliferation | | | | 4/212 | | 36/18888 | | | 0.111111111111111 | | | 9.89937106918239 | | | | 5.69428681597369 | | 0.000685753043827039 | | | 0.0116143996536782 | | | | 0.00919667473167109 | | TMEM119/CCN1/SFRP1/LTF | 4 |
| BP | GO:2000406 | | | positive regulation of T cell migration | | | | 4/212 | | 36/18888 | | | 0.111111111111111 | | | 9.89937106918239 | | | | 5.69428681597369 | | 0.000685753043827039 | | | 0.0116143996536782 | | | | 0.00919667473167109 | | CXCL12/CCL21/CCL5/CXCL13 | 4 |
| BP | GO:0002399 | | | MHC class II protein complex assembly | | | | 3/212 | | 16/18888 | | | 0.1875 | | | 16.7051886792453 | | | | 6.69578767592589 | | 0.000700852825270859 | | | 0.0116489575181666 | | | | 0.00922403881837358 | | HLA-DPB1/HLA-DRA/HLA-DRB5 | 3 |
| BP | GO:0002503 | | | peptide antigen assembly with MHC class II protein complex | | | | 3/212 | | 16/18888 | | | 0.1875 | | | 16.7051886792453 | | | | 6.69578767592589 | | 0.000700852825270859 | | | 0.0116489575181666 | | | | 0.00922403881837358 | | HLA-DPB1/HLA-DRA/HLA-DRB5 | 3 |
| BP | GO:0010819 | | | regulation of T cell chemotaxis | | | | 3/212 | | 16/18888 | | | 0.1875 | | | 16.7051886792453 | | | | 6.69578767592589 | | 0.000700852825270859 | | | 0.0116489575181666 | | | | 0.00922403881837358 | | CCL21/CCL5/CXCL13 | 3 |
| BP | GO:0045103 | | | intermediate filament-based process | | | | 6/212 | | 95/18888 | | | 0.0631578947368421 | | | 5.62701092353525 | | | | 4.81694578742212 | | 0.000705948664314247 | | | 0.0116612260845983 | | | | 0.00923375348450019 | | KRT7/KRT5/KRT14/KRT17/KRT6B/KRT81 | 6 |
| BP | GO:0070374 | | | positive regulation of ERK1 and ERK2 cascade | | | | 9/212 | | 215/18888 | | | 0.0418604651162791 | | | 3.7295304958315 | | | | 4.2885217813565 | | 0.000743955023507441 | | | 0.0122136419810179 | | | | 0.00967117508763629 | | CCN2/ACTA2/CD74/CX3CL1/CCL21/CCL19/PLA2G2A/CCL5/CHI3L1 | 9 |
| BP | GO:0002504 | | | antigen processing and presentation of peptide or polysaccharide antigen via MHC class II | | | | 4/212 | | 37/18888 | | | 0.108108108108108 | | | 9.63182049974503 | | | | 5.59942657132236 | | 0.000762172969270229 | | | 0.0123610597925281 | | | | 0.00978790550010188 | | HLA-DPB1/CD74/HLA-DRA/HLA-DRB5 | 4 |
| BP | GO:0070229 | | | negative regulation of lymphocyte apoptotic process | | | | 4/212 | | 37/18888 | | | 0.108108108108108 | | | 9.63182049974503 | | | | 5.59942657132236 | | 0.000762172969270229 | | | 0.0123610597925281 | | | | 0.00978790550010188 | | CD74/IL7R/CCL5/PIP | 4 |
| BP | GO:0010812 | | | negative regulation of cell-substrate adhesion | | | | 5/212 | | 65/18888 | | | 0.0769230769230769 | | | 6.85341074020319 | | | | 5.03649892160164 | | 0.000812470163623588 | | | 0.0130974105894983 | | | | 0.0103709729827158 | | FBLN1/COL1A1/CX3CL1/POSTN/APOD | 5 |
| BP | GO:0002696 | | | positive regulation of leukocyte activation | | | | 12/212 | | 362/18888 | | | 0.0331491712707182 | | | 2.95340352340248 | | | | 3.99818522692869 | | 0.000834121106952579 | | | 0.0133659166599108 | | | | 0.0105835851767762 | | ANXA1/CAV1/HLA-DPB1/CD74/HLA-DRA/IL33/CCL21/IL7R/CCL19/HLA-DRB5/CCL5/VTCN1 | 12 |
| BP | GO:0032905 | | | transforming growth factor beta1 production | | | | 3/212 | | 17/18888 | | | 0.176470588235294 | | | 15.7225305216426 | | | | 6.47018881710653 | | 0.000844019768442477 | | | 0.0134440291687623 | | | | 0.0106454373049794 | | COL3A1/CX3CL1/LUM | 3 |
| BP | GO:0060349 | | | bone morphogenesis | | | | 6/212 | | 99/18888 | | | 0.0606060606060606 | | | 5.39965694682676 | | | | 4.67618911647807 | | 0.000877105341043395 | | | 0.0138883662285925 | | | | 0.010997278427408 | | TMEM119/COL1A1/COL3A1/SFRP4/LTF/COMP | 6 |
| BP | GO:0030216 | | | keratinocyte differentiation | | | | 8/212 | | 177/18888 | | | 0.0451977401129944 | | | 4.02686280780301 | | | | 4.31060629542174 | | 0.000889615338724549 | | | 0.0140035920378053 | | | | 0.0110885181229258 | | ANXA1/ZFP36/KRT7/KRT5/KRT14/KRT17/KRT6B/KRT81 | 8 |
| BP | GO:0002237 | | | response to molecule of bacterial origin | | | | 12/212 | | 369/18888 | | | 0.032520325203252 | | | 2.89737689829729 | | | | 3.9216200444725 | | 0.000983991405727183 | | | 0.0153986023492745 | | | | 0.0121931345012547 | | COL6A1/ZFP36/MMP3/CX3CL1/GSTP1/FOS/LTF/CLDN1/SLPI/CXCL9/CXCL13/S100A9 | 12 |
| BP | GO:0001101 | | | response to acid chemical | | | | 7/212 | | 139/18888 | | | 0.0503597122302158 | | | 4.48676530473734 | | | | 4.39590754204584 | | 0.000993381037850835 | | | 0.0154551607981909 | | | | 0.0122379193953105 | | MMP2/COL16A1/COL6A1/COL1A1/COL1A2/COL3A1/COL5A2 | 7 |
| BP | GO:0048246 | | | macrophage chemotaxis | | | | 4/212 | | 40/18888 | | | 0.1 | | | 8.90943396226415 | | | | 5.33519443586188 | | 0.00102734067546878 | | | 0.0158911193500258 | | | | 0.0125831261315404 | | MMP2/RARRES2/SAA1/CCL5 | 4 |
| BP | GO:1903706 | | | regulation of hemopoiesis | | | | 13/212 | | 424/18888 | | | 0.0306603773584906 | | | 2.73166607333571 | | | | 3.84230352231027 | | 0.00104687576104696 | | | 0.0161002272216188 | | | | 0.0127487048214612 | | ANXA1/LOX/ZFP36L2/ZFP36/CD74/FBN1/HLA-DRA/IL7R/FOS/CCL19/SFRP1/LTF/PTN | 13 |
| BP | GO:0007596 | | | blood coagulation | | | | 9/212 | | 226/18888 | | | 0.0398230088495575 | | | 3.54800467523794 | | | | 4.10565862022653 | | 0.00105790473012894 | | | 0.0161768746161431 | | | | 0.0128093968225537 | | SERPING1/FBLN1/EMILIN1/CAV1/MYL9/COL3A1/SAA1/F13A1/COMP | 9 |
| BP | GO:0070167 | | | regulation of biomineral tissue development | | | | 6/212 | | 103/18888 | | | 0.058252427184466 | | | 5.18996153141601 | | | | 4.54287237673666 | | 0.00107859297028545 | | | 0.0163995158436583 | | | | 0.0129856916817261 | | TMEM119/CCN1/LTF/ASPN/PTN/COMP | 6 |
| BP | GO:0002478 | | | antigen processing and presentation of exogenous peptide antigen | | | | 4/212 | | 41/18888 | | | 0.0975609756097561 | | | 8.69213069489185 | | | | 5.25321233899991 | | 0.00112849615765943 | | | 0.0169162621728835 | | | | 0.0133948689143319 | | HLA-DPB1/CD74/HLA-DRA/HLA-DRB5 | 4 |
| BP | GO:0045730 | | | respiratory burst | | | | 4/212 | | 41/18888 | | | 0.0975609756097561 | | | 8.69213069489185 | | | | 5.25321233899991 | | 0.00112849615765943 | | | 0.0169162621728835 | | | | 0.0133948689143319 | | IGHA2/IGHA1/JCHAIN/S100A9 | 4 |
| BP | GO:0043627 | | | response to estrogen | | | | 5/212 | | 70/18888 | | | 0.0714285714285714 | | | 6.36388140161725 | | | | 4.79014811899635 | | 0.00113786516857961 | | | 0.0169162621728835 | | | | 0.0133948689143319 | | MMP2/CAV1/SFRP1/SLC34A2/ESR1 | 5 |
| BP | GO:2000401 | | | regulation of lymphocyte migration | | | | 5/212 | | 70/18888 | | | 0.0714285714285714 | | | 6.36388140161725 | | | | 4.79014811899635 | | 0.00113786516857961 | | | 0.0169162621728835 | | | | 0.0133948689143319 | | CXCL12/CCL21/CCL5/APOD/CXCL13 | 5 |
| BP | GO:0002523 | | | leukocyte migration involved in inflammatory response | | | | 3/212 | | 19/18888 | | | 0.157894736842105 | | | 14.0675273088381 | | | | 6.07160154211914 | | 0.00118299547417071 | | | 0.0173939334553891 | | | | 0.0137731052024618 | | CX3CL1/PTN/S100A9 | 3 |
| BP | GO:0002544 | | | chronic inflammatory response | | | | 3/212 | | 19/18888 | | | 0.157894736842105 | | | 14.0675273088381 | | | | 6.07160154211914 | | 0.00118299547417071 | | | 0.0173939334553891 | | | | 0.0137731052024618 | | CCL5/CXCL13/S100A9 | 3 |
| BP | GO:0050817 | | | coagulation | | | | 9/212 | | 231/18888 | | | 0.038961038961039 | | | 3.47120803724577 | | | | 4.02626058837762 | | 0.00123233437746233 | | | 0.0179820377351645 | | | | 0.0142387860754017 | | SERPING1/FBLN1/EMILIN1/CAV1/MYL9/COL3A1/SAA1/F13A1/COMP | 9 |
| BP | GO:0071364 | | | cellular response to epidermal growth factor stimulus | | | | 4/212 | | 42/18888 | | | 0.0952380952380952 | | | 8.48517520215633 | | | | 5.17397719219098 | | 0.00123643308791863 | | | 0.0179820377351645 | | | | 0.0142387860754017 | | ZFP36L2/COL1A1/ZFP36/FOS | 4 |
| BP | GO:0051251 | | | positive regulation of lymphocyte activation | | | | 11/212 | | 328/18888 | | | 0.0335365853658537 | | | 2.98791992636908 | | | | 3.86949518777251 | | 0.00124396128828083 | | | 0.0179937319321054 | | | | 0.0142480459363261 | | ANXA1/CAV1/HLA-DPB1/CD74/HLA-DRA/CCL21/IL7R/CCL19/HLA-DRB5/CCL5/VTCN1 | 11 |
| BP | GO:0042698 | | | ovulation cycle | | | | 5/212 | | 72/18888 | | | 0.0694444444444444 | | | 6.18710691823899 | | | | 4.69824092877449 | | 0.00129186054967827 | | | 0.0185861227469841 | | | | 0.0147171210328373 | | SERPINF1/MMP2/EGR1/PTN/ESR1 | 5 |
| BP | GO:0007599 | | | hemostasis | | | | 9/212 | | 233/18888 | | | 0.0386266094420601 | | | 3.44141226010203 | | | | 3.99511185304488 | | 0.00130830270053466 | | | 0.0187220215327848 | | | | 0.0148247302908881 | | SERPING1/FBLN1/EMILIN1/CAV1/MYL9/COL3A1/SAA1/F13A1/COMP | 9 |
| BP | GO:0050863 | | | regulation of T cell activation | | | | 12/212 | | 382/18888 | | | 0.031413612565445 | | | 2.79877506667984 | | | | 3.78407512291134 | | 0.00132195768457494 | | | 0.0188168019357582 | | | | 0.0148997806217769 | | ANXA1/CAV1/HLA-DPB1/CD74/HLA-DRA/CCL21/IL7R/CCL19/HLA-DRB5/PLA2G2A/CCL5/VTCN1 | 12 |
| BP | GO:2000403 | | | positive regulation of lymphocyte migration | | | | 4/212 | | 43/18888 | | | 0.0930232558139535 | | | 8.28784554629223 | | | | 5.09733063593707 | | 0.00135138994098026 | | | 0.0191339655135618 | | | | 0.0151509214769884 | | CXCL12/CCL21/CCL5/CXCL13 | 4 |
| BP | GO:0002577 | | | regulation of antigen processing and presentation | | | | 3/212 | | 20/18888 | | | 0.15 | | | 13.3641509433962 | | | | 5.89418618786581 | | 0.00138030436475751 | | | 0.0192379920838079 | | | | 0.0152332932360574 | | CD74/CCL21/CCL19 | 3 |
| BP | GO:0055093 | | | response to hyperoxia | | | | 3/212 | | 20/18888 | | | 0.15 | | | 13.3641509433962 | | | | 5.89418618786581 | | 0.00138030436475751 | | | 0.0192379920838079 | | | | 0.0152332932360574 | | MMP2/CAV1/COL1A1 | 3 |
| BP | GO:0140131 | | | positive regulation of lymphocyte chemotaxis | | | | 3/212 | | 20/18888 | | | 0.15 | | | 13.3641509433962 | | | | 5.89418618786581 | | 0.00138030436475751 | | | 0.0192379920838079 | | | | 0.0152332932360574 | | CCL21/CCL5/CXCL13 | 3 |
| BP | GO:0072678 | | | T cell migration | | | | 5/212 | | 74/18888 | | | 0.0675675675675676 | | | 6.01988781234064 | | | | 4.60974380535811 | | 0.00146072693736772 | | | 0.0202223888697064 | | | | 0.0160127719277472 | | CXCL12/CCL21/CCL5/APOD/CXCL13 | 5 |
| BP | GO:0030501 | | | positive regulation of bone mineralization | | | | 4/212 | | 44/18888 | | | 0.0909090909090909 | | | 8.09948542024014 | | | | 5.02312690176088 | | 0.00147360457010192 | | | 0.0202223888697064 | | | | 0.0160127719277472 | | TMEM119/CCN1/LTF/PTN | 4 |
| BP | GO:1905521 | | | regulation of macrophage migration | | | | 4/212 | | 44/18888 | | | 0.0909090909090909 | | | 8.09948542024014 | | | | 5.02312690176088 | | 0.00147360457010192 | | | 0.0202223888697064 | | | | 0.0160127719277472 | | EMILIN1/RARRES2/CX3CL1/CCL5 | 4 |
| BP | GO:0072593 | | | reactive oxygen species metabolic process | | | | 9/212 | | 238/18888 | | | 0.0378151260504202 | | | 3.36911368320913 | | | | 3.91870390366717 | | 0.00151485442949739 | | | 0.0206824002721174 | | | | 0.0163770245251248 | | FBLN5/CCN1/CCN2/COL6A1/SOD3/MMP3/GSTP1/GPX3/CRYAB | 9 |
| BP | GO:0030324 | | | lung development | | | | 8/212 | | 193/18888 | | | 0.0414507772020725 | | | 3.6930296216639 | | | | 4.0064943825792 | | 0.00154808064217731 | | | 0.0210287502460227 | | | | 0.0166512761565745 | | CRISPLD2/CCN2/LOX/COL6A1/ADAMTS2/COL3A1/TNC/CHI3L1 | 8 |
| BP | GO:0002396 | | | MHC protein complex assembly | | | | 3/212 | | 21/18888 | | | 0.142857142857143 | | | 12.7277628032345 | | | | 5.72902720182191 | | 0.00159710804114429 | | | 0.0214766890356891 | | | | 0.0170059692505869 | | HLA-DPB1/HLA-DRA/HLA-DRB5 | 3 |
| BP | GO:0002501 | | | peptide antigen assembly with MHC protein complex | | | | 3/212 | | 21/18888 | | | 0.142857142857143 | | | 12.7277628032345 | | | | 5.72902720182191 | | 0.00159710804114429 | | | 0.0214766890356891 | | | | 0.0170059692505869 | | HLA-DPB1/HLA-DRA/HLA-DRB5 | 3 |
| BP | GO:0060840 | | | artery development | | | | 6/212 | | 113/18888 | | | 0.0530973451327434 | | | 4.73067290031725 | | | | 4.23783369120648 | | 0.00173783577472202 | | | 0.0230606863637964 | | | | 0.0182602319448989 | | LOX/PRRX1/COL3A1/NGFR/COMP/TFAP2B | 6 |
| BP | GO:0061383 | | | trabecula morphogenesis | | | | 4/212 | | 46/18888 | | | 0.0869565217391304 | | | 7.7473338802297 | | | | 4.88152036101068 | | 0.00174075435182618 | | | 0.0230606863637964 | | | | 0.0182602319448989 | | MMP2/COL1A1/SFRP1/SLC40A1 | 4 |
| BP | GO:0070849 | | | response to epidermal growth factor | | | | 4/212 | | 46/18888 | | | 0.0869565217391304 | | | 7.7473338802297 | | | | 4.88152036101068 | | 0.00174075435182618 | | | 0.0230606863637964 | | | | 0.0182602319448989 | | ZFP36L2/COL1A1/ZFP36/FOS | 4 |
| BP | GO:0030323 | | | respiratory tube development | | | | 8/212 | | 197/18888 | | | 0.0406091370558376 | | | 3.61804424863519 | | | | 3.93551266903654 | | 0.00176162817862491 | | | 0.023222251261085 | | | | 0.0183881644986878 | | CRISPLD2/CCN2/LOX/COL6A1/ADAMTS2/COL3A1/TNC/CHI3L1 | 8 |
| BP | GO:0009913 | | | epidermal cell differentiation | | | | 9/212 | | 246/18888 | | | 0.0365853658536585 | | | 3.25954901058445 | | | | 3.80058592382428 | | 0.00189919495688445 | | | 0.0248018882496404 | | | | 0.0196389745285682 | | ANXA1/ZFP36/SFRP4/KRT7/KRT5/KRT14/KRT17/KRT6B/KRT81 | 9 |
| BP | GO:0032355 | | | response to estradiol | | | | 6/212 | | 115/18888 | | | 0.0521739130434783 | | | 4.64840032813782 | | | | 4.18111443573503 | | 0.00189999517607484 | | | 0.0248018882496404 | | | | 0.0196389745285682 | | MMP2/COL1A1/SFRP1/CRYAB/POSTN/ESR1 | 6 |
| BP | GO:0010720 | | | positive regulation of cell development | | | | 13/212 | | 454/18888 | | | 0.0286343612334802 | | | 2.55115950461308 | | | | 3.5643642666642 | | 0.00192377126031183 | | | 0.0249903489931769 | | | | 0.019788204123698 | | SERPINF1/ANXA1/CXCL12/CD74/CX3CL1/HLA-DRA/IL33/IL7R/VCAN/FOS/ID4/CCL19/PTN | 13 |
| BP | GO:2001236 | | | regulation of extrinsic apoptotic signaling pathway | | | | 7/212 | | 157/18888 | | | 0.0445859872611465 | | | 3.97235909145535 | | | | 3.98453923642623 | | 0.00200344224706258 | | | 0.0258147066470508 | | | | 0.0204409584141741 | | SRPX/CAV1/SFRP2/CX3CL1/GSTP1/SFRP1/G0S2 | 7 |
| BP | GO:0045444 | | | fat cell differentiation | | | | 9/212 | | 248/18888 | | | 0.0362903225806452 | | | 3.23326232501522 | | | | 3.77181265991378 | | 0.00200652428347779 | | | 0.0258147066470508 | | | | 0.0204409584141741 | | RARRES2/ZFP36L2/LAMB3/SFRP2/ZFP36/ID4/SFRP1/FABP4/TFAP2B | 9 |
| BP | GO:0031670 | | | cellular response to nutrient | | | | 4/212 | | 48/18888 | | | 0.0833333333333333 | | | 7.42452830188679 | | | | 4.74819891126302 | | 0.00203976593134298 | | | 0.0261168116376737 | | | | 0.0206801753704025 | | COL1A1/TNC/SFRP1/POSTN | 4 |
| BP | GO:0019882 | | | antigen processing and presentation | | | | 6/212 | | 117/18888 | | | 0.0512820512820513 | | | 4.5689404934688 | | | | 4.1256845701058 | | 0.00207336248631397 | | | 0.0262953460349582 | | | | 0.0208215449486157 | | HLA-DPB1/CD74/HLA-DRA/CCL21/CCL19/HLA-DRB5 | 6 |
| BP | GO:0051928 | | | positive regulation of calcium ion transport | | | | 6/212 | | 117/18888 | | | 0.0512820512820513 | | | 4.5689404934688 | | | | 4.1256845701058 | | 0.00207336248631397 | | | 0.0262953460349582 | | | | 0.0208215449486157 | | CAV1/CXCL12/CX3CL1/CCL5/CXCL9/STAC2 | 6 |
| BP | GO:0010560 | | | positive regulation of glycoprotein biosynthetic process | | | | 3/212 | | 23/18888 | | | 0.130434782608696 | | | 11.6210008203445 | | | | 5.43010770259542 | | 0.00209185871312365 | | | 0.026331206704424 | | | | 0.0208499406403924 | | IL33/CCL21/CCL19 | 3 |
| BP | GO:0043491 | | | phosphatidylinositol 3-kinase/protein kinase B signal transduction | | | | 10/212 | | 299/18888 | | | 0.0334448160535117 | | | 2.97974380008834 | | | | 3.67641102670933 | | 0.00209586959194406 | | | 0.026331206704424 | | | | 0.0208499406403924 | | COL6A2/LOX/DCN/COL6A1/CX3CL1/CCL21/COL6A3/CCL19/CCL5/CHI3L1 | 10 |
| BP | GO:0033628 | | | regulation of cell adhesion mediated by integrin | | | | 4/212 | | 49/18888 | | | 0.0816326530612245 | | | 7.273007316 | | | | 4.68438302998938 | | 0.00220180285033336 | | | 0.027532824427533 | | | | 0.0218014222219432 | | SFRP2/CCL21/CCL5/CXCL13 | 4 |
| BP | GO:0070482 | | | response to oxygen levels | | | | 11/212 | | 354/18888 | | | 0.0310734463276836 | | | 2.76846818036457 | | | | 3.57867112972989 | | 0.0022669070901255 | | | 0.0282150854566318 | | | | 0.0223416595957045 | | MMP2/CAV1/COL6A1/EGR1/SOD3/COL1A1/CXCL12/FOS/SFRP1/CRYAB/POSTN | 11 |
| BP | GO:0030500 | | | regulation of bone mineralization | | | | 5/212 | | 82/18888 | | | 0.0609756097560976 | | | 5.43258168430741 | | | | 4.28571163301851 | | 0.00230199732715843 | | | 0.0283877642740827 | | | | 0.0224783925276251 | | TMEM119/CCN1/LTF/PTN/COMP | 5 |
| BP | GO:0061844 | | | antimicrobial humoral immune response mediated by antimicrobial peptide | | | | 5/212 | | 82/18888 | | | 0.0609756097560976 | | | 5.43258168430741 | | | | 4.28571163301851 | | 0.00230199732715843 | | | 0.0283877642740827 | | | | 0.0224783925276251 | | LTF/CXCL9/CXCL13/CXCL14/S100A9 | 5 |
| BP | GO:0002407 | | | dendritic cell chemotaxis | | | | 3/212 | | 24/18888 | | | 0.125 | | | 11.1367924528302 | | | | 5.29415642989691 | | 0.00237106134438035 | | | 0.0287276674075777 | | | | 0.0227475393326465 | | CCL21/CCL19/CCL5 | 3 |
| BP | GO:0034695 | | | response to prostaglandin E | | | | 3/212 | | 24/18888 | | | 0.125 | | | 11.1367924528302 | | | | 5.29415642989691 | | 0.00237106134438035 | | | 0.0287276674075777 | | | | 0.0227475393326465 | | CCL21/CCL19/SFRP1 | 3 |
| BP | GO:0070233 | | | negative regulation of T cell apoptotic process | | | | 3/212 | | 24/18888 | | | 0.125 | | | 11.1367924528302 | | | | 5.29415642989691 | | 0.00237106134438035 | | | 0.0287276674075777 | | | | 0.0227475393326465 | | IL7R/CCL5/PIP | 3 |
| BP | GO:0019884 | | | antigen processing and presentation of exogenous antigen | | | | 4/212 | | 50/18888 | | | 0.08 | | | 7.12754716981132 | | | | 4.62233861435086 | | 0.00237250168052117 | | | 0.0287276674075777 | | | | 0.0227475393326465 | | HLA-DPB1/CD74/HLA-DRA/HLA-DRB5 | 4 |
| BP | GO:0031424 | | | keratinization | | | | 5/212 | | 83/18888 | | | 0.0602409638554217 | | | 5.36712889293021 | | | | 4.24820900642155 | | 0.00242759697290315 | | | 0.0292623851328325 | | | | 0.023170946924865 | | KRT7/KRT5/KRT17/KRT6B/KRT81 | 5 |
| BP | GO:0007517 | | | muscle organ development | | | | 11/212 | | 359/18888 | | | 0.0306406685236769 | | | 2.72991012771325 | | | | 3.52575607491765 | | 0.00252584424733554 | | | 0.0303101309680265 | | | | 0.0240005875378166 | | LOX/TAGLN/CAV1/COL6A1/EGR1/FHL1/COL3A1/ELN/COL6A3/FOS/CRYAB | 11 |
| BP | GO:2001238 | | | positive regulation of extrinsic apoptotic signaling pathway | | | | 4/212 | | 51/18888 | | | 0.0784313725490196 | | | 6.98779134295228 | | | | 4.56197987629264 | | 0.00255209110289735 | | | 0.0304353066727054 | | | | 0.0240997058973157 | | SRPX/CAV1/SFRP1/G0S2 | 4 |
| BP | GO:0030278 | | | regulation of ossification | | | | 6/212 | | 122/18888 | | | 0.0491803278688525 | | | 4.3816888339004 | | | | 3.99241051583138 | | 0.0025590224220324 | | | 0.0304353066727054 | | | | 0.0240997058973157 | | TMEM119/CCN1/SFRP1/LTF/PTN/COMP | 6 |
| BP | GO:0071675 | | | regulation of mononuclear cell migration | | | | 6/212 | | 123/18888 | | | 0.048780487804878 | | | 4.34606534744593 | | | | 3.96661616729297 | | 0.00266560570522027 | | | 0.0313397636471543 | | | | 0.0248158855407606 | | RARRES2/CXCL12/CCL21/CCL5/APOD/CXCL13 | 6 |
| BP | GO:0090023 | | | positive regulation of neutrophil chemotaxis | | | | 3/212 | | 25/18888 | | | 0.12 | | | 10.691320754717 | | | | 5.16600803901029 | | 0.00267227144651391 | | | 0.0313397636471543 | | | | 0.0248158855407606 | | CD74/CCL21/CCL19 | 3 |
| BP | GO:1905523 | | | positive regulation of macrophage migration | | | | 3/212 | | 25/18888 | | | 0.12 | | | 10.691320754717 | | | | 5.16600803901029 | | 0.00267227144651391 | | | 0.0313397636471543 | | | | 0.0248158855407606 | | RARRES2/CX3CL1/CCL5 | 3 |
| BP | GO:1901342 | | | regulation of vasculature development | | | | 11/212 | | 362/18888 | | | 0.0303867403314917 | | | 2.70728656311894 | | | | 3.49443821157149 | | 0.00269236855944999 | | | 0.0313397636471543 | | | | 0.0248158855407606 | | SERPINF1/C3/EMILIN1/DCN/SFRP2/CXCL12/SPARC/SFRP1/THBS2/CHI3L1/CXCL13 | 11 |
| BP | GO:0007492 | | | endoderm development | | | | 5/212 | | 85/18888 | | | 0.0588235294117647 | | | 5.24084350721421 | | | | 4.17499177793711 | | 0.00269362692034585 | | | 0.0313397636471543 | | | | 0.0248158855407606 | | MMP2/COL6A1/LAMB3/COL5A1/COL5A2 | 5 |
| BP | GO:0009636 | | | response to toxic substance | | | | 9/212 | | 260/18888 | | | 0.0346153846153846 | | | 3.08403483309144 | | | | 3.60508925061218 | | 0.00275650131600525 | | | 0.0319324568035933 | | | | 0.0252852000415519 | | FBLN5/COL6A1/SOD3/GSTP1/FOS/GPX3/CLDN1/CCL5/S100A9 | 9 |
| BP | GO:0048678 | | | response to axon injury | | | | 5/212 | | 86/18888 | | | 0.0581395348837209 | | | 5.17990346643265 | | | | 4.13924317564716 | | 0.00283427718934232 | | | 0.0326919213736209 | | | | 0.0258865697919514 | | MMP2/DPYSL3/TNC/PTN/APOD | 5 |
| BP | GO:0001952 | | | regulation of cell-matrix adhesion | | | | 6/212 | | 125/18888 | | | 0.048 | | | 4.27652830188679 | | | | 3.91584307014567 | | 0.0028887252813236 | | | 0.0331769478661887 | | | | 0.0262706301843434 | | COL16A1/CX3CL1/CCL21/SFRP1/POSTN/APOD | 6 |
| BP | GO:0045778 | | | positive regulation of ossification | | | | 4/212 | | 53/18888 | | | 0.0754716981132075 | | | 6.72410110359558 | | | | 4.44600458805059 | | 0.00293884672235014 | | | 0.0333235331737668 | | | | 0.0263867013920197 | | TMEM119/CCN1/LTF/PTN | 4 |
| BP | GO:0048260 | | | positive regulation of receptor-mediated endocytosis | | | | 4/212 | | 53/18888 | | | 0.0754716981132075 | | | 6.72410110359558 | | | | 4.44600458805059 | | 0.00293884672235014 | | | 0.0333235331737668 | | | | 0.0263867013920197 | | C3/SFRP4/CCL21/CCL19 | 4 |
| BP | GO:1902692 | | | regulation of neuroblast proliferation | | | | 4/212 | | 53/18888 | | | 0.0754716981132075 | | | 6.72410110359558 | | | | 4.44600458805059 | | 0.00293884672235014 | | | 0.0333235331737668 | | | | 0.0263867013920197 | | CX3CL1/VCAN/ID4/PTN | 4 |
| BP | GO:0035988 | | | chondrocyte proliferation | | | | 3/212 | | 26/18888 | | | 0.115384615384615 | | | 10.2801161103048 | | | | 5.04491323884344 | | 0.00299604614953269 | | | 0.0335456882684078 | | | | 0.0265626113147294 | | CCN2/LTF/COMP | 3 |
| BP | GO:1901623 | | | regulation of lymphocyte chemotaxis | | | | 3/212 | | 26/18888 | | | 0.115384615384615 | | | 10.2801161103048 | | | | 5.04491323884344 | | 0.00299604614953269 | | | 0.0335456882684078 | | | | 0.0265626113147294 | | CCL21/CCL5/CXCL13 | 3 |
| BP | GO:1903020 | | | positive regulation of glycoprotein metabolic process | | | | 3/212 | | 26/18888 | | | 0.115384615384615 | | | 10.2801161103048 | | | | 5.04491323884344 | | 0.00299604614953269 | | | 0.0335456882684078 | | | | 0.0265626113147294 | | IL33/CCL21/CCL19 | 3 |
| BP | GO:0031103 | | | axon regeneration | | | | 4/212 | | 54/18888 | | | 0.0740740740740741 | | | 6.59958071278826 | | | | 4.39024335111822 | | 0.00314646016196852 | | | 0.0347930884025941 | | | | 0.0275503449588631 | | MMP2/TNC/PTN/APOD | 4 |
| BP | GO:0070169 | | | positive regulation of biomineral tissue development | | | | 4/212 | | 54/18888 | | | 0.0740740740740741 | | | 6.59958071278826 | | | | 4.39024335111822 | | 0.00314646016196852 | | | 0.0347930884025941 | | | | 0.0275503449588631 | | TMEM119/CCN1/LTF/PTN | 4 |
| BP | GO:0071385 | | | cellular response to glucocorticoid stimulus | | | | 4/212 | | 54/18888 | | | 0.0740740740740741 | | | 6.59958071278826 | | | | 4.39024335111822 | | 0.00314646016196852 | | | 0.0347930884025941 | | | | 0.0275503449588631 | | SERPINF1/ANXA1/ZFP36L2/ZFP36 | 4 |
| BP | GO:0050673 | | | epithelial cell proliferation | | | | 13/212 | | 482/18888 | | | 0.0269709543568465 | | | 2.40295936741564 | | | | 3.32427077395432 | | 0.00322099917083557 | | | 0.0354707563010535 | | | | 0.028086945336331 | | SERPINF1/CAV1/COL8A2/SFRP2/CXCL12/SPARC/ZFP36/NGFR/SFRP1/PTN/CLDN1/CCL5/ESR1 | 13 |
| BP | GO:0070663 | | | regulation of leukocyte proliferation | | | | 9/212 | | 267/18888 | | | 0.0337078651685393 | | | 3.00317998728005 | | | | 3.51221902752326 | | 0.00328711147854372 | | | 0.0357140700964005 | | | | 0.0282796094343696 | | ANXA1/HLA-DPB1/CD74/GSTP1/IL33/CCL19/PLA2G2A/CCL5/VTCN1 | 9 |
| BP | GO:1903131 | | | mononuclear cell differentiation | | | | 13/212 | | 484/18888 | | | 0.0268595041322314 | | | 2.39302978325277 | | | | 3.30776355896847 | | 0.00333586954284917 | | | 0.0357140700964005 | | | | 0.0282796094343696 | | ANXA1/ZFP36L2/EGR1/CTSK/CD74/HLA-DRA/ITM2A/IL7R/FOS/CCL19/SFRP1/CD3D/CD79A | 13 |
| BP | GO:0010818 | | | T cell chemotaxis | | | | 3/212 | | 27/18888 | | | 0.111111111111111 | | | 9.89937106918239 | | | | 4.93022032868203 | | 0.00334291553401082 | | | 0.0357140700964005 | | | | 0.0282796094343696 | | CCL21/CCL5/CXCL13 | 3 |
| BP | GO:0018149 | | | peptide cross-linking | | | | 3/212 | | 27/18888 | | | 0.111111111111111 | | | 9.89937106918239 | | | | 4.93022032868203 | | 0.00334291553401082 | | | 0.0357140700964005 | | | | 0.0282796094343696 | | ANXA1/COL3A1/F13A1 | 3 |
| BP | GO:0036296 | | | response to increased oxygen levels | | | | 3/212 | | 27/18888 | | | 0.111111111111111 | | | 9.89937106918239 | | | | 4.93022032868203 | | 0.00334291553401082 | | | 0.0357140700964005 | | | | 0.0282796094343696 | | MMP2/CAV1/COL1A1 | 3 |
| BP | GO:0071624 | | | positive regulation of granulocyte chemotaxis | | | | 3/212 | | 27/18888 | | | 0.111111111111111 | | | 9.89937106918239 | | | | 4.93022032868203 | | 0.00334291553401082 | | | 0.0357140700964005 | | | | 0.0282796094343696 | | CD74/CCL21/CCL19 | 3 |
| BP | GO:2000050 | | | regulation of non-canonical Wnt signaling pathway | | | | 3/212 | | 27/18888 | | | 0.111111111111111 | | | 9.89937106918239 | | | | 4.93022032868203 | | 0.00334291553401082 | | | 0.0357140700964005 | | | | 0.0282796094343696 | | SFRP2/SFRP4/SFRP1 | 3 |
| BP | GO:0010559 | | | regulation of glycoprotein biosynthetic process | | | | 4/212 | | 55/18888 | | | 0.0727272727272727 | | | 6.47958833619211 | | | | 4.33587758355022 | | 0.00336385843003808 | | | 0.0357140700964005 | | | | 0.0282796094343696 | | ITM2A/IL33/CCL21/CCL19 | 4 |
| BP | GO:0032612 | | | interleukin-1 production | | | | 6/212 | | 129/18888 | | | 0.0465116279069767 | | | 4.14392277314612 | | | | 3.81741495891044 | | 0.00337655445978675 | | | 0.0357140700964005 | | | | 0.0282796094343696 | | ANXA1/EGR1/CX3CL1/SAA1/GSTP1/CCL19 | 6 |
| BP | GO:0032652 | | | regulation of interleukin-1 production | | | | 6/212 | | 129/18888 | | | 0.0465116279069767 | | | 4.14392277314612 | | | | 3.81741495891044 | | 0.00337655445978675 | | | 0.0357140700964005 | | | | 0.0282796094343696 | | ANXA1/EGR1/CX3CL1/SAA1/GSTP1/CCL19 | 6 |
| BP | GO:0060541 | | | respiratory system development | | | | 8/212 | | 220/18888 | | | 0.0363636363636364 | | | 3.23979416809605 | | | | 3.56022988843413 | | 0.00348075934324616 | | | 0.0366713070965619 | | | | 0.0290375820884978 | | CRISPLD2/CCN2/LOX/COL6A1/ADAMTS2/COL3A1/TNC/CHI3L1 | 8 |
| BP | GO:0048660 | | | regulation of smooth muscle cell proliferation | | | | 7/212 | | 174/18888 | | | 0.0402298850574713 | | | 3.58425504229018 | | | | 3.64866652218203 | | 0.00355738685143152 | | | 0.0372811152808962 | | | | 0.0295204488476297 | | MMP2/CNN1/CX3CL1/ELN/OGN/CCL5/APOD | 7 |
| BP | GO:0009314 | | | response to radiation | | | | 12/212 | | 431/18888 | | | 0.0278422273781903 | | | 2.48058486188329 | | | | 3.3128278738504 | | 0.00356650430190936 | | | 0.0372811152808962 | | | | 0.0295204488476297 | | MMP2/COL6A2/MFAP4/COL6A1/EGR1/SFRP2/COL3A1/MMP3/COL6A3/FOS/SFRP1/CRYAB | 12 |
| BP | GO:0001954 | | | positive regulation of cell-matrix adhesion | | | | 4/212 | | 56/18888 | | | 0.0714285714285714 | | | 6.36388140161725 | | | | 4.28284587145423 | | 0.00359125934134123 | | | 0.0373938132195686 | | | | 0.0296096868896985 | | COL16A1/CX3CL1/CCL21/SFRP1 | 4 |
| BP | GO:0001704 | | | formation of primary germ layer | | | | 6/212 | | 131/18888 | | | 0.0458015267175573 | | | 4.08065677660954 | | | | 3.76968239902646 | | 0.00364228785237783 | | | 0.0377781484223375 | | | | 0.0299140165109611 | | MMP2/COL6A1/LAMB3/SFRP2/COL5A1/COL5A2 | 6 |
| BP | GO:0048525 | | | negative regulation of viral process | | | | 5/212 | | 93/18888 | | | 0.0537634408602151 | | | 4.7900182592818 | | | | 3.90363406790429 | | 0.00397162570357231 | | | 0.0408832143553143 | | | | 0.0323727128067216 | | ZFP36/LRRC15/LTF/CCL5/SLPI | 5 |
| BP | GO:0036293 | | | response to decreased oxygen levels | | | | 10/212 | | 327/18888 | | | 0.0305810397553517 | | | 2.7245975419768 | | | | 3.35172548623361 | | 0.00397221066232501 | | | 0.0408832143553143 | | | | 0.0323727128067216 | | MMP2/CAV1/COL6A1/EGR1/SOD3/CXCL12/FOS/SFRP1/CRYAB/POSTN | 10 |
| BP | GO:0048659 | | | smooth muscle cell proliferation | | | | 7/212 | | 178/18888 | | | 0.0393258426966292 | | | 3.50370998516006 | | | | 3.57572916489764 | | 0.00402922402718934 | | | 0.0413111245086539 | | | | 0.0327115465486273 | | MMP2/CNN1/CX3CL1/ELN/OGN/CCL5/APOD | 7 |
| BP | GO:1902105 | | | regulation of leukocyte differentiation | | | | 10/212 | | 328/18888 | | | 0.0304878048780488 | | | 2.7162908421537 | | | | 3.340767939 | | 0.00405800466779809 | | | 0.0414474064543042 | | | | 0.032819459205615 | | ANXA1/ZFP36L2/CD74/FBN1/HLA-DRA/IL7R/FOS/CCL19/SFRP1/LTF | 10 |
| BP | GO:0070228 | | | regulation of lymphocyte apoptotic process | | | | 4/212 | | 58/18888 | | | 0.0689655172413793 | | | 6.14443721535459 | | | | 4.18055745095983 | | 0.00407692761251603 | | | 0.041482350916703 | | | | 0.0328471294136273 | | CD74/IL7R/CCL5/PIP | 4 |
| BP | GO:1903034 | | | regulation of response to wounding | | | | 7/212 | | 179/18888 | | | 0.0391061452513966 | | | 3.48413618636028 | | | | 3.55782118854943 | | 0.00415426810737402 | | | 0.0421091721792912 | | | | 0.033343467703923 | | SERPING1/ANXA1/EMILIN1/CAV1/IL33/PTN/CLDN1 | 7 |
| BP | GO:0001819 | | | positive regulation of cytokine production | | | | 13/212 | | 500/18888 | | | 0.026 | | | 2.31645283018868 | | | | 3.1785609613412 | | 0.00438023039083273 | | | 0.0442228731759043 | | | | 0.0350171676906204 | | C3/ANXA1/EGR1/HLA-DPB1/CD74/CX3CL1/LUM/SAA1/IL33/CCL19/POSTN/CHI3L1/VTCN1 | 13 |
| BP | GO:0046631 | | | alpha-beta T cell activation | | | | 7/212 | | 181/18888 | | | 0.0386740331491713 | | | 3.44563744396956 | | | | 3.52238462956416 | | 0.00441315648439195 | | | 0.0442228731759043 | | | | 0.0350171676906204 | | ANXA1/HLA-DRA/TRAC/CCL19/TRBC2/CD3D/TRBC1 | 7 |
| BP | GO:0071478 | | | cellular response to radiation | | | | 7/212 | | 181/18888 | | | 0.0386740331491713 | | | 3.44563744396956 | | | | 3.52238462956416 | | 0.00441315648439195 | | | 0.0442228731759043 | | | | 0.0350171676906204 | | MMP2/MFAP4/EGR1/SFRP2/MMP3/SFRP1/CRYAB | 7 |
| BP | GO:0050920 | | | regulation of chemotaxis | | | | 8/212 | | 229/18888 | | | 0.0349344978165939 | | | 3.11246601301804 | | | | 3.42665812387979 | | 0.00442889761253451 | | | 0.0442228731759043 | | | | 0.0350171676906204 | | RARRES2/CXCL12/CD74/CCL21/CCL19/PTN/CCL5/CXCL13 | 8 |
| BP | GO:1902230 | | | negative regulation of intrinsic apoptotic signaling pathway in response to DNA damage | | | | 3/212 | | 30/18888 | | | 0.1 | | | 8.90943396226415 | | | | 4.61918869907155 | | 0.00452700062723271 | | | 0.0450344002917276 | | | | 0.0356597623268223 | | SFRP2/CXCL12/CD74 | 3 |
| BP | GO:0097191 | | | extrinsic apoptotic signaling pathway | | | | 8/212 | | 230/18888 | | | 0.0347826086956522 | | | 3.09893355209188 | | | | 3.41222414372976 | | 0.00454540887968031 | | | 0.0450500524519427 | | | | 0.0356721562370233 | | SRPX/CAV1/SFRP2/CX3CL1/GSTP1/IL33/SFRP1/G0S2 | 8 |
| BP | GO:0060350 | | | endochondral bone morphogenesis | | | | 4/212 | | 60/18888 | | | 0.0666666666666667 | | | 5.93962264150943 | | | | 4.08295685751583 | | 0.00460515509529683 | | | 0.045473782417027 | | | | 0.0360076799643912 | | TMEM119/COL1A1/COL3A1/COMP | 4 |
| BP | GO:0007565 | | | female pregnancy | | | | 7/212 | | 183/18888 | | | 0.0382513661202186 | | | 3.40798020414476 | | | | 3.4874427655841 | | 0.00468406471014324 | | | 0.0460829307512621 | | | | 0.0364900242318821 | | MMP2/FBLN1/COL16A1/FOS/PTN/FOSB/ESR1 | 7 |
| BP | GO:1903018 | | | regulation of glycoprotein metabolic process | | | | 4/212 | | 61/18888 | | | 0.0655737704918033 | | | 5.84225177853387 | | | | 4.0357960556057 | | 0.00488574442319437 | | | 0.047891033247136 | | | | 0.0379217409828042 | | ITM2A/IL33/CCL21/CCL19 | 4 |
| BP | GO:0002825 | | | regulation of T-helper 1 type immune response | | | | 3/212 | | 31/18888 | | | 0.0967741935483871 | | | 8.62203286670724 | | | | 4.52504446916958 | | 0.00497103141539198 | | | 0.0481973915492353 | | | | 0.0381643258550116 | | ANXA1/IL33/CCL19 | 3 |
| BP | GO:0036336 | | | dendritic cell migration | | | | 3/212 | | 31/18888 | | | 0.0967741935483871 | | | 8.62203286670724 | | | | 4.52504446916958 | | 0.00497103141539198 | | | 0.0481973915492353 | | | | 0.0381643258550116 | | CCL21/CCL19/CCL5 | 3 |
| BP | GO:0060325 | | | face morphogenesis | | | | 3/212 | | 31/18888 | | | 0.0967741935483871 | | | 8.62203286670724 | | | | 4.52504446916958 | | 0.00497103141539198 | | | 0.0481973915492353 | | | | 0.0381643258550116 | | CRISPLD2/MMP2/COL1A1 | 3 |
| CC | GO:0019814 | | | immunoglobulin complex | | | | 58/216 | | 165/19894 | | | 0.351515151515152 | | | 32.3751964085297 | | | | 42.3996652354196 | | 2.24608845674463e-73 | | | 4.64940310546139e-71 | | | | 3.71195671272534e-71 | | IGHA2/IGHA1/JCHAIN/IGHV3-7/IGHV3-74/IGHV3-72/IGLV7-43/IGKV1-17/IGHV3-15/IGHM/IGLV2-11/IGLV2-8/IGLV2-18/CD79A/IGKV2-24/IGKV2-30/IGLC2/IGKV1-9/IGLV1-40/IGHV5-51/IGKJ1/IGKV1-27/IGKV1-6/IGHV3-49/IGLV7-46/IGKC/IGHV3-23/IGLV6-57/IGLV4-69/IGKV1-5/IGKV4-1/IGKV3-15/IGLV2-14/IGKV1-16/IGLV1-44/IGHV2-5/IGLV2-23/IGLV1-47/IGHV4-59/IGHV3-30/IGKV1-12/IGHV6-1/IGHV4-39/IGHV3-11/IGLV3-21/IGHV4-34/IGHV3-48/IGLC3/IGHV1-24/IGKV3-20/IGHV4-4/IGHV3-21/IGLV8-61/IGHV1-18/IGHV1-3/IGLV3-25/IGLV3-19/IGHG1 | 58 |
| CC | GO:0062023 | | | collagen-containing extracellular matrix | | | | 64/216 | | 428/19894 | | | 0.149532710280374 | | | 13.7722395292489 | | | | 27.9857608392716 | | 5.68554751461713e-55 | | | 5.88454167762873e-53 | | | | 4.698057683131e-53 | | SERPING1/FBLN5/FBLN2/SERPINF1/MMP2/SRPX/CCDC80/FBLN1/CCN1/CCN2/ANXA1/DPT/ANGPTL2/COL16A1/COL6A2/MFAP4/LOX/EMILIN1/BGN/DCN/RARRES2/COL6A1/PRELP/LTBP2/PODN/ADAMTS2/LAMB3/COL8A2/SOD3/HTRA1/COL14A1/SFRP2/COL1A1/CXCL12/COL5A1/SPARC/ACTA2/MXRA5/AEBP1/COL1A2/COL3A1/SPON1/LUM/FBN1/ELN/TNC/MFGE8/MFAP5/COL5A2/FMOD/COL6A3/LRRC15/CILP/VCAN/F13A1/OGN/SFRP1/ASPN/COL17A1/THBS2/POSTN/COMP/SLPI/S100A9 | 64 |
| CC | GO:0072562 | | | blood microparticle | | | | 24/216 | | 144/19894 | | | 0.166666666666667 | | | 15.3503086419753 | | | | 18.1069572685897 | | 8.45131679121379e-22 | | | 5.83140858593751e-20 | | | | 4.65563767094935e-20 | | SERPING1/C1R/C1S/C3/F13A1/IGHA2/IGHA1/JCHAIN/IGHV3-7/IGKV1-17/IGHM/IGKV2-30/IGLC2/IGKC/IGHV3-23/IGKV1-5/IGKV4-1/IGKV3-15/IGLV1-47/IGLV3-21/IGLC3/IGKV3-20/IGLV3-25/IGHG1 | 24 |
| CC | GO:0005581 | | | collagen trimer | | | | 15/216 | | 86/19894 | | | 0.174418604651163 | | | 16.0642764857881 | | | | 14.6677577538902 | | 2.32608414282657e-14 | | | 1.20374854391275e-12 | | | | 9.61040027430978e-13 | | COL16A1/COL6A2/LOX/EMILIN1/COL6A1/COL8A2/COL14A1/COL1A1/COL5A1/COL1A2/COL3A1/LUM/COL5A2/COL6A3/COL17A1 | 15 |
| CC | GO:0005788 | | | endoplasmic reticulum lumen | | | | 22/216 | | 313/19894 | | | 0.0702875399361022 | | | 6.47361259022601 | | | | 10.2262246627764 | | 4.39411957259625e-12 | | | 1.81916550305485e-10 | | | | 1.45237215346865e-10 | | SERPING1/FSTL1/C3/CCN1/COL16A1/COL6A2/COL6A1/COL8A2/COL14A1/COL1A1/COL5A1/COL1A2/COL3A1/SPON1/FBN1/TNC/MFGE8/COL5A2/COL6A3/VCAN/CHRDL1/COL17A1 | 22 |
| CC | GO:0005604 | | | basement membrane | | | | 13/216 | | 92/19894 | | | 0.141304347826087 | | | 13.0143921095008 | | | | 12.1011801443883 | | 2.12967765381758e-11 | | | 7.34738790567066e-10 | | | | 5.86595423946246e-10 | | SERPINF1/CCDC80/FBLN1/COL6A1/LAMB3/COL8A2/COL5A1/SPARC/ACTA2/FBN1/TNC/COL17A1/THBS2 | 13 |
| CC | GO:0071745 | | | IgA immunoglobulin complex | | | | 6/216 | | 10/19894 | | | 0.6 | | | 55.2611111111111 | | | | 17.9813934599656 | | 3.095474720535e-10 | | | 8.00954083938432e-09 | | | | 6.39459909373679e-09 | | IGHA2/IGHA1/JCHAIN/IGKC/IGLC3/IGKV3-20 | 6 |
| CC | GO:0071753 | | | IgM immunoglobulin complex | | | | 6/216 | | 10/19894 | | | 0.6 | | | 55.2611111111111 | | | | 17.9813934599656 | | 3.095474720535e-10 | | | 8.00954083938432e-09 | | | | 6.39459909373679e-09 | | JCHAIN/IGHM/CD79A/IGKC/IGLC3/IGKV3-20 | 6 |
| CC | GO:0005583 | | | fibrillar collagen trimer | | | | 6/216 | | 12/19894 | | | 0.5 | | | 46.0509259259259 | | | | 16.3550112980894 | | 1.33748238264296e-09 | | | 2.51689866551902e-08 | | | | 2.0094232925832e-08 | | COL1A1/COL5A1/COL1A2/COL3A1/LUM/COL5A2 | 6 |
| CC | GO:0042571 | | | immunoglobulin complex, circulating | | | | 6/216 | | 12/19894 | | | 0.5 | | | 46.0509259259259 | | | | 16.3550112980894 | | 1.33748238264296e-09 | | | 2.51689866551902e-08 | | | | 2.0094232925832e-08 | | IGHA2/IGHA1/JCHAIN/IGHM/IGKV3-20/IGHG1 | 6 |
| CC | GO:0098643 | | | banded collagen fibril | | | | 6/216 | | 12/19894 | | | 0.5 | | | 46.0509259259259 | | | | 16.3550112980894 | | 1.33748238264296e-09 | | | 2.51689866551902e-08 | | | | 2.0094232925832e-08 | | COL1A1/COL5A1/COL1A2/COL3A1/LUM/COL5A2 | 6 |
| CC | GO:0098644 | | | complex of collagen trimers | | | | 6/216 | | 21/19894 | | | 0.285714285714286 | | | 26.3148148148148 | | | | 12.1601581888509 | | 7.23841962150541e-08 | | | 1.24862738470968e-06 | | | | 9.96870070681008e-07 | | COL1A1/COL5A1/COL1A2/COL3A1/LUM/COL5A2 | 6 |
| CC | GO:0043202 | | | lysosomal lumen | | | | 10/216 | | 98/19894 | | | 0.102040816326531 | | | 9.39814814814815 | | | | 8.73161112685925 | | 1.12640771776688e-07 | | | 1.79358767367496e-06 | | | | 1.43195151165507e-06 | | BGN/DCN/PRELP/CTSK/CD74/LUM/FMOD/OMD/VCAN/OGN | 10 |
| CC | GO:0071735 | | | IgG immunoglobulin complex | | | | 5/216 | | 13/19894 | | | 0.384615384615385 | | | 35.4237891737892 | | | | 13.0076180363068 | | 1.72734227279499e-07 | | | 2.55399893191831e-06 | | | | 2.03904313405123e-06 | | IGHA1/IGLC2/IGKC/IGLC3/IGHG1 | 5 |
| CC | GO:0005796 | | | Golgi lumen | | | | 10/216 | | 106/19894 | | | 0.0943396226415094 | | | 8.68885394828791 | | | | 8.31572525232654 | | 2.37569618911716e-07 | | | 3.27846074098168e-06 | | | | 2.61743369607996e-06 | | BGN/DCN/PRELP/SOD3/LUM/FMOD/OMD/VCAN/OGN/MUCL1 | 10 |
| CC | GO:0005775 | | | vacuolar lumen | | | | 11/216 | | 176/19894 | | | 0.0625 | | | 5.75636574074074 | | | | 6.64028445303853 | | 3.66261370562996e-06 | | | 4.73850648165876e-05 | | | | 3.78309441963095e-05 | | C3/BGN/DCN/PRELP/CTSK/CD74/LUM/FMOD/OMD/VCAN/OGN | 11 |
| CC | GO:0042105 | | | alpha-beta T cell receptor complex | | | | 4/216 | | 12/19894 | | | 0.333333333333333 | | | 30.7006172839506 | | | | 10.7823295804826 | | 6.24905104306365e-06 | | | 7.60913862302456e-05 | | | | 6.07492887777705e-05 | | TRAC/TRBC2/CD3D/TRBC1 | 4 |
| CC | GO:0042613 | | | MHC class II protein complex | | | | 4/216 | | 17/19894 | | | 0.235294117647059 | | | 21.6710239651416 | | | | 8.93300487686355 | | 2.87905695881075e-05 | | | 0.000331091550263236 | | | | 0.000264334469317712 | | HLA-DPB1/CD74/HLA-DRA/HLA-DRB5 | 4 |
| CC | GO:0042611 | | | MHC protein complex | | | | 4/216 | | 25/19894 | | | 0.16 | | | 14.7362962962963 | | | | 7.20009436475668 | | 0.000142945420967842 | | | 0.0015573527442286 | | | | 0.00124334798293359 | | HLA-DPB1/CD74/HLA-DRA/HLA-DRB5 | 4 |
| CC | GO:0042383 | | | sarcolemma | | | | 8/216 | | 141/19894 | | | 0.0567375886524823 | | | 5.22563698450223 | | | | 5.27559864739446 | | 0.000156059827540895 | | | 0.00161521921504826 | | | | 0.00128954699599582 | | ANXA1/COL6A2/BGN/CAV1/COL6A1/COL6A3/KCNJ3/STAC2 | 8 |
| CC | GO:0030669 | | | clathrin-coated endocytic vesicle membrane | | | | 6/216 | | 75/19894 | | | 0.08 | | | 7.36814814814815 | | | | 5.78881219224926 | | 0.000164914880833363 | | | 0.00162558953964315 | | | | 0.00129782638049313 | | HLA-DPB1/CD74/HLA-DRA/IL7R/HLA-DRB5/CD3D | 6 |
| CC | GO:0005614 | | | interstitial matrix | | | | 3/216 | | 11/19894 | | | 0.272727272727273 | | | 25.1186868686869 | | | | 8.3829250692448 | | 0.000195309036851233 | | | 0.00183768048310024 | | | | 0.00146715400888247 | | CCDC80/COL14A1/TNC | 3 |
| CC | GO:0034774 | | | secretory granule lumen | | | | 12/216 | | 322/19894 | | | 0.0372670807453416 | | | 3.43236714975845 | | | | 4.61026617802064 | | 0.000219571820982348 | | | 0.00197614638884113 | | | | 0.00157770141392351 | | SERPING1/CRISPLD2/C3/RARRES2/ISLR/SPARC/GSTP1/F13A1/LTF/SLPI/CHI3L1/S100A9 | 12 |
| CC | GO:0060205 | | | cytoplasmic vesicle lumen | | | | 12/216 | | 325/19894 | | | 0.0369230769230769 | | | 3.40068376068376 | | | | 4.57171193288914 | | 0.000239025843979758 | | | 0.00203545915722885 | | | | 0.001625055111543 | | SERPING1/CRISPLD2/C3/RARRES2/ISLR/SPARC/GSTP1/F13A1/LTF/SLPI/CHI3L1/S100A9 | 12 |
| CC | GO:0031983 | | | vesicle lumen | | | | 12/216 | | 326/19894 | | | 0.0368098159509202 | | | 3.39025221540559 | | | | 4.55896069917437 | | 0.000245828400631504 | | | 0.00203545915722885 | | | | 0.001625055111543 | | SERPING1/CRISPLD2/C3/RARRES2/ISLR/SPARC/GSTP1/F13A1/LTF/SLPI/CHI3L1/S100A9 | 12 |
| CC | GO:0098553 | | | lumenal side of endoplasmic reticulum membrane | | | | 4/216 | | 29/19894 | | | 0.137931034482759 | | | 12.7037037037037 | | | | 6.60791688100517 | | 0.000259414618705679 | | | 0.0020653394643106 | | | | 0.00164891073428306 | | HLA-DPB1/CD74/HLA-DRA/HLA-DRB5 | 4 |
| CC | GO:0001527 | | | microfibril | | | | 3/216 | | 13/19894 | | | 0.230769230769231 | | | 21.2542735042735 | | | | 7.65342397422886 | | 0.000333142850868453 | | | 0.00255409518999147 | | | | 0.00203911998387318 | | MFAP4/FBN1/MFAP5 | 3 |
| CC | GO:0045095 | | | keratin filament | | | | 6/216 | | 92/19894 | | | 0.0652173913043478 | | | 6.00664251207729 | | | | 5.04280885832663 | | 0.000501335286284152 | | | 0.00370630015217212 | | | | 0.00295900902054932 | | KRT7/KRT5/KRT14/KRT17/KRT6B/KRT81 | 6 |
| CC | GO:0045334 | | | clathrin-coated endocytic vesicle | | | | 6/216 | | 93/19894 | | | 0.0645161290322581 | | | 5.94205495818399 | | | | 5.00486101742919 | | 0.000531127585826554 | | | 0.00379115207814127 | | | | 0.00302675248547256 | | HLA-DPB1/CD74/HLA-DRA/IL7R/HLA-DRB5/CD3D | 6 |
| CC | GO:0098576 | | | lumenal side of membrane | | | | 4/216 | | 39/19894 | | | 0.102564102564103 | | | 9.44634377967711 | | | | 5.53162397820022 | | 0.000825322350029625 | | | 0.00569472421520441 | | | | 0.00454651259490004 | | HLA-DPB1/CD74/HLA-DRA/HLA-DRB5 | 4 |
| CC | GO:0071682 | | | endocytic vesicle lumen | | | | 3/216 | | 23/19894 | | | 0.130434782608696 | | | 12.0132850241546 | | | | 5.5367826333683 | | 0.00190423887955512 | | | 0.0127154015505777 | | | | 0.0101516300200392 | | SPARC/SAA1/LTF | 3 |
| CC | GO:0031091 | | | platelet alpha granule | | | | 5/216 | | 91/19894 | | | 0.0549450549450549 | | | 5.06054131054131 | | | | 4.06748232497125 | | 0.00314533894648737 | | | 0.0203464113100902 | | | | 0.0162440202170565 | | SERPING1/ISLR/SPARC/F13A1/THBS2 | 5 |
| CC | GO:0030665 | | | clathrin-coated vesicle membrane | | | | 6/216 | | 138/19894 | | | 0.0434782608695652 | | | 4.00442834138486 | | | | 3.71055115533166 | | 0.00400659083617408 | | | 0.0251322516087283 | | | | 0.0200649046660073 | | HLA-DPB1/CD74/HLA-DRA/IL7R/HLA-DRB5/CD3D | 6 |
| CC | GO:0030139 | | | endocytic vesicle | | | | 10/216 | | 350/19894 | | | 0.0285714285714286 | | | 2.63148148148148 | | | | 3.22623488262748 | | 0.00507473691390372 | | | 0.0305425467150275 | | | | 0.0243843368129129 | | CAV1/HLA-DPB1/SPARC/CD74/HLA-DRA/SAA1/IL7R/LTF/HLA-DRB5/CD3D | 10 |
| CC | GO:0012507 | | | ER to Golgi transport vesicle membrane | | | | 4/216 | | 64/19894 | | | 0.0625 | | | 5.75636574074074 | | | | 3.99291817937307 | | 0.00516419871993218 | | | 0.0305425467150275 | | | | 0.0243843368129129 | | HLA-DPB1/CD74/HLA-DRA/HLA-DRB5 | 4 |
| CC | GO:0098966 | | | perisynaptic extracellular matrix | | | | 2/216 | | 11/19894 | | | 0.181818181818182 | | | 16.7457912457912 | | | | 5.4727601418802 | | 0.00605057336367965 | | | 0.0339872582660301 | | | | 0.0271345006242905 | | TNC/VCAN | 2 |
| CC | GO:0031093 | | | platelet alpha granule lumen | | | | 4/216 | | 67/19894 | | | 0.0597014925373134 | | | 5.49861802100608 | | | | 3.86433310502892 | | 0.00607501717798606 | | | 0.0339872582660301 | | | | 0.0271345006242905 | | SERPING1/ISLR/SPARC/F13A1 | 4 |
| CC | GO:0030666 | | | endocytic vesicle membrane | | | | 7/216 | | 204/19894 | | | 0.0343137254901961 | | | 3.16035766158315 | | | | 3.2494093205746 | | 0.00702738605406436 | | | 0.0382632718361085 | | | | 0.0305483533092755 | | CAV1/HLA-DPB1/CD74/HLA-DRA/IL7R/HLA-DRB5/CD3D | 7 |
| CC | GO:0099535 | | | synapse-associated extracellular matrix | | | | 2/216 | | 12/19894 | | | 0.166666666666667 | | | 15.3503086419753 | | | | 5.2096478628758 | | 0.00720902222999146 | | | 0.0382632718361085 | | | | 0.0305483533092755 | | TNC/VCAN | 2 |
| MF | GO:0003823 | | | antigen binding | | | | 51/200 | | 177/18522 | | | 0.288135593220339 | | | 26.6842372881356 | | | | 35.8719696107819 | | 1.72400217225707e-59 | | | 4.84444610404237e-57 | | | | 3.88354173540014e-57 | | HLA-DPB1/HLA-DRA/IL7R/IGHA2/IGHA1/HLA-DRB5/JCHAIN/IGHV3-7/IGHV3-74/IGHV3-72/IGLV7-43/IGKV1-17/IGHV3-15/IGHM/IGLV2-11/IGLV2-8/IGKV2-30/IGLC2/IGLV1-40/IGHV5-51/IGHV3-49/IGKC/IGHV3-23/IGLV6-57/IGKV1-5/IGKV4-1/IGKV3-15/IGLV2-14/IGKV1-16/IGLV1-44/IGHV2-5/IGLV2-23/IGLV1-47/IGHV4-59/IGHV3-30/IGHV6-1/IGHV4-39/IGHV3-11/IGLV3-21/IGHV4-34/IGHV3-48/IGLC3/IGHV1-24/IGKV3-20/IGHV4-4/IGHV3-21/IGHV1-18/IGHV1-3/IGLV3-25/IGLV3-19/IGHG1 | 51 |
| MF | GO:0005201 | | | extracellular matrix structural constituent | | | | 45/200 | | 166/18522 | | | 0.271084337349398 | | | 25.1051204819277 | | | | 32.5938078042826 | | 5.34601055782056e-51 | | | 7.51114483373789e-49 | | | | 6.02129610196632e-49 | | FBLN5/FBLN2/SRPX/FBLN1/CCN1/DPT/COL16A1/COL6A2/MFAP4/EMILIN1/BGN/DCN/COL6A1/PRELP/LTBP2/PODN/LAMB3/COL8A2/COL14A1/COL1A1/COL5A1/SPARC/MXRA5/AEBP1/COL1A2/COL3A1/SPON1/LUM/FBN1/ELN/TNC/MFGE8/MFAP5/COL5A2/FMOD/COL6A3/CILP/VCAN/OGN/ASPN/COL17A1/THBS2/POSTN/COMP/CHI3L1 | 45 |
| MF | GO:0005539 | | | glycosaminoglycan binding | | | | 25/200 | | 239/18522 | | | 0.104602510460251 | | | 9.68723849372385 | | | | 14.1227001917833 | | 1.02924490311896e-17 | | | 9.64059392588089e-16 | | | | 7.72836523745462e-16 | | CRISPLD2/FSTL1/CCDC80/DPYSL3/CCN1/CCN2/BGN/DCN/PRELP/LTBP2/SOD3/COL5A1/FBN1/SAA1/VCAN/SFRP1/LTF/COL17A1/PTN/THBS2/JCHAIN/POSTN/COMP/IGHM/CXCL13 | 25 |
| MF | GO:0030020 | | | extracellular matrix structural constituent conferring tensile strength | | | | 12/200 | | 45/18522 | | | 0.266666666666667 | | | 24.696 | | | | 16.6274765095896 | | 3.7958814662872e-14 | | | 2.61015617166197e-12 | | | | 2.09242712393955e-12 | | COL16A1/COL6A2/COL6A1/COL8A2/COL14A1/COL1A1/COL5A1/COL1A2/COL3A1/COL5A2/COL6A3/COL17A1 | 12 |
| MF | GO:0008201 | | | heparin binding | | | | 19/200 | | 174/18522 | | | 0.109195402298851 | | | 10.1125862068966 | | | | 12.6177722039827 | | 4.64440599939852e-14 | | | 2.61015617166197e-12 | | | | 2.09242712393955e-12 | | CRISPLD2/FSTL1/CCDC80/CCN1/CCN2/PRELP/LTBP2/SOD3/COL5A1/FBN1/SAA1/SFRP1/LTF/COL17A1/PTN/THBS2/POSTN/COMP/CXCL13 | 19 |
| MF | GO:0030021 | | | extracellular matrix structural constituent conferring compression resistance | | | | 9/200 | | 22/18522 | | | 0.409090909090909 | | | 37.8859090909091 | | | | 18.0861755559932 | | 7.33986666972651e-13 | | | 3.43750422365525e-11 | | | | 2.75566924091487e-11 | | BGN/DCN/PRELP/PODN/LUM/FMOD/VCAN/OGN/ASPN | 9 |
| MF | GO:1901681 | | | sulfur compound binding | | | | 21/200 | | 267/18522 | | | 0.0786516853932584 | | | 7.28393258426966 | | | | 10.8058019506503 | | 1.36565389877995e-12 | | | 5.48212493653092e-11 | | | | 4.39473585472042e-11 | | CRISPLD2/FSTL1/CCDC80/DPYSL3/CCN1/CCN2/PRELP/LTBP2/SOD3/COL5A1/FBN1/SAA1/GSTP1/SFRP1/LTF/COL17A1/PTN/THBS2/POSTN/COMP/CXCL13 | 21 |
| MF | GO:0005518 | | | collagen binding | | | | 12/200 | | 68/18522 | | | 0.176470588235294 | | | 16.3429411764706 | | | | 13.2427625660961 | | 7.73959400687653e-12 | | | 2.71853239491538e-10 | | | | 2.17930673351523e-10 | | COL6A2/MRC2/LOX/COL6A1/PODN/CTSK/COL14A1/SPARC/AEBP1/LUM/LRRC15/COMP | 12 |
| MF | GO:0005178 | | | integrin binding | | | | 16/200 | | 157/18522 | | | 0.101910828025478 | | | 9.43796178343949 | | | | 11.0930938757831 | | 1.45425639091463e-11 | | | 4.54051162052233e-10 | | | | 3.6398931889559e-10 | | FBLN5/FBLN1/CCN1/CCN2/COL16A1/EMILIN1/SFRP2/CXCL12/COL5A1/COL3A1/CX3CL1/FBN1/TNC/MFGE8/PTN/COMP | 16 |
| MF | GO:0019838 | | | growth factor binding | | | | 12/200 | | 135/18522 | | | 0.0888888888888889 | | | 8.232 | | | | 8.81111030159409 | | 2.61617565595948e-08 | | | 7.35145359324615e-07 | | | | 5.89327989868768e-07 | | CCN1/CCN2/COL6A1/LTBP2/HTRA1/COL1A1/COL5A1/COL1A2/COL3A1/NGFR/CHRDL1/CXCL13 | 12 |
| MF | GO:0030023 | | | extracellular matrix constituent conferring elasticity | | | | 5/200 | | 10/18522 | | | 0.5 | | | 46.305 | | | | 14.9720144720916 | | 3.36770329943092e-08 | | | 8.60295115581899e-07 | | | | 6.89654072802122e-07 | | FBLN5/FBLN2/EMILIN1/FBN1/ELN | 5 |
| MF | GO:0008009 | | | chemokine activity | | | | 8/200 | | 49/18522 | | | 0.163265306122449 | | | 15.12 | | | | 10.3401013579192 | | 4.95797462085881e-08 | | | 1.16099239038444e-06 | | | | 9.3070751654718e-07 | | CXCL12/CX3CL1/CCL21/CCL19/CCL5/CXCL9/CXCL13/CXCL14 | 8 |
| MF | GO:0048407 | | | platelet-derived growth factor binding | | | | 5/200 | | 11/18522 | | | 0.454545454545455 | | | 42.0954545454545 | | | | 14.2441311890785 | | 6.11999662907813e-08 | | | 1.32286080982381e-06 | | | | 1.06046905151637e-06 | | COL6A1/COL1A1/COL5A1/COL1A2/COL3A1 | 5 |
| MF | GO:0097493 | | | structural molecule activity conferring elasticity | | | | 5/200 | | 12/18522 | | | 0.416666666666667 | | | 38.5875 | | | | 13.6079162910422 | | 1.03994679155256e-07 | | | 2.08732177447335e-06 | | | | 1.67329784505449e-06 | | FBLN5/FBLN2/EMILIN1/FBN1/ELN | 5 |
| MF | GO:0050840 | | | extracellular matrix binding | | | | 8/200 | | 64/18522 | | | 0.125 | | | 11.57625 | | | | 8.855032276 | | 4.23699201412388e-07 | | | 7.9372983731254e-06 | | | | 6.36292134050884e-06 | | FBLN2/CCN1/BGN/DCN/LTBP2/SPARC/ELN/LRRC15 | 8 |
| MF | GO:0034987 | | | immunoglobulin receptor binding | | | | 5/200 | | 16/18522 | | | 0.3125 | | | 28.940625 | | | | 11.681553525257 | | 5.5371153986541e-07 | | | 9.72455891888627e-06 | | | | 7.79567562705249e-06 | | IGHA2/IGHA1/JCHAIN/IGHM/IGHG1 | 5 |
| MF | GO:0001968 | | | fibronectin binding | | | | 6/200 | | 30/18522 | | | 0.2 | | | 18.522 | | | | 10.0349019933574 | | 7.03524905324034e-07 | | | 1.16288528468267e-05 | | | | 9.32224951946397e-06 | | MMP2/CCDC80/FBLN1/CTSK/SFRP2/LRRC15 | 6 |
| MF | GO:0042379 | | | chemokine receptor binding | | | | 8/200 | | 74/18522 | | | 0.108108108108108 | | | 10.0118918918919 | | | | 8.1155500862876 | | 1.31566807897611e-06 | | | 2.05390405662382e-05 | | | | 1.64650859006368e-05 | | CXCL12/CX3CL1/CCL21/CCL19/CCL5/CXCL9/CXCL13/CXCL14 | 8 |
| MF | GO:0061134 | | | peptidase regulator activity | | | | 12/200 | | 232/18522 | | | 0.0517241379310345 | | | 4.7901724137931 | | | | 6.06955540755565 | | 8.64182428663264e-06 | | | 0.000127808032870725 | | | | 0.000102457085725174 | | SERPING1/SERPINF1/C3/FBLN1/CAV1/CTSK/SFRP2/COL6A3/LTF/PI16/SLPI/RARRES1 | 12 |
| MF | GO:0045236 | | | CXCR chemokine receptor binding | | | | 4/200 | | 18/18522 | | | 0.222222222222222 | | | 20.58 | | | | 8.68314202216887 | | 3.58532679798714e-05 | | | 0.000503738415117194 | | | | 0.000403821018299604 | | CXCL12/CX3CL1/CXCL9/CXCL13 | 4 |
| MF | GO:0004252 | | | serine-type endopeptidase activity | | | | 9/200 | | 174/18522 | | | 0.0517241379310345 | | | 4.7901724137931 | | | | 5.24807459103839 | | 0.000117480025229819 | | | 0.00157199462331329 | | | | 0.00126018673680107 | | C1R/C1S/MMP2/CTSK/HTRA1/MMP3/HTRA3/LTF/MMP7 | 9 |
| MF | GO:0023026 | | | MHC class II protein complex binding | | | | 4/200 | | 27/18522 | | | 0.148148148148148 | | | 13.72 | | | | 6.91039066235875 | | 0.000190562700722297 | | | 0.00243400540468025 | | | | 0.00195121617007519 | | HLA-DPB1/CD74/HLA-DRA/HLA-DRB5 | 4 |
| MF | GO:0048020 | | | CCR chemokine receptor binding | | | | 5/200 | | 50/18522 | | | 0.1 | | | 9.261 | | | | 6.11112882161873 | | 0.000199388135671176 | | | 0.00243600287493915 | | | | 0.00195281743861015 | | CX3CL1/CCL21/CCL19/CCL5/CXCL13 | 5 |
| MF | GO:0008236 | | | serine-type peptidase activity | | | | 9/200 | | 191/18522 | | | 0.0471204188481675 | | | 4.3638219895288 | | | | 4.88222043845636 | | 0.000236822538071334 | | | 0.00277279721658521 | | | | 0.00222280803277481 | | C1R/C1S/MMP2/CTSK/HTRA1/MMP3/HTRA3/LTF/MMP7 | 9 |
| MF | GO:0017171 | | | serine hydrolase activity | | | | 9/200 | | 195/18522 | | | 0.0461538461538462 | | | 4.27430769230769 | | | | 4.80232865580832 | | 0.000276147709647126 | | | 0.00310390025643369 | | | | 0.00248823620482042 | | C1R/C1S/MMP2/CTSK/HTRA1/MMP3/HTRA3/LTF/MMP7 | 9 |
| MF | GO:0001664 | | | G protein-coupled receptor binding | | | | 11/200 | | 291/18522 | | | 0.0378006872852234 | | | 3.50072164948454 | | | | 4.49228578797192 | | 0.000335566225247638 | | | 0.00362669651133024 | | | | 0.00290733490700383 | | C3/CXCL12/CX3CL1/SAA1/CCL21/CCL19/SFRP1/CCL5/CXCL9/CXCL13/CXCL14 | 11 |
| MF | GO:0043394 | | | proteoglycan binding | | | | 4/200 | | 36/18522 | | | 0.111111111111111 | | | 10.29 | | | | 5.8291638687594 | | 0.000592877092952182 | | | 0.00617031344887271 | | | | 0.00494642097043926 | | CTSK/COL5A1/TNC/COMP | 4 |
| MF | GO:0023023 | | | MHC protein complex binding | | | | 4/200 | | 37/18522 | | | 0.108108108108108 | | | 10.0118918918919 | | | | 5.73281440509272 | | 0.000659170390199137 | | | 0.00623310826646002 | | | | 0.0049967603259878 | | HLA-DPB1/CD74/HLA-DRA/HLA-DRB5 | 4 |
| MF | GO:0030280 | | | structural constituent of skin epidermis | | | | 4/200 | | 37/18522 | | | 0.108108108108108 | | | 10.0118918918919 | | | | 5.73281440509272 | | 0.000659170390199137 | | | 0.00623310826646002 | | | | 0.0049967603259878 | | KRT7/KRT5/KRT6B/KRT81 | 4 |
| MF | GO:0030414 | | | peptidase inhibitor activity | | | | 8/200 | | 176/18522 | | | 0.0454545454545455 | | | 4.20954545454545 | | | | 4.46981907248452 | | 0.00066545639855445 | | | 0.00623310826646002 | | | | 0.0049967603259878 | | SERPING1/SERPINF1/C3/COL6A3/LTF/PI16/SLPI/RARRES1 | 8 |
| MF | GO:0061135 | | | endopeptidase regulator activity | | | | 8/200 | | 186/18522 | | | 0.043010752688172 | | | 3.98322580645161 | | | | 4.27219506003533 | | 0.000953513109249161 | | | 0.00864313495803272 | | | | 0.00692875400269339 | | SERPING1/SERPINF1/C3/SFRP2/COL6A3/LTF/SLPI/RARRES1 | 8 |
| MF | GO:0004175 | | | endopeptidase activity | | | | 12/200 | | 388/18522 | | | 0.0309278350515464 | | | 2.86422680412371 | | | | 3.87729262804902 | | 0.0010799794565729 | | | 0.00948356960303082 | | | | 0.00760248696403294 | | C1R/C1S/MMP2/ADAMTS2/CTSK/HTRA1/MMP3/HTRA3/SFRP1/LTF/MMP7/PIP | 12 |
| MF | GO:0005125 | | | cytokine activity | | | | 9/200 | | 238/18522 | | | 0.0378151260504202 | | | 3.50205882352941 | | | | 4.05892626821475 | | 0.00115609660761377 | | | 0.00984433777998392 | | | | 0.00789169614128698 | | CXCL12/CX3CL1/IL33/CCL21/CCL19/CCL5/CXCL9/CXCL13/CXCL14 | 9 |
| MF | GO:0004866 | | | endopeptidase inhibitor activity | | | | 7/200 | | 169/18522 | | | 0.0414201183431953 | | | 3.83591715976331 | | | | 3.86940452129029 | | 0.00243859584048278 | | | 0.0201542773875195 | | | | 0.0161566411722389 | | SERPING1/SERPINF1/C3/COL6A3/LTF/SLPI/RARRES1 | 7 |
| MF | GO:0016504 | | | peptidase activator activity | | | | 4/200 | | 55/18522 | | | 0.0727272727272727 | | | 6.73527272727273 | | | | 4.4503894665792 | | 0.00292692353627113 | | | 0.0229533993352665 | | | | 0.018400552379648 | | FBLN1/CAV1/CTSK/SFRP2 | 4 |
| MF | GO:0005126 | | | cytokine receptor binding | | | | 9/200 | | 273/18522 | | | 0.032967032967033 | | | 3.05307692307692 | | | | 3.5704896928518 | | 0.0029406490251587 | | | 0.0229533993352665 | | | | 0.018400552379648 | | CXCL12/CX3CL1/IL33/CCL21/CCL19/CCL5/CXCL9/CXCL13/CXCL14 | 9 |
| MF | GO:0004857 | | | enzyme inhibitor activity | | | | 11/200 | | 389/18522 | | | 0.0282776349614396 | | | 2.61879177377892 | | | | 3.37125600432451 | | 0.00345131513274924 | | | 0.0262113392514199 | | | | 0.0210122742079186 | | SERPING1/SERPINF1/C3/ANXA1/CAV1/COL6A3/LTF/PTN/PI16/SLPI/RARRES1 | 11 |
| MF | GO:0017147 | | | Wnt-protein binding | | | | 3/200 | | 32/18522 | | | 0.09375 | | | 8.6821875 | | | | 4.54414531181151 | | 0.00488498879990958 | | | 0.0356419902222048 | | | | 0.028572339043086 | | SFRP2/SFRP4/SFRP1 | 3 |
| MF | GO:0019955 | | | cytokine binding | | | | 6/200 | | 145/18522 | | | 0.0413793103448276 | | | 3.83213793103448 | | | | 3.57702411761146 | | 0.00494675309133803 | | | 0.0356419902222048 | | | | 0.028572339043086 | | BGN/ZFP36/CD74/ACKR1/CHRDL1/COMP | 6 |
| MF | GO:0050998 | | | nitric-oxide synthase binding | | | | 2/200 | | 11/18522 | | | 0.181818181818182 | | | 16.8381818181818 | | | | 5.48968595456533 | | 0.00598455169063382 | | | 0.0420414756267026 | | | | 0.0337024753104115 | | CAV1/CD74 | 2 |
| **Table S2. Differential methylation sites** | | | | | | | | | | | | | | | | | | | | | | | | | | | |
| **logFC** | | | | | **AveExpr** | | | | **t** | | | | | **P.Value** | | | | **adj.P.Val** | | | | | **B** | | | | |
| -1.162471443 | | | | | -1.067215674 | | | | -14.78625782 | | | | | 1.1014808538408e-43 | | | | 4.6446143163905e-38 | | | | | 88.2267519955175 | | | | |
| -1.027571405 | | | | | -1.733520584 | | | | -14.47919599 | | | | | 3.73713630428441e-42 | | | | 7.87919132713804e-37 | | | | | 84.7608032562232 | | | | |
| -1.157100458 | | | | | -1.353146244 | | | | -14.19459529 | | | | | 9.8141987805505e-41 | | | | 1.37945106659824e-35 | | | | | 81.5467119271353 | | | | |
| -1.265072457 | | | | | -1.240913245 | | | | -13.96679453 | | | | | 1.39638265859901e-39 | | | | 1.17762535130289e-34 | | | | | 78.9351472139766 | | | | |
| -1.778582552 | | | | | -0.224001423 | | | | -13.75158728 | | | | | 1.57555171785705e-38 | | | | 1.10727148811464e-33 | | | | | 76.5496410440284 | | | | |
| -1.12375036 | | | | | -2.098308195 | | | | -13.58328745 | | | | | 8.66136771100733e-38 | | | | 5.21748417528637e-33 | | | | | 74.8751841073105 | | | | |
| -1.054211839 | | | | | -1.127191558 | | | | -13.49940726 | | | | | 2.15072545545525e-37 | | | | 1.02515768353259e-32 | | | | | 73.9836763703918 | | | | |
| -1.053093927 | | | | | -1.409068506 | | | | -13.5038137 | | | | | 2.18806629634389e-37 | | | | 1.02515768353259e-32 | | | | | 73.9638609320492 | | | | |
| -1.213465937 | | | | | -0.369982489 | | | | -13.4821505 | | | | | 5.20233432571257e-37 | | | | 2.14766296782449e-32 | | | | | 73.0667799944497 | | | | |
| -1.156188651 | | | | | -1.206038683 | | | | -13.30318414 | | | | | 1.82809232434231e-36 | | | | 5.50608350289587e-32 | | | | | 71.8798738081048 | | | | |
| -1.050667344 | | | | | -1.905436583 | | | | -13.27000288 | | | | | 2.62023409947695e-36 | | | | 6.90546320454028e-32 | | | | | 71.525999312945 | | | | |
| -1.053123266 | | | | | -0.712299451 | | | | -13.26368744 | | | | | 2.80588437626791e-36 | | | | 6.95974861729934e-32 | | | | | 71.4587078664855 | | | | |
| -1.128573786 | | | | | -3.447049395 | | | | -13.24046515 | | | | | 3.60838561210373e-36 | | | | 8.00814716345147e-32 | | | | | 71.2114438298731 | | | | |
| -1.666749969 | | | | | -2.26734918 | | | | -13.1687786 | | | | | 7.83067819707868e-36 | | | | 1.43563568494007e-31 | | | | | 70.4498483598268 | | | | |
| -1.438264041 | | | | | -0.902890061 | | | | -13.16522373 | | | | | 8.81588308364139e-36 | | | | 1.54891392494961e-31 | | | | | 70.3330178379824 | | | | |
| -1.079042447 | | | | | -1.562606632 | | | | -13.08765182 | | | | | 2.11166183239635e-35 | | | | 3.29786831432063e-31 | | | | | 69.4675027887583 | | | | |
| -1.179799874 | | | | | -1.831534182 | | | | -13.01314736 | | | | | 4.17260817645078e-35 | | | | 6.06711617160001e-31 | | | | | 68.8053532171743 | | | | |
| -1.098959856 | | | | | -1.154858304 | | | | -13.00369915 | | | | | 4.84300931680749e-35 | | | | 6.58758625360715e-31 | | | | | 68.6565898320033 | | | | |
| -1.141190825 | | | | | -0.789748439 | | | | -12.89703855 | | | | | 1.45543066251771e-34 | | | | 1.65867958774012e-30 | | | | | 67.5774087907646 | | | | |
| -1.150241403 | | | | | -1.406237259 | | | | -12.89327836 | | | | | 1.50094442194859e-34 | | | | 1.66553482737648e-30 | | | | | 67.5471748290732 | | | | |
| -1.157966444 | | | | | -2.218177909 | | | | -12.78423741 | | | | | 4.77837051886649e-34 | | | | 4.38020760150094e-30 | | | | | 66.409106475189 | | | | |
| -1.071129923 | | | | | -2.251006618 | | | | -12.74948529 | | | | | 6.90228415248006e-34 | | | | 6.04051930572598e-30 | | | | | 66.047699954386 | | | | |
| -1.049800831 | | | | | -1.733506719 | | | | -12.6636569 | | | | | 1.70707471217848e-33 | | | | 1.28628635034293e-29 | | | | | 65.1578441232074 | | | | |
| 1.31158965313794 | | | | | 2.55486160062189 | | | | 12.628557656419 | | | | | 2.4693631151166e-33 | | | | 1.7648412622902e-29 | | | | | 64.7950621336064 | | | | |
| 1.04666939353014 | | | | | 1.24901285744324 | | | | 12.5903255008329 | | | | | 3.68895348415001e-33 | | | | 2.50890486397021e-29 | | | | | 64.4006436022718 | | | | |
| -1.176896119 | | | | | -1.663108824 | | | | -12.43168205 | | | | | 1.96632396784798e-32 | | | | 1.08758349280891e-28 | | | | | 62.7563551095207 | | | | |
| -1.190461077 | | | | | -0.992425703 | | | | -12.37353612 | | | | | 3.56737053846538e-32 | | | | 1.7491315522729e-28 | | | | | 62.1711173192997 | | | | |
| -1.045023991 | | | | | -0.929333371 | | | | -12.37177592 | | | | | 3.75185404438742e-32 | | | | 1.81844171827223e-28 | | | | | 62.116392457536 | | | | |
| -1.011545522 | | | | | -1.049619288 | | | | -12.35369533 | | | | | 4.34700669788368e-32 | | | | 2.05955316213103e-28 | | | | | 61.9769256985962 | | | | |
| -1.110612879 | | | | | -0.930796528 | | | | -12.3826645 | | | | | 4.31217496099773e-32 | | | | 2.05955316213103e-28 | | | | | 61.9696627956267 | | | | |
| -1.032213344 | | | | | -1.290049386 | | | | -12.33249876 | | | | | 5.41385323602195e-32 | | | | 2.50863680663008e-28 | | | | | 61.7612976670314 | | | | |
| -1.208132101 | | | | | -0.941274172 | | | | -12.33926066 | | | | | 5.64964161883326e-32 | | | | 2.58801559343514e-28 | | | | | 61.7159737773986 | | | | |
| 1.03923263407946 | | | | | 1.38611117111691 | | | | 12.3182505447099 | | | | | 6.27360572548481e-32 | | | | 2.78462244870019e-28 | | | | | 61.6164917963996 | | | | |
| 1.12538843812434 | | | | | 1.58222473468906 | | | | 12.2819423708029 | | | | | 9.12880444413228e-32 | | | | 3.92790098975231e-28 | | | | | 61.2479914880054 | | | | |
| -1.200579503 | | | | | -0.610756617 | | | | -12.26946124 | | | | | 1.0712106353196e-31 | | | | 4.55391068768742e-28 | | | | | 61.0876048827629 | | | | |
| -1.043059335 | | | | | -1.388476193 | | | | -12.26433831 | | | | | 1.09466406434819e-31 | | | | 4.57016827736338e-28 | | | | | 61.069584265572 | | | | |
| -1.003991668 | | | | | -0.667896299 | | | | -12.23403858 | | | | | 1.49572112320652e-31 | | | | 5.95000684926881e-28 | | | | | 60.7629132284423 | | | | |
| -1.208645149 | | | | | -3.218835916 | | | | -12.21927103 | | | | | 1.74118470156751e-31 | | | | 6.79819771398122e-28 | | | | | 60.6136307828833 | | | | |
| -1.448493611 | | | | | -0.038166161 | | | | -12.1999302 | | | | | 2.22405972684011e-31 | | | | 8.38811288757591e-28 | | | | | 60.3700271764125 | | | | |
| -1.067316004 | | | | | -3.60446253 | | | | -12.1537466 | | | | | 3.41216045232759e-31 | | | | 1.19900474827748e-27 | | | | | 59.9527129564305 | | | | |
| -1.122857034 | | | | | -1.681299799 | | | | -12.12876913 | | | | | 4.47319417620865e-31 | | | | 1.47360295959524e-27 | | | | | 59.6867456963175 | | | | |
| -1.98276919 | | | | | 0.731180238118645 | | | | -12.431418 | | | | | 4.50929747908794e-31 | | | | 1.47398098295117e-27 | | | | | 59.3917280217757 | | | | |
| -1.143772406 | | | | | -0.185826804 | | | | -12.09774059 | | | | | 6.09751865112901e-31 | | | | 1.91876170867281e-27 | | | | | 59.3824430443555 | | | | |
| -1.403050772 | | | | | 0.0114373888649157 | | | | -12.10477947 | | | | | 6.25766165440589e-31 | | | | 1.95456902949136e-27 | | | | | 59.3540045061172 | | | | |
| -1.015536549 | | | | | -1.71532274 | | | | -12.0874184 | | | | | 6.77542286498457e-31 | | | | 2.08539602881609e-27 | | | | | 59.2788896179967 | | | | |
| -1.583226926 | | | | | -0.096369537 | | | | -12.08030321 | | | | | 8.73783850022311e-31 | | | | 2.57656248978257e-27 | | | | | 59.0229796372733 | | | | |
| -1.063905987 | | | | | -0.752357867 | | | | -12.07826612 | | | | | 8.85132459218134e-31 | | | | 2.59190141721188e-27 | | | | | 59.0103109510661 | | | | |
| -1.285300821 | | | | | -0.483131535 | | | | -12.05035876 | | | | | 9.81595930198981e-31 | | | | 2.81571126453744e-27 | | | | | 58.9147451071322 | | | | |
| -1.130869326 | | | | | -0.898775855 | | | | -12.04604035 | | | | | 1.04844934461384e-30 | | | | 2.9871596969143e-27 | | | | | 58.8500458644307 | | | | |
| -1.036503852 | | | | | -1.488314595 | | | | -12.03197861 | | | | | 1.18370260371943e-30 | | | | 3.2622998490874e-27 | | | | | 58.7308434571433 | | | | |
| -1.209473569 | | | | | -1.153210247 | | | | -12.03488359 | | | | | 1.20062928092225e-30 | | | | 3.28746330445771e-27 | | | | | 58.716938941809 | | | | |
| -1.576192891 | | | | | -0.223108888 | | | | -12.06428033 | | | | | 1.25554976077566e-30 | | | | 3.41566237178242e-27 | | | | | 58.6484582443082 | | | | |
| -1.126827781 | | | | | -1.081876495 | | | | -12.01665836 | | | | | 1.44498343917419e-30 | | | | 3.8778731510838e-27 | | | | | 58.5349802205301 | | | | |
| -1.066173636 | | | | | -1.59557194 | | | | -12.00966067 | | | | | 1.48546009367349e-30 | | | | 3.91483723562062e-27 | | | | | 58.5077979691941 | | | | |
| -1.586016495 | | | | | -0.14568555 | | | | -12.21841668 | | | | | 1.45304137802367e-30 | | | | 3.8778731510838e-27 | | | | | 58.4289265042352 | | | | |
| -1.003273017 | | | | | -1.174835342 | | | | -11.97811783 | | | | | 2.04660212751318e-30 | | | | 5.23024678247566e-27 | | | | | 58.1930379530439 | | | | |
| -1.189695835 | | | | | -0.427027421 | | | | -11.95172254 | | | | | 2.75196833518983e-30 | | | | 6.7860964204649e-27 | | | | | 57.9022147141897 | | | | |
| -1.151850544 | | | | | -0.852110346 | | | | -11.92472086 | | | | | 3.64240186135685e-30 | | | | 8.72665677771786e-27 | | | | | 57.6255386966886 | | | | |
| -1.118041829 | | | | | -1.781597045 | | | | -11.89978184 | | | | | 4.52483583151777e-30 | | | | 1.04261613392137e-26 | | | | | 57.4137751267693 | | | | |
| -1.196090172 | | | | | -0.689105383 | | | | -11.89770514 | | | | | 4.65308883063468e-30 | | | | 1.057121544428e-26 | | | | | 57.3863365537639 | | | | |
| -1.169466743 | | | | | 0.414333902543359 | | | | -11.89852598 | | | | | 4.64699641346242e-30 | | | | 1.057121544428e-26 | | | | | 57.3862952191256 | | | | |
| -1.071809569 | | | | | -0.885381949 | | | | -11.89203736 | | | | | 4.89311216928693e-30 | | | | 1.09748862150171e-26 | | | | | 57.3369248589499 | | | | |
| -1.120526034 | | | | | -0.430728065 | | | | -11.86921514 | | | | | 6.16085729327111e-30 | | | | 1.34300243886813e-26 | | | | | 57.1106534838138 | | | | |
| -1.16604768 | | | | | -1.621445529 | | | | -11.88289279 | | | | | 6.80684735977506e-30 | | | | 1.47191965445967e-26 | | | | | 57.0075038655525 | | | | |
| -1.085035855 | | | | | -0.71479707 | | | | -11.84168763 | | | | | 8.35781669339836e-30 | | | | 1.77097515834436e-26 | | | | | 56.8098831882008 | | | | |
| -1.138358009 | | | | | -0.473063555 | | | | -11.83642383 | | | | | 8.57385184714548e-30 | | | | 1.79867468078897e-26 | | | | | 56.7860644561268 | | | | |
| -1.375770116 | | | | | -0.573720277 | | | | -11.83564713 | | | | | 8.70044574646996e-30 | | | | 1.81619651381881e-26 | | | | | 56.770388182485 | | | | |
| -1.055013714 | | | | | -1.099548012 | | | | -11.82738583 | | | | | 9.39053079985644e-30 | | | | 1.92218695260945e-26 | | | | | 56.6967088572489 | | | | |
| -1.044490602 | | | | | -1.265608413 | | | | -11.83200517 | | | | | 9.8778367656496e-30 | | | | 1.99291264544089e-26 | | | | | 56.644588462176 | | | | |
| -1.035542618 | | | | | -0.586565127 | | | | -11.81686746 | | | | | 1.05100090640091e-29 | | | | 2.09045071793429e-26 | | | | | 56.5861156863939 | | | | |
| -1.039601477 | | | | | -1.843297506 | | | | -11.80321954 | | | | | 1.2137584488134e-29 | | | | 2.39161460332311e-26 | | | | | 56.4447341328686 | | | | |
| -1.611406023 | | | | | 0.626111572368171 | | | | -11.87773583 | | | | | 1.22658709315243e-29 | | | | 2.40565106776552e-26 | | | | | 56.3945667884806 | | | | |
| 1.16881180858921 | | | | | 2.02949259751116 | | | | 11.795521713195 | | | | | 1.2937109118134e-29 | | | | 2.52555129714979e-26 | | | | | 56.382053161021 | | | | |
| -1.141047788 | | | | | -1.00153167 | | | | -11.80666625 | | | | | 1.32864711705832e-29 | | | | 2.56995701766048e-26 | | | | | 56.3535811324782 | | | | |
| -1.057801839 | | | | | -1.568093458 | | | | -11.81649811 | | | | | 1.34241391706345e-29 | | | | 2.58472911601892e-26 | | | | | 56.336358658294 | | | | |
| -1.044086014 | | | | | -0.658487436 | | | | -11.79082563 | | | | | 1.35619776425181e-29 | | | | 2.59939959660028e-26 | | | | | 56.3357290326575 | | | | |
| -1.035210464 | | | | | -0.345738941 | | | | -11.77712409 | | | | | 1.55618706081593e-29 | | | | 2.90879924210378e-26 | | | | | 56.2006438873104 | | | | |
| -1.168265412 | | | | | -0.23704394 | | | | -11.77638671 | | | | | 1.56774507211332e-29 | | | | 2.91220733285473e-26 | | | | | 56.1933770338884 | | | | |
| -1.531358269 | | | | | -0.207635436 | | | | -11.82595268 | | | | | 1.65422410261974e-29 | | | | 3.05937139189328e-26 | | | | | 56.1173044590048 | | | | |
| -1.053327381 | | | | | -1.230916982 | | | | -11.75213965 | | | | | 2.54935578854342e-29 | | | | 4.55502904811484e-26 | | | | | 55.7084065030563 | | | | |
| -1.143037609 | | | | | -1.678817707 | | | | -11.71756228 | | | | | 2.82620193669137e-29 | | | | 5.00724609514558e-26 | | | | | 55.6146753100558 | | | | |
| -1.126253542 | | | | | -1.350026476 | | | | -11.71320703 | | | | | 2.95200392872476e-29 | | | | 5.14368387035277e-26 | | | | | 55.5719088911992 | | | | |
| -1.37421798 | | | | | -0.219906422 | | | | -11.73772734 | | | | | 3.07325160818763e-29 | | | | 5.28937961479379e-26 | | | | | 55.520472678535 | | | | |
| -1.113975317 | | | | | -1.32616309 | | | | -11.70530687 | | | | | 3.19457492182737e-29 | | | | 5.45366966512934e-26 | | | | | 55.4943612796499 | | | | |
| -1.261472074 | | | | | 0.168319548107275 | | | | -11.65024131 | | | | | 5.56970103681979e-29 | | | | 8.76334267237239e-26 | | | | | 54.9485219855252 | | | | |
| -1.052305348 | | | | | -1.245075661 | | | | -11.64071079 | | | | | 6.08478151414443e-29 | | | | 9.50285118914548e-26 | | | | | 54.8616488583399 | | | | |
| -1.076579269 | | | | | -1.548295919 | | | | -11.60880406 | | | | | 8.57432668493777e-29 | | | | 1.28249274324834e-25 | | | | | 54.5249618616259 | | | | |
| -1.112436406 | | | | | -0.488122643 | | | | -11.61072564 | | | | | 8.57691924007003e-29 | | | | 1.28249274324834e-25 | | | | | 54.5224079092078 | | | | |
| -1.216318007 | | | | | -1.324554812 | | | | -11.60579896 | | | | | 8.61079074968743e-29 | | | | 1.28300782170343e-25 | | | | | 54.5207030249851 | | | | |
| -1.068051966 | | | | | -0.874904452 | | | | -11.60467756 | | | | | 8.70726299634306e-29 | | | | 1.29281393931971e-25 | | | | | 54.5097633442623 | | | | |
| 1.03708533931806 | | | | | 3.05574911008377 | | | | 11.5981299559968 | | | | | 9.29237886527357e-29 | | | | 1.36526738540763e-25 | | | | | 54.4459037172762 | | | | |
| -1.328610311 | | | | | -0.367298613 | | | | -11.58593813 | | | | | 1.0554635470509e-28 | | | | 1.53518846199483e-25 | | | | | 54.3208640768182 | | | | |
| 1.09546543196486 | | | | | 1.81552293204007 | | | | 11.585266234131 | | | | | 1.05581296743543e-28 | | | | 1.53518846199483e-25 | | | | | 54.3205152308157 | | | | |
| -1.145855292 | | | | | -1.179448122 | | | | -11.59470564 | | | | | 1.2037004116028e-28 | | | | 1.71474443432619e-25 | | | | | 54.1890278703279 | | | | |
| -1.079974635 | | | | | -0.688224002 | | | | -11.56738707 | | | | | 1.26066657185705e-28 | | | | 1.78984940523557e-25 | | | | | 54.1464001415564 | | | | |
| -1.031852779 | | | | | -0.972176549 | | | | -11.5482164 | | | | | 1.52435385310097e-28 | | | | 2.12136729121151e-25 | | | | | 53.9599162079947 | | | | |
| -1.097761553 | | | | | -1.694716711 | | | | -11.54085895 | | | | | 1.67062470822905e-28 | | | | 2.30967974006211e-25 | | | | | 53.8689329431757 | | | | |
| -1.124603057 | | | | | -0.904863313 | | | | -11.52301884 | | | | | 1.9559599587729e-28 | | | | 2.66054721230894e-25 | | | | | 53.715134276664 | | | | |
| -1.182519741 | | | | | -1.352762905 | | | | -11.52519562 | | | | | 2.00010980072492e-28 | | | | 2.71185305360668e-25 | | | | | 53.6911915709433 | | | | |
| -1.756590587 | | | | | -0.80181607 | | | | -11.75622301 | | | | | 2.30007677320318e-28 | | | | 3.06921953467274e-25 | | | | | 53.4660057470716 | | | | |
| -1.104598509 | | | | | -1.629261277 | | | | -11.48465589 | | | | | 2.8568856254349e-28 | | | | 3.72960669249887e-25 | | | | | 53.3431760208697 | | | | |
| -1.247141116 | | | | | -1.245482747 | | | | -11.45980283 | | | | | 3.67221565782597e-28 | | | | 4.67813648469933e-25 | | | | | 53.0967164438141 | | | | |
| -1.012192632 | | | | | -0.283695442 | | | | -11.4554292 | | | | | 3.83380779162028e-28 | | | | 4.82382691395729e-25 | | | | | 53.0544386660895 | | | | |
| -1.134564664 | | | | | -0.46161468 | | | | -11.47757288 | | | | | 3.96120070109616e-28 | | | | 4.94177366754798e-25 | | | | | 53.0151578218122 | | | | |
| -1.430123435 | | | | | -3.68129108 | | | | -11.44468831 | | | | | 4.23549897716227e-28 | | | | 5.22217208684215e-25 | | | | | 52.9565856050307 | | | | |
| -1.097647196 | | | | | -0.514514728 | | | | -11.43611932 | | | | | 4.60803249590466e-28 | | | | 5.57301083874298e-25 | | | | | 52.8738245509639 | | | | |
| -1.196058484 | | | | | -1.680419915 | | | | -11.43283993 | | | | | 4.75906563741927e-28 | | | | 5.70100911173462e-25 | | | | | 52.8421630411413 | | | | |
| -1.120144049 | | | | | -0.447020866 | | | | -11.43119713 | | | | | 4.83656436432944e-28 | | | | 5.77743369831953e-25 | | | | | 52.8263047542185 | | | | |
| -1.080784433 | | | | | -1.065502518 | | | | -11.42886531 | | | | | 4.94872616144663e-28 | | | | 5.86159932723933e-25 | | | | | 52.8037978808785 | | | | |
| -1.117073939 | | | | | -1.225977621 | | | | -11.43156251 | | | | | 5.2188056044433e-28 | | | | 6.11281599784891e-25 | | | | | 52.7499191460391 | | | | |
| 1.13392589886998 | | | | | 0.709149741119095 | | | | 11.4192683347968 | | | | | 5.43820871283151e-28 | | | | 6.29879574732574e-25 | | | | | 52.7112015064309 | | | | |
| 1.09177522382837 | | | | | 0.870270507736712 | | | | 11.4169021484812 | | | | | 5.56610919745529e-28 | | | | 6.41273569751632e-25 | | | | | 52.6883797821095 | | | | |
| -1.102850929 | | | | | -0.460595762 | | | | -11.41650682 | | | | | 5.62146249262493e-28 | | | | 6.45886127865165e-25 | | | | | 52.6776833075874 | | | | |
| -1.170886429 | | | | | -0.868181857 | | | | -11.42100614 | | | | | 5.68157029875658e-28 | | | | 6.510184097491e-25 | | | | | 52.66749879 | | | | |
| -1.09079103 | | | | | -0.682326254 | | | | -11.37565376 | | | | | 8.34283570231826e-28 | | | | 9.13746371583517e-25 | | | | | 52.2910774551709 | | | | |
| -1.191803809 | | | | | -0.361210408 | | | | -11.36223087 | | | | | 9.6283956853842e-28 | | | | 1.03308030754605e-24 | | | | | 52.1494771176188 | | | | |
| -1.10113217 | | | | | -1.004024674 | | | | -11.36107015 | | | | | 9.79637950157118e-28 | | | | 1.0457821125133e-24 | | | | | 52.1335055449731 | | | | |
| 1.03254716659524 | | | | | 2.56927999089149 | | | | 11.3548513303269 | | | | | 1.02883873881136e-27 | | | | 1.0927718664851e-24 | | | | | 52.0853380971693 | | | | |
| -1.091217466 | | | | | -1.654687855 | | | | -11.35839984 | | | | | 1.04861601988877e-27 | | | | 1.11097969122236e-24 | | | | | 52.0617195866096 | | | | |
| -1.033466468 | | | | | -2.046698174 | | | | -11.34801693 | | | | | 1.09352453625404e-27 | | | | 1.1527662280056e-24 | | | | | 52.0254493364092 | | | | |
| -1.150727171 | | | | | -1.966622428 | | | | -11.32687259 | | | | | 1.3446282881907e-27 | | | | 1.37618788903245e-24 | | | | | 51.8225316678817 | | | | |
| 1.63147511574651 | | | | | 1.45641411610639 | | | | 11.3083843622122 | | | | | 1.62004340149562e-27 | | | | 1.63036682842161e-24 | | | | | 51.6396533665567 | | | | |
| 1.25427607971354 | | | | | -2.142883057 | | | | 11.2989557460988 | | | | | 1.76584059508269e-27 | | | | 1.75200471465534e-24 | | | | | 51.5550306752635 | | | | |
| -1.349365377 | | | | | -1.095255197 | | | | -11.29660048 | | | | | 1.80687022499234e-27 | | | | 1.78850461918432e-24 | | | | | 51.5324838032082 | | | | |
| -1.074539679 | | | | | -0.593156686 | | | | -11.28770122 | | | | | 1.97063276459856e-27 | | | | 1.92797382331387e-24 | | | | | 51.447321576705 | | | | |
| -1.125022083 | | | | | -0.954467472 | | | | -11.28912 | | | | | 2.07262925703138e-27 | | | | 2.00911627313201e-24 | | | | | 51.3962642057205 | | | | |
| -1.115136875 | | | | | 0.407807601263765 | | | | -11.26987299 | | | | | 2.34437215871766e-27 | | | | 2.24161317044552e-24 | | | | | 51.2768562155182 | | | | |
| -1.110764529 | | | | | -0.763324274 | | | | -11.33218018 | | | | | 2.75926453963552e-27 | | | | 2.5970961572056e-24 | | | | | 51.0997504308266 | | | | |
| -1.103110752 | | | | | -0.162787675 | | | | -11.25422888 | | | | | 2.92616782053191e-27 | | | | 2.74194929974153e-24 | | | | | 51.0578364902833 | | | | |
| -1.056442378 | | | | | -1.799864556 | | | | -11.24008276 | | | | | 3.13231523814645e-27 | | | | 2.88385014512929e-24 | | | | | 50.9924435621211 | | | | |
| -1.057306092 | | | | | -0.598979692 | | | | -11.24668775 | | | | | 3.61608858095115e-27 | | | | 3.25811126480699e-24 | | | | | 50.8456081869211 | | | | |
| -1.047486902 | | | | | -0.467290693 | | | | -11.22001509 | | | | | 3.80630088628673e-27 | | | | 3.42207975952134e-24 | | | | | 50.8011562030367 | | | | |
| -1.237675245 | | | | | -0.625404692 | | | | -11.23298207 | | | | | 3.81430380860633e-27 | | | | 3.42207975952134e-24 | | | | | 50.7929030341689 | | | | |
| 1.02311103652244 | | | | | 1.71965912427884 | | | | 11.2100443685912 | | | | | 4.19291726342808e-27 | | | | 3.69880213905799e-24 | | | | | 50.7062048153843 | | | | |
| -1.059506551 | | | | | -0.02455232 | | | | -11.19764704 | | | | | 4.72843929426152e-27 | | | | 4.10255349220422e-24 | | | | | 50.5882287663041 | | | | |
| -1.287635733 | | | | | -0.157018852 | | | | -11.24402221 | | | | | 4.7815445790879e-27 | | | | 4.14011068308829e-24 | | | | | 50.5428268965536 | | | | |
| 1.32131661970669 | | | | | 0.795383365408489 | | | | 11.2005283501416 | | | | | 5.06809196133143e-27 | | | | 4.34362263685899e-24 | | | | | 50.5180451266076 | | | | |
| -1.092763389 | | | | | -0.708366459 | | | | -11.18297844 | | | | | 5.51170168670674e-27 | | | | 4.69519040451239e-24 | | | | | 50.4378617406935 | | | | |
| 1.11348921103563 | | | | | 1.82038556494889 | | | | 11.1802470716967 | | | | | 5.59648738952596e-27 | | | | 4.7578041079464e-24 | | | | | 50.4228037120522 | | | | |
| -1.200121985 | | | | | 0.179049471515461 | | | | -11.17670603 | | | | | 5.92264799800208e-27 | | | | 4.99409889444838e-24 | | | | | 50.3673588235635 | | | | |
| -1.271562261 | | | | | -0.425290383 | | | | -11.17192918 | | | | | 6.75753461147742e-27 | | | | 5.59813284797973e-24 | | | | | 50.2348724308952 | | | | |
| -1.03086647 | | | | | -0.307089154 | | | | -11.1604289 | | | | | 6.77935822448212e-27 | | | | 5.60519996572034e-24 | | | | | 50.2346123745484 | | | | |
| 1.23282497107419 | | | | | 1.38509046703833 | | | | 11.1547726161939 | | | | | 7.2001109369097e-27 | | | | 5.89528306556644e-24 | | | | | 50.1755529960338 | | | | |
| -1.205628145 | | | | | -0.450306833 | | | | -11.14775724 | | | | | 7.92225555790818e-27 | | | | 6.35090779677403e-24 | | | | | 50.0793395422927 | | | | |
| -1.231621481 | | | | | -0.685680231 | | | | -11.14543299 | | | | | 8.01210692946232e-27 | | | | 6.41074977029673e-24 | | | | | 50.0707939890811 | | | | |
| 1.06089104388021 | | | | | 2.80812815219361 | | | | 11.1364528051121 | | | | | 8.54660393335666e-27 | | | | 6.76143804986586e-24 | | | | | 50.0072574095371 | | | | |
| 1.14520613349454 | | | | | 1.44879213602694 | | | | 11.1353057329011 | | | | | 8.64177460427984e-27 | | | | 6.811172144648e-24 | | | | | 49.9963890010297 | | | | |
| -1.144746094 | | | | | -2.080117695 | | | | -11.13310552 | | | | | 8.82727835547956e-27 | | | | 6.89874328309096e-24 | | | | | 49.9755444682994 | | | | |
| -1.6824979 | | | | | 0.135751533719256 | | | | -11.15769714 | | | | | 8.83468440455598e-27 | | | | 6.89874328309096e-24 | | | | | 49.9625006994123 | | | | |
| 1.05098327264889 | | | | | 2.36388810474986 | | | | 11.1314670236709 | | | | | 8.96798846676459e-27 | | | | 6.9898922306481e-24 | | | | | 49.9600234056532 | | | | |
| -1.143616177 | | | | | -0.772408371 | | | | -11.12609267 | | | | | 9.49727041168911e-27 | | | | 7.36160664429586e-24 | | | | | 49.9037844389022 | | | | |
| 1.04534460522423 | | | | | 0.441834983097137 | | | | 11.1114094099988 | | | | | 1.09416692253181e-26 | | | | 8.3397530096441e-24 | | | | | 49.7640308566437 | | | | |
| -1.29226898 | | | | | -0.192601623 | | | | -11.11126446 | | | | | 1.09569643734267e-26 | | | | 8.3397530096441e-24 | | | | | 49.7634704756113 | | | | |
| -1.683590024 | | | | | 0.090592834468156 | | | | -11.22921843 | | | | | 1.17220695562082e-26 | | | | 8.84745510484726e-24 | | | | | 49.6553893985917 | | | | |
| -1.063281029 | | | | | -2.097907014 | | | | -11.09878801 | | | | | 1.22893520557636e-26 | | | | 9.20435360808853e-24 | | | | | 49.6508066613212 | | | | |
| -1.106074442 | | | | | -0.479238488 | | | | -11.08408561 | | | | | 1.41578840180282e-26 | | | | 1.05104840737358e-23 | | | | | 49.5119020781593 | | | | |
| 1.05161594550825 | | | | | 0.752358025513981 | | | | 11.0828548929277 | | | | | 1.43265380348241e-26 | | | | 1.06169970002536e-23 | | | | | 49.5002805647386 | | | | |
| 1.03845561780595 | | | | | 1.30404983307332 | | | | 11.0752472121383 | | | | | 1.54142771744328e-26 | | | | 1.13631787694809e-23 | | | | | 49.4284628049107 | | | | |
| -1.130149526 | | | | | -0.751050413 | | | | -11.06698511 | | | | | 1.6688661553808e-26 | | | | 1.21749271927236e-23 | | | | | 49.3505073851169 | | | | |
| 1.21792472078533 | | | | | 0.933771922062055 | | | | 11.0632732731099 | | | | | 1.72947510550627e-26 | | | | 1.25735822023936e-23 | | | | | 49.3154987390121 | | | | |
| -1.190567323 | | | | | -2.021937666 | | | | -11.05735319 | | | | | 1.83069718212581e-26 | | | | 1.31732095697438e-23 | | | | | 49.2596802021628 | | | | |
| -1.287683071 | | | | | 0.0752723047433195 | | | | -11.06011152 | | | | | 1.9675307213976e-26 | | | | 1.39911911671735e-23 | | | | | 49.186364557059 | | | | |
| -1.116790988 | | | | | -0.14948339 | | | | -11.03773917 | | | | | 2.23369373473727e-26 | | | | 1.57069144222264e-23 | | | | | 49.064516669141 | | | | |
| -1.145133943 | | | | | -0.898271939 | | | | -11.0479649 | | | | | 2.23495829756346e-26 | | | | 1.57069144222264e-23 | | | | | 49.0614178015725 | | | | |
| 1.21790781382231 | | | | | 0.890203559869537 | | | | 11.0379743124057 | | | | | 2.24062376315738e-26 | | | | 1.57205294876967e-23 | | | | | 49.0599624109506 | | | | |
| -1.235978067 | | | | | -0.360858888 | | | | -11.03428515 | | | | | 2.28444682316918e-26 | | | | 1.60013736200291e-23 | | | | | 49.0423846876542 | | | | |
| -1.324068721 | | | | | -0.509844118 | | | | -11.12972242 | | | | | 2.21920929100357e-26 | | | | 1.56746060592542e-23 | | | | | 49.0382365114868 | | | | |
| -1.307850429 | | | | | -0.803348471 | | | | -11.08640771 | | | | | 2.32370848335098e-26 | | | | 1.6249388991287e-23 | | | | | 49.0130185457371 | | | | |
| -1.173679778 | | | | | -0.422207034 | | | | -11.06237769 | | | | | 2.42147324019725e-26 | | | | 1.68377266622777e-23 | | | | | 48.9802408674989 | | | | |
| 1.0353298007389 | | | | | 0.811026032225929 | | | | 11.025922618407 | | | | | 2.475181589264e-26 | | | | 1.71380922946626e-23 | | | | | 48.9636924523168 | | | | |
| -1.227657845 | | | | | -1.250243446 | | | | -11.01894341 | | | | | 2.64641448428418e-26 | | | | 1.81744885274936e-23 | | | | | 48.8980503563427 | | | | |
| -1.388913025 | | | | | 0.204819269468284 | | | | -11.01700244 | | | | | 2.82888059316882e-26 | | | | 1.91777183234967e-23 | | | | | 48.8306586025296 | | | | |
| -1.162089461 | | | | | -2.946485506 | | | | -11.0045177 | | | | | 3.03852734672304e-26 | | | | 2.04347021737273e-23 | | | | | 48.7624663102981 | | | | |
| -1.126938694 | | | | | -0.789230164 | | | | -10.99988877 | | | | | 3.1761855110028e-26 | | | | 2.12250736041925e-23 | | | | | 48.7189872009565 | | | | |
| 1.0141330101346 | | | | | 1.02268715212597 | | | | 10.9979616800786 | | | | | 3.23530470755905e-26 | | | | 2.151783810783e-23 | | | | | 48.7008901039229 | | | | |
| 1.01601054737587 | | | | | 1.30542053029172 | | | | 10.9917394552372 | | | | | 3.43375281551571e-26 | | | | 2.26236023393517e-23 | | | | | 48.6424735711294 | | | | |
| -1.081550867 | | | | | -0.989174739 | | | | -10.9900678 | | | | | 3.69888070425855e-26 | | | | 2.42190532075265e-23 | | | | | 48.5684653944371 | | | | |
| 1.60450162115109 | | | | | 0.725591240453011 | | | | 10.9996940238714 | | | | | 3.68452137475577e-26 | | | | 2.41625525364427e-23 | | | | | 48.5676220709423 | | | | |
| 1.09615107000335 | | | | | 1.01560479355543 | | | | 10.9781329777718 | | | | | 3.91082864648215e-26 | | | | 2.54487517802798e-23 | | | | | 48.5148142868009 | | | | |
| -1.097084576 | | | | | -0.532765846 | | | | -10.97659579 | | | | | 3.98948101102956e-26 | | | | 2.58806839680129e-23 | | | | | 48.4953194190749 | | | | |
| -1.08697629 | | | | | -0.980155298 | | | | -10.9697973 | | | | | 4.23503881456784e-26 | | | | 2.73056394027343e-23 | | | | | 48.4366634900242 | | | | |
| -1.206368443 | | | | | -1.757024939 | | | | -10.96641529 | | | | | 4.39689119671721e-26 | | | | 2.82059510099139e-23 | | | | | 48.3999055603339 | | | | |
| -1.007269623 | | | | | -0.439431869 | | | | -10.96863608 | | | | | 4.51374973453535e-26 | | | | 2.88818338476711e-23 | | | | | 48.3715023484227 | | | | |
| -1.528277775 | | | | | -0.420040507 | | | | -10.98825617 | | | | | 4.77376120281507e-26 | | | | 3.03155404576962e-23 | | | | | 48.3155501560978 | | | | |
| -1.033989465 | | | | | -2.136792683 | | | | -10.94545537 | | | | | 5.37033018196301e-26 | | | | 3.34582954065043e-23 | | | | | 48.203666360762 | | | | |
| 1.21827586770434 | | | | | 1.82205161709695 | | | | 10.940343612048 | | | | | 5.69722387437919e-26 | | | | 3.53806832269436e-23 | | | | | 48.1457765684861 | | | | |
| 1.18500647700707 | | | | | 0.246910348351175 | | | | 10.9366339258111 | | | | | 5.84144871723815e-26 | | | | 3.60638899062637e-23 | | | | | 48.1211561188215 | | | | |
| -1.175750313 | | | | | -1.024146243 | | | | -10.93464117 | | | | | 5.9842452314681e-26 | | | | 3.67839167165183e-23 | | | | | 48.096806406619 | | | | |
| -1.147017431 | | | | | -0.495958411 | | | | -10.93323287 | | | | | 6.12821183293871e-26 | | | | 3.755934714528e-23 | | | | | 48.0728460854777 | | | | |
| -1.098200072 | | | | | -0.916019686 | | | | -10.92371942 | | | | | 6.60612832958151e-26 | | | | 3.99083973171151e-23 | | | | | 48.000449025303 | | | | |
| -1.363034491 | | | | | 0.463041236591434 | | | | -10.93174642 | | | | | 6.7281900959874e-26 | | | | 4.04718390552783e-23 | | | | | 47.9777670200148 | | | | |
| -1.2216158 | | | | | -0.792797925 | | | | -10.93276935 | | | | | 7.03624565532446e-26 | | | | 4.19656818314097e-23 | | | | | 47.935993056966 | | | | |
| -1.234399013 | | | | | -0.484897808 | | | | -10.92570173 | | | | | 7.44147608881934e-26 | | | | 4.42566981854086e-23 | | | | | 47.8764235941732 | | | | |
| -1.297537528 | | | | | 0.0258732824855009 | | | | -10.90763878 | | | | | 8.2795944306993e-26 | | | | 4.86925604406272e-23 | | | | | 47.775831834561 | | | | |
| 1.19844770967866 | | | | | -0.884328711 | | | | 10.8915179790115 | | | | | 8.9281402298044e-26 | | | | 5.22879012597447e-23 | | | | | 47.7048603563087 | | | | |
| -1.059909441 | | | | | -1.189907484 | | | | -10.88954162 | | | | | 9.23727597305561e-26 | | | | 5.37994773419663e-23 | | | | | 47.6702377419638 | | | | |
| -1.164618209 | | | | | -0.800836967 | | | | -10.88305551 | | | | | 9.72470836742017e-26 | | | | 5.62913715736674e-23 | | | | | 47.6203916200922 | | | | |
| -1.04582859 | | | | | -0.927563662 | | | | -10.97687504 | | | | | 9.6526964872115e-26 | | | | 5.60640844044418e-23 | | | | | 47.6103964822939 | | | | |
| -1.214447823 | | | | | -0.389388927 | | | | -10.89126125 | | | | | 9.82494214087122e-26 | | | | 5.66724566809228e-23 | | | | | 47.6096705359875 | | | | |
| -1.076358796 | | | | | -1.003142339 | | | | -10.88074467 | | | | | 9.94045462757814e-26 | | | | 5.71061512644533e-23 | | | | | 47.5995260663094 | | | | |
| 1.30571254919402 | | | | | 1.65291552293149 | | | | 10.8798292092132 | | | | | 9.97664646624894e-26 | | | | 5.72360886452135e-23 | | | | | 47.5959134448354 | | | | |
| -1.050721365 | | | | | -1.212898577 | | | | -10.87672196 | | | | | 1.02753761828129e-25 | | | | 5.86308237483994e-23 | | | | | 47.566966183413 | | | | |
| -1.148269078 | | | | | -0.688141689 | | | | -10.88016394 | | | | | 1.03060520353978e-25 | | | | 5.86500582881865e-23 | | | | | 47.5630123848701 | | | | |
| -1.214323168 | | | | | -0.771496506 | | | | -10.92869602 | | | | | 1.02514134019552e-25 | | | | 5.85737201783767e-23 | | | | | 47.5575267787686 | | | | |
| -1.16296861 | | | | | -0.039459006 | | | | -10.87176688 | | | | | 1.11031973747406e-25 | | | | 6.29285650135332e-23 | | | | | 47.4891687416011 | | | | |
| -1.128562764 | | | | | -1.050333352 | | | | -10.86263213 | | | | | 1.27481752621327e-25 | | | | 7.06376223756042e-23 | | | | | 47.354812401365 | | | | |
| 1.00036582611357 | | | | | 0.748357263926353 | | | | 10.8522046487112 | | | | | 1.29660038699409e-25 | | | | 7.16562890149143e-23 | | | | | 47.338773325882 | | | | |
| -1.095583822 | | | | | -0.894612122 | | | | -10.87653747 | | | | | 1.32335924001279e-25 | | | | 7.27537015301425e-23 | | | | | 47.3146735463436 | | | | |
| 1.05706303705236 | | | | | 2.06380494543155 | | | | 10.8474850665329 | | | | | 1.3559120933497e-25 | | | | 7.40605508293742e-23 | | | | | 47.2948892910459 | | | | |
| -1.608125569 | | | | | 0.447892502375277 | | | | -10.92504504 | | | | | 1.31231555548884e-25 | | | | 7.23351765075791e-23 | | | | | 47.2935256041956 | | | | |
| -1.009461263 | | | | | -0.338067242 | | | | -10.84526464 | | | | | 1.39867436500764e-25 | | | | 7.60024509655632e-23 | | | | | 47.264521099981 | | | | |
| -1.130402056 | | | | | -3.323684745 | | | | -10.84105949 | | | | | 1.44101631008322e-25 | | | | 7.7801965105351e-23 | | | | | 47.2351648535227 | | | | |
| -1.190801253 | | | | | -0.53896078 | | | | -10.85355825 | | | | | 1.45638812374788e-25 | | | | 7.85313529591777e-23 | | | | | 47.2198946583221 | | | | |
| -1.203887253 | | | | | -0.993212137 | | | | -10.83795088 | | | | | 1.48407192373076e-25 | | | | 7.97182940228724e-23 | | | | | 47.2062801485662 | | | | |
| -1.303787468 | | | | | -1.589981184 | | | | -10.83779397 | | | | | 1.48627872690386e-25 | | | | 7.97352609126657e-23 | | | | | 47.2048223387939 | | | | |
| -1.002334759 | | | | | -1.041797877 | | | | -10.83396481 | | | | | 1.54885059918121e-25 | | | | 8.26713711590813e-23 | | | | | 47.1644123420781 | | | | |
| -1.173204753 | | | | | -1.068986979 | | | | -10.82751611 | | | | | 1.64631765383688e-25 | | | | 8.71019780543788e-23 | | | | | 47.1045383310997 | | | | |
| -1.1359418 | | | | | -1.390176602 | | | | -10.82597943 | | | | | 1.66214989206557e-25 | | | | 8.77147779704413e-23 | | | | | 47.0951000475794 | | | | |
| -1.306014808 | | | | | -1.550889887 | | | | -10.8919567 | | | | | 1.64527272315189e-25 | | | | 8.71019780543788e-23 | | | | | 47.0906874033575 | | | | |
| -1.212248591 | | | | | -0.554730758 | | | | -10.82911227 | | | | | 2.05491871191205e-25 | | | | 1.06318720644411e-22 | | | | | 46.8823209432388 | | | | |
| -1.066254284 | | | | | -0.405281727 | | | | -10.80296181 | | | | | 2.07648202572043e-25 | | | | 1.07171380145108e-22 | | | | | 46.8767978842328 | | | | |
| -1.019422037 | | | | | -1.044182684 | | | | -10.81262511 | | | | | 2.17607679340991e-25 | | | | 1.1203739944776e-22 | | | | | 46.8271456106407 | | | | |
| -1.103291795 | | | | | -1.174615567 | | | | -10.79634748 | | | | | 2.19948358133028e-25 | | | | 1.13104419724334e-22 | | | | | 46.8202913129896 | | | | |
| -1.052101006 | | | | | -0.818774406 | | | | -10.80024137 | | | | | 2.22829318091086e-25 | | | | 1.14329433199863e-22 | | | | | 46.8067592935032 | | | | |
| -1.259256447 | | | | | -0.14119326 | | | | -10.79851421 | | | | | 2.2535143216934e-25 | | | | 1.15320313595687e-22 | | | | | 46.7950044661092 | | | | |
| -1.232846529 | | | | | -1.246898119 | | | | -10.79828309 | | | | | 2.26981950186778e-25 | | | | 1.1601391386092e-22 | | | | | 46.7879799154513 | | | | |
| -1.116194675 | | | | | -1.331850071 | | | | -10.79089737 | | | | | 2.32702958364521e-25 | | | | 1.18650370560541e-22 | | | | | 46.7650390367351 | | | | |
| 1.27129722547771 | | | | | 2.45180103264975 | | | | 10.7853438253089 | | | | | 2.44024810734038e-25 | | | | 1.23973424026774e-22 | | | | | 46.7183829317209 | | | | |
| -1.127750445 | | | | | -1.062135161 | | | | -10.78699143 | | | | | 2.47477647992012e-25 | | | | 1.25576293416115e-22 | | | | | 46.7048977832909 | | | | |
| 1.12365037308918 | | | | | 1.35821803265884 | | | | 10.7830804229609 | | | | | 2.49292652043503e-25 | | | | 1.26345231474981e-22 | | | | | 46.6974302723037 | | | | |
| -1.145205468 | | | | | -0.70463918 | | | | -10.76684104 | | | | | 2.91966640681293e-25 | | | | 1.45352506937522e-22 | | | | | 46.5424663030102 | | | | |
| -1.045553962 | | | | | -0.554408551 | | | | -10.76192598 | | | | | 3.04321980332288e-25 | | | | 1.50791362452075e-22 | | | | | 46.5017561872726 | | | | |
| -1.419523087 | | | | | -0.752631944 | | | | -10.76590935 | | | | | 3.0943864834901e-25 | | | | 1.52967168639305e-22 | | | | | 46.4847372382589 | | | | |
| -1.597448392 | | | | | 1.30891213295283 | | | | -10.76111286 | | | | | 3.23717180218603e-25 | | | | 1.59464746942498e-22 | | | | | 46.4411099483874 | | | | |
| -1.06956629 | | | | | -0.387022801 | | | | -10.7578384 | | | | | 3.38882481505641e-25 | | | | 1.65965825756659e-22 | | | | | 46.3963513543247 | | | | |
| 1.26498524393392 | | | | | 2.62102810708622 | | | | 10.7423361702867 | | | | | 3.65966283172155e-25 | | | | 1.78401159104281e-22 | | | | | 46.3208058373635 | | | | |
| 1.08948914770951 | | | | | 1.28006242424482 | | | | 10.7387467159583 | | | | | 3.78536596826085e-25 | | | | 1.83257780463439e-22 | | | | | 46.2876764122618 | | | | |
| -1.079351063 | | | | | -1.401117456 | | | | -10.74063694 | | | | | 4.14671203199938e-25 | | | | 1.98247626137549e-22 | | | | | 46.1961746301167 | | | | |
| -1.294191875 | | | | | -1.097280781 | | | | -10.72857322 | | | | | 4.18549383868895e-25 | | | | 1.99875106110982e-22 | | | | | 46.1885909762652 | | | | |
| -1.018786204 | | | | | -0.207401251 | | | | -10.71772843 | | | | | 4.61231890770626e-25 | | | | 2.16820124170847e-22 | | | | | 46.0938481313104 | | | | |
| 1.14005725824438 | | | | | 2.37552222128561 | | | | 10.7082969168145 | | | | | 5.03948313393353e-25 | | | | 2.35066244810371e-22 | | | | | 46.0069624688529 | | | | |
| 1.65419800340898 | | | | | -3.601611816 | | | | 10.7042283656705 | | | | | 5.23566843342292e-25 | | | | 2.4260706684851e-22 | | | | | 45.9694992447576 | | | | |
| -1.025589803 | | | | | -0.955928553 | | | | -10.69166246 | | | | | 6.30233921149322e-25 | | | | 2.85753481216166e-22 | | | | | 45.7872204088846 | | | | |
| 1.09486007343888 | | | | | 1.01772325121056 | | | | 10.6837224644191 | | | | | 6.34597879603769e-25 | | | | 2.87423080443095e-22 | | | | | 45.7808403690829 | | | | |
| -1.122957018 | | | | | -0.869824759 | | | | -10.6684716 | | | | | 7.32056683243398e-25 | | | | 3.26887693574007e-22 | | | | | 45.6407018305233 | | | | |
| -1.114056687 | | | | | -0.861363483 | | | | -10.6704161 | | | | | 7.32584415366131e-25 | | | | 3.26887693574007e-22 | | | | | 45.6391196593639 | | | | |
| -1.313188866 | | | | | -0.535903609 | | | | -10.69270752 | | | | | 7.38822519318451e-25 | | | | 3.28627944853387e-22 | | | | | 45.6296314200603 | | | | |
| -1.231297467 | | | | | -1.314161575 | | | | -10.66579413 | | | | | 7.5774820846017e-25 | | | | 3.36336512696211e-22 | | | | | 45.6069740568977 | | | | |
| -1.047850373 | | | | | -0.836944268 | | | | -10.66012964 | | | | | 7.91508833325454e-25 | | | | 3.50215666052827e-22 | | | | | 45.5641108051124 | | | | |
| -1.033524287 | | | | | -0.790778985 | | | | -10.65443916 | | | | | 8.38713863317022e-25 | | | | 3.69291732812785e-22 | | | | | 45.5073432074247 | | | | |
| 1.48320261737541 | | | | | -1.16764918 | | | | 10.6512761365984 | | | | | 9.01553572317993e-25 | | | | 3.93131432098581e-22 | | | | | 45.4358883719595 | | | | |
| 1.12241472194576 | | | | | 1.05225608024833 | | | | 10.6413076746275 | | | | | 9.43840574045532e-25 | | | | 4.10298200884309e-22 | | | | | 45.3914610431671 | | | | |
| -1.129587756 | | | | | -0.545883915 | | | | -10.65374182 | | | | | 9.62341170258047e-25 | | | | 4.17479836690031e-22 | | | | | 45.3716432410792 | | | | |
| -1.374503419 | | | | | -0.881553923 | | | | -10.63693622 | | | | | 1.05566347529982e-24 | | | | 4.54226140438445e-22 | | | | | 45.2808256442425 | | | | |
| -1.024233329 | | | | | -0.675537247 | | | | -10.63558121 | | | | | 1.12260259493579e-24 | | | | 4.81064874193672e-22 | | | | | 45.218501920388 | | | | |
| 1.12863644590455 | | | | | 1.76027560294042 | | | | 10.61866303 | | | | | 1.16610345416313e-24 | | | | 4.98692539063862e-22 | | | | | 45.184045274761 | | | | |
| 1.28681967282589 | | | | | 2.58385907410757 | | | | 10.613133306777 | | | | | 1.22784081636659e-24 | | | | 5.22445647868112e-22 | | | | | 45.1334448758981 | | | | |
| 1.13189524638574 | | | | | 1.01187487176723 | | | | 10.6120837203238 | | | | | 1.23992026811148e-24 | | | | 5.27053608321137e-22 | | | | | 45.1238427076884 | | | | |
| -1.452991566 | | | | | 0.585119385806006 | | | | -10.59754749 | | | | | 1.41986193567674e-24 | | | | 5.93961490492869e-22 | | | | | 44.9909298963949 | | | | |
| 1.97147293782351 | | | | | -4.806938954 | | | | 10.5966937942682 | | | | | 1.43120099872817e-24 | | | | 5.98111521440742e-22 | | | | | 44.9831282628431 | | | | |
| -1.007407643 | | | | | -0.802673534 | | | | -10.58375368 | | | | | 1.64444703057201e-24 | | | | 6.78487259668588e-22 | | | | | 44.846649817845 | | | | |
| 1.00634413940954 | | | | | 1.2965224361839 | | | | 10.5812950661453 | | | | | 1.6518758190532e-24 | | | | 6.80886096402898e-22 | | | | | 44.8424846633749 | | | | |
| -1.004073176 | | | | | -3.266373249 | | | | -10.58041377 | | | | | 1.66548065649672e-24 | | | | 6.83820086100266e-22 | | | | | 44.8344400124059 | | | | |
| 1.13825603490028 | | | | | 3.43868279369612 | | | | 10.5771951428531 | | | | | 1.71611954374377e-24 | | | | 7.01877912716234e-22 | | | | | 44.8050637366885 | | | | |
| -1.097128778 | | | | | -0.997401136 | | | | -10.56797422 | | | | | 1.86981015087731e-24 | | | | 7.57389861979287e-22 | | | | | 44.720941564339 | | | | |
| -1.041714539 | | | | | -0.346387388 | | | | -10.56772456 | | | | | 1.89132017542263e-24 | | | | 7.64633728063719e-22 | | | | | 44.70936868 | | | | |
| -1.247975297 | | | | | -0.409786136 | | | | -10.69798744 | | | | | 1.93744119388236e-24 | | | | 7.81033296581619e-22 | | | | | 44.6222422183713 | | | | |
| -1.155009925 | | | | | -0.395763584 | | | | -10.57307001 | | | | | 2.13790296741395e-24 | | | | 8.52069512542003e-22 | | | | | 44.5883631247765 | | | | |
| -1.324815757 | | | | | -0.310997191 | | | | -10.6490241 | | | | | 2.21543895951407e-24 | | | | 8.77992618475843e-22 | | | | | 44.5266803858628 | | | | |
| -1.414136816 | | | | | -0.625577157 | | | | -10.63677647 | | | | | 2.32293893391682e-24 | | | | 9.14578581012798e-22 | | | | | 44.4963099651428 | | | | |
| -1.28979028 | | | | | -0.026963608 | | | | -10.66213024 | | | | | 2.37144650136992e-24 | | | | 9.30202647658282e-22 | | | | | 44.4562878922169 | | | | |
| -1.1563184 | | | | | -0.997726915 | | | | -10.53040018 | | | | | 2.65058478944329e-24 | | | | 1.02821719242369e-21 | | | | | 44.3787177721144 | | | | |
| -1.142502522 | | | | | -0.417328446 | | | | -10.52420884 | | | | | 2.80721038865196e-24 | | | | 1.08299762541891e-21 | | | | | 44.3224140355838 | | | | |
| -1.161198506 | | | | | -0.585017351 | | | | -10.52337003 | | | | | 2.82912494935557e-24 | | | | 1.08945855469842e-21 | | | | | 44.3147878299429 | | | | |
| -1.235562328 | | | | | -0.586544855 | | | | -10.52453869 | | | | | 2.87545500106108e-24 | | | | 1.10427423524356e-21 | | | | | 44.2978039572674 | | | | |
| -1.009656024 | | | | | -0.737113431 | | | | -10.56162281 | | | | | 3.03062506611998e-24 | | | | 1.15753955763661e-21 | | | | | 44.2406565229297 | | | | |
| -1.475685574 | | | | | -0.353283241 | | | | -10.51849711 | | | | | 3.15440124221623e-24 | | | | 1.19614781637169e-21 | | | | | 44.2079539750967 | | | | |
| -1.01627586 | | | | | -1.229138687 | | | | -10.53378044 | | | | | 3.52645396016574e-24 | | | | 1.32060376677006e-21 | | | | | 44.0979517243765 | | | | |
| 1.06274552176568 | | | | | 2.37949399074226 | | | | 10.4944369983315 | | | | | 3.69845448917508e-24 | | | | 1.37403286735723e-21 | | | | | 44.0520144113048 | | | | |
| 1.13853882065035 | | | | | 2.39145337979161 | | | | 10.492966526934 | | | | | 3.74911053387171e-24 | | | | 1.38836936423291e-21 | | | | | 44.0386737830879 | | | | |
| -1.209125961 | | | | | -0.266062458 | | | | -10.48656381 | | | | | 4.21704492150517e-24 | | | | 1.54760777376073e-21 | | | | | 43.9236709811416 | | | | |
| -1.151717645 | | | | | -0.693881608 | | | | -10.47722227 | | | | | 4.43379933081013e-24 | | | | 1.61870144053914e-21 | | | | | 43.8744733545188 | | | | |
| -1.091839284 | | | | | -0.506319032 | | | | -10.47532925 | | | | | 4.47201234634496e-24 | | | | 1.63124000526235e-21 | | | | | 43.865529925951 | | | | |
| -1.003950652 | | | | | -0.862602954 | | | | -10.47163004 | | | | | 4.56651158783936e-24 | | | | 1.65996632865881e-21 | | | | | 43.8452584382781 | | | | |
| -1.339995622 | | | | | -0.376334498 | | | | -10.46921236 | | | | | 4.71096226509466e-24 | | | | 1.71100039476526e-21 | | | | | 43.8148362498784 | | | | |
| 1.00422649481992 | | | | | 2.89975817177348 | | | | 10.4684706700225 | | | | | 4.72245663098486e-24 | | | | 1.71369904267417e-21 | | | | | 43.8123877623245 | | | | |
| -1.309411139 | | | | | -1.314709777 | | | | -10.46984766 | | | | | 4.76731734107621e-24 | | | | 1.72849071643302e-21 | | | | | 43.8030013879522 | | | | |
| -1.132467959 | | | | | -0.127650873 | | | | -10.46557629 | | | | | 4.8503526672405e-24 | | | | 1.75407222057916e-21 | | | | | 43.7861830966127 | | | | |
| -1.166058876 | | | | | 0.039622377172982 | | | | -10.46689164 | | | | | 4.92102316336901e-24 | | | | 1.77506230735484e-21 | | | | | 43.7715135475624 | | | | |
| -1.164647888 | | | | | -0.788726366 | | | | -10.55286307 | | | | | 4.98940879775938e-24 | | | | 1.79819145961641e-21 | | | | | 43.742965856305 | | | | |
| -1.035131334 | | | | | -0.156222163 | | | | -10.45935673 | | | | | 5.15956106044355e-24 | | | | 1.85158473482411e-21 | | | | | 43.725239461938 | | | | |
| -1.493954339 | | | | | 0.0409533546401321 | | | | -10.46780479 | | | | | 5.15261885799884e-24 | | | | 1.85068551435466e-21 | | | | | 43.7245552130284 | | | | |
| -1.11460263 | | | | | -0.637920877 | | | | -10.45707615 | | | | | 5.24616540552496e-24 | | | | 1.87629394957397e-21 | | | | | 43.7092572191226 | | | | |
| 1.04434535487687 | | | | | 0.4843960836101 | | | | 10.4562776855316 | | | | | 5.26184928624106e-24 | | | | 1.88030846485531e-21 | | | | | 43.7062709510528 | | | | |
| 1.13617505683109 | | | | | 2.47395568840559 | | | | 10.4560509893079 | | | | | 5.27286712411005e-24 | | | | 1.88265019493945e-21 | | | | | 43.7042197706287 | | | | |
| -1.162568827 | | | | | -0.933518464 | | | | -10.45348981 | | | | | 5.39894681031303e-24 | | | | 1.92440735545621e-21 | | | | | 43.6810481494803 | | | | |
| -1.222683598 | | | | | -0.317427853 | | | | -10.48722427 | | | | | 5.75405585021757e-24 | | | | 2.03208771387039e-21 | | | | | 43.6167272702928 | | | | |
| 1.02497349478782 | | | | | 0.339193112834312 | | | | 10.4383202011804 | | | | | 6.20945402056388e-24 | | | | 2.18013361935984e-21 | | | | | 43.5438918708973 | | | | |
| -1.240510901 | | | | | 0.410078630886054 | | | | -10.47726027 | | | | | 6.26751779786418e-24 | | | | 2.19868904311596e-21 | | | | | 43.5249191390542 | | | | |
| -1.479941081 | | | | | -0.203406962 | | | | -10.48569061 | | | | | 6.37015824133061e-24 | | | | 2.2241402104544e-21 | | | | | 43.5117572533728 | | | | |
| 1.07106316615332 | | | | | 1.99036409062767 | | | | 10.4317488047793 | | | | | 6.59701553706771e-24 | | | | 2.29329228484364e-21 | | | | | 43.4845226601591 | | | | |
| -1.288368398 | | | | | 0.181255195554016 | | | | -10.44524536 | | | | | 6.67192126875127e-24 | | | | 2.31170833310957e-21 | | | | | 43.4707040840314 | | | | |
| -1.095992049 | | | | | -0.277746679 | | | | -10.42976932 | | | | | 6.74762514747449e-24 | | | | 2.33218942289801e-21 | | | | | 43.4620662056731 | | | | |
| -1.019721113 | | | | | -0.932268181 | | | | -10.42585277 | | | | | 6.96512545876765e-24 | | | | 2.39753832832535e-21 | | | | | 43.4312787938115 | | | | |
| -1.148157367 | | | | | -0.172441404 | | | | -10.4225594 | | | | | 7.27364544888622e-24 | | | | 2.49558834534732e-21 | | | | | 43.388958834924 | | | | |
| -1.063425379 | | | | | -0.003937069 | | | | -10.4182321 | | | | | 7.47125666996042e-24 | | | | 2.55697601715506e-21 | | | | | 43.3624940212201 | | | | |
| 1.02132967962745 | | | | | 1.3956113415911 | | | | 10.417425842196 | | | | | 7.52689477666117e-24 | | | | 2.57201436019021e-21 | | | | | 43.3552188443834 | | | | |
| 1.19820582787632 | | | | | 0.590326032751759 | | | | 10.4162247795588 | | | | | 7.61054051438362e-24 | | | | 2.59849118923088e-21 | | | | | 43.3443820139544 | | | | |
| -1.362948619 | | | | | 0.257437740904663 | | | | -10.43449176 | | | | | 8.30717657105933e-24 | | | | 2.80455335846164e-21 | | | | | 43.2528805119984 | | | | |
| -1.051368421 | | | | | -0.547747105 | | | | -10.40155573 | | | | | 8.70969279683658e-24 | | | | 2.92638737979449e-21 | | | | | 43.2121030066461 | | | | |
| -1.22430654 | | | | | -0.750604571 | | | | -10.49201932 | | | | | 9.34688530112387e-24 | | | | 3.10583224974382e-21 | | | | | 43.1233836739563 | | | | |
| -1.143260955 | | | | | -0.286412669 | | | | -10.39258681 | | | | | 9.7057335003101e-24 | | | | 3.21667310626867e-21 | | | | | 43.1051440046212 | | | | |
| 1.07145116461387 | | | | | 1.25832966768617 | | | | 10.3822281506768 | | | | | 1.04461893549788e-23 | | | | 3.43591627559588e-21 | | | | | 43.0339029271723 | | | | |
| 1.05745394409729 | | | | | 1.47091569038036 | | | | 10.3807707356288 | | | | | 1.07239206379973e-23 | | | | 3.5081114161554e-21 | | | | | 43.0083584360577 | | | | |
| -1.241589704 | | | | | -0.25222008 | | | | -10.37960738 | | | | | 1.07924848688879e-23 | | | | 3.5278039493519e-21 | | | | | 43.0016959825918 | | | | |
| -1.080299191 | | | | | -1.19534382 | | | | -10.40515287 | | | | | 1.11267673615003e-23 | | | | 3.63015378456225e-21 | | | | | 42.9711998060506 | | | | |
| -1.099305678 | | | | | 0.0737085594656263 | | | | -10.36294625 | | | | | 1.25734949785099e-23 | | | | 4.05651539983802e-21 | | | | | 42.8522870155698 | | | | |
| -1.04295589 | | | | | -2.541710579 | | | | -10.35753725 | | | | | 1.30450604016808e-23 | | | | 4.17036438178676e-21 | | | | | 42.816002125569 | | | | |
| 1.02589172895183 | | | | | 2.71742436365851 | | | | 10.3567612701382 | | | | | 1.31381427089192e-23 | | | | 4.19693987581057e-21 | | | | | 42.8090307940567 | | | | |
| -1.066431982 | | | | | -1.219995743 | | | | -10.36160221 | | | | | 1.34668282177116e-23 | | | | 4.29868088914646e-21 | | | | | 42.7842657383394 | | | | |
| -1.109008243 | | | | | -0.13617542 | | | | -10.35097871 | | | | | 1.47104545553883e-23 | | | | 4.64989308273657e-21 | | | | | 42.6971403854366 | | | | |
| 1.26966568097333 | | | | | 3.40960646042926 | | | | 10.33637788 | | | | | 1.58338167892386e-23 | | | | 4.96404871785742e-21 | | | | | 42.6260479239447 | | | | |
| -1.277297089 | | | | | -0.475193221 | | | | -10.35951005 | | | | | 1.61223751232577e-23 | | | | 5.04089071969885e-21 | | | | | 42.6061742840637 | | | | |
| -1.122145831 | | | | | -0.369036589 | | | | -10.33082358 | | | | | 1.77598031149222e-23 | | | | 5.5186265139788e-21 | | | | | 42.5129941254899 | | | | |
| -1.084658646 | | | | | -0.663032072 | | | | -10.32269955 | | | | | 1.80187919842217e-23 | | | | 5.59498086596963e-21 | | | | | 42.4993714077199 | | | | |
| -1.230144918 | | | | | 0.412948565582052 | | | | -10.3177234 | | | | | 1.90957690966312e-23 | | | | 5.88174795834658e-21 | | | | | 42.441993124933 | | | | |
| -1.355001529 | | | | | -0.503433292 | | | | -10.33910407 | | | | | 1.91288020614644e-23 | | | | 5.88762187245088e-21 | | | | | 42.437898859418 | | | | |
| 1.05240740365303 | | | | | 1.97702497961927 | | | | 10.3119299474904 | | | | | 1.97990443586958e-23 | | | | 6.05853630967436e-21 | | | | | 42.406934680684 | | | | |
| 1.00199832907179 | | | | | 0.102726925571455 | | | | 10.3072869616169 | | | | | 2.07426378988339e-23 | | | | 6.30154763890584e-21 | | | | | 42.3613518413007 | | | | |
| -1.133304568 | | | | | 0.0363767856503691 | | | | -10.30731241 | | | | | 2.0737819011195e-23 | | | | 6.30154763890584e-21 | | | | | 42.3612726836641 | | | | |
| -1.252982215 | | | | | -0.141983005 | | | | -10.30695503 | | | | | 2.08055859457321e-23 | | | | 6.31612053688757e-21 | | | | | 42.3583810875789 | | | | |
| -1.003796097 | | | | | -1.917737697 | | | | -10.30610959 | | | | | 2.08797902010702e-23 | | | | 6.33408714682393e-21 | | | | | 42.3548276205704 | | | | |
| 1.05695654383401 | | | | | 1.22214935450361 | | | | 10.3023528906912 | | | | | 2.16082239329585e-23 | | | | 6.53156973893233e-21 | | | | | 42.3212072558642 | | | | |
| -1.056837826 | | | | | -2.110720792 | | | | -10.29615508 | | | | | 2.29604188093214e-23 | | | | 6.91057801522237e-21 | | | | | 42.2617624365917 | | | | |
| -1.105015036 | | | | | -0.429802599 | | | | -10.30229023 | | | | | 2.39537549403097e-23 | | | | 7.17370727676164e-21 | | | | | 42.2203074719921 | | | | |
| -1.329339969 | | | | | -1.524389191 | | | | -10.28610983 | | | | | 2.52671442880737e-23 | | | | 7.52429147736727e-21 | | | | | 42.1676759531261 | | | | |
| -1.47626255 | | | | | 0.339723462503613 | | | | -10.41160634 | | | | | 2.58365322115643e-23 | | | | 7.68299755828656e-21 | | | | | 42.1180589803868 | | | | |
| -1.003084261 | | | | | -0.471736778 | | | | -10.27494722 | | | | | 2.77423880817377e-23 | | | | 8.19196973559267e-21 | | | | | 42.0762214677457 | | | | |
| -1.121613712 | | | | | -0.096071934 | | | | -10.27040831 | | | | | 2.8913260161086e-23 | | | | 8.48683634996184e-21 | | | | | 42.0356944941072 | | | | |
| -1.118879163 | | | | | -3.569705954 | | | | -10.26424102 | | | | | 3.07086467512734e-23 | | | | 8.94262090857006e-21 | | | | | 41.9766982714203 | | | | |
| -1.035248292 | | | | | -1.058118412 | | | | -10.25449106 | | | | | 3.3420257329877e-23 | | | | 9.6921044761274e-21 | | | | | 41.89367966 | | | | |
| -1.306022003 | | | | | -1.52854259 | | | | -10.25333068 | | | | | 3.40519311024616e-23 | | | | 9.83471081368149e-21 | | | | | 41.8751770562903 | | | | |
| 1.27223881524002 | | | | | 1.71554917988509 | | | | 10.2478944648888 | | | | | 3.54863713451245e-23 | | | | 1.01862070831169e-20 | | | | | 41.8348731075132 | | | | |
| -1.029526854 | | | | | -0.171268831 | | | | -10.24391203 | | | | | 3.67945384320964e-23 | | | | 1.04832115004474e-20 | | | | | 41.7993847540013 | | | | |
| -1.212129214 | | | | | 0.748460581700677 | | | | -10.24164957 | | | | | 3.83335849925907e-23 | | | | 1.08614892255868e-20 | | | | | 41.7589778014401 | | | | |
| 1.30684317707216 | | | | | -3.650327218 | | | | 10.2391940123062 | | | | | 3.84063613250534e-23 | | | | 1.08690002549901e-20 | | | | | 41.7573550050441 | | | | |
| -1.096083011 | | | | | -1.688232483 | | | | -10.24014484 | | | | | 3.902097603934e-23 | | | | 1.10281333555687e-20 | | | | | 41.7419110629215 | | | | |
| -1.254664859 | | | | | -0.643834128 | | | | -10.32930247 | | | | | 3.84753900414816e-23 | | | | 1.08812325411077e-20 | | | | | 41.7390408099896 | | | | |
| 1.31690641988053 | | | | | 3.23068699943612 | | | | 10.2369252220269 | | | | | 3.92062008903788e-23 | | | | 1.10624822262249e-20 | | | | | 41.7371490229888 | | | | |
| -1.222354242 | | | | | 0.149578333560438 | | | | -10.2285765 | | | | | 4.2294345474996e-23 | | | | 1.18029494748124e-20 | | | | | 41.6628239493066 | | | | |
| -1.68881305 | | | | | 1.22854218524231 | | | | -10.34532941 | | | | | 4.14424205556617e-23 | | | | 1.16036025735099e-20 | | | | | 41.6244800759114 | | | | |
| 1.44477430318476 | | | | | 1.98196489689125 | | | | 10.2199784069389 | | | | | 4.59117427466537e-23 | | | | 1.27617696532508e-20 | | | | | 41.5824392380684 | | | | |
| -1.374709817 | | | | | 0.394970956282614 | | | | -10.22863977 | | | | | 4.69091306267676e-23 | | | | 1.30124563820055e-20 | | | | | 41.5596841908837 | | | | |
| -1.408418719 | | | | | -1.80931051 | | | | -10.2035679 | | | | | 5.30637195079237e-23 | | | | 1.45673037792358e-20 | | | | | 41.4404575826307 | | | | |
| 1.09358150079791 | | | | | 3.84864121423407 | | | | 10.2034050365654 | | | | | 5.31420954550495e-23 | | | | 1.45793281656023e-20 | | | | | 41.4390107919861 | | | | |
| -1.098177351 | | | | | -0.485844662 | | | | -10.21477779 | | | | | 5.33976009838235e-23 | | | | 1.46399001344921e-20 | | | | | 41.432836422019 | | | | |
| -1.059805867 | | | | | -0.209976586 | | | | -10.20922011 | | | | | 5.47339168934447e-23 | | | | 1.49479603215407e-20 | | | | | 41.4102389318337 | | | | |
| 1.00306543352019 | | | | | 0.223556470356806 | | | | 10.205784751907 | | | | | 5.48347254175404e-23 | | | | 1.49557490846778e-20 | | | | | 41.4091375199496 | | | | |
| -1.164191612 | | | | | -2.714249739 | | | | -10.19995555 | | | | | 5.48293281713196e-23 | | | | 1.49557490846778e-20 | | | | | 41.4083720145491 | | | | |
| 1.27900811544764 | | | | | 2.45362943054788 | | | | 10.1987141324661 | | | | | 5.54494494112196e-23 | | | | 1.50944927909806e-20 | | | | | 41.3973475472707 | | | | |
| -1.270836753 | | | | | 0.323123581623197 | | | | -10.19903522 | | | | | 5.73363409916161e-23 | | | | 1.55579246498937e-20 | | | | | 41.3646258397181 | | | | |
| -1.146177696 | | | | | -0.578122308 | | | | -10.19174701 | | | | | 5.90610297556703e-23 | | | | 1.59540451102329e-20 | | | | | 41.3354944037453 | | | | |
| 1.18860348659258 | | | | | 1.22880972737019 | | | | 10.1899643114918 | | | | | 6.00220313676378e-23 | | | | 1.61928918533537e-20 | | | | | 41.3196729521115 | | | | |
| -1.006612477 | | | | | -0.913229699 | | | | -10.18731954 | | | | | 6.24679396906674e-23 | | | | 1.67243530980087e-20 | | | | | 41.2807856500077 | | | | |
| -1.323509823 | | | | | 0.33019348063589 | | | | -10.18581663 | | | | | 6.43537523630939e-23 | | | | 1.71747131385733e-20 | | | | | 41.2516595687407 | | | | |
| -1.218932206 | | | | | -0.706654441 | | | | -10.17985525 | | | | | 6.65632045585663e-23 | | | | 1.76860154166419e-20 | | | | | 41.2184768157654 | | | | |
| 1.36366556719864 | | | | | 1.58000718431008 | | | | 10.1787963093574 | | | | | 6.69358176931519e-23 | | | | 1.77738200545789e-20 | | | | | 41.212938485193 | | | | |
| -1.314408573 | | | | | -0.463567937 | | | | -10.17689754 | | | | | 6.83672389034753e-23 | | | | 1.81083000178571e-20 | | | | | 41.1920338396356 | | | | |
| 1.15583336541871 | | | | | 2.37048444281801 | | | | 10.1661702373008 | | | | | 7.4435995614064e-23 | | | | 1.94952958202375e-20 | | | | | 41.1087018488867 | | | | |
| -1.09758761 | | | | | -0.570415115 | | | | -10.1658171 | | | | | 7.61726311640627e-23 | | | | 1.98760602617267e-20 | | | | | 41.0862049371746 | | | | |
| -1.047868888 | | | | | -3.596601227 | | | | -10.15843497 | | | | | 7.98243074213719e-23 | | | | 2.0764661141499e-20 | | | | | 41.0401973556335 | | | | |
| -1.146820326 | | | | | 0.149592754744933 | | | | -10.15604764 | | | | | 8.22103832580065e-23 | | | | 2.13458450174899e-20 | | | | | 41.0114611862184 | | | | |
| 1.15026010316985 | | | | | 2.8630049243239 | | | | 10.1532876326914 | | | | | 8.36224928997284e-23 | | | | 2.16724625574852e-20 | | | | | 40.9946338188325 | | | | |
| -1.103725528 | | | | | 0.128607303524226 | | | | -10.1522127 | | | | | 8.44380236005134e-23 | | | | 2.18569560537928e-20 | | | | | 40.9851208639762 | | | | |
| -1.136943921 | | | | | -0.585396592 | | | | -10.1537964 | | | | | 8.76775785345177e-23 | | | | 2.26122351930581e-20 | | | | | 40.9488738193264 | | | | |
| -1.071535427 | | | | | -0.23199667 | | | | -10.14760499 | | | | | 8.80241237335614e-23 | | | | 2.26738743156572e-20 | | | | | 40.9443521767363 | | | | |
| -1.166509331 | | | | | 0.0992034238435257 | | | | -10.14780492 | | | | | 8.85595400141958e-23 | | | | 2.27886736307975e-20 | | | | | 40.9385425255237 | | | | |
| 1.02157109589766 | | | | | 1.0188310758889 | | | | 10.1473454528159 | | | | | 8.8577883370591e-23 | | | | 2.27886736307975e-20 | | | | | 40.938272319235 | | | | |
| -1.150124111 | | | | | 0.707563244547786 | | | | -10.14258495 | | | | | 9.21032291183554e-23 | | | | 2.35519518631516e-20 | | | | | 40.899951275775 | | | | |
| 1.14983877817277 | | | | | 2.47010152381473 | | | | 10.1423086583564 | | | | | 9.23331029384014e-23 | | | | 2.35964239491126e-20 | | | | | 40.8975079929125 | | | | |
| 1.12220553650152 | | | | | 2.69346933283447 | | | | 10.1407810210194 | | | | | 9.36143813176139e-23 | | | | 2.38949008294178e-20 | | | | | 40.884000027344 | | | | |
| -1.246431417 | | | | | -0.878127333 | | | | -10.15237861 | | | | | 9.69215595035405e-23 | | | | 2.46792958912185e-20 | | | | | 40.8498157190608 | | | | |
| -1.003124077 | | | | | -2.047919634 | | | | -10.15996465 | | | | | 9.93084598134778e-23 | | | | 2.519578715376e-20 | | | | | 40.8280989063802 | | | | |
| -1.200247535 | | | | | -1.814950375 | | | | -10.13122466 | | | | | 1.02039491856341e-22 | | | | 2.57338472075738e-20 | | | | | 40.7995340221117 | | | | |
| 1.19913214402701 | | | | | 2.4502415477452 | | | | 10.1216618712101 | | | | | 1.11222557443019e-22 | | | | 2.7816853972122e-20 | | | | | 40.7150718137658 | | | | |
| -1.003832921 | | | | | -0.554809426 | | | | -10.12160435 | | | | | 1.15747020795438e-22 | | | | 2.88287337618501e-20 | | | | | 40.6760066633358 | | | | |
| -1.3767909 | | | | | 0.118032098011505 | | | | -10.17554328 | | | | | 1.14874742593801e-22 | | | | 2.86283881261987e-20 | | | | | 40.6737918488799 | | | | |
| -1.222489704 | | | | | -0.726527158 | | | | -10.11588814 | | | | | 1.19000574870271e-22 | | | | 2.95692235742764e-20 | | | | | 40.6488971872448 | | | | |
| 1.2460583461259 | | | | | 0.953460111622475 | | | | 10.1141602919553 | | | | | 1.18996003725273e-22 | | | | 2.95692235742764e-20 | | | | | 40.6488574355153 | | | | |
| -1.537530237 | | | | | -0.702745271 | | | | -10.3641122 | | | | | 1.12712176774824e-22 | | | | 2.81226885092545e-20 | | | | | 40.5909356469828 | | | | |
| -1.182209022 | | | | | -0.694298771 | | | | -10.10289099 | | | | | 1.31697453806745e-22 | | | | 3.24753598518656e-20 | | | | | 40.5494566409532 | | | | |
| 1.19494012158394 | | | | | 1.56073579073093 | | | | 10.1003594007776 | | | | | 1.34730785995104e-22 | | | | 3.31269927914851e-20 | | | | | 40.5271383307275 | | | | |
| -1.468737049 | | | | | 0.6423418816875 | | | | -10.11602972 | | | | | 1.35408303935705e-22 | | | | 3.32736710492826e-20 | | | | | 40.523123230259 | | | | |
| -1.322785036 | | | | | -0.584028654 | | | | -10.1681363 | | | | | 1.3756868465544e-22 | | | | 3.37259228248021e-20 | | | | | 40.5026578471942 | | | | |
| 1.14815452126642 | | | | | 1.36164223466151 | | | | 10.0882880893629 | | | | | 1.52511642617658e-22 | | | | 3.70020623375075e-20 | | | | | 40.4057402015507 | | | | |
| -1.005260516 | | | | | -0.207633635 | | | | -10.08963392 | | | | | 1.57341987655092e-22 | | | | 3.80426582193364e-20 | | | | | 40.3752584037972 | | | | |
| -1.157628368 | | | | | -1.480126478 | | | | -10.07841952 | | | | | 1.64095172560735e-22 | | | | 3.95620419746626e-20 | | | | | 40.3338966421353 | | | | |
| -1.08256074 | | | | | -1.079937857 | | | | -10.07796409 | | | | | 1.6667730660811e-22 | | | | 4.01182751718177e-20 | | | | | 40.3188016714886 | | | | |
| -1.011386808 | | | | | -0.352612366 | | | | -10.08543122 | | | | | 1.66687737095417e-22 | | | | 4.01182751718177e-20 | | | | | 40.3174970670757 | | | | |
| -1.231886847 | | | | | -0.006573862 | | | | -10.07503245 | | | | | 1.69161982135479e-22 | | | | 4.06441783516053e-20 | | | | | 40.3040926580369 | | | | |
| -1.495201786 | | | | | -0.017836566 | | | | -10.11608655 | | | | | 1.71368077883414e-22 | | | | 4.11039689426047e-20 | | | | | 40.2927241365054 | | | | |
| 1.10529109979481 | | | | | 1.19660616843689 | | | | 10.0680944080838 | | | | | 1.80030252729847e-22 | | | | 4.29374189302007e-20 | | | | | 40.2430661731307 | | | | |
| -1.027199063 | | | | | -0.979007959 | | | | -10.06022265 | | | | | 1.93938616970508e-22 | | | | 4.58911877766296e-20 | | | | | 40.1702034928733 | | | | |
| -1.099588675 | | | | | -0.39608249 | | | | -10.07376633 | | | | | 1.95922546896502e-22 | | | | 4.62568087065218e-20 | | | | | 40.1608039418053 | | | | |
| -1.050782743 | | | | | -0.121360207 | | | | -10.06009512 | | | | | 1.98685539700249e-22 | | | | 4.6856673112642e-20 | | | | | 40.1469228829973 | | | | |
| -1.1986517 | | | | | -3.005744812 | | | | -10.05232894 | | | | | 2.08157626704677e-22 | | | | 4.89536120761635e-20 | | | | | 40.1008624181689 | | | | |
| -1.514907838 | | | | | 0.478478324055173 | | | | -10.07073897 | | | | | 2.12641964112236e-22 | | | | 4.9841432466485e-20 | | | | | 40.0794790373731 | | | | |
| 1.00494558337151 | | | | | 0.621930726416461 | | | | 10.0479870280049 | | | | | 2.15595818353165e-22 | | | | 5.04776728067624e-20 | | | | | 40.066384424883 | | | | |
| -1.302534552 | | | | | -0.362851187 | | | | -10.1312102 | | | | | 2.19907762111379e-22 | | | | 5.14301198277898e-20 | | | | | 40.0432292284515 | | | | |
| -1.03096041 | | | | | -0.88319164 | | | | -10.04521346 | | | | | 2.21019691627846e-22 | | | | 5.16615151711274e-20 | | | | | 40.0420345006226 | | | | |
| -1.0733857 | | | | | -0.542646211 | | | | -10.0440084 | | | | | 2.2341823055757e-22 | | | | 5.21354539453296e-20 | | | | | 40.0314565 | | | | |
| -1.387958462 | | | | | -0.560892765 | | | | -10.03703133 | | | | | 2.38720911383279e-22 | | | | 5.512675065881e-20 | | | | | 39.9666010390682 | | | | |
| 1.26416580215841 | | | | | 2.61785516472768 | | | | 10.0340367484586 | | | | | 2.45205153418646e-22 | | | | 5.64695013883344e-20 | | | | | 39.9403369787177 | | | | |
| -1.105621253 | | | | | -0.838823336 | | | | -10.03345565 | | | | | 2.45555858058051e-22 | | | | 5.65193988358834e-20 | | | | | 39.9388664106983 | | | | |
| 1.01791314828786 | | | | | 1.85080580867383 | | | | 10.0338095771772 | | | | | 2.45704120798387e-22 | | | | 5.65226713677336e-20 | | | | | 39.9382005885205 | | | | |
| -1.074679298 | | | | | -0.846490319 | | | | -10.03280302 | | | | | 2.46994245135836e-22 | | | | 5.67884751071036e-20 | | | | | 39.9331426417753 | | | | |
| -1.123543468 | | | | | -1.768872843 | | | | -10.02781447 | | | | | 2.58268480358747e-22 | | | | 5.91548452541406e-20 | | | | | 39.8894009626679 | | | | |
| -1.031482235 | | | | | -0.242448838 | | | | -10.0214805 | | | | | 2.73321384801171e-22 | | | | 6.22307928342925e-20 | | | | | 39.8338860975953 | | | | |
| -1.010241156 | | | | | -1.98910682 | | | | -10.03799698 | | | | | 2.77439458169781e-22 | | | | 6.3032271727614e-20 | | | | | 39.8195124629378 | | | | |
| -1.07612222 | | | | | 0.180171366567063 | | | | -10.01952731 | | | | | 2.79181755728274e-22 | | | | 6.33939531168236e-20 | | | | | 39.8131664424629 | | | | |
| 1.12281820434422 | | | | | 1.39579141605579 | | | | 10.0163352449111 | | | | | 2.8618711327125e-22 | | | | 6.48449865948887e-20 | | | | | 39.7888096394275 | | | | |
| 1.1488222023776 | | | | | 2.14016141438155 | | | | 10.0160768050364 | | | | | 2.868489505184e-22 | | | | 6.49600413346367e-20 | | | | | 39.786545969661 | | | | |
| 1.15814826653199 | | | | | -0.524823627 | | | | 10.0133969434309 | | | | | 2.93801897334546e-22 | | | | 6.62307317733403e-20 | | | | | 39.7630757478055 | | | | |
| 1.23812992007039 | | | | | 3.4608906655887 | | | | 10.0125460703757 | | | | | 3.0280764910753e-22 | | | | 6.80098201405466e-20 | | | | | 39.7337775670524 | | | | |
| -1.058056202 | | | | | 0.169886794986306 | | | | -10.01052852 | | | | | 3.04838009465224e-22 | | | | 6.83728954527666e-20 | | | | | 39.7270195188815 | | | | |
| -1.243870938 | | | | | -0.607073586 | | | | -10.00928952 | | | | | 3.15314851347954e-22 | | | | 7.05351795055128e-20 | | | | | 39.6943291839774 | | | | |
| 1.16425680688118 | | | | | 4.39725936716457 | | | | 9.9982248529438 | | | | | 3.36436135243739e-22 | | | | 7.482332550012e-20 | | | | | 39.6302892278323 | | | | |
| 1.33196878468647 | | | | | -3.720696447 | | | | 9.99797979933357 | | | | | 3.37172834737879e-22 | | | | 7.49476379672755e-20 | | | | | 39.6281457769749 | | | | |
| -1.197680158 | | | | | 0.869691011903283 | | | | -10.00215657 | | | | | 3.4252120245529e-22 | | | | 7.59363382961736e-20 | | | | | 39.6123753983485 | | | | |
| -1.17507701 | | | | | -0.849515195 | | | | -10.04649325 | | | | | 3.42715620010042e-22 | | | | 7.59395141826771e-20 | | | | | 39.6110329188107 | | | | |
| -1.02554634 | | | | | -0.857581209 | | | | -9.995019009 | | | | | 3.4620122306517e-22 | | | | 7.65911173818941e-20 | | | | | 39.6022513249788 | | | | |
| -1.083966034 | | | | | -2.3526708 | | | | -9.994602412 | | | | | 3.47490650367361e-22 | | | | 7.68360684532801e-20 | | | | | 39.5986083241461 | | | | |
| 1.01179837488273 | | | | | 1.626974521 | | | | 9.9895948167703 | | | | | 3.8901335251596e-22 | | | | 8.54350314351067e-20 | | | | | 39.4890150582521 | | | | |
| -1.737514651 | | | | | -0.241794352 | | | | -10.12488883 | | | | | 3.87499930697618e-22 | | | | 8.51913950872078e-20 | | | | | 39.4777680599116 | | | | |
| 1.60952688288992 | | | | | -0.180935528 | | | | 9.99358088605031 | | | | | 4.08208258372686e-22 | | | | 8.91399152294203e-20 | | | | | 39.4392182139066 | | | | |
| -1.128213468 | | | | | -1.433263607 | | | | -9.973008271 | | | | | 4.37277669252405e-22 | | | | 9.47517342207922e-20 | | | | | 39.3733520127652 | | | | |
| -1.094753209 | | | | | 0.349569447825674 | | | | -9.969665101 | | | | | 4.50475488718405e-22 | | | | 9.75112932894712e-20 | | | | | 39.3447316840683 | | | | |
| -1.002637641 | | | | | -1.242914077 | | | | -9.964642008 | | | | | 4.53860746713419e-22 | | | | 9.81936690952527e-20 | | | | | 39.3369198259044 | | | | |
| 1.24166864796586 | | | | | 0.479326718198783 | | | | 9.96331086532045 | | | | | 4.59271547568916e-22 | | | | 9.93133504940434e-20 | | | | | 39.3253069586033 | | | | |
| -1.007711946 | | | | | -0.08669736 | | | | -9.959169132 | | | | | 4.78271122186988e-22 | | | | 1.03051908069794e-19 | | | | | 39.2856580178136 | | | | |
| 1.09096569676164 | | | | | 1.47236631361534 | | | | 9.95298178285797 | | | | | 5.03488793618359e-22 | | | | 1.07988870602774e-19 | | | | | 39.2352366844476 | | | | |
| -1.213051664 | | | | | -1.467450839 | | | | -9.94972279 | | | | | 5.18297181168329e-22 | | | | 1.10658416396582e-19 | | | | | 39.2068329328346 | | | | |
| -1.121945027 | | | | | -0.395395721 | | | | -10.05298972 | | | | | 5.25362302927284e-22 | | | | 1.12110082123152e-19 | | | | | 39.1867795321735 | | | | |
| 1.0189017526882 | | | | | 2.48724555729859 | | | | 9.94604742322857 | | | | | 5.35516142443359e-22 | | | | 1.14046005951561e-19 | | | | | 39.174808836524 | | | | |
| -1.201017114 | | | | | -0.33164381 | | | | -9.944538976 | | | | | 5.44730931119752e-22 | | | | 1.15832925731349e-19 | | | | | 39.1581636455084 | | | | |
| -1.044605046 | | | | | -0.510713134 | | | | -9.9464667 | | | | | 5.45418168970921e-22 | | | | 1.15920604490911e-19 | | | | | 39.157193324835 | | | | |
| -1.02859636 | | | | | -0.804954557 | | | | -9.944990322 | | | | | 5.46540283708834e-22 | | | | 1.16100575028466e-19 | | | | | 39.155058959022 | | | | |
| -1.254358236 | | | | | -0.023309757 | | | | -9.948598697 | | | | | 5.57754115102871e-22 | | | | 1.18066354274813e-19 | | | | | 39.1356479115309 | | | | |
| -1.058996346 | | | | | -0.870732441 | | | | -9.951749 | | | | | 5.79486615702649e-22 | | | | 1.22543691696758e-19 | | | | | 39.098221280949 | | | | |
| -1.011037815 | | | | | -0.242744758 | | | | -9.943406703 | | | | | 5.9302148088471e-22 | | | | 1.25029683922328e-19 | | | | | 39.075690693824 | | | | |
| -1.283956794 | | | | | 0.194909931739307 | | | | -9.954479585 | | | | | 6.13781926490486e-22 | | | | 1.29084002465458e-19 | | | | | 39.0409680860336 | | | | |
| -1.099479784 | | | | | -0.902082304 | | | | -10.04496813 | | | | | 6.20915885584732e-22 | | | | 1.30389243762208e-19 | | | | | 39.0228691631864 | | | | |
| 1.54131599423676 | | | | | 0.49848002565318 | | | | 9.93343442319998 | | | | | 6.38001673464735e-22 | | | | 1.33644394262233e-19 | | | | | 39.0038064084184 | | | | |
| -1.057876144 | | | | | -2.031422745 | | | | -9.932279092 | | | | | 6.6457364236371e-22 | | | | 1.38659459562348e-19 | | | | | 38.9639839216599 | | | | |
| 1.00268912500554 | | | | | 3.37450308001184 | | | | 9.91301670052599 | | | | | 7.180511681705e-22 | | | | 1.49153022700717e-19 | | | | | 38.8874144662043 | | | | |
| 1.107801921 | | | | | 3.26463775411455 | | | | 9.90949131233366 | | | | | 7.40853289199661e-22 | | | | 1.5343595602005e-19 | | | | | 38.8567841031661 | | | | |
| -1.146942643 | | | | | -0.249056641 | | | | -9.909816645 | | | | | 7.60514256289201e-22 | | | | 1.57276138523525e-19 | | | | | 38.8314240442372 | | | | |
| -1.003686777 | | | | | -1.711604111 | | | | -9.904674265 | | | | | 7.73174533863616e-22 | | | | 1.59425186158568e-19 | | | | | 38.8149446943474 | | | | |
| -1.126490119 | | | | | -1.71838349 | | | | -9.903652465 | | | | | 7.80208240816429e-22 | | | | 1.60690767840399e-19 | | | | | 38.8060716643709 | | | | |
| 1.06871878447121 | | | | | 2.18589501616588 | | | | 9.90361670375972 | | | | | 7.80455551822839e-22 | | | | 1.60690767840399e-19 | | | | | 38.8057611391342 | | | | |
| -1.076399023 | | | | | -0.923994981 | | | | -9.903063313 | | | | | 7.842925452441e-22 | | | | 1.61401970499307e-19 | | | | | 38.8009559544367 | | | | |
| 1.16525179457857 | | | | | 1.33227247244521 | | | | 9.90278243438912 | | | | | 7.86247198162819e-22 | | | | 1.61725295633813e-19 | | | | | 38.7985171148689 | | | | |
| 1.0708133969403 | | | | | 2.72062203365119 | | | | 9.9008848196363 | | | | | 7.99579946339529e-22 | | | | 1.63908058324253e-19 | | | | | 38.7820417306805 | | | | |
| -1.083034588 | | | | | -1.705283346 | | | | -9.899310569 | | | | | 8.10810647684391e-22 | | | | 1.66048822636754e-19 | | | | | 38.7683756836869 | | | | |
| -1.426400457 | | | | | 0.47122265129448 | | | | -9.896091454 | | | | | 8.34264941746769e-22 | | | | 1.70355689097511e-19 | | | | | 38.7404358128436 | | | | |
| -1.278838132 | | | | | 0.0930659843976087 | | | | -9.896716369 | | | | | 8.50908360029983e-22 | | | | 1.73586128773025e-19 | | | | | 38.7214423465928 | | | | |
| -1.023212962 | | | | | -0.457082052 | | | | -9.891793506 | | | | | 8.66631153690785e-22 | | | | 1.76622696267179e-19 | | | | | 38.7031432514063 | | | | |
| -1.47238482 | | | | | 0.414726859851745 | | | | -9.89422325 | | | | | 8.69886452559991e-22 | | | | 1.77200492971484e-19 | | | | | 38.6999182172824 | | | | |
| -1.233206694 | | | | | 0.0928902392276102 | | | | -9.898416188 | | | | | 8.89588740460006e-22 | | | | 1.80690213964244e-19 | | | | | 38.6786543398446 | | | | |
| -1.040635346 | | | | | -0.379445384 | | | | -9.88735946 | | | | | 9.07800643422945e-22 | | | | 1.83946322591136e-19 | | | | | 38.6578180656088 | | | | |
| -1.306838138 | | | | | 0.478146814647173 | | | | -9.896854118 | | | | | 9.1578892269792e-22 | | | | 1.85475847758901e-19 | | | | | 38.6505109195794 | | | | |
| 1.41989721346425 | | | | | -2.120131508 | | | | 10.0109329390901 | | | | | 8.89176050271814e-22 | | | | 1.80690213964244e-19 | | | | | 38.6481300854144 | | | | |
| -1.0581058 | | | | | -0.319600415 | | | | -9.884546321 | | | | | 9.24049812534672e-22 | | | | 1.86969330351005e-19 | | | | | 38.640288923449 | | | | |
| 1.32071971803074 | | | | | 1.24062447408133 | | | | 9.88441335746685 | | | | | 9.25137757522249e-22 | | | | 1.87099682596838e-19 | | | | | 38.6391360682815 | | | | |
| -1.067738572 | | | | | -0.955210704 | | | | -9.908720987 | | | | | 9.41455133990949e-22 | | | | 1.90067429408149e-19 | | | | | 38.6254113224628 | | | | |
| 1.04990872041663 | | | | | 2.16956968270283 | | | | 9.88302771276459 | | | | | 9.39899158636807e-22 | | | | 1.89902864505214e-19 | | | | | 38.6237001454286 | | | | |
| -1.137072306 | | | | | -0.43976759 | | | | -9.881442659 | | | | | 9.66950082591476e-22 | | | | 1.94808333170735e-19 | | | | | 38.5959691621522 | | | | |
| -1.058340041 | | | | | -0.334025869 | | | | -9.879435983 | | | | | 9.77206853582056e-22 | | | | 1.96592945586806e-19 | | | | | 38.5856544915135 | | | | |
| 1.12047690840106 | | | | | 0.378308023955458 | | | | 9.87830525217169 | | | | | 9.79993534826328e-22 | | | | 1.97059548798387e-19 | | | | | 38.5827727516765 | | | | |
| -1.278881439 | | | | | 0.141757385 | | | | -9.890256632 | | | | | 9.85670639383181e-22 | | | | 1.97844970960578e-19 | | | | | 38.5787806178894 | | | | |
| -1.00205643 | | | | | -1.143820959 | | | | -9.88628182 | | | | | 1.02079715731582e-21 | | | | 2.04484340772143e-19 | | | | | 38.5448940180033 | | | | |
| -1.240822324 | | | | | -0.226006878 | | | | -9.871519952 | | | | | 1.0405869930462e-21 | | | | 2.08151953205784e-19 | | | | | 38.5239940489706 | | | | |
| -1.483921482 | | | | | 0.499840872196807 | | | | -9.959535593 | | | | | 1.04703907026877e-21 | | | | 2.09210810453456e-19 | | | | | 38.4980883933217 | | | | |
| -1.148669071 | | | | | -1.727530685 | | | | -9.867736609 | | | | | 1.07215955603096e-21 | | | | 2.13960018926444e-19 | | | | | 38.494636087755 | | | | |
| -1.083806389 | | | | | -0.599913597 | | | | -9.867302622 | | | | | 1.08010483271885e-21 | | | | 2.15250157828866e-19 | | | | | 38.4874764664652 | | | | |
| -1.381368152 | | | | | -1.55636939 | | | | -9.866213585 | | | | | 1.08668850619402e-21 | | | | 2.16347470447041e-19 | | | | | 38.4814488300398 | | | | |
| -1.10650315 | | | | | -0.97390407 | | | | -9.869429722 | | | | | 1.0908708360913e-21 | | | | 2.16872939865449e-19 | | | | | 38.4783554125404 | | | | |
| -1.109095024 | | | | | -0.733160545 | | | | -9.865815504 | | | | | 1.090518085105e-21 | | | | 2.16872939865449e-19 | | | | | 38.4780022616687 | | | | |
| -1.097581104 | | | | | -1.068157505 | | | | -9.863238382 | | | | | 1.11563595990187e-21 | | | | 2.21483152171292e-19 | | | | | 38.455692250528 | | | | |
| 1.05059766908411 | | | | | 1.96310291324865 | | | | 9.8602553089538 | | | | | 1.14542729812981e-21 | | | | 2.26970079324434e-19 | | | | | 38.4298735529798 | | | | |
| -1.219508589 | | | | | 1.50959673126168 | | | | -9.859169936 | | | | | 1.156461248413e-21 | | | | 2.29048856091269e-19 | | | | | 38.4204810694645 | | | | |
| -1.191111939 | | | | | 1.89657751478454 | | | | -9.852681591 | | | | | 1.22898309812337e-21 | | | | 2.41935248826182e-19 | | | | | 38.3609282061178 | | | | |
| 1.16762824601889 | | | | | 3.30070448019509 | | | | 9.85184030456594 | | | | | 1.23378341262333e-21 | | | | 2.42643357317828e-19 | | | | | 38.357073595023 | | | | |
| -1.010716738 | | | | | -0.997015592 | | | | -9.851792291 | | | | | 1.23430645160135e-21 | | | | 2.42643357317828e-19 | | | | | 38.3566583565419 | | | | |
| -1.233365324 | | | | | 0.175066735387412 | | | | -9.848436117 | | | | | 1.27141702641628e-21 | | | | 2.48894344256709e-19 | | | | | 38.3276368785363 | | | | |
| 1.12803284563221 | | | | | 1.8293887669034 | | | | 9.84223175332037 | | | | | 1.34295706773963e-21 | | | | 2.61563374944004e-19 | | | | | 38.2740066622579 | | | | |
| -1.576632588 | | | | | 0.0617795356468369 | | | | -9.839862047 | | | | | 1.38588077698895e-21 | | | | 2.686824584979e-19 | | | | | 38.2434080572358 | | | | |
| -1.015221145 | | | | | -0.533013524 | | | | -9.838435218 | | | | | 1.38868663053795e-21 | | | | 2.6910270749032e-19 | | | | | 38.2412024593813 | | | | |
| 1.08671466123187 | | | | | 1.67158932158792 | | | | 9.83626921838711 | | | | | 1.41546433016756e-21 | | | | 2.73383358135518e-19 | | | | | 38.2224913835614 | | | | |
| -1.129837837 | | | | | -3.341403952 | | | | -9.83456813 | | | | | 1.43685275374778e-21 | | | | 2.77162717599646e-19 | | | | | 38.2077986925897 | | | | |
| -1.180709057 | | | | | -1.881252633 | | | | -9.833615498 | | | | | 1.44897018141552e-21 | | | | 2.79244632722798e-19 | | | | | 38.1995714454657 | | | | |
| -1.043601661 | | | | | -0.761100176 | | | | -9.83193483 | | | | | 1.48616214898595e-21 | | | | 2.85368849436659e-19 | | | | | 38.1748939976412 | | | | |
| -1.137639672 | | | | | 0.180521582526831 | | | | -9.831018708 | | | | | 1.48771611897947e-21 | | | | 2.8553721251255e-19 | | | | | 38.1737672821108 | | | | |
| -1.051643022 | | | | | -0.238981123 | | | | -9.841868877 | | | | | 1.52003374273947e-21 | | | | 2.90865279343825e-19 | | | | | 38.1547366543141 | | | | |
| -1.031829014 | | | | | -0.12330234 | | | | -9.831217898 | | | | | 1.52231091055598e-21 | | | | 2.910450086499e-19 | | | | | 38.1518011865885 | | | | |
| -1.053764289 | | | | | -1.570557489 | | | | -9.848979725 | | | | | 1.55694179279189e-21 | | | | 2.95994430011973e-19 | | | | | 38.1309370964514 | | | | |
| 1.00003263928421 | | | | | -0.108443609 | | | | 9.82457335132312 | | | | | 1.56914945271245e-21 | | | | 2.98046508885251e-19 | | | | | 38.1215111326259 | | | | |
| -1.274319404 | | | | | -0.158200864 | | | | -9.83955769 | | | | | 1.63762799836784e-21 | | | | 3.09658564157744e-19 | | | | | 38.0815583742195 | | | | |
| -1.246014838 | | | | | -0.551461649 | | | | -9.919719296 | | | | | 1.73375078039344e-21 | | | | 3.26370844450224e-19 | | | | | 38.0177047583146 | | | | |
| -1.149764109 | | | | | -0.045055117 | | | | -9.809984039 | | | | | 1.82844895740774e-21 | | | | 3.41717285641259e-19 | | | | | 37.9721656794339 | | | | |
| -1.919318538 | | | | | 3.77399417592259 | | | | -9.805542852 | | | | | 1.85531052616717e-21 | | | | 3.46002594204192e-19 | | | | | 37.9574033012402 | | | | |
| -1.146519655 | | | | | 0.0469079541973766 | | | | -9.863434732 | | | | | 1.86645251224076e-21 | | | | 3.47626780404842e-19 | | | | | 37.9505799293767 | | | | |
| -1.19318745 | | | | | -0.143705221 | | | | -9.805506214 | | | | | 1.90183954365595e-21 | | | | 3.53748866507898e-19 | | | | | 37.9336526352351 | | | | |
| 1.31589939369347 | | | | | 2.40415641360373 | | | | 9.7925191877555 | | | | | 2.08038489236494e-21 | | | | 3.8403675671394e-19 | | | | | 37.8452367368988 | | | | |
| -1.547594923 | | | | | -0.361700065 | | | | -10.15936377 | | | | | 1.94334876561173e-21 | | | | 3.60578542901442e-19 | | | | | 37.8055729819126 | | | | |
| -1.300665141 | | | | | -0.075173082 | | | | -9.802098125 | | | | | 2.17007170576149e-21 | | | | 3.99412543067851e-19 | | | | | 37.8046942812356 | | | | |
| -1.148112869 | | | | | -1.026368405 | | | | -9.798602423 | | | | | 2.20414575407495e-21 | | | | 4.04448276814963e-19 | | | | | 37.7905915846852 | | | | |
| -1.145234012 | | | | | 0.429299845179221 | | | | -9.863438099 | | | | | 2.17627198205988e-21 | | | | 4.00378973243975e-19 | | | | | 37.7840945081642 | | | | |
| -1.143529311 | | | | | -1.013485788 | | | | -9.785896847 | | | | | 2.22025607393866e-21 | | | | 4.07227219964209e-19 | | | | | 37.7816465952488 | | | | |
| 1.05161228303003 | | | | | 0.77774441273715 | | | | 9.78312085509099 | | | | | 2.25942237675395e-21 | | | | 4.13691113159288e-19 | | | | | 37.7643650893014 | | | | |
| -1.582974744 | | | | | 0.50205015526352 | | | | -9.918867258 | | | | | 2.18638918601681e-21 | | | | 4.01714478460875e-19 | | | | | 37.7420153098303 | | | | |
| -1.016423241 | | | | | -0.634321217 | | | | -9.833343456 | | | | | 2.31830007999511e-21 | | | | 4.2355181747467e-19 | | | | | 37.7404396251851 | | | | |
| -1.16007208 | | | | | 0.367273500715397 | | | | -9.792875708 | | | | | 2.39722740855226e-21 | | | | 4.37025024368453e-19 | | | | | 37.7073128249642 | | | | |
| -1.185779875 | | | | | -0.965140324 | | | | -9.782301686 | | | | | 2.46829154115297e-21 | | | | 4.4823621626097e-19 | | | | | 37.6794886424637 | | | | |
| -1.257435173 | | | | | -2.308013464 | | | | -9.773218698 | | | | | 2.49011156905427e-21 | | | | 4.51615202289512e-19 | | | | | 37.6693610857106 | | | | |
| 1.20357106082432 | | | | | 1.35539895869989 | | | | 9.77076478112137 | | | | | 2.51822765632788e-21 | | | | 4.56125883094405e-19 | | | | | 37.6581339064187 | | | | |
| 1.49029721157365 | | | | | -2.036125835 | | | | 9.85344987550357 | | | | | 2.48928483873967e-21 | | | | 4.51615202289512e-19 | | | | | 37.6533352170227 | | | | |
| -1.337256416 | | | | | 0.168376914404793 | | | | -9.765801973 | | | | | 2.65742118067208e-21 | | | | 4.80512345306173e-19 | | | | | 37.6056620926079 | | | | |
| -1.351528344 | | | | | 0.833804887175378 | | | | -9.769659788 | | | | | 2.6695485306979e-21 | | | | 4.82498297873717e-19 | | | | | 37.6016877032857 | | | | |
| -1.099145475 | | | | | 0.112224172863414 | | | | -9.759002459 | | | | | 2.8013488268614e-21 | | | | 5.04589816242053e-19 | | | | | 37.5538430724948 | | | | |
| -1.326420099 | | | | | 0.309726582167973 | | | | -9.767256352 | | | | | 2.87754140253388e-21 | | | | 5.17429800940922e-19 | | | | | 37.5295246745941 | | | | |
| -1.008908201 | | | | | -1.458906546 | | | | -9.755467111 | | | | | 2.87966870231683e-21 | | | | 5.17591603455216e-19 | | | | | 37.5267565499063 | | | | |
| 1.20293777814587 | | | | | 1.39494148361354 | | | | 9.75644139851176 | | | | | 2.9551639414476e-21 | | | | 5.29580951632049e-19 | | | | | 37.5021813775425 | | | | |
| 1.83967300505658 | | | | | -4.334680211 | | | | 9.75229799973113 | | | | | 2.96074231803484e-21 | | | | 5.30130026855946e-19 | | | | | 37.4995599779082 | | | | |
| 1.12189402114914 | | | | | 1.04646854654997 | | | | 9.74580177951935 | | | | | 3.13406972021929e-21 | | | | 5.58084112721651e-19 | | | | | 37.4438323661382 | | | | |
| -1.038719643 | | | | | -0.321834632 | | | | -9.7522384 | | | | | 3.26856724804853e-21 | | | | 5.80563079816607e-19 | | | | | 37.404765453105 | | | | |
| -1.119236932 | | | | | 0.394274387984817 | | | | -9.740218688 | | | | | 3.30219730427294e-21 | | | | 5.85549847473832e-19 | | | | | 37.3927495376008 | | | | |
| -1.067453338 | | | | | 0.108297687890261 | | | | -9.757452866 | | | | | 3.32732503210223e-21 | | | | 5.89262136197626e-19 | | | | | 37.3881735638307 | | | | |
| -1.134568096 | | | | | -0.05796314 | | | | -9.741531013 | | | | | 3.33205044785211e-21 | | | | 5.89851264628799e-19 | | | | | 37.3843853635634 | | | | |
| -1.160595877 | | | | | -0.117182831 | | | | -9.736873815 | | | | | 3.43507956404721e-21 | | | | 6.06561976453847e-19 | | | | | 37.3543373272954 | | | | |
| -1.353245557 | | | | | 0.0488946393653051 | | | | -9.808656966 | | | | | 3.42659500971483e-21 | | | | 6.05317267593822e-19 | | | | | 37.3428592503324 | | | | |
| -1.062339572 | | | | | -0.051162687 | | | | -9.730933362 | | | | | 3.56954538752925e-21 | | | | 6.27753143938347e-19 | | | | | 37.316392601266 | | | | |
| 1.05879565677277 | | | | | 0.820212692979594 | | | | 9.73027568325472 | | | | | 3.59013486811792e-21 | | | | 6.30771737433034e-19 | | | | | 37.3107590138541 | | | | |
| -1.139937067 | | | | | -0.524882926 | | | | -9.729614827 | | | | | 3.61094236712895e-21 | | | | 6.33899278912266e-19 | | | | | 37.3050985087609 | | | | |
| 1.06871227010926 | | | | | 2.95612070603295 | | | | 9.71229730991752 | | | | | 4.20090397603842e-21 | | | | 7.28369728444128e-19 | | | | | 37.156873216634 | | | | |
| -1.105397167 | | | | | 0.785829704278474 | | | | -9.721875188 | | | | | 4.21290884681021e-21 | | | | 7.2985097511687e-19 | | | | | 37.1558265681376 | | | | |
| 1.07824189454168 | | | | | 1.79620721177625 | | | | 9.70845341695722 | | | | | 4.34428950492988e-21 | | | | 7.51685086394658e-19 | | | | | 37.1240001329866 | | | | |
| -1.236083598 | | | | | 0.114941320275394 | | | | -9.69999162 | | | | | 4.80471601120794e-21 | | | | 8.23244453655445e-19 | | | | | 37.0260043337983 | | | | |
| 1.32886036752342 | | | | | 0.450249307549049 | | | | 9.6957305866531 | | | | | 4.85436387118448e-21 | | | | 8.31075766773188e-19 | | | | | 37.0152662968984 | | | | |
| 1.03207857318818 | | | | | 2.18135089827731 | | | | 9.69468943272468 | | | | | 4.89864181103982e-21 | | | | 8.37635966123747e-19 | | | | | 37.0063731342236 | | | | |
| -1.288686311 | | | | | 0.0469778874377267 | | | | -9.687691246 | | | | | 5.20682376667605e-21 | | | | 8.86379240086512e-19 | | | | | 36.946616418448 | | | | |
| -1.289842454 | | | | | -1.362654358 | | | | -9.685294403 | | | | | 5.334379501961e-21 | | | | 9.04442221387976e-19 | | | | | 36.922990161928 | | | | |
| -1.226640154 | | | | | 0.133426134264809 | | | | -9.682161201 | | | | | 5.4638775600918e-21 | | | | 9.2413683604194e-19 | | | | | 36.8994197571915 | | | | |
| -1.142653212 | | | | | -0.639620511 | | | | -9.681769107 | | | | | 5.5007482927315e-21 | | | | 9.28914910931554e-19 | | | | | 36.8929110088995 | | | | |
| 1.05350593582068 | | | | | 0.857454776480459 | | | | 9.68083021735341 | | | | | 5.52760012471082e-21 | | | | 9.32329257834724e-19 | | | | | 36.888063492532 | | | | |
| 1.05845926879777 | | | | | 2.30878515447621 | | | | 9.68042123159812 | | | | | 5.54732836480591e-21 | | | | 9.34908853552242e-19 | | | | | 36.8845741736614 | | | | |
| -1.021448312 | | | | | -0.06743582 | | | | -9.680732047 | | | | | 5.55066166893018e-21 | | | | 9.35096886111782e-19 | | | | | 36.884064049179 | | | | |
| 1.09614020514438 | | | | | 2.97562946765049 | | | | 9.68023647638956 | | | | | 5.55626326355349e-21 | | | | 9.35666745344489e-19 | | | | | 36.8829979465304 | | | | |
| -1.151556806 | | | | | 0.216054844064758 | | | | -9.686527565 | | | | | 5.70860389850308e-21 | | | | 9.57116105718406e-19 | | | | | 36.8579045341014 | | | | |
| -1.222192215 | | | | | 0.112095944178077 | | | | -9.678771092 | | | | | 5.76036158382018e-21 | | | | 9.65026487504749e-19 | | | | | 36.8482176461772 | | | | |
| -1.213227658 | | | | | -0.133813026 | | | | -9.692854235 | | | | | 5.78670804544655e-21 | | | | 9.6867057623003e-19 | | | | | 36.8463727448236 | | | | |
| -1.161386089 | | | | | 0.661100080828419 | | | | -9.673959178 | | | | | 5.90736537254912e-21 | | | | 9.85737537254764e-19 | | | | | 36.8232054261068 | | | | |
| -1.0510259 | | | | | -0.471447816 | | | | -9.670629084 | | | | | 6.24575977303342e-21 | | | | 1.0364618352991e-18 | | | | | 36.7692806476917 | | | | |
| 1.09824472135944 | | | | | 0.831367630016452 | | | | 9.66610904854686 | | | | | 6.28344370570219e-21 | | | | 1.04189528406742e-18 | | | | | 36.7625401021279 | | | | |
| -1.109005845 | | | | | -0.992661134 | | | | -9.671218405 | | | | | 6.38556251320902e-21 | | | | 1.05675044935041e-18 | | | | | 36.7482081690263 | | | | |
| 1.15061049251679 | | | | | 1.03267577922613 | | | | 9.66176052977564 | | | | | 6.52570217287775e-21 | | | | 1.07740518216028e-18 | | | | | 36.7254899419299 | | | | |
| 1.02380061108714 | | | | | 0.881796868281175 | | | | 9.65898535980181 | | | | | 6.70709365330344e-21 | | | | 1.10432650557925e-18 | | | | | 36.6987888257038 | | | | |
| 1.31310414138336 | | | | | 0.945723632992927 | | | | 9.65644192212392 | | | | | 6.94811505472415e-21 | | | | 1.14089239685574e-18 | | | | | 36.6644576558229 | | | | |
| -1.438195259 | | | | | 0.441536820708746 | | | | -9.655452181 | | | | | 6.98496757980761e-21 | | | | 1.14515990644536e-18 | | | | | 36.6592688629173 | | | | |
| -1.058138223 | | | | | -1.362497149 | | | | -9.668555732 | | | | | 7.11494223470219e-21 | | | | 1.16465748917192e-18 | | | | | 36.6439592568089 | | | | |
| 1.05075533362292 | | | | | 0.806039378118556 | | | | 9.65184995406632 | | | | | 7.13627078666706e-21 | | | | 1.16769549965615e-18 | | | | | 36.6380485779341 | | | | |
| -1.019027458 | | | | | -0.646075775 | | | | -9.651390773 | | | | | 7.1414008452297e-21 | | | | 1.16808165027463e-18 | | | | | 36.6371902008427 | | | | |
| 1.03237457251462 | | | | | 1.09200114978937 | | | | 9.64967803888285 | | | | | 7.24849105411803e-21 | | | | 1.18376112424088e-18 | | | | | 36.6226131710966 | | | | |
| -1.007489386 | | | | | -1.853643025 | | | | -9.64920989 | | | | | 7.27803837212078e-21 | | | | 1.18812638032217e-18 | | | | | 36.6186291217367 | | | | |
| -1.037777877 | | | | | -1.021088941 | | | | -9.674354921 | | | | | 7.52582909055472e-21 | | | | 1.22620415479683e-18 | | | | | 36.5901838673445 | | | | |
| -1.418855802 | | | | | -1.651800057 | | | | -9.644973764 | | | | | 7.55088692420825e-21 | | | | 1.22981169923943e-18 | | | | | 36.5825856165164 | | | | |
| -1.170966679 | | | | | -0.643599586 | | | | -9.644269307 | | | | | 7.59723366297895e-21 | | | | 1.23545141483546e-18 | | | | | 36.5765928711694 | | | | |
| -1.058453563 | | | | | -0.394082861 | | | | -9.648204763 | | | | | 7.71726466623887e-21 | | | | 1.25448688967346e-18 | | | | | 36.5625955717879 | | | | |
| -1.102552504 | | | | | -0.006285275 | | | | -9.642349355 | | | | | 7.72498467843036e-21 | | | | 1.25525791497254e-18 | | | | | 36.5602617588712 | | | | |
| -1.043858802 | | | | | -0.456683063 | | | | -9.644314081 | | | | | 7.98198052619281e-21 | | | | 1.29104784368229e-18 | | | | | 36.529481942198 | | | | |
| -1.176331677 | | | | | -0.68890813 | | | | -9.664526899 | | | | | 8.032185425183e-21 | | | | 1.29867010285158e-18 | | | | | 36.526749145393 | | | | |
| -1.087173316 | | | | | -0.624949704 | | | | -9.655685485 | | | | | 8.03965279269998e-21 | | | | 1.29937922311146e-18 | | | | | 36.5241944200302 | | | | |
| -1.043774588 | | | | | -0.940157322 | | | | -9.658339427 | | | | | 8.06364389457224e-21 | | | | 1.30275736437712e-18 | | | | | 36.5220996738034 | | | | |
| -1.10390495 | | | | | -0.366148188 | | | | -9.634871291 | | | | | 8.24317892584154e-21 | | | | 1.33074320737351e-18 | | | | | 36.4966775395374 | | | | |
| 1.1273312883113 | | | | | 4.00139427650382 | | | | 9.63397426762423 | | | | | 8.33465124196921e-21 | | | | 1.34396649682645e-18 | | | | | 36.4859493678539 | | | | |
| -1.070913274 | | | | | -0.538324621 | | | | -9.629374264 | | | | | 8.64592383117376e-21 | | | | 1.38673514716281e-18 | | | | | 36.4499621693438 | | | | |
| 1.10539969748329 | | | | | 2.22522824846625 | | | | 9.62401967641132 | | | | | 9.05696318624303e-21 | | | | 1.44880488116203e-18 | | | | | 36.404477298775 | | | | |
| -1.006252492 | | | | | -0.745812469 | | | | -9.627699748 | | | | | 9.1252980780239e-21 | | | | 1.45862943159982e-18 | | | | | 36.3983294900076 | | | | |
| 1.01981805622244 | | | | | 1.05654917450305 | | | | 9.62245251037387 | | | | | 9.18088683063586e-21 | | | | 1.46640323858872e-18 | | | | | 36.3911686484853 | | | | |
| -1.355057336 | | | | | -0.276755124 | | | | -9.623676109 | | | | | 9.20282110977764e-21 | | | | 1.46935008608858e-18 | | | | | 36.3891506424069 | | | | |
| 1.01735255189718 | | | | | 1.15213306514791 | | | | 9.62170610217978 | | | | | 9.2404982606142e-21 | | | | 1.4748073056598e-18 | | | | | 36.3848306135303 | | | | |
| 1.19044650605075 | | | | | 2.50403621775807 | | | | 9.62008311623546 | | | | | 9.3714438454117e-21 | | | | 1.49401010445926e-18 | | | | | 36.3710505497344 | | | | |
| 1.18609305282879 | | | | | 1.95382235228362 | | | | 9.62057246107793 | | | | | 9.48461803483152e-21 | | | | 1.50919957990468e-18 | | | | | 36.3599204131157 | | | | |
| 1.2446160348578 | | | | | 1.23846980905454 | | | | 9.61766870382348 | | | | | 9.6317468734037e-21 | | | | 1.53100476096467e-18 | | | | | 36.3443797977946 | | | | |
| -1.453559392 | | | | | 0.512878263934652 | | | | -9.773881858 | | | | | 9.32999979329804e-21 | | | | 1.48852857088157e-18 | | | | | 36.3140060568008 | | | | |
| -1.627504837 | | | | | 0.946450485331288 | | | | -9.612704721 | | | | | 1.01534478856687e-20 | | | | 1.60592812076141e-18 | | | | | 36.2929645767056 | | | | |
| -1.150765796 | | | | | -0.326805498 | | | | -9.611149549 | | | | | 1.01585031978492e-20 | | | | 1.60612525063258e-18 | | | | | 36.2921566622152 | | | | |
| -1.014426594 | | | | | -0.257138313 | | | | -9.598096762 | | | | | 1.17109648395238e-20 | | | | 1.83302247360133e-18 | | | | | 36.1538018820126 | | | | |
| -1.000256475 | | | | | -0.291895142 | | | | -9.596023336 | | | | | 1.17299947058233e-20 | | | | 1.83531980245065e-18 | | | | | 36.1518168979207 | | | | |
| -1.3216626 | | | | | 0.293505780231739 | | | | -9.612640739 | | | | | 1.23119510042682e-20 | | | | 1.917127171333e-18 | | | | | 36.1087385798855 | | | | |
| -1.173964287 | | | | | 0.321557761666481 | | | | -9.589102347 | | | | | 1.22548824677421e-20 | | | | 1.91035722372377e-18 | | | | | 36.108354760311 | | | | |
| -1.174913452 | | | | | -0.003330191 | | | | -9.587743338 | | | | | 1.23997551726122e-20 | | | | 1.92890284248634e-18 | | | | | 36.0968464551098 | | | | |
| -1.313375878 | | | | | 0.271518010191355 | | | | -9.590465344 | | | | | 1.24277099396638e-20 | | | | 1.93158586445192e-18 | | | | | 36.0955577216545 | | | | |
| -1.037860863 | | | | | -1.573145993 | | | | -9.594879161 | | | | | 1.2531510829236e-20 | | | | 1.94556780978054e-18 | | | | | 36.0885697886822 | | | | |
| 1.03864108397153 | | | | | 2.19214481571681 | | | | 9.58366389590899 | | | | | 1.28449000524922e-20 | | | | 1.98982696735282e-18 | | | | | 36.0623087757381 | | | | |
| -1.102982803 | | | | | 0.33872090774891 | | | | -9.58952737 | | | | | 1.29918538147928e-20 | | | | 2.00890172280297e-18 | | | | | 36.0529712250243 | | | | |
| -1.261737298 | | | | | -0.559076034 | | | | -9.582809354 | | | | | 1.29814420699147e-20 | | | | 2.00802812825419e-18 | | | | | 36.0520348323714 | | | | |
| -1.055273857 | | | | | -0.43453605 | | | | -9.595154615 | | | | | 1.31640570865763e-20 | | | | 2.03329228999877e-18 | | | | | 36.0409174802993 | | | | |
| -1.321109758 | | | | | 0.262504844294309 | | | | -9.580063891 | | | | | 1.33783424721311e-20 | | | | 2.06487762453278e-18 | | | | | 36.0229203818394 | | | | |
| 1.10573789175774 | | | | | 0.825037752970921 | | | | 9.57572162996546 | | | | | 1.37574615886378e-20 | | | | 2.11256694394788e-18 | | | | | 35.9951003591607 | | | | |
| -1.32664935 | | | | | 0.270675557030002 | | | | -9.577148236 | | | | | 1.38523511922732e-20 | | | | 2.12558985707636e-18 | | | | | 35.9889686855576 | | | | |
| -1.067720969 | | | | | -0.861753016 | | | | -9.574607528 | | | | | 1.3890505699161e-20 | | | | 2.12989437751462e-18 | | | | | 35.9856761843987 | | | | |
| -1.252006109 | | | | | -0.415888674 | | | | -9.576625317 | | | | | 1.40052213912223e-20 | | | | 2.14436517938878e-18 | | | | | 35.9786332283938 | | | | |
| -1.289253028 | | | | | 0.370668051042016 | | | | -9.581229193 | | | | | 1.44840762693781e-20 | | | | 2.2120610070658e-18 | | | | | 35.9472180474573 | | | | |
| -1.220908206 | | | | | -0.493048686 | | | | -9.643742599 | | | | | 1.4506542442669e-20 | | | | 2.21388843713364e-18 | | | | | 35.9409116981314 | | | | |
| -1.042506649 | | | | | -1.216239725 | | | | -9.567019372 | | | | | 1.48312059794901e-20 | | | | 2.25690170529468e-18 | | | | | 35.9215108795652 | | | | |
| -1.357660142 | | | | | -0.836405016 | | | | -9.566144938 | | | | | 1.50386516591122e-20 | | | | 2.28434735053956e-18 | | | | | 35.9081943971845 | | | | |
| -1.169222698 | | | | | -0.358586381 | | | | -9.563750833 | | | | | 1.54506223324797e-20 | | | | 2.34233844575768e-18 | | | | | 35.8818933005171 | | | | |
| -1.373634786 | | | | | -0.132195246 | | | | -9.670018773 | | | | | 1.54537566250809e-20 | | | | 2.34233844575768e-18 | | | | | 35.8736389209316 | | | | |
| -1.374703054 | | | | | 1.78617894287198 | | | | -9.561323471 | | | | | 1.56278666273148e-20 | | | | 2.36363074631988e-18 | | | | | 35.8703582180066 | | | | |
| 1.05451762639822 | | | | | 0.750804192616724 | | | | 9.55999656255426 | | | | | 1.57579323350849e-20 | | | | 2.37903592113686e-18 | | | | | 35.862161647509 | | | | |
| -1.002324695 | | | | | -0.226380153 | | | | -9.558322942 | | | | | 1.62947615882874e-20 | | | | 2.45480961733946e-18 | | | | | 35.829971348086 | | | | |
| -1.343233762 | | | | | 0.193656869905122 | | | | -9.562223291 | | | | | 1.64373833519596e-20 | | | | 2.47541122786457e-18 | | | | | 35.8228355969636 | | | | |
| -1.000980332 | | | | | -2.951316117 | | | | -9.55496046 | | | | | 1.64575943238525e-20 | | | | 2.47668586671623e-18 | | | | | 35.8196229433095 | | | | |
| -1.275439639 | | | | | 0.207165360750235 | | | | -9.569582484 | | | | | 1.66897521973706e-20 | | | | 2.5089368303263e-18 | | | | | 35.809669526077 | | | | |
| -1.268161296 | | | | | 0.325622514615291 | | | | -9.558710159 | | | | | 1.67204598493258e-20 | | | | 2.51086762986653e-18 | | | | | 35.8057173885329 | | | | |
| 1.15473493393114 | | | | | 2.8736371837411 | | | | 9.55060401752403 | | | | | 1.70876001790311e-20 | | | | 2.55871035777416e-18 | | | | | 35.7828393324715 | | | | |
| -1.424032176 | | | | | 0.859664596946311 | | | | -9.621296164 | | | | | 1.67748807696712e-20 | | | | 2.5172469658887e-18 | | | | | 35.7733965165563 | | | | |
| -1.101953978 | | | | | 1.19437751201006 | | | | -9.550135585 | | | | | 1.78263016631214e-20 | | | | 2.66459291821637e-18 | | | | | 35.7429061545079 | | | | |
| -1.005888833 | | | | | -0.629826362 | | | | -9.543860979 | | | | | 1.82819797316078e-20 | | | | 2.72787062753966e-18 | | | | | 35.7171842731421 | | | | |
| -1.255017543 | | | | | -1.966476506 | | | | -9.537495787 | | | | | 1.91309513596913e-20 | | | | 2.84548439500565e-18 | | | | | 35.6722393394606 | | | | |
| 1.06456656641391 | | | | | 1.77896979834337 | | | | 9.53666398977796 | | | | | 1.92684906307092e-20 | | | | 2.86493104522256e-18 | | | | | 35.6652251158923 | | | | |
| -1.118891663 | | | | | -0.867616927 | | | | -9.536390579 | | | | | 1.93139131904762e-20 | | | | 2.8705976346708e-18 | | | | | 35.6629196537022 | | | | |
| -1.371583077 | | | | | -0.350317326 | | | | -9.533468273 | | | | | 1.98060806189778e-20 | | | | 2.93761168294209e-18 | | | | | 35.6382813302664 | | | | |
| -1.145769393 | | | | | 0.309250408343784 | | | | -9.532851139 | | | | | 2.00997037889852e-20 | | | | 2.97906576333968e-18 | | | | | 35.6242580703552 | | | | |
| -1.023130929 | | | | | -1.061578885 | | | | -9.530831576 | | | | | 2.04520559265769e-20 | | | | 3.03022432275463e-18 | | | | | 35.6072436680295 | | | | |
| -1.268008174 | | | | | -0.264503521 | | | | -9.561441094 | | | | | 2.058833293323e-20 | | | | 3.0472033513356e-18 | | | | | 35.6062674096744 | | | | |
| -1.053925423 | | | | | -0.046895826 | | | | -9.53560154 | | | | | 2.05300262598755e-20 | | | | 3.04070817457032e-18 | | | | | 35.6050857081693 | | | | |
| 1.29817966005214 | | | | | -3.629635578 | | | | 9.5292550918933 | | | | | 2.05375980742259e-20 | | | | 3.04076158004172e-18 | | | | | 35.6027699166505 | | | | |
| -1.135456466 | | | | | -0.444184375 | | | | -9.548237944 | | | | | 2.06883131366286e-20 | | | | 3.05878015439066e-18 | | | | | 35.5999015466916 | | | | |
| -1.017070261 | | | | | -0.470116764 | | | | -9.51898542 | | | | | 2.24347267207106e-20 | | | | 3.29045259698157e-18 | | | | | 35.5162620875962 | | | | |
| 1.04561674803494 | | | | | 1.15730289848531 | | | | 9.51741983902356 | | | | | 2.27388010204111e-20 | | | | 3.33227381439084e-18 | | | | | 35.5030806617495 | | | | |
| 1.63803024338037 | | | | | -2.196979645 | | | | 9.51528732589705 | | | | | 2.31595653346729e-20 | | | | 3.38498922518943e-18 | | | | | 35.4851286796713 | | | | |
| 1.40605562048506 | | | | | 3.20599663636508 | | | | 9.51501405330832 | | | | | 2.32862356923292e-20 | | | | 3.40114548125543e-18 | | | | | 35.4798699584582 | | | | |
| -1.1299626 | | | | | -0.310602731 | | | | -9.51290127 | | | | | 2.36395039458669e-20 | | | | 3.44320194433634e-18 | | | | | 35.4650460598557 | | | | |
| 1.03067600316115 | | | | | 1.57084688573649 | | | | 9.51208795632726 | | | | | 2.38053374370476e-20 | | | | 3.46496259478076e-18 | | | | | 35.4582015818464 | | | | |
| -1.26397575 | | | | | 0.132652504548805 | | | | -9.513303932 | | | | | 2.44639973085273e-20 | | | | 3.5534735601401e-18 | | | | | 35.4324732845917 | | | | |
| -1.123653264 | | | | | -0.653569004 | | | | -9.516240914 | | | | | 2.48019438764711e-20 | | | | 3.59884228299779e-18 | | | | | 35.4207049946292 | | | | |
| -1.508707235 | | | | | 1.85826659925214 | | | | -9.505818008 | | | | | 2.51230565984305e-20 | | | | 3.64418275743384e-18 | | | | | 35.4054519703514 | | | | |
| -1.267136562 | | | | | -0.139218508 | | | | -9.510055219 | | | | | 2.5235931173666e-20 | | | | 3.65803887865237e-18 | | | | | 35.4024183174787 | | | | |
| -1.200750038 | | | | | 2.21953407901685 | | | | -9.504556131 | | | | | 2.53968648356558e-20 | | | | 3.67883751125077e-18 | | | | | 35.3948389927102 | | | | |
| -1.060280923 | | | | | -0.826307496 | | | | -9.49888036 | | | | | 2.66654265561944e-20 | | | | 3.84805284597895e-18 | | | | | 35.3471167861478 | | | | |
| -1.66074551 | | | | | 0.23402339253112 | | | | -9.680272698 | | | | | 2.64373542291633e-20 | | | | 3.81906103385108e-18 | | | | | 35.330478511094 | | | | |
| -1.123319751 | | | | | -0.11272551 | | | | -9.496457318 | | | | | 2.77373890885911e-20 | | | | 3.99318021747566e-18 | | | | | 35.3091830498135 | | | | |
| -1.271978631 | | | | | -0.466352905 | | | | -9.490552503 | | | | | 2.87293989424996e-20 | | | | 4.12809114236793e-18 | | | | | 35.2742070411897 | | | | |
| 1.0500218184733 | | | | | 0.794543768149175 | | | | 9.49003604192654 | | | | | 2.87683577539438e-20 | | | | 4.13172800207953e-18 | | | | | 35.2727979200569 | | | | |
| -1.405421985 | | | | | 0.728665582159475 | | | | -9.499357118 | | | | | 2.92320484808097e-20 | | | | 4.19261152479695e-18 | | | | | 35.2602900290885 | | | | |
| -1.134453481 | | | | | -0.193851226 | | | | -9.549200829 | | | | | 3.07899156686407e-20 | | | | 4.4055594638601e-18 | | | | | 35.2082690375785 | | | | |
| 1.03176615294536 | | | | | 1.45652799720733 | | | | 9.48108044936724 | | | | | 3.11604233399924e-20 | | | | 4.45403922365241e-18 | | | | | 35.1946815580168 | | | | |
| 1.12034180893235 | | | | | 1.99392268833329 | | | | 9.47770659470635 | | | | | 3.19766390849138e-20 | | | | 4.55988819849022e-18 | | | | | 35.1692843432734 | | | | |
| -1.398895678 | | | | | 0.240044230873983 | | | | -9.607138414 | | | | | 3.09608764974098e-20 | | | | 4.42701688459233e-18 | | | | | 35.1689634814705 | | | | |
| 1.06084685997431 | | | | | -1.06130997 | | | | 9.47250706722664 | | | | | 3.34336140568826e-20 | | | | 4.75478989523295e-18 | | | | | 35.1256627316233 | | | | |
| -1.096134953 | | | | | -2.252615254 | | | | -9.467684992 | | | | | 3.48435037638161e-20 | | | | 4.93366696846485e-18 | | | | | 35.0852246055816 | | | | |
| -1.473997738 | | | | | -0.196625035 | | | | -9.467505263 | | | | | 3.50037283233845e-20 | | | | 4.95136602553557e-18 | | | | | 35.0808158949743 | | | | |
| 1.03735149257582 | | | | | 2.81965089407085 | | | | 9.46421382884629 | | | | | 3.58947118260245e-20 | | | | 5.05535174872403e-18 | | | | | 35.056125319975 | | | | |
| -1.21138792 | | | | | 0.0275763445531915 | | | | -9.464424505 | | | | | 3.64947527795304e-20 | | | | 5.13300280338378e-18 | | | | | 35.0403950483586 | | | | |
| 1.28288758381367 | | | | | 1.06390915014317 | | | | 9.46085458270271 | | | | | 3.70544233496269e-20 | | | | 5.1995802641721e-18 | | | | | 35.0250783159601 | | | | |
| -1.537354071 | | | | | -0.064689249 | | | | -9.579486892 | | | | | 3.72249373107366e-20 | | | | 5.21829764488641e-18 | | | | | 35.0207977971669 | | | | |
| -1.276220236 | | | | | 0.312756026027485 | | | | -9.49560094 | | | | | 3.78788678290519e-20 | | | | 5.30115572435325e-18 | | | | | 35.0057742674204 | | | | |
| -1.190392921 | | | | | -0.679250162 | | | | -9.455753295 | | | | | 3.85903288447743e-20 | | | | 5.38820661058807e-18 | | | | | 34.9852345825416 | | | | |
| -1.052287146 | | | | | -3.387104975 | | | | -9.451291105 | | | | | 4.00918259631275e-20 | | | | 5.58306481303566e-18 | | | | | 34.947866046426 | | | | |
| -1.009956223 | | | | | -0.922309687 | | | | -9.448539985 | | | | | 4.10462461543051e-20 | | | | 5.70091258757767e-18 | | | | | 34.9248337648899 | | | | |
| -1.15372518 | | | | | 0.272839680811885 | | | | -9.455724777 | | | | | 4.1205439983837e-20 | | | | 5.71925539104165e-18 | | | | | 34.9238258327406 | | | | |
| -1.022178309 | | | | | -0.971545062 | | | | -9.447821606 | | | | | 4.12991500960987e-20 | | | | 5.73037598585783e-18 | | | | | 34.9188203919414 | | | | |
| -1.563222291 | | | | | -0.178729521 | | | | -9.721930017 | | | | | 3.72860638933951e-20 | | | | 5.22512946557923e-18 | | | | | 34.8760741040641 | | | | |
| -1.021803261 | | | | | -0.27035476 | | | | -9.439442348 | | | | | 4.4634236791462e-20 | | | | 6.14460288209461e-18 | | | | | 34.8429618943525 | | | | |
| -1.082716016 | | | | | -0.703122652 | | | | -9.475912105 | | | | | 4.54425201895268e-20 | | | | 6.24568040688324e-18 | | | | | 34.8308691143647 | | | | |
| -1.029977029 | | | | | -1.985128467 | | | | -9.438900806 | | | | | 4.56688886065912e-20 | | | | 6.27270366734245e-18 | | | | | 34.8215606660612 | | | | |
| 1.11170268028636 | | | | | -0.232612039 | | | | 9.43241326000112 | | | | | 4.71109404862226e-20 | | | | 6.45232947415422e-18 | | | | | 34.7899276712284 | | | | |
| 1.06246926202632 | | | | | 3.00104481144637 | | | | 9.42888722830458 | | | | | 4.85508506033992e-20 | | | | 6.63612226059493e-18 | | | | | 34.7604553045223 | | | | |
| -1.347949149 | | | | | 0.468084533651646 | | | | -9.430804171 | | | | | 4.86372066603404e-20 | | | | 6.64577152704658e-18 | | | | | 34.7592200528963 | | | | |
| -1.208569966 | | | | | -0.334512096 | | | | -9.440453453 | | | | | 4.9594742947869e-20 | | | | 6.77002760078599e-18 | | | | | 34.7438716564401 | | | | |
| 1.25294961389185 | | | | | 0.893357589449889 | | | | 9.42147319260158 | | | | | 5.21900897601469e-20 | | | | 7.10590737783699e-18 | | | | | 34.6901522329803 | | | | |
| 1.07049626300888 | | | | | 1.69187494684095 | | | | 9.41563093828475 | | | | | 5.43650167414003e-20 | | | | 7.39010206619802e-18 | | | | | 34.649730272676 | | | | |
| 1.06068261903557 | | | | | 2.7470505978597 | | | | 9.41549346994145 | | | | | 5.4428786498184e-20 | | | | 7.39638620776322e-18 | | | | | 34.6485826929743 | | | | |
| -1.241476453 | | | | | -1.706247027 | | | | -9.416234854 | | | | | 5.49039955984097e-20 | | | | 7.45375654345828e-18 | | | | | 34.6408894249186 | | | | |
| -1.318054997 | | | | | 0.157374028409618 | | | | -9.434520146 | | | | | 5.52428511451563e-20 | | | | 7.48770589597494e-18 | | | | | 34.6389945898902 | | | | |
| -1.025576406 | | | | | -0.272149297 | | | | -9.41327929 | | | | | 5.5466180947676e-20 | | | | 7.51314632836702e-18 | | | | | 34.6301006406031 | | | | |
| -1.084165435 | | | | | 0.861612576208586 | | | | -9.410431044 | | | | | 5.78616806808265e-20 | | | | 7.81503359791292e-18 | | | | | 34.5897978155755 | | | | |
| 1.96204057462987 | | | | | -3.503875572 | | | | 9.42238813264172 | | | | | 5.82007562941736e-20 | | | | 7.85831345070899e-18 | | | | | 34.5872005909268 | | | | |
| -1.212304843 | | | | | 0.738422188903216 | | | | -9.443593913 | | | | | 5.94419579665055e-20 | | | | 8.01819910932065e-18 | | | | | 34.5654898314589 | | | | |
| 1.03508059277002 | | | | | 1.07320272461526 | | | | 9.40399169787597 | | | | | 6.00352257521783e-20 | | | | 8.09159585971572e-18 | | | | | 34.5526132583939 | | | | |
| -1.169894982 | | | | | 0.0764578133669605 | | | | -9.480669625 | | | | | 6.12836297043993e-20 | | | | 8.24289254783223e-18 | | | | | 34.5398664392017 | | | | |
| -1.106039989 | | | | | -1.83374199 | | | | -9.421949701 | | | | | 6.27382328685453e-20 | | | | 8.42510530371958e-18 | | | | | 34.515655 | | | | |
| -1.263090256 | | | | | 0.211150544684999 | | | | -9.413929775 | | | | | 6.37876922834191e-20 | | | | 8.5524185072017e-18 | | | | | 34.4982668510865 | | | | |
| 1.23611384172247 | | | | | 1.43775570096332 | | | | 9.39610025529223 | | | | | 6.42089618678944e-20 | | | | 8.5996907626922e-18 | | | | | 34.4868215774138 | | | | |
| -1.155616186 | | | | | 0.0193885137264231 | | | | -9.395707897 | | | | | 6.5000749150218e-20 | | | | 8.69570618469937e-18 | | | | | 34.4752949930553 | | | | |
| -1.066643091 | | | | | -0.857777627 | | | | -9.391650738 | | | | | 6.68853481298521e-20 | | | | 8.92799770367671e-18 | | | | | 34.4469319536291 | | | | |
| -1.151750619 | | | | | -0.231601338 | | | | -9.39179263 | | | | | 7.09893413605086e-20 | | | | 9.43994814616388e-18 | | | | | 34.3913908032481 | | | | |
| -1.369356986 | | | | | -2.385823439 | | | | -9.381791887 | | | | | 7.25224364994958e-20 | | | | 9.61652069142843e-18 | | | | | 34.3676427191044 | | | | |
| -1.346330131 | | | | | 1.31632586550918 | | | | -9.376241329 | | | | | 7.6026990743214e-20 | | | | 1.00401820190075e-17 | | | | | 34.3214490449791 | | | | |
| -1.69953769 | | | | | -1.117081928 | | | | -9.40620843 | | | | | 7.75266926153446e-20 | | | | 1.02158376484726e-17 | | | | | 34.3100443874223 | | | | |
| 1.03211261220519 | | | | | 0.638915616561117 | | | | 9.37480444082162 | | | | | 7.69612266413807e-20 | | | | 1.01526232001552e-17 | | | | | 34.3094942816034 | | | | |
| -1.108404879 | | | | | -0.675964919 | | | | -9.376524506 | | | | | 7.8134526606065e-20 | | | | 1.02862896765468e-17 | | | | | 34.295970385249 | | | | |
| -1.275163628 | | | | | 0.721281625919637 | | | | -9.369530411 | | | | | 8.04887787100141e-20 | | | | 1.05830069593551e-17 | | | | | 34.2656273274467 | | | | |
| 1.10209710283179 | | | | | 0.442090863353346 | | | | 9.36742799638334 | | | | | 8.19392084584685e-20 | | | | 1.07469070079883e-17 | | | | | 34.2481458491201 | | | | |
| -1.367700606 | | | | | 2.16771022616674 | | | | -9.397162984 | | | | | 8.36554893314622e-20 | | | | 1.09447751121308e-17 | | | | | 34.2348435624019 | | | | |
| -1.176471413 | | | | | -1.075392271 | | | | -9.365090313 | | | | | 8.35823514913651e-20 | | | | 1.09419963220627e-17 | | | | | 34.2287117742767 | | | | |
| -1.182348825 | | | | | 0.298484179343093 | | | | -9.382778129 | | | | | 8.4697120382449e-20 | | | | 1.10638893282736e-17 | | | | | 34.2215428537185 | | | | |
| 1.29477349344727 | | | | | 0.609765616635631 | | | | 9.36405309521074 | | | | | 8.43218168999936e-20 | | | | 1.10251102425489e-17 | | | | | 34.2200902014936 | | | | |
| 1.11202076350665 | | | | | -0.978053349 | | | | 9.36088364716446 | | | | | 8.66217966783638e-20 | | | | 1.13013035288879e-17 | | | | | 34.1937497762634 | | | | |
| 1.51759964753719 | | | | | 2.12632818035168 | | | | 9.36013696022594 | | | | | 8.71726252910946e-20 | | | | 1.13626216094268e-17 | | | | | 34.1875452903002 | | | | |
| -1.258658684 | | | | | 0.301566337211206 | | | | -9.386620761 | | | | | 9.59337088678265e-20 | | | | 1.24201311078589e-17 | | | | | 34.1022652227548 | | | | |
| -1.185319673 | | | | | 0.395477930227057 | | | | -9.34962838 | | | | | 9.58600893497182e-20 | | | | 1.24182254611661e-17 | | | | | 34.0949748383556 | | | | |
| -1.487910715 | | | | | 0.077805847993656 | | | | -9.484860098 | | | | | 9.72414942038436e-20 | | | | 1.25662950845647e-17 | | | | | 34.0780124597828 | | | | |
| -1.004845949 | | | | | -0.822815261 | | | | -9.347617482 | | | | | 9.83695473349476e-20 | | | | 1.269650658853e-17 | | | | | 34.0703840660074 | | | | |
| -1.240112594 | | | | | -0.405954811 | | | | -9.453558767 | | | | | 9.99996863364001e-20 | | | | 1.28911243465209e-17 | | | | | 34.057652709228 | | | | |
| -1.087239091 | | | | | -0.150073761 | | | | -9.342381474 | | | | | 1.01637302011529e-19 | | | | 1.30862293554814e-17 | | | | | 34.0373681172421 | | | | |
| -1.245790503 | | | | | 0.149401553277923 | | | | -9.344278253 | | | | | 1.03934317219416e-19 | | | | 1.3353438007895e-17 | | | | | 34.0174888092055 | | | | |
| -2.209524912 | | | | | 3.38885975441799 | | | | -9.339553841 | | | | | 1.03800777788491e-19 | | | | 1.33403456172121e-17 | | | | | 34.0166671862517 | | | | |
| -1.566443983 | | | | | 0.253802085539677 | | | | -9.354970797 | | | | | 1.04978746205653e-19 | | | | 1.34761267764397e-17 | | | | | 34.0110624712158 | | | | |
| -1.152794694 | | | | | -2.308121575 | | | | -9.33529673 | | | | | 1.07613339341307e-19 | | | | 1.37715680728524e-17 | | | | | 33.9813624453953 | | | | |
| -2.026392551 | | | | | 0.473169993513817 | | | | -9.552870446 | | | | | 1.1006292266744e-19 | | | | 1.40466805693642e-17 | | | | | 33.9514135369177 | | | | |
| -1.015715073 | | | | | -1.120155045 | | | | -9.459484624 | | | | | 1.12069150026258e-19 | | | | 1.42638691492822e-17 | | | | | 33.9426399163563 | | | | |
| -1.099979099 | | | | | 0.325991025470127 | | | | -9.341341464 | | | | | 1.12954709004459e-19 | | | | 1.43549162585624e-17 | | | | | 33.938446792608 | | | | |
| -1.108183446 | | | | | 0.0985051533532555 | | | | -9.337895843 | | | | | 1.13079200287502e-19 | | | | 1.43664074676803e-17 | | | | | 33.9363167983447 | | | | |
| -1.056185933 | | | | | 0.218934276629766 | | | | -9.336688757 | | | | | 1.16016419708331e-19 | | | | 1.47218307849569e-17 | | | | | 33.9120735562292 | | | | |
| -1.335198378 | | | | | -1.203297776 | | | | -9.356917277 | | | | | 1.18132441424792e-19 | | | | 1.4969392960137e-17 | | | | | 33.8990395749265 | | | | |
| -1.135404582 | | | | | -0.144811393 | | | | -9.352969761 | | | | | 1.18231348744534e-19 | | | | 1.49758524557247e-17 | | | | | 33.896622086311 | | | | |
| -1.341497852 | | | | | 0.0554323577758072 | | | | -9.332956194 | | | | | 1.17895291896828e-19 | | | | 1.49467551816402e-17 | | | | | 33.8953645129265 | | | | |
| -2.006960435 | | | | | 0.310230312271178 | | | | -9.612163131 | | | | | 1.12186613291664e-19 | | | | 1.4274510931411e-17 | | | | | 33.8795458590073 | | | | |
| -1.155934348 | | | | | -0.697680211 | | | | -9.358111146 | | | | | 1.2089254637561e-19 | | | | 1.52762241624823e-17 | | | | | 33.8770050420745 | | | | |
| 1.09423996416149 | | | | | 2.5144209813043 | | | | 9.32028316168353 | | | | | 1.22199542098273e-19 | | | | 1.54182767554096e-17 | | | | | 33.8569549715197 | | | | |
| -1.060669553 | | | | | -0.597545265 | | | | -9.336201852 | | | | | 1.23359833018722e-19 | | | | 1.55367804029285e-17 | | | | | 33.8535793144121 | | | | |
| -1.023906725 | | | | | -1.154988445 | | | | -9.313819027 | | | | | 1.29067358364724e-19 | | | | 1.6187933670926e-17 | | | | | 33.8034398789964 | | | | |
| -1.050708711 | | | | | -1.728592177 | | | | -9.332765476 | | | | | 1.33003960906991e-19 | | | | 1.66519537398014e-17 | | | | | 33.7811102151066 | | | | |
| -1.205515819 | | | | | 1.36537828542795 | | | | -9.324889347 | | | | | 1.33082048054347e-19 | | | | 1.66567845660661e-17 | | | | | 33.7789666343733 | | | | |
| -1.524918288 | | | | | 0.738786291501174 | | | | -9.424680179 | | | | | 1.31867080091245e-19 | | | | 1.65145208381571e-17 | | | | | 33.7697500915136 | | | | |
| -1.183299048 | | | | | -1.81566876 | | | | -9.30613596 | | | | | 1.37728741793686e-19 | | | | 1.71975358460597e-17 | | | | | 33.739871851389 | | | | |
| -1.223485314 | | | | | 0.932276022486348 | | | | -9.355538909 | | | | | 1.42213026123145e-19 | | | | 1.77259730196117e-17 | | | | | 33.7179138059341 | | | | |
| 1.06799868279593 | | | | | -0.265670439 | | | | 9.30205203239498 | | | | | 1.42564458445285e-19 | | | | 1.77645257661416e-17 | | | | | 33.7060992631902 | | | | |
| -1.176542906 | | | | | 0.261332218467866 | | | | -9.309537663 | | | | | 1.44076529074661e-19 | | | | 1.79264532354418e-17 | | | | | 33.699582206987 | | | | |
| -1.401523596 | | | | | 0.854392062224024 | | | | -9.307961086 | | | | | 1.46896466823683e-19 | | | | 1.82503928006901e-17 | | | | | 33.6802921621983 | | | | |
| -1.349685157 | | | | | 0.172220886441822 | | | | -9.297866331 | | | | | 1.51134123866199e-19 | | | | 1.8727978954563e-17 | | | | | 33.649932877844 | | | | |
| -1.136477316 | | | | | 0.0375958212666119 | | | | -9.302450804 | | | | | 1.52474392661948e-19 | | | | 1.88766521297015e-17 | | | | | 33.6444652878622 | | | | |
| -1.152700076 | | | | | 0.233893900917209 | | | | -9.299028164 | | | | | 1.54130288690403e-19 | | | | 1.9048100478336e-17 | | | | | 33.6325027425684 | | | | |
| 1.07898768366561 | | | | | 3.0318708790751 | | | | 9.29045849485167 | | | | | 1.57227324122071e-19 | | | | 1.93853934978227e-17 | | | | | 33.6102890880246 | | | | |
| -1.092095466 | | | | | -0.081452547 | | | | -9.295988671 | | | | | 1.61486760249776e-19 | | | | 1.98640963227897e-17 | | | | | 33.5883906409323 | | | | |
| 1.26828991592477 | | | | | 0.965346431772403 | | | | 9.28884504899967 | | | | | 1.63080660557937e-19 | | | | 2.00426179357229e-17 | | | | | 33.5757449278191 | | | | |
| -1.011947184 | | | | | -0.499735248 | | | | -9.285996561 | | | | | 1.67527944713754e-19 | | | | 2.05353222230955e-17 | | | | | 33.549244264377 | | | | |
| -1.18428446 | | | | | -0.687839355 | | | | -9.285486422 | | | | | 1.68249652527245e-19 | | | | 2.06127835927486e-17 | | | | | 33.5457815285568 | | | | |
| 1.34107964708503 | | | | | 1.74363388227208 | | | | 9.28238541059937 | | | | | 1.68310040128582e-19 | | | | 2.06132136569908e-17 | | | | | 33.5436283535729 | | | | |
| -1.13528914 | | | | | -0.900689222 | | | | -9.279888767 | | | | | 1.7535914616933e-19 | | | | 2.13648341997172e-17 | | | | | 33.5043642248118 | | | | |
| -1.015362834 | | | | | -0.707089383 | | | | -9.330378545 | | | | | 1.77414759132364e-19 | | | | 2.15840973696896e-17 | | | | | 33.5022780177566 | | | | |
| -1.185142924 | | | | | -0.516326801 | | | | -9.276178106 | | | | | 1.77354307129873e-19 | | | | 2.1582969895369e-17 | | | | | 33.4924049714589 | | | | |
| -1.198011877 | | | | | -0.007424217 | | | | -9.274926419 | | | | | 1.79235529514406e-19 | | | | 2.17741992884873e-17 | | | | | 33.48207921 | | | | |
| 1.02611305065674 | | | | | 0.212347477859039 | | | | 9.27294687507096 | | | | | 1.82251090704358e-19 | | | | 2.20896284614276e-17 | | | | | 33.4657512686419 | | | | |
| 1.0426435426743 | | | | | 0.0214512621111841 | | | | 9.26966577900083 | | | | | 1.87360398689226e-19 | | | | 2.26438117842608e-17 | | | | | 33.4386937920412 | | | | |
| -1.121740809 | | | | | -0.535707213 | | | | -9.26711941 | | | | | 1.92506328470098e-19 | | | | 2.32125088721722e-17 | | | | | 33.4123524942381 | | | | |
| -1.007064325 | | | | | -0.785819166 | | | | -9.276130613 | | | | | 1.9492550780862e-19 | | | | 2.34773604334935e-17 | | | | | 33.4045311095764 | | | | |
| -1.221323262 | | | | | -2.443361638 | | | | -9.264310839 | | | | | 1.96005353319339e-19 | | | | 2.36006788504185e-17 | | | | | 33.3945507690837 | | | | |
| -1.007564715 | | | | | -0.604979497 | | | | -9.262807835 | | | | | 1.98501977723158e-19 | | | | 2.38672166941899e-17 | | | | | 33.3821645200668 | | | | |
| 1.18516811292487 | | | | | 1.51794188524364 | | | | 9.27972299146618 | | | | | 2.00116794969679e-19 | | | | 2.40339643790557e-17 | | | | | 33.3804932513062 | | | | |
| 1.29516839805612 | | | | | -3.754303688 | | | | 9.26018132253917 | | | | | 2.02940658960663e-19 | | | | 2.43315290486047e-17 | | | | | 33.3605232805484 | | | | |
| -1.309621397 | | | | | 1.2312639985005 | | | | -9.264519738 | | | | | 2.1164028120568e-19 | | | | 2.53169808158862e-17 | | | | | 33.3240926039315 | | | | |
| -1.360330458 | | | | | 0.691130821690809 | | | | -9.290586517 | | | | | 2.15487756722665e-19 | | | | 2.57334246324685e-17 | | | | | 33.3113146486336 | | | | |
| 1.01670923417992 | | | | | 1.00411733453544 | | | | 9.2522372455979 | | | | | 2.16972595869636e-19 | | | | 2.58667895109837e-17 | | | | | 33.2950975078775 | | | | |
| 1.04216997136668 | | | | | 2.1903480020912 | | | | 9.25110587131152 | | | | | 2.19047608338826e-19 | | | | 2.61067849090539e-17 | | | | | 33.285783378428 | | | | |
| -1.354361466 | | | | | -1.121493406 | | | | -9.249149884 | | | | | 2.23932568710258e-19 | | | | 2.66513255004387e-17 | | | | | 33.2643749798331 | | | | |
| -1.196087677 | | | | | 0.363076039537907 | | | | -9.248033065 | | | | | 2.25412514659677e-19 | | | | 2.68123258269523e-17 | | | | | 33.2578414847562 | | | | |
| -1.128836525 | | | | | -1.488457016 | | | | -9.24496762 | | | | | 2.33253873153624e-19 | | | | 2.76592127932195e-17 | | | | | 33.2246426559022 | | | | |
| 1.01864867530864 | | | | | 3.26029784903313 | | | | 9.24408571891558 | | | | | 2.34988911244872e-19 | | | | 2.78336444395015e-17 | | | | | 33.21797669 | | | | |
| -1.628799974 | | | | | -0.487108429 | | | | -9.301352736 | | | | | 2.40132332054032e-19 | | | | 2.84037002864811e-17 | | | | | 33.2100480297194 | | | | |
| -1.117068535 | | | | | -0.396332517 | | | | -9.239409352 | | | | | 2.41685363133357e-19 | | | | 2.8538635416534e-17 | | | | | 33.1895439887579 | | | | |
| -1.293497579 | | | | | 1.64130502260788 | | | | -9.243707856 | | | | | 2.44671054707811e-19 | | | | 2.88588653534665e-17 | | | | | 33.1811819334669 | | | | |
| -1.237467321 | | | | | -0.050832335 | | | | -9.241103378 | | | | | 2.54505019316838e-19 | | | | 2.99016805503848e-17 | | | | | 33.142761885191 | | | | |
| -1.192117197 | | | | | 0.428721390942902 | | | | -9.268612629 | | | | | 2.5864758965324e-19 | | | | 3.03265573335658e-17 | | | | | 33.1306740840344 | | | | |
| -1.432669306 | | | | | -0.865761187 | | | | -9.232195608 | | | | | 2.5821915675227e-19 | | | | 3.02958463627517e-17 | | | | | 33.1249674697842 | | | | |
| -1.069113034 | | | | | -1.765039238 | | | | -9.231335119 | | | | | 2.58648704076677e-19 | | | | 3.03265573335658e-17 | | | | | 33.1231654689998 | | | | |
| -1.132512851 | | | | | 0.164254791322915 | | | | -9.229071153 | | | | | 2.65082409358778e-19 | | | | 3.10147889995328e-17 | | | | | 33.099298675419 | | | | |
| -1.679313798 | | | | | 2.25736214277196 | | | | -9.227924335 | | | | | 2.66163814461352e-19 | | | | 3.1124042053222e-17 | | | | | 33.095139200957 | | | | |
| 1.08364158902748 | | | | | -4.521317009 | | | | 9.22676187119223 | | | | | 2.68774186727674e-19 | | | | 3.1394462968825e-17 | | | | | 33.0855891714201 | | | | |
| 1.24980279694001 | | | | | 2.77385654062532 | | | | 9.22682336385505 | | | | | 2.69381455764681e-19 | | | | 3.14566819308483e-17 | | | | | 33.0834685783229 | | | | |
| -1.067514728 | | | | | -1.425119238 | | | | -9.224695975 | | | | | 2.73475955129638e-19 | | | | 3.18642182922118e-17 | | | | | 33.0686195088627 | | | | |
| 1.33646564659931 | | | | | 0.38293893216949 | | | | 9.22418927649726 | | | | | 2.74641529564072e-19 | | | | 3.19823512210114e-17 | | | | | 33.064457849088 | | | | |
| -1.033188154 | | | | | -0.809910033 | | | | -9.303198714 | | | | | 2.79393688650776e-19 | | | | 3.24998446050683e-17 | | | | | 33.0588373746729 | | | | |
| -1.487806957 | | | | | 0.432860011570721 | | | | -9.295499491 | | | | | 2.81355703279582e-19 | | | | 3.27010086554303e-17 | | | | | 33.0531972949553 | | | | |
| 1.06676211430475 | | | | | 2.44962704586963 | | | | 9.2211516848145 | | | | | 2.81732817569684e-19 | | | | 3.27358162536811e-17 | | | | | 33.0395130822671 | | | | |
| -1.06774944 | | | | | 0.143628801986529 | | | | -9.220969036 | | | | | 2.85316506624189e-19 | | | | 3.31248379262726e-17 | | | | | 33.0278086515588 | | | | |
| 1.18709213365401 | | | | | 2.49676760669411 | | | | 9.21763769215758 | | | | | 2.90162637215201e-19 | | | | 3.3604196439037e-17 | | | | | 33.010664270409 | | | | |
| -1.311773711 | | | | | -1.178534119 | | | | -9.21840604 | | | | | 2.90704596687602e-19 | | | | 3.36577175412579e-17 | | | | | 33.0091027970026 | | | | |
| -1.131514577 | | | | | -0.957623405 | | | | -9.217613902 | | | | | 2.91023514628373e-19 | | | | 3.36853926470893e-17 | | | | | 33.0081689476378 | | | | |
| -1.21786652 | | | | | 0.155437891250059 | | | | -9.216928879 | | | | | 3.0016039517825e-19 | | | | 3.46289011859952e-17 | | | | | 32.9790107649329 | | | | |
| -1.067477795 | | | | | 0.514191015797395 | | | | -9.212591241 | | | | | 3.03543044691587e-19 | | | | 3.49617578954115e-17 | | | | | 32.9666397768255 | | | | |
| -1.428111305 | | | | | 2.14959837071645 | | | | -9.219097458 | | | | | 3.0778531589999e-19 | | | | 3.54116873548564e-17 | | | | | 32.9563340437041 | | | | |
| 1.12234741782188 | | | | | 0.945627590191309 | | | | 9.20690092582877 | | | | | 3.18371864521141e-19 | | | | 3.6559875847666e-17 | | | | | 32.91996929 | | | | |
| -1.003563766 | | | | | -3.764323761 | | | | -9.205106359 | | | | | 3.2230825638357e-19 | | | | 3.69816931889143e-17 | | | | | 32.9078572174643 | | | | |
| 1.05649301264487 | | | | | 1.15861659670436 | | | | 9.20454276403751 | | | | | 3.23834038710706e-19 | | | | 3.71264543510449e-17 | | | | | 32.9032361067811 | | | | |
| 1.37910312373366 | | | | | 1.35005162510559 | | | | 9.20286163204587 | | | | | 3.28427855316364e-19 | | | | 3.76224324235945e-17 | | | | | 32.8894532601674 | | | | |
| 1.44081067609946 | | | | | 1.39816590388147 | | | | 9.20183731798515 | | | | | 3.31258434384569e-19 | | | | 3.7915782852047e-17 | | | | | 32.8810563536288 | | | | |
| -1.003108389 | | | | | -0.190944574 | | | | -9.237197036 | | | | | 3.4548737102786e-19 | | | | 3.93627829617179e-17 | | | | | 32.8507981414636 | | | | |
| 1.05567676120044 | | | | | -0.019505165 | | | | 9.19722315194858 | | | | | 3.44311524752743e-19 | | | | 3.92500245045929e-17 | | | | | 32.8432405589705 | | | | |
| 1.19101045785812 | | | | | 1.3458996361682 | | | | 9.19678343000454 | | | | | 3.48441119959371e-19 | | | | 3.96671617314439e-17 | | | | | 32.8321656911473 | | | | |
| -1.464075713 | | | | | -0.135281629 | | | | -9.221850422 | | | | | 3.59109420493821e-19 | | | | 4.07386788645761e-17 | | | | | 32.8115538790999 | | | | |
| 1.06951761711399 | | | | | 1.25636555160066 | | | | 9.19075448213926 | | | | | 3.63472149149013e-19 | | | | 4.11892773801839e-17 | | | | | 32.7902515043501 | | | | |
| -1.00610989 | | | | | -0.986026127 | | | | -9.189500528 | | | | | 3.67306764754289e-19 | | | | 4.16126393051964e-17 | | | | | 32.7799830023918 | | | | |
| 1.12124961472136 | | | | | -4.608223459 | | | | 9.18939533998185 | | | | | 3.68635939303995e-19 | | | | 4.17520055133805e-17 | | | | | 32.77653722 | | | | |
| 1.56449875365572 | | | | | -0.033136434 | | | | 9.18899863020484 | | | | | 3.69861652317441e-19 | | | | 4.18795818831083e-17 | | | | | 32.7732893039153 | | | | |
| -1.14680037 | | | | | 0.179484234094988 | | | | -9.189767099 | | | | | 3.88691502182269e-19 | | | | 4.3870328084903e-17 | | | | | 32.7286880790474 | | | | |
| 1.53516569667437 | | | | | 0.768350813170449 | | | | 9.19826503802515 | | | | | 3.92470149399546e-19 | | | | 4.42494352666596e-17 | | | | | 32.7211432472481 | | | | |
| -1.070729785 | | | | | -0.432047431 | | | | -9.182031646 | | | | | 3.94206539683062e-19 | | | | 4.44214515200847e-17 | | | | | 32.7117511336227 | | | | |
| -1.268719249 | | | | | -1.090053013 | | | | -9.18098337 | | | | | 3.99866516185053e-19 | | | | 4.49991229996667e-17 | | | | | 32.6973266273085 | | | | |
| -1.023165164 | | | | | 0.246820474129814 | | | | -9.218043601 | | | | | 4.2472943563604e-19 | | | | 4.76191600969553e-17 | | | | | 32.6477641560645 | | | | |
| -1.211105977 | | | | | -1.937852484 | | | | -9.172534341 | | | | | 4.23300624324614e-19 | | | | 4.74715888986596e-17 | | | | | 32.6411585731987 | | | | |
| 1.0082748666278 | | | | | 0.333146531433192 | | | | 9.17168956678358 | | | | | 4.26299428238638e-19 | | | | 4.77697794061617e-17 | | | | | 32.6342516360021 | | | | |
| -1.315318065 | | | | | 1.13619012243241 | | | | -9.249525527 | | | | | 4.33052913616266e-19 | | | | 4.84493027552589e-17 | | | | | 32.6153706131423 | | | | |
| -1.267543964 | | | | | -1.265635008 | | | | -9.1709952 | | | | | 4.35857339357145e-19 | | | | 4.872427473137e-17 | | | | | 32.6130937478831 | | | | |
| -1.014996218 | | | | | -0.96639708 | | | | -9.170109995 | | | | | 4.36689987362397e-19 | | | | 4.87914856839167e-17 | | | | | 32.6113854282487 | | | | |
| -1.06175097 | | | | | -1.191126532 | | | | -9.168880704 | | | | | 4.36422418821406e-19 | | | | 4.87745140059428e-17 | | | | | 32.611289811509 | | | | |
| -1.050296766 | | | | | 0.125580744844688 | | | | -9.167268882 | | | | | 4.43537747821457e-19 | | | | 4.94779264877972e-17 | | | | | 32.5955558230469 | | | | |
| 1.00135223737729 | | | | | 1.42937496763877 | | | | 9.16581306347873 | | | | | 4.47749987312522e-19 | | | | 4.99082043748535e-17 | | | | | 32.5862189578053 | | | | |
| 1.01684769487277 | | | | | 1.5805602557923 | | | | 9.16604662038099 | | | | | 4.49305789676794e-19 | | | | 5.00287225595495e-17 | | | | | 32.583677491721 | | | | |
| -1.308929084 | | | | | -0.011303224 | | | | -9.180017636 | | | | | 4.5675597284715e-19 | | | | 5.07376952240405e-17 | | | | | 32.5743972318602 | | | | |
| -1.274353446 | | | | | 0.59878028926316 | | | | -9.166438757 | | | | | 4.53999928413746e-19 | | | | 5.04581312109184e-17 | | | | | 32.5739484179044 | | | | |
| -1.214943607 | | | | | 0.0982063442643092 | | | | -9.163660785 | | | | | 4.55870726190269e-19 | | | | 5.06527033234917e-17 | | | | | 32.5686330744448 | | | | |
| -1.01778554 | | | | | 0.178765413734803 | | | | -9.160573676 | | | | | 4.67773790197396e-19 | | | | 5.17570648419145e-17 | | | | | 32.5434146134186 | | | | |
| 1.03794699395233 | | | | | 0.727783624096131 | | | | 9.15819657182344 | | | | | 4.78437446379783e-19 | | | | 5.2839894713191e-17 | | | | | 32.5214504222714 | | | | |
| -1.149174247 | | | | | 0.594981449292625 | | | | -9.159316921 | | | | | 4.91211495976782e-19 | | | | 5.41655730932348e-17 | | | | | 32.498957020468 | | | | |
| -1.179438289 | | | | | -0.138841612 | | | | -9.161567261 | | | | | 5.04577938525547e-19 | | | | 5.54654273561177e-17 | | | | | 32.4732879942459 | | | | |
| -1.073944582 | | | | | 0.432242931503044 | | | | -9.161986359 | | | | | 5.27283270908493e-19 | | | | 5.7765533084953e-17 | | | | | 32.432879751243 | | | | |
| -1.241919248 | | | | | -0.010468565 | | | | -9.145539161 | | | | | 5.4189715776725e-19 | | | | 5.91666945923657e-17 | | | | | 32.4005743629697 | | | | |
| 1.04764712630646 | | | | | 2.75055682121716 | | | | 9.14072201300175 | | | | | 5.5201366820924e-19 | | | | 6.02088989844258e-17 | | | | | 32.3814096027264 | | | | |
| 1.25449745398314 | | | | | 3.25614405105069 | | | | 9.14034393840598 | | | | | 5.53755752379101e-19 | | | | 6.03832914677258e-17 | | | | | 32.3783269484767 | | | | |
| -1.125447931 | | | | | 0.0321961871849957 | | | | -9.138916106 | | | | | 5.67972600756651e-19 | | | | 6.18056791125309e-17 | | | | | 32.3539761971703 | | | | |
| -1.244149986 | | | | | 0.454534828627274 | | | | -9.168154186 | | | | | 5.85262015760972e-19 | | | | 6.36050088108064e-17 | | | | | 32.3355263361415 | | | | |
| 1.02706709933987 | | | | | 2.57161092683332 | | | | 9.13558710456703 | | | | | 5.88708329321537e-19 | | | | 6.39301162052569e-17 | | | | | 32.3195216939428 | | | | |
| -1.118742485 | | | | | -0.785242998 | | | | -9.152831664 | | | | | 5.99530690201633e-19 | | | | 6.49548063045536e-17 | | | | | 32.3090509551098 | | | | |
| 1.14164078700876 | | | | | 0.102813062475048 | | | | 9.13208927223562 | | | | | 5.94762970301483e-19 | | | | 6.45211478484761e-17 | | | | | 32.3085254063041 | | | | |
| -1.043144205 | | | | | -1.518387918 | | | | -9.138749349 | | | | | 6.16994915322647e-19 | | | | 6.66006336936585e-17 | | | | | 32.2774951102035 | | | | |
| -1.051505355 | | | | | -0.666466537 | | | | -9.129959777 | | | | | 6.16926210406417e-19 | | | | 6.66006336936585e-17 | | | | | 32.2733656953719 | | | | |
| -1.174000726 | | | | | -0.623233749 | | | | -9.179290641 | | | | | 6.43344130509203e-19 | | | | 6.91685159387597e-17 | | | | | 32.2467317920547 | | | | |
| -1.00742961 | | | | | 0.163887419379625 | | | | -9.123853724 | | | | | 6.35273396075561e-19 | | | | 6.8387983896651e-17 | | | | | 32.2439723030386 | | | | |
| 1.22671542698623 | | | | | 1.72289772055316 | | | | 9.12923989173624 | | | | | 6.3793705484569e-19 | | | | 6.86396830611846e-17 | | | | | 32.2428521572698 | | | | |
| 1.54505663164106 | | | | | -0.60799297 | | | | 9.12409812524179 | | | | | 6.4599153712753e-19 | | | | 6.94354451849518e-17 | | | | | 32.2285976529891 | | | | |
| -1.28721705 | | | | | -0.431551264 | | | | -9.163210503 | | | | | 6.5439189337697e-19 | | | | 7.02130864326379e-17 | | | | | 32.2279358029443 | | | | |
| 1.03519457379506 | | | | | 1.87052216238062 | | | | 9.12650665222118 | | | | | 6.67419160386619e-19 | | | | 7.15562261276953e-17 | | | | | 32.1996831084841 | | | | |
| -1.367324502 | | | | | 1.41747461982792 | | | | -9.128694858 | | | | | 6.74543628315614e-19 | | | | 7.22649420101232e-17 | | | | | 32.1925467527583 | | | | |
| -1.138047776 | | | | | -0.369235034 | | | | -9.169336457 | | | | | 6.86860407401894e-19 | | | | 7.34351997944109e-17 | | | | | 32.1822679794242 | | | | |
| 1.4786380974717 | | | | | -0.684466511 | | | | 9.11439811324135 | | | | | 6.87261717077487e-19 | | | | 7.34594799087615e-17 | | | | | 32.1670200021808 | | | | |
| -1.053170463 | | | | | -0.530694568 | | | | -9.111650882 | | | | | 7.03140701939735e-19 | | | | 7.49477603101436e-17 | | | | | 32.1446742822683 | | | | |
| -1.048186151 | | | | | 0.058634951697782 | | | | -9.126374119 | | | | | 7.10340569336579e-19 | | | | 7.56769347832631e-17 | | | | | 32.1418553397181 | | | | |
| -1.259586998 | | | | | 0.519321529397509 | | | | -9.166900354 | | | | | 7.14710458797553e-19 | | | | 7.60464191675913e-17 | | | | | 32.1354508256637 | | | | |
| -1.287207004 | | | | | 3.23669732602361 | | | | -9.110050932 | | | | | 7.12555149681205e-19 | | | | 7.58553723721469e-17 | | | | | 32.1316629303543 | | | | |
| -1.155877417 | | | | | -0.359398602 | | | | -9.110266434 | | | | | 7.28594355999441e-19 | | | | 7.74455210724186e-17 | | | | | 32.1121059991614 | | | | |
| 1.47121947359159 | | | | | 1.07311695128759 | | | | 9.10796882712386 | | | | | 7.28841894667742e-19 | | | | 7.74523089023555e-17 | | | | | 32.1101083611419 | | | | |
| -1.091279906 | | | | | -0.566302175 | | | | -9.108368481 | | | | | 7.32253763600356e-19 | | | | 7.77756787147007e-17 | | | | | 32.1058088320798 | | | | |
| -1.059914503 | | | | | -0.998252062 | | | | -9.105732664 | | | | | 7.38592051247344e-19 | | | | 7.83502164149604e-17 | | | | | 32.0965543968514 | | | | |
| -1.012998019 | | | | | -0.534224484 | | | | -9.147696005 | | | | | 7.48614682259126e-19 | | | | 7.93305217779892e-17 | | | | | 32.0963826183983 | | | | |
| -1.387560975 | | | | | 1.58921818869674 | | | | -9.106964112 | | | | | 7.59081082256396e-19 | | | | 8.03619683542693e-17 | | | | | 32.0727774499083 | | | | |
| -1.169822609 | | | | | 0.869691066623887 | | | | -9.099727557 | | | | | 7.7841979015058e-19 | | | | 8.23266297749674e-17 | | | | | 32.0456450975333 | | | | |
| 1.34782425383846 | | | | | -0.066024978 | | | | 9.09791615271385 | | | | | 7.88138380070511e-19 | | | | 8.32743878617024e-17 | | | | | 32.0330382753519 | | | | |
| -1.103353391 | | | | | 1.10395159292966 | | | | -9.098150486 | | | | | 7.8868025752883e-19 | | | | 8.33073156794042e-17 | | | | | 32.0328357705385 | | | | |
| 1.00077245197454 | | | | | 0.758886289465663 | | | | 9.09732807535375 | | | | | 7.91996357916674e-19 | | | | 8.36366401809977e-17 | | | | | 32.028261391503 | | | | |
| -1.118101326 | | | | | -3.737774731 | | | | -9.096952333 | | | | | 7.96564837199856e-19 | | | | 8.4055929655171e-17 | | | | | 32.02272511 | | | | |
| -1.101296689 | | | | | 0.0178645181082799 | | | | -9.095651676 | | | | | 8.03096957005391e-19 | | | | 8.46816438760848e-17 | | | | | 32.014645551636 | | | | |
| -1.057371412 | | | | | -0.954103418 | | | | -9.112957392 | | | | | 8.30658700053411e-19 | | | | 8.7304051358804e-17 | | | | | 31.989823017988 | | | | |
| -1.020998493 | | | | | 0.49151793658482 | | | | -9.083803951 | | | | | 8.86061615255099e-19 | | | | 9.26191376560777e-17 | | | | | 31.918474853654 | | | | |
| -1.319883336 | | | | | 2.94083736967289 | | | | -9.083999721 | | | | | 8.86943171643132e-19 | | | | 9.26883090921337e-17 | | | | | 31.9179797454991 | | | | |
| 1.1445608515731 | | | | | 0.396343731100364 | | | | 9.08207685197365 | | | | | 9.01198395466039e-19 | | | | 9.39451983723522e-17 | | | | | 31.9023836662221 | | | | |
| 1.24370569285432 | | | | | -4.702250266 | | | | 9.08090147729702 | | | | | 9.07647059223371e-19 | | | | 9.45006260401775e-17 | | | | | 31.8949301483043 | | | | |
| 1.02920099512024 | | | | | 3.2223159070332 | | | | 9.07395385676167 | | | | | 9.61452482697404e-19 | | | | 9.95373602698292e-17 | | | | | 31.838595966284 | | | | |
| -1.238669037 | | | | | 0.113256463858174 | | | | -9.07394576 | | | | | 9.64027328703748e-19 | | | | 9.97304719564547e-17 | | | | | 31.8360705561299 | | | | |
| -1.416601517 | | | | | 0.655803841189437 | | | | -9.103941058 | | | | | 9.91967210235448e-19 | | | | 1.02495176069586e-16 | | | | | 31.8202371696906 | | | | |
| 1.04938478415096 | | | | | 3.76547400399643 | | | | 9.07155730752136 | | | | | 9.80735097230585e-19 | | | | 1.0135945305128e-16 | | | | | 31.8191717829641 | | | | |
| 1.21397855829534 | | | | | 1.68677230037826 | | | | 9.06574527002369 | | | | | 1.03447191514627e-18 | | | | 1.0654757510008e-16 | | | | | 31.7675696631681 | | | | |
| 1.14375738573276 | | | | | 2.63398781177317 | | | | 9.06413351927361 | | | | | 1.04292872322885e-18 | | | | 1.07313751762788e-16 | | | | | 31.7590276601389 | | | | |
| -1.161329853 | | | | | -2.0476759 | | | | -9.063633111 | | | | | 1.04998135551137e-18 | | | | 1.07960409212017e-16 | | | | | 31.752924060897 | | | | |
| -1.102387583 | | | | | -0.481056928 | | | | -9.067246521 | | | | | 1.05469288037446e-18 | | | | 1.08365581595394e-16 | | | | | 31.7504702490946 | | | | |
| -1.146786159 | | | | | -0.155920228 | | | | -9.065218384 | | | | | 1.06111688943672e-18 | | | | 1.08919464159878e-16 | | | | | 31.7445301690279 | | | | |
| -1.305818497 | | | | | 0.368496986875294 | | | | -9.068700412 | | | | | 1.07980368474892e-18 | | | | 1.1070284944033e-16 | | | | | 31.7302400459558 | | | | |
| -1.064867173 | | | | | -0.070891696 | | | | -9.059500461 | | | | | 1.09218528834748e-18 | | | | 1.11863437099218e-16 | | | | | 31.7145596221596 | | | | |
| 1.13833296436325 | | | | | 0.453422888657225 | | | | 9.0571968741159 | | | | | 1.10742554875338e-18 | | | | 1.13314275938567e-16 | | | | | 31.7008251694306 | | | | |
| -1.061150636 | | | | | -1.136716334 | | | | -9.055829433 | | | | | 1.11712981493072e-18 | | | | 1.14224085611502e-16 | | | | | 31.6917986392951 | | | | |
| 1.04817784280487 | | | | | 2.99286502401332 | | | | 9.05434063601988 | | | | | 1.13097461507311e-18 | | | | 1.15555625378696e-16 | | | | | 31.6797507232002 | | | | |
| -1.127980978 | | | | | 0.583142387046804 | | | | -9.052941116 | | | | | 1.1441439555025e-18 | | | | 1.16675013716261e-16 | | | | | 31.6684267290226 | | | | |
| -1.239745102 | | | | | -0.064130639 | | | | -9.083832047 | | | | | 1.1656226080301e-18 | | | | 1.18578548884935e-16 | | | | | 31.6629046693193 | | | | |
| 1.06349193048141 | | | | | 2.30592113630449 | | | | 9.04964692833027 | | | | | 1.17574389335774e-18 | | | | 1.19492872381817e-16 | | | | | 31.6417778735248 | | | | |
| -1.005290157 | | | | | -0.212347649 | | | | -9.048640915 | | | | | 1.19170770770643e-18 | | | | 1.20940406524325e-16 | | | | | 31.6295693591346 | | | | |
| 1.15959555782431 | | | | | -0.385265588 | | | | 9.05116688284518 | | | | | 1.20131499534987e-18 | | | | 1.21827439655887e-16 | | | | | 31.6227121236754 | | | | |
| -1.209400561 | | | | | 0.566478357342531 | | | | -9.050645055 | | | | | 1.20972410250201e-18 | | | | 1.22591771762081e-16 | | | | | 31.6163553327043 | | | | |
| -1.39403848 | | | | | 1.42113289835796 | | | | -9.04301174 | | | | | 1.24203763321457e-18 | | | | 1.25654992513817e-16 | | | | | 31.5881251405154 | | | | |
| 1.49242482566099 | | | | | -1.324489468 | | | | 9.04258027859288 | | | | | 1.24968724804814e-18 | | | | 1.26368254648551e-16 | | | | | 31.5822107893767 | | | | |
| -1.202822892 | | | | | -0.299099141 | | | | -9.040815302 | | | | | 1.26478735445789e-18 | | | | 1.27528188367828e-16 | | | | | 31.5703715135093 | | | | |
| 1.45266838003508 | | | | | -0.143185299 | | | | 9.11458190196458 | | | | | 1.37107088238626e-18 | | | | 1.3735791375049e-16 | | | | | 31.5075172134912 | | | | |
| -1.666951871 | | | | | 0.273687821870014 | | | | -9.050424488 | | | | | 1.36826820214674e-18 | | | | 1.37142299215406e-16 | | | | | 31.5028807133234 | | | | |
| -1.096011064 | | | | | 0.153848996817177 | | | | -9.044705583 | | | | | 1.38447308483393e-18 | | | | 1.38568897622104e-16 | | | | | 31.4894421106441 | | | | |
| -1.060405532 | | | | | 0.402413268600592 | | | | -9.064232957 | | | | | 1.43924554375836e-18 | | | | 1.43234993730608e-16 | | | | | 31.4568031090851 | | | | |
| -1.029082535 | | | | | -1.046778443 | | | | -9.191621884 | | | | | 1.43257823224936e-18 | | | | 1.42739901510536e-16 | | | | | 31.4484433064253 | | | | |
| -1.111426673 | | | | | 0.235728598533613 | | | | -9.029955573 | | | | | 1.44244654643784e-18 | | | | 1.43466769068567e-16 | | | | | 31.4462309207921 | | | | |
| -1.2839166 | | | | | 0.941016629518551 | | | | -9.034036837 | | | | | 1.452759154565e-18 | | | | 1.44375430757819e-16 | | | | | 31.4408765290316 | | | | |
| 1.05912144371229 | | | | | 0.00613198341080395 | | | | 9.02350793359632 | | | | | 1.45908014306186e-18 | | | | 1.44935294210812e-16 | | | | | 31.4305988548474 | | | | |
| -1.0763139 | | | | | -0.658858241 | | | | -9.026331853 | | | | | 1.46665465685562e-18 | | | | 1.45612782215707e-16 | | | | | 31.4277515734422 | | | | |
| 1.01411345406985 | | | | | 3.56815293014291 | | | | 9.02288834260778 | | | | | 1.46655778422539e-18 | | | | 1.45612782215707e-16 | | | | | 31.4255990929049 | | | | |
| -1.385188424 | | | | | 1.30828069020227 | | | | -9.021462438 | | | | | 1.50695141372008e-18 | | | | 1.49373813498671e-16 | | | | | 31.4007963508179 | | | | |
| -1.148540513 | | | | | -0.362100359 | | | | -9.025643899 | | | | | 1.60520315310867e-18 | | | | 1.58628079112101e-16 | | | | | 31.3443267752631 | | | | |
| -1.45950208 | | | | | 0.744650042652432 | | | | -9.044447771 | | | | | 1.64570239686154e-18 | | | | 1.62325925072422e-16 | | | | | 31.3269145124697 | | | | |
| 1.02390390987664 | | | | | 3.62695476072563 | | | | 9.00997417321642 | | | | | 1.63133902085597e-18 | | | | 1.6105987471888e-16 | | | | | 31.3214516960944 | | | | |
| -1.577098636 | | | | | 0.206669134801231 | | | | -9.00752605 | | | | | 1.68594181384725e-18 | | | | 1.65868195204146e-16 | | | | | 31.2905522179497 | | | | |
| -1.218619083 | | | | | -0.0283584 | | | | -9.00770652 | | | | | 1.70089347034733e-18 | | | | 1.6714419707326e-16 | | | | | 31.2830752221876 | | | | |
| -1.296436234 | | | | | 1.83788060004042 | | | | -9.008486407 | | | | | 1.7077159026141e-18 | | | | 1.67775527645687e-16 | | | | | 31.2816021318519 | | | | |
| -1.014718017 | | | | | 0.371516036176985 | | | | -9.002393988 | | | | | 1.73645814043883e-18 | | | | 1.70361168934119e-16 | | | | | 31.2603766152553 | | | | |
| -1.604844659 | | | | | 1.31879002671118 | | | | -9.013091902 | | | | | 1.76430382532985e-18 | | | | 1.73012556750428e-16 | | | | | 31.2525198755689 | | | | |
| -1.298163058 | | | | | 0.943705115446353 | | | | -9.002907957 | | | | | 1.76939267788832e-18 | | | | 1.73471241684531e-16 | | | | | 31.2444710213245 | | | | |
| -1.299661711 | | | | | -0.529681473 | | | | -9.144846908 | | | | | 1.79221976043272e-18 | | | | 1.75382990573606e-16 | | | | | 31.2342386605174 | | | | |
| -1.033517568 | | | | | -0.262921757 | | | | -8.996632806 | | | | | 1.82541508662546e-18 | | | | 1.78300389061236e-16 | | | | | 31.2120400638936 | | | | |
| 1.09450731160651 | | | | | 1.42089751535775 | | | | 8.99592852684839 | | | | | 1.83139508220559e-18 | | | | 1.78801658789913e-16 | | | | | 31.2083158156542 | | | | |
| -1.8235221 | | | | | 0.58028914312445 | | | | -9.193610982 | | | | | 1.78666661152795e-18 | | | | 1.74920759248431e-16 | | | | | 31.1597442502982 | | | | |
| -1.015906661 | | | | | -0.25967646 | | | | -8.991490972 | | | | | 1.94353673533718e-18 | | | | 1.88754670937434e-16 | | | | | 31.1518783601185 | | | | |
| -1.645452801 | | | | | 0.41959084470702 | | | | -8.995016839 | | | | | 1.96917644748434e-18 | | | | 1.90927255141578e-16 | | | | | 31.1405589767777 | | | | |
| 1.17039977569068 | | | | | 2.21816438262744 | | | | 8.98396740349615 | | | | | 2.02076370799956e-18 | | | | 1.95479567045693e-16 | | | | | 31.1120826589875 | | | | |
| -1.194490241 | | | | | -0.349930367 | | | | -8.985662033 | | | | | 2.03887922009147e-18 | | | | 1.96931524341727e-16 | | | | | 31.1062576396025 | | | | |
| -1.404562762 | | | | | 1.78796936125562 | | | | -8.98317775 | | | | | 2.03904246276942e-18 | | | | 1.96931524341727e-16 | | | | | 31.1033685356278 | | | | |
| -1.230705625 | | | | | -1.927011023 | | | | -8.98237435 | | | | | 2.04740454847784e-18 | | | | 1.97693857558198e-16 | | | | | 31.0992735479326 | | | | |
| 1.18180476912766 | | | | | 3.50226741586545 | | | | 8.97323411298331 | | | | | 2.20711180893463e-18 | | | | 2.12094994638438e-16 | | | | | 31.0258159267294 | | | | |
| -1.262199455 | | | | | -0.810635475 | | | | -8.972799174 | | | | | 2.2150112767722e-18 | | | | 2.12708677995112e-16 | | | | | 31.0223219432192 | | | | |
| -1.132500188 | | | | | -0.403015477 | | | | -8.978621745 | | | | | 2.28903923282549e-18 | | | | 2.19367993933074e-16 | | | | | 30.9961853049551 | | | | |
| -1.026287646 | | | | | -0.104552583 | | | | -8.967109749 | | | | | 2.33844304405517e-18 | | | | 2.23645107368279e-16 | | | | | 30.9704534746018 | | | | |
| 1.66496682988089 | | | | | -0.268133963 | | | | 8.96513250176325 | | | | | 2.35893747260915e-18 | | | | 2.25400671668956e-16 | | | | | 30.9607559093232 | | | | |
| -1.269828132 | | | | | -0.794542034 | | | | -8.992668218 | | | | | 2.41194835768535e-18 | | | | 2.29840963612471e-16 | | | | | 30.951916520238 | | | | |
| 1.8335228941618 | | | | | -2.098769822 | | | | 8.96540546629667 | | | | | 2.4015065788245e-18 | | | | 2.29104814274418e-16 | | | | | 30.9457319749523 | | | | |
| -1.252685048 | | | | | -0.074807349 | | | | -8.964804379 | | | | | 2.40117419706521e-18 | | | | 2.29104814274418e-16 | | | | | 30.9444063867263 | | | | |
| -1.330158519 | | | | | 2.28193386334754 | | | | -9.000991217 | | | | | 2.46418171208511e-18 | | | | 2.34288952093558e-16 | | | | | 30.9341568406291 | | | | |
| -1.102753306 | | | | | -1.25208911 | | | | -8.959173682 | | | | | 2.47715838710418e-18 | | | | 2.35363536973912e-16 | | | | | 30.912933861448 | | | | |
| -1.070024898 | | | | | -0.22323321 | | | | -8.958782402 | | | | | 2.51637651366672e-18 | | | | 2.38820725752385e-16 | | | | | 30.8993610282648 | | | | |
| 1.53951814701109 | | | | | -0.48335255 | | | | 8.95915311694812 | | | | | 2.57345387872472e-18 | | | | 2.43853549897045e-16 | | | | | 30.8791633945275 | | | | |
| -1.346266535 | | | | | -0.04229416 | | | | -8.953805878 | | | | | 2.58866128013458e-18 | | | | 2.4507427076658e-16 | | | | | 30.8698769423252 | | | | |
| -1.220451616 | | | | | 0.819751660872579 | | | | -8.950067345 | | | | | 2.67586261201308e-18 | | | | 2.52592564944606e-16 | | | | | 30.8375707575612 | | | | |
| -1.638447449 | | | | | 0.70100774921907 | | | | -8.950437728 | | | | | 2.68776139770853e-18 | | | | 2.53658985803885e-16 | | | | | 30.8339673218067 | | | | |
| 1.10093009798544 | | | | | 3.00519384216722 | | | | 8.94794458978087 | | | | | 2.71609090914728e-18 | | | | 2.56160602473749e-16 | | | | | 30.8228854246682 | | | | |
| -1.046463617 | | | | | 0.225743314270548 | | | | -8.947776377 | | | | | 2.7607991443658e-18 | | | | 2.60260714331484e-16 | | | | | 30.8083817770587 | | | | |
| 1.07032924662156 | | | | | 3.58702547742751 | | | | 8.94581068676069 | | | | | 2.76400853169413e-18 | | | | 2.60505024041006e-16 | | | | | 30.8057835249711 | | | | |
| 1.15350463098541 | | | | | 1.62821125438479 | | | | 8.94488997490609 | | | | | 2.78494085025997e-18 | | | | 2.62301989798776e-16 | | | | | 30.7984056122196 | | | | |
| 1.15349691441408 | | | | | -0.362470529 | | | | 8.9492125003452 | | | | | 2.79878424660685e-18 | | | | 2.63311770028271e-16 | | | | | 30.797202691344 | | | | |
| -1.205084683 | | | | | -2.677782202 | | | | -8.944125528 | | | | | 2.80243960346365e-18 | | | | 2.63596856478366e-16 | | | | | 30.7922803555485 | | | | |
| 1.08947327784915 | | | | | 1.02902668558748 | | | | 8.9430031870077 | | | | | 2.82832804453959e-18 | | | | 2.65617168494657e-16 | | | | | 30.7832881880947 | | | | |
| 1.05418619680312 | | | | | 1.2845415704945 | | | | 8.94209323463131 | | | | | 2.84949098784816e-18 | | | | 2.67425965912738e-16 | | | | | 30.7759983394127 | | | | |
| 1.09228232942613 | | | | | 3.60809420970367 | | | | 8.9403057741825 | | | | | 2.89151943622616e-18 | | | | 2.70888025032989e-16 | | | | | 30.7616803063141 | | | | |
| -1.288283293 | | | | | 0.122290242103679 | | | | -8.947973783 | | | | | 2.91732933932126e-18 | | | | 2.7324528265473e-16 | | | | | 30.7585184606258 | | | | |
| -1.166457884 | | | | | -0.420460679 | | | | -8.940793859 | | | | | 2.99824830634155e-18 | | | | 2.80077838576659e-16 | | | | | 30.7290740710839 | | | | |
| -1.038257375 | | | | | 0.558190265205708 | | | | -8.936461577 | | | | | 3.06703046479226e-18 | | | | 2.85911662796746e-16 | | | | | 30.7059975333867 | | | | |
| 1.15094822318994 | | | | | 0.169505775580365 | | | | 8.93253143345736 | | | | | 3.08156565762374e-18 | | | | 2.870976117654e-16 | | | | | 30.6994326834564 | | | | |
| -1.293665609 | | | | | -0.304742212 | | | | -8.932691269 | | | | | 3.08511861187758e-18 | | | | 2.873016707311e-16 | | | | | 30.6983992850555 | | | | |
| 1.69687670989079 | | | | | -0.318202362 | | | | 8.94012271051121 | | | | | 3.12697125283462e-18 | | | | 2.90621549081502e-16 | | | | | 30.693169582172 | | | | |
| 1.06987205427268 | | | | | 1.17584502655527 | | | | 8.93094855183429 | | | | | 3.16843297392437e-18 | | | | 2.94086095556832e-16 | | | | | 30.6732757567923 | | | | |
| -1.01140465 | | | | | -0.178730275 | | | | -8.932090153 | | | | | 3.28644966282163e-18 | | | | 3.04503895698087e-16 | | | | | 30.6401012737485 | | | | |
| 1.18327662124688 | | | | | 1.10010991525609 | | | | 8.92366307989107 | | | | | 3.31347652175957e-18 | | | | 3.0680580696758e-16 | | | | | 30.6284789697332 | | | | |
| -1.399397585 | | | | | 0.416687683009296 | | | | -8.960570835 | | | | | 3.40481935393317e-18 | | | | 3.14022348419291e-16 | | | | | 30.6186258253622 | | | | |
| 1.4437552710392 | | | | | 0.994437615461103 | | | | 8.92351133563234 | | | | | 3.36705185832413e-18 | | | | 3.11083426183071e-16 | | | | | 30.6142850547266 | | | | |
| -1.056603639 | | | | | 0.171879774609685 | | | | -8.925546525 | | | | | 3.37872061306847e-18 | | | | 3.12074491187528e-16 | | | | | 30.6125226558417 | | | | |
| -1.035329632 | | | | | 0.158480350916973 | | | | -8.920449449 | | | | | 3.41842501978021e-18 | | | | 3.15070443298518e-16 | | | | | 30.5981752809336 | | | | |
| 1.33662049799775 | | | | | -0.82831484 | | | | 8.91831590616047 | | | | | 3.46156454130607e-18 | | | | 3.18698235836797e-16 | | | | | 30.5857249729732 | | | | |
| 1.21246693803649 | | | | | 3.002538921 | | | | 8.91662231784784 | | | | | 3.50981903851002e-18 | | | | 3.22647785909858e-16 | | | | | 30.572187998171 | | | | |
| -1.466127504 | | | | | -1.112371234 | | | | -9.029290178 | | | | | 3.59354182340943e-18 | | | | 3.29625577697858e-16 | | | | | 30.5685840804729 | | | | |
| 1.0137284799377 | | | | | 2.97522766459018 | | | | 8.91590524849647 | | | | | 3.53045006564289e-18 | | | | 3.24261572463436e-16 | | | | | 30.5664570373225 | | | | |
| 1.10334717390696 | | | | | 1.33198673166434 | | | | 8.91295112357305 | | | | | 3.6167167155017e-18 | | | | 3.3146292923834e-16 | | | | | 30.542851016059 | | | | |
| -1.280941841 | | | | | -0.045666295 | | | | -8.969631421 | | | | | 3.80998013128518e-18 | | | | 3.48040364375871e-16 | | | | | 30.5106689533004 | | | | |
| -1.22086972 | | | | | -0.479068693 | | | | -8.929000806 | | | | | 3.81647338194452e-18 | | | | 3.4848253160774e-16 | | | | | 30.5028272753419 | | | | |
| 1.21826690649707 | | | | | 0.959597788140849 | | | | 8.90946483019428 | | | | | 3.77636271488226e-18 | | | | 3.45418409107246e-16 | | | | | 30.5011806366442 | | | | |
| -1.327401769 | | | | | 1.38599761651284 | | | | -8.926948099 | | | | | 3.83557647171182e-18 | | | | 3.50151013385305e-16 | | | | | 30.497607522384 | | | | |
| -1.27848954 | | | | | 0.329149942725397 | | | | -8.915403656 | | | | | 3.8655449354042e-18 | | | | 3.52581512635061e-16 | | | | | 30.4844007503126 | | | | |
| -1.680734017 | | | | | 0.371424452921991 | | | | -9.103100801 | | | | | 3.86020264898446e-18 | | | | 3.5224662432315e-16 | | | | | 30.4439639238051 | | | | |
| -1.019593093 | | | | | 0.771697839192331 | | | | -8.898853175 | | | | | 4.05799502671394e-18 | | | | 3.68779043731566e-16 | | | | | 30.4302833840628 | | | | |
| 1.20333743502455 | | | | | 1.39360997224499 | | | | 8.8972675589899 | | | | | 4.1208222165005e-18 | | | | 3.74004972886734e-16 | | | | | 30.4153549801216 | | | | |
| -1.157598319 | | | | | -0.464473452 | | | | -8.896410454 | | | | | 4.14973828028092e-18 | | | | 3.76142066929196e-16 | | | | | 30.4085177702949 | | | | |
| -1.324889698 | | | | | -0.431104586 | | | | -8.891527588 | | | | | 4.31834064662179e-18 | | | | 3.90335412746198e-16 | | | | | 30.3695768646675 | | | | |
| -1.015114898 | | | | | -0.100484761 | | | | -8.890617916 | | | | | 4.33998476275368e-18 | | | | 3.92207752874056e-16 | | | | | 30.3645942149932 | | | | |
| -1.178720217 | | | | | 0.0507545034738408 | | | | -8.908914856 | | | | | 4.4263578409261e-18 | | | | 3.98901968536719e-16 | | | | | 30.3584226567776 | | | | |
| -1.062261656 | | | | | -0.292259244 | | | | -8.895419538 | | | | | 4.40800594262163e-18 | | | | 3.97587992689896e-16 | | | | | 30.3549868662058 | | | | |
| 1.14558681156744 | | | | | 3.82966524312617 | | | | 8.88656601952748 | | | | | 4.48575807815586e-18 | | | | 4.03996071938484e-16 | | | | | 30.3322920402007 | | | | |
| -1.63464974 | | | | | -0.573153603 | | | | -9.149958442 | | | | | 4.38244133340345e-18 | | | | 3.95613044541947e-16 | | | | | 30.3142363175001 | | | | |
| -1.127951818 | | | | | -0.627987129 | | | | -8.884246502 | | | | | 4.59350446073876e-18 | | | | 4.12818206726282e-16 | | | | | 30.3092730149743 | | | | |
| -1.288193754 | | | | | 0.752844827021423 | | | | -8.889545259 | | | | | 4.68343526231036e-18 | | | | 4.20005135486688e-16 | | | | | 30.2966004318844 | | | | |
| 1.0076724681771 | | | | | 0.335755658708827 | | | | 8.87970634402563 | | | | | 4.7436648293053e-18 | | | | 4.24774081242974e-16 | | | | | 30.2776331292947 | | | | |
| -1.132013723 | | | | | 1.62069471807814 | | | | -8.878299277 | | | | | 4.79835238886637e-18 | | | | 4.29306440019793e-16 | | | | | 30.2664256369464 | | | | |
| -1.068420395 | | | | | 0.0862338898938805 | | | | -8.877892008 | | | | | 4.81429749062773e-18 | | | | 4.30550333589183e-16 | | | | | 30.2631819498357 | | | | |
| -1.32332913 | | | | | 1.30302546920343 | | | | -8.899904462 | | | | | 4.90111134678434e-18 | | | | 4.37757174666078e-16 | | | | | 30.2592523152531 | | | | |
| -1.034972488 | | | | | -1.069658109 | | | | -8.875172243 | | | | | 4.92213160731114e-18 | | | | 4.39262483567173e-16 | | | | | 30.2415235202339 | | | | |
| 1.3297989250817 | | | | | -1.028283438 | | | | 8.87448764226204 | | | | | 4.9496491167514e-18 | | | | 4.41624744617132e-16 | | | | | 30.2360726522108 | | | | |
| -1.086397357 | | | | | -0.312635176 | | | | -8.873833595 | | | | | 4.97608061612565e-18 | | | | 4.43420100042625e-16 | | | | | 30.2308653763236 | | | | |
| 1.13378534684597 | | | | | 3.87646856922562 | | | | 8.87363782514631 | | | | | 4.98401923249731e-18 | | | | 4.43846122442902e-16 | | | | | 30.2293067896073 | | | | |
| -1.312482466 | | | | | 1.22474434696714 | | | | -8.899539151 | | | | | 5.11015171479343e-18 | | | | 4.54119636159525e-16 | | | | | 30.2185890794177 | | | | |
| 1.1716200029206 | | | | | 3.02125016045176 | | | | 8.86839433834495 | | | | | 5.20137032319056e-18 | | | | 4.61739331406266e-16 | | | | | 30.1875721201839 | | | | |
| -1.157145361 | | | | | -0.14615036 | | | | -8.866552906 | | | | | 5.33089373507908e-18 | | | | 4.72144079241923e-16 | | | | | 30.1648922402356 | | | | |
| -1.314310444 | | | | | 0.543735276464624 | | | | -8.873381691 | | | | | 5.36785326992049e-18 | | | | 4.75186445356567e-16 | | | | | 30.1631202893229 | | | | |
| 1.01726224756354 | | | | | 1.47443670895674 | | | | 8.87210589217046 | | | | | 5.38208453671818e-18 | | | | 4.76277772633358e-16 | | | | | 30.1585592688876 | | | | |
| -1.308375336 | | | | | -3.540737473 | | | | -8.862826998 | | | | | 5.44241107964051e-18 | | | | 4.81212304456283e-16 | | | | | 30.1432816899084 | | | | |
| 1.21405700120518 | | | | | 0.766830975461221 | | | | 8.89949373277514 | | | | | 5.65946888457052e-18 | | | | 4.9911045440754e-16 | | | | | 30.1218648254192 | | | | |
| -1.769731092 | | | | | 0.22458338803768 | | | | -8.858930294 | | | | | 5.644568581324e-18 | | | | 4.97938333407299e-16 | | | | | 30.1078131098284 | | | | |
| -1.440051004 | | | | | 1.71114695058907 | | | | -8.868226365 | | | | | 5.79068571979925e-18 | | | | 5.09230124602241e-16 | | | | | 30.0923688988169 | | | | |
| -1.075113164 | | | | | -2.410010561 | | | | -8.856235466 | | | | | 5.74211037953151e-18 | | | | 5.05802315382714e-16 | | | | | 30.0908725642563 | | | | |
| 1.21694315270682 | | | | | 0.734740601567963 | | | | 8.85584309681902 | | | | | 5.76045631393529e-18 | | | | 5.07100545698767e-16 | | | | | 30.0877538446688 | | | | |
| 1.14998970462508 | | | | | 0.617207409867391 | | | | 8.85576791039112 | | | | | 5.76397840646011e-18 | | | | 5.07304691014826e-16 | | | | | 30.0871562430363 | | | | |
| -1.363205267 | | | | | -0.080511504 | | | | -8.878480666 | | | | | 5.87767514965236e-18 | | | | 5.16019004862359e-16 | | | | | 30.0812569108352 | | | | |
| -1.010856286 | | | | | 0.015574979664486 | | | | -8.855032206 | | | | | 5.82628396013686e-18 | | | | 5.1214699968124e-16 | | | | | 30.0773409215121 | | | | |
| -1.446064379 | | | | | 0.546793303425421 | | | | -8.923268159 | | | | | 6.04786059331333e-18 | | | | 5.29746858409313e-16 | | | | | 30.062804683698 | | | | |
| -1.139558527 | | | | | -3.048529493 | | | | -8.851521737 | | | | | 5.96638474742377e-18 | | | | 5.23262366149372e-16 | | | | | 30.0534132126209 | | | | |
| 1.00244346773033 | | | | | 0.714179665661474 | | | | 8.85142168623593 | | | | | 5.98545906102059e-18 | | | | 5.24826059941891e-16 | | | | | 30.0503874788222 | | | | |
| -1.204922035 | | | | | 0.412168199609858 | | | | -8.865147857 | | | | | 6.0987519227739e-18 | | | | 5.33871854531051e-16 | | | | | 30.0441842073288 | | | | |
| 1.0098257902741 | | | | | 1.49326519399782 | | | | 8.84524652462487 | | | | | 6.27844708466098e-18 | | | | 5.48236235698695e-16 | | | | | 30.003570087565 | | | | |
| -1.324685701 | | | | | 0.507077082143897 | | | | -8.934591802 | | | | | 6.3899202383527e-18 | | | | 5.5716246213941e-16 | | | | | 30.0014605444894 | | | | |
| -1.728293829 | | | | | 2.64875952332243 | | | | -8.845007433 | | | | | 6.30559277890718e-18 | | | | 5.50264757260304e-16 | | | | | 29.9999596270152 | | | | |
| -1.248897434 | | | | | 0.278111163775138 | | | | -8.846861431 | | | | | 6.39383292124916e-18 | | | | 5.57388366322748e-16 | | | | | 29.9894361259302 | | | | |
| -1.197283035 | | | | | -0.294269216 | | | | -8.843230546 | | | | | 6.47408370302354e-18 | | | | 5.63685086734243e-16 | | | | | 29.9756469009743 | | | | |
| -1.244557192 | | | | | 0.0239817731173875 | | | | -8.841205685 | | | | | 6.50326962506441e-18 | | | | 5.65875712505347e-16 | | | | | 29.9697832481485 | | | | |
| -1.196464617 | | | | | -0.61098529 | | | | -8.900425363 | | | | | 6.69428909728152e-18 | | | | 5.80580189973406e-16 | | | | | 29.963549515583 | | | | |
| 1.10037608068348 | | | | | 0.0677860047171032 | | | | 8.83899964823136 | | | | | 6.60512321040283e-18 | | | | 5.73791162779267e-16 | | | | | 29.9539805315483 | | | | |
| -1.299072242 | | | | | -0.830507758 | | | | -8.835899519 | | | | | 6.77342776171923e-18 | | | | 5.86599154710238e-16 | | | | | 29.9293813521023 | | | | |
| -1.004938682 | | | | | -0.035178279 | | | | -8.834912358 | | | | | 6.84405485788172e-18 | | | | 5.9222914260681e-16 | | | | | 29.9193353838807 | | | | |
| -1.588668644 | | | | | 0.747905822840182 | | | | -8.994955942 | | | | | 6.69641763926935e-18 | | | | 5.80645368281042e-16 | | | | | 29.9193137461591 | | | | |
| 1.1462672393448 | | | | | 0.436014309167892 | | | | 8.83351013630578 | | | | | 6.90603418049979e-18 | | | | 5.97224658099128e-16 | | | | | 29.910426643925 | | | | |
| -1.036140999 | | | | | 0.0246675893993874 | | | | -8.866924534 | | | | | 7.04524400030134e-18 | | | | 6.08389931928541e-16 | | | | | 29.9083739166165 | | | | |
| 1.50163810064419 | | | | | -2.207024447 | | | | 8.86067065066722 | | | | | 7.26967638580515e-18 | | | | 6.2560171693916e-16 | | | | | 29.8774406580187 | | | | |
| -1.072650548 | | | | | -0.213003733 | | | | -8.828908056 | | | | | 7.16871721872999e-18 | | | | 6.17787244966662e-16 | | | | | 29.8739305886446 | | | | |
| -1.325670208 | | | | | 0.474769553927615 | | | | -8.837050472 | | | | | 7.39209634045059e-18 | | | | 6.34961349333429e-16 | | | | | 29.8514138213596 | | | | |
| -1.378659275 | | | | | 0.971458465718293 | | | | -8.826918412 | | | | | 7.44291945201473e-18 | | | | 6.3906655372247e-16 | | | | | 29.8391213787939 | | | | |
| 1.12385203316808 | | | | | 1.14156019496021 | | | | 8.82227089757379 | | | | | 7.56505018591123e-18 | | | | 6.47969675379482e-16 | | | | | 29.821322891278 | | | | |
| -1.358126531 | | | | | 1.09473955185831 | | | | -8.821576713 | | | | | 7.66167449528765e-18 | | | | 6.553140536365e-16 | | | | | 29.809725233761 | | | | |
| -1.387549742 | | | | | 1.26809825622808 | | | | -8.826779857 | | | | | 7.71097188388245e-18 | | | | 6.59147659889862e-16 | | | | | 29.809092047382 | | | | |
| -1.252464586 | | | | | 2.58535341609042 | | | | -8.81991011 | | | | | 7.71118506471101e-18 | | | | 6.59147659889862e-16 | | | | | 29.8026184800038 | | | | |
| -1.030197163 | | | | | -0.763715015 | | | | -8.82439729 | | | | | 7.78348549762912e-18 | | | | 6.64654177761294e-16 | | | | | 29.7968410067291 | | | | |
| -1.278792422 | | | | | 0.44409757269744 | | | | -8.819980175 | | | | | 7.89195675735148e-18 | | | | 6.73098989860922e-16 | | | | | 29.7834338142359 | | | | |
| -1.360882847 | | | | | -0.210047681 | | | | -8.898452955 | | | | | 8.30198654970573e-18 | | | | 7.05359393192507e-16 | | | | | 29.757753407246 | | | | |
| -1.259788537 | | | | | 0.181700163442556 | | | | -8.860514026 | | | | | 8.30090885074823e-18 | | | | 7.05359393192507e-16 | | | | | 29.7516641882711 | | | | |
| -1.182399249 | | | | | 0.412428802629419 | | | | -8.884145822 | | | | | 8.45065906965682e-18 | | | | 7.16835528043088e-16 | | | | | 29.7301018321581 | | | | |
| 1.03281947354174 | | | | | 1.35542632002511 | | | | 8.81069105311694 | | | | | 8.30907714371167e-18 | | | | 7.05819613051753e-16 | | | | | 29.7296152787297 | | | | |
| 1.44780016544802 | | | | | 1.96360281600097 | | | | 8.80498023235753 | | | | | 8.7634768984699e-18 | | | | 7.41729286185829e-16 | | | | | 29.6783844498101 | | | | |
| -1.298688444 | | | | | 0.367613000250214 | | | | -8.824664201 | | | | | 8.98366680064664e-18 | | | | 7.58842704292602e-16 | | | | | 29.6682406676666 | | | | |
| 1.17558027893232 | | | | | -0.964584488 | | | | 8.80259723746003 | | | | | 8.91313914853051e-18 | | | | 7.53489050673789e-16 | | | | | 29.6617367356259 | | | | |
| 1.10813468446387 | | | | | 2.05351181717649 | | | | 8.80151835180117 | | | | | 9.01233589802954e-18 | | | | 7.60959487008834e-16 | | | | | 29.6510132433096 | | | | |
| -1.289345812 | | | | | -0.619401138 | | | | -8.951649858 | | | | | 9.04762707446708e-18 | | | | 7.63786368066173e-16 | | | | | 29.6477477155676 | | | | |
| 1.02146126945561 | | | | | -1.561103058 | | | | 8.79972674050501 | | | | | 9.31854852894939e-18 | | | | 7.85556249140761e-16 | | | | | 29.6206552695956 | | | | |
| 1.02688444958897 | | | | | 1.30081376395921 | | | | 8.7919044798074 | | | | | 9.67294892797227e-18 | | | | 8.12670327646556e-16 | | | | | 29.5810417267639 | | | | |
| -1.449893211 | | | | | 1.51709360966452 | | | | -8.839705125 | | | | | 9.93299653480012e-18 | | | | 8.32196830683324e-16 | | | | | 29.5761792310058 | | | | |
| -1.195841085 | | | | | -0.122717737 | | | | -8.801035148 | | | | | 9.90820538580481e-18 | | | | 8.30614903584953e-16 | | | | | 29.5676915970759 | | | | |
| -1.098133007 | | | | | -0.034825193 | | | | -8.787468845 | | | | | 1.00492312881669e-17 | | | | 8.40598959984391e-16 | | | | | 29.5438324472042 | | | | |
| -1.078428272 | | | | | 0.387433543678031 | | | | -8.787728636 | | | | | 1.00984283401531e-17 | | | | 8.44379194565215e-16 | | | | | 29.5398872693285 | | | | |
| -1.143800279 | | | | | 0.165337283157254 | | | | -8.848679509 | | | | | 1.06518068375992e-17 | | | | 8.8537565744509e-16 | | | | | 29.5094531457799 | | | | |
| -1.272577599 | | | | | 0.915366961860956 | | | | -8.782239375 | | | | | 1.05071044427739e-17 | | | | 8.7473459632467e-16 | | | | | 29.5003752180515 | | | | |
| 1.03790089433932 | | | | | 2.93363133658587 | | | | 8.78119878251781 | | | | | 1.05468598001472e-17 | | | | 8.77697724872328e-16 | | | | | 29.4964912003193 | | | | |
| -1.242435857 | | | | | 0.0602711245847418 | | | | -8.785426348 | | | | | 1.06357616861713e-17 | | | | 8.84223507533096e-16 | | | | | 29.49260825 | | | | |
| 1.05113746810669 | | | | | 3.03303133054547 | | | | 8.77826830882436 | | | | | 1.07993927312953e-17 | | | | 8.96236948042762e-16 | | | | | 29.473361781492 | | | | |
| 1.01815041004457 | | | | | 1.62349005383464 | | | | 8.77342319899936 | | | | | 1.12300969872206e-17 | | | | 9.29055325995944e-16 | | | | | 29.435134459785 | | | | |
| 1.09391840289975 | | | | | 3.02627332965938 | | | | 8.770041646 | | | | | 1.15672803223863e-17 | | | | 9.55076384088631e-16 | | | | | 29.4063138657234 | | | | |
| -1.694598333 | | | | | -0.14484297 | | | | -9.002781205 | | | | | 1.14930364182402e-17 | | | | 9.49318054158536e-16 | | | | | 29.4057956286061 | | | | |
| -1.277529592 | | | | | 0.451684797530804 | | | | -8.844972016 | | | | | 1.19466023386746e-17 | | | | 9.85045719231312e-16 | | | | | 29.3922075647914 | | | | |
| -1.234636154 | | | | | -1.444131319 | | | | -8.766168439 | | | | | 1.21579587763506e-17 | | | | 1.00032126384854e-15 | | | | | 29.3589574306453 | | | | |
| -1.006940977 | | | | | -0.064396874 | | | | -8.764652124 | | | | | 1.25126380834194e-17 | | | | 1.02649885226371e-15 | | | | | 29.3336218392483 | | | | |
| -1.294626193 | | | | | 2.0933827088655 | | | | -8.759659631 | | | | | 1.26351242531947e-17 | | | | 1.03574122158721e-15 | | | | | 29.3212994328509 | | | | |
| 1.1357733130643 | | | | | 0.295581083636412 | | | | 8.75318314742846 | | | | | 1.3220659938483e-17 | | | | 1.07828929908319e-15 | | | | | 29.275628915258 | | | | |
| -1.409342652 | | | | | 0.436686645586881 | | | | -8.779633376 | | | | | 1.3567101682627e-17 | | | | 1.10376997231591e-15 | | | | | 29.267159523739 | | | | |
| -1.347578934 | | | | | 0.223800968135783 | | | | -8.767280737 | | | | | 1.35819086427981e-17 | | | | 1.10476146169148e-15 | | | | | 29.2641525467994 | | | | |
| 1.11132428837797 | | | | | 0.738782114030804 | | | | 8.75105117606449 | | | | | 1.34496375777698e-17 | | | | 1.09484723502282e-15 | | | | | 29.2588449946076 | | | | |
| -1.055280274 | | | | | 0.528043450467411 | | | | -8.819736824 | | | | | 1.42654296403023e-17 | | | | 1.15501223433684e-15 | | | | | 29.2254093318957 | | | | |
| -1.22962704 | | | | | 1.59713766929912 | | | | -8.756804627 | | | | | 1.40995360010374e-17 | | | | 1.14333679722258e-15 | | | | | 29.2249080489236 | | | | |
| -1.38195214 | | | | | 0.00809657603365744 | | | | -8.763601907 | | | | | 1.42136826618509e-17 | | | | 1.15122401049495e-15 | | | | | 29.216735890611 | | | | |
| -1.302918133 | | | | | 0.0453290570850643 | | | | -8.763433796 | | | | | 1.46668612847122e-17 | | | | 1.18433079240226e-15 | | | | | 29.1888699945963 | | | | |
| 1.03424375298987 | | | | | 0.854615668475907 | | | | 8.7426667694802 | | | | | 1.45206713670387e-17 | | | | 1.17401269402098e-15 | | | | | 29.1854602673354 | | | | |
| -1.258988206 | | | | | 0.555004947386578 | | | | -8.790235592 | | | | | 1.5116972074554e-17 | | | | 1.2185764891373e-15 | | | | | 29.1670186272978 | | | | |
| -1.017066514 | | | | | -0.445828156 | | | | -8.735384731 | | | | | 1.53611960476146e-17 | | | | 1.2366085409312e-15 | | | | | 29.1292459407536 | | | | |
| -1.264583228 | | | | | 0.644564817462042 | | | | -8.738856084 | | | | | 1.55795355978721e-17 | | | | 1.25298927628357e-15 | | | | | 29.1214085861197 | | | | |
| 1.08564368983918 | | | | | 1.67360947574103 | | | | 8.73141417034717 | | | | | 1.57875051999634e-17 | | | | 1.26826392030264e-15 | | | | | 29.102867233827 | | | | |
| -1.080668438 | | | | | 0.548515592247509 | | | | -8.730136764 | | | | | 1.59144243473456e-17 | | | | 1.27700005985637e-15 | | | | | 29.0943733573321 | | | | |
| 1.02017806828668 | | | | | 0.64921388330463 | | | | 8.72764184847316 | | | | | 1.62367666073208e-17 | | | | 1.30063779926082e-15 | | | | | 29.0747746995436 | | | | |
| -1.074242818 | | | | | 2.17317491824615 | | | | -8.774418839 | | | | | 1.68344089834812e-17 | | | | 1.34467990832819e-15 | | | | | 29.0608634908836 | | | | |
| -1.449131913 | | | | | 0.00628301763808385 | | | | -8.766729467 | | | | | 1.72384321693548e-17 | | | | 1.37460848957107e-15 | | | | | 29.0388614992032 | | | | |
| -1.419107072 | | | | | 1.40306434709679 | | | | -8.72118114 | | | | | 1.71017974446772e-17 | | | | 1.3642290821977e-15 | | | | | 29.0240442443126 | | | | |
| -1.334545026 | | | | | 0.638465808374072 | | | | -8.853002933 | | | | | 1.6924874148827e-17 | | | | 1.35113814508442e-15 | | | | | 29.0189866037526 | | | | |
| -1.275980307 | | | | | -0.049043549 | | | | -8.734189305 | | | | | 1.75117519160551e-17 | | | | 1.39376754066496e-15 | | | | | 29.0102908737995 | | | | |
| -1.519027347 | | | | | 2.20230744041816 | | | | -8.719061712 | | | | | 1.74345547063877e-17 | | | | 1.38893419290431e-15 | | | | | 29.0058845360028 | | | | |
| -1.396627649 | | | | | 1.52147917880613 | | | | -8.739489125 | | | | | 1.78510836361321e-17 | | | | 1.41863295078172e-15 | | | | | 28.9976178288268 | | | | |
| -1.214336502 | | | | | 0.311933571279295 | | | | -8.717933095 | | | | | 1.86452618538638e-17 | | | | 1.47645963679225e-15 | | | | | 28.9432981764541 | | | | |
| -1.412612427 | | | | | -1.298318874 | | | | -8.740383748 | | | | | 1.90930678577147e-17 | | | | 1.50908602128633e-15 | | | | | 28.9352124585834 | | | | |
| -1.006700039 | | | | | -0.678855228 | | | | -8.730337571 | | | | | 1.94677570126629e-17 | | | | 1.53524208062127e-15 | | | | | 28.9137805332608 | | | | |
| -1.00473059 | | | | | -0.443615028 | | | | -8.702431963 | | | | | 1.9878558362576e-17 | | | | 1.56676480462569e-15 | | | | | 28.8769966307827 | | | | |
| -1.291859414 | | | | | -0.224633086 | | | | -8.732869134 | | | | | 2.05696047263574e-17 | | | | 1.61519277932274e-15 | | | | | 28.8634364642855 | | | | |
| -1.195307611 | | | | | 0.84389334245871 | | | | -8.700514507 | | | | | 2.01865057949096e-17 | | | | 1.58806789151857e-15 | | | | | 28.861972823981 | | | | |
| -1.086342728 | | | | | -1.932633259 | | | | -8.702308134 | | | | | 2.03064519176707e-17 | | | | 1.59601520598774e-15 | | | | | 28.858218658343 | | | | |
| 1.00589719757126 | | | | | -3.698352733 | | | | 8.6988942084556 | | | | | 2.04504021500583e-17 | | | | 1.60613169577483e-15 | | | | | 28.8492794367204 | | | | |
| -1.167834739 | | | | | 1.95985641670168 | | | | -8.717911259 | | | | | 2.13701838175248e-17 | | | | 1.67276135332016e-15 | | | | | 28.8213064607903 | | | | |
| -1.303353235 | | | | | -0.429615349 | | | | -8.749527172 | | | | | 2.17368287774051e-17 | | | | 1.69705028523762e-15 | | | | | 28.8149944192603 | | | | |
| -1.498340073 | | | | | 1.25745485104529 | | | | -8.700588553 | | | | | 2.15219592388743e-17 | | | | 1.68307947927599e-15 | | | | | 28.8079198456455 | | | | |
| -1.427130112 | | | | | 1.96171451083863 | | | | -8.692691609 | | | | | 2.16367815838716e-17 | | | | 1.69017815681199e-15 | | | | | 28.7956324428595 | | | | |
| -1.274338474 | | | | | -0.255738889 | | | | -8.699714238 | | | | | 2.2041874626676e-17 | | | | 1.71768569096848e-15 | | | | | 28.7833211811785 | | | | |
| -1.138732049 | | | | | -0.126708145 | | | | -8.689292735 | | | | | 2.21841058342286e-17 | | | | 1.72781158240103e-15 | | | | | 28.7705413301797 | | | | |
| -1.175316034 | | | | | 0.654253478989034 | | | | -8.686889034 | | | | | 2.25148538428398e-17 | | | | 1.7515580830791e-15 | | | | | 28.7552913446317 | | | | |
| -1.256493288 | | | | | 0.282354281323708 | | | | -8.685845786 | | | | | 2.27036946222157e-17 | | | | 1.76436913220599e-15 | | | | | 28.7471288065157 | | | | |
| 1.08141913458322 | | | | | 1.98901579830387 | | | | 8.68564930631006 | | | | | 2.27394346241068e-17 | | | | 1.76649546756579e-15 | | | | | 28.7455916104815 | | | | |
| 1.03178723827967 | | | | | 1.77718672399021 | | | | 8.6854884460362 | | | | | 2.29211078450466e-17 | | | | 1.77929741255906e-15 | | | | | 28.7387014917217 | | | | |
| 1.12953982857316 | | | | | 2.76591642762023 | | | | 8.68411735876535 | | | | | 2.31224135238956e-17 | | | | 1.79335321498894e-15 | | | | | 28.7300595289052 | | | | |
| 1.22100323995727 | | | | | -0.219302123 | | | | 8.68355729792137 | | | | | 2.31234411504135e-17 | | | | 1.79335321498894e-15 | | | | | 28.7292261213002 | | | | |
| -1.235763628 | | | | | 0.135233818427881 | | | | -8.698558334 | | | | | 2.35058918507159e-17 | | | | 1.82167421736654e-15 | | | | | 28.7231927698477 | | | | |
| 1.40128334083526 | | | | | -1.605031404 | | | | 8.67504652130521 | | | | | 2.49732666176809e-17 | | | | 1.92583711314512e-15 | | | | | 28.6561815573679 | | | | |
| 1.26590609427173 | | | | | -0.033271252 | | | | 8.67323032935148 | | | | | 2.51150145933524e-17 | | | | 1.93570612384918e-15 | | | | | 28.6484869475428 | | | | |
| 1.3251943685911 | | | | | 1.90675278943512 | | | | 8.67174170826972 | | | | | 2.54157445144626e-17 | | | | 1.95709587096666e-15 | | | | | 28.6368549682777 | | | | |
| 1.18477497871921 | | | | | -1.35110948 | | | | 8.66936338378408 | | | | | 2.59036143745708e-17 | | | | 1.98957688038712e-15 | | | | | 28.6182743008945 | | | | |
| -1.15735738 | | | | | 0.0374619396894626 | | | | -8.702501007 | | | | | 2.72860320033759e-17 | | | | 2.089666021588e-15 | | | | | 28.5900442683901 | | | | |
| 1.02552520716614 | | | | | 2.90610197908972 | | | | 8.66535844797627 | | | | | 2.67461582128231e-17 | | | | 2.05204740422145e-15 | | | | | 28.5869951552096 | | | | |
| 1.05871809580521 | | | | | 3.46147625957534 | | | | 8.66437614199517 | | | | | 2.69569151051653e-17 | | | | 2.06708899661667e-15 | | | | | 28.57932501 | | | | |
| 1.00090128232432 | | | | | 4.3450621035669 | | | | 8.66407489457261 | | | | | 2.70218770144917e-17 | | | | 2.07169361467286e-15 | | | | | 28.5769729155108 | | | | |
| -1.538361603 | | | | | 0.984772291855498 | | | | -8.66278926 | | | | | 2.73008590835338e-17 | | | | 2.09042187211798e-15 | | | | | 28.5669356419581 | | | | |
| 1.90036841685289 | | | | | -2.329851898 | | | | 8.66240819986199 | | | | | 2.75047801708361e-17 | | | | 2.10450746772573e-15 | | | | | 28.5598602119877 | | | | |
| -1.212527891 | | | | | 0.19783732024872 | | | | -8.74404599 | | | | | 2.81179879039462e-17 | | | | 2.14752978798352e-15 | | | | | 28.5585149490788 | | | | |
| 1.34253232102441 | | | | | -0.566905451 | | | | 8.66194076337831 | | | | | 2.7729903601064e-17 | | | | 2.12019373553231e-15 | | | | | 28.552695567437 | | | | |
| -1.118390962 | | | | | -0.643602606 | | | | -8.67104634 | | | | | 2.81750682977498e-17 | | | | 2.15149964670629e-15 | | | | | 28.5437250875115 | | | | |
| -1.174633737 | | | | | -0.208053785 | | | | -8.66049242 | | | | | 2.81146531878064e-17 | | | | 2.14752978798352e-15 | | | | | 28.5387258832921 | | | | |
| -1.341686943 | | | | | 0.51693595792669 | | | | -8.661019079 | | | | | 2.86329572505408e-17 | | | | 2.18290708440346e-15 | | | | | 28.5242134016028 | | | | |
| -1.65046782 | | | | | 0.0152415789223167 | | | | -8.66438983 | | | | | 2.87935388384296e-17 | | | | 2.19197897129456e-15 | | | | | 28.522282925802 | | | | |
| 1.13172044413202 | | | | | 1.20102307945484 | | | | 8.66104765581906 | | | | | 2.86917274250215e-17 | | | | 2.18580681179924e-15 | | | | | 28.5211561067177 | | | | |
| -1.17635028 | | | | | 2.8998638632145 | | | | -8.668175643 | | | | | 2.91104993897367e-17 | | | | 2.21291225485313e-15 | | | | | 28.5191107355895 | | | | |
| -1.021342943 | | | | | -0.092469469 | | | | -8.656300047 | | | | | 2.88161055035163e-17 | | | | 2.19286339781879e-15 | | | | | 28.5142493137944 | | | | |
| 1.56241381794784 | | | | | -1.430206904 | | | | 8.65645504238908 | | | | | 2.89073013688094e-17 | | | | 2.19905137437955e-15 | | | | | 28.5113588783503 | | | | |
| -1.278012457 | | | | | 0.0769858906358358 | | | | -8.655770324 | | | | | 2.90018148970696e-17 | | | | 2.20544549822314e-15 | | | | | 28.5080704229836 | | | | |
| -1.334442586 | | | | | -0.644068693 | | | | -8.680444559 | | | | | 3.01573141598072e-17 | | | | 2.28179340781731e-15 | | | | | 28.4861069925428 | | | | |
| 1.33936846194145 | | | | | 0.196229230774009 | | | | 8.65198945585304 | | | | | 2.97596531504018e-17 | | | | 2.25575282112708e-15 | | | | | 28.4826670288987 | | | | |
| 1.50842303391632 | | | | | -0.768205129 | | | | 8.65117391499023 | | | | | 2.99539766416707e-17 | | | | 2.26803615200095e-15 | | | | | 28.4763070304998 | | | | |
| -1.858181696 | | | | | 0.738918914678809 | | | | -8.888679312 | | | | | 2.71428401641049e-17 | | | | 2.08058924050138e-15 | | | | | 28.4697038680163 | | | | |
| -1.081178572 | | | | | 1.00291341 | | | | -8.654454813 | | | | | 3.03778554532604e-17 | | | | 2.29683168531044e-15 | | | | | 28.4678420553061 | | | | |
| -1.033325957 | | | | | -0.289766957 | | | | -8.649644021 | | | | | 3.03881990371276e-17 | | | | 2.29720184438609e-15 | | | | | 28.4629542143185 | | | | |
| 1.21833987840451 | | | | | -0.602958359 | | | | 8.64746279485902 | | | | | 3.08542045186746e-17 | | | | 2.32783904444257e-15 | | | | | 28.4473720494948 | | | | |
| 1.11590796521762 | | | | | -1.823121585 | | | | 8.64526800816074 | | | | | 3.13991373420251e-17 | | | | 2.36429897196638e-15 | | | | | 28.430264452774 | | | | |
| 1.25037870959612 | | | | | 2.57062654224088 | | | | 8.64475582193706 | | | | | 3.15276683464738e-17 | | | | 2.37228263948209e-15 | | | | | 28.4262726519192 | | | | |
| -1.282748099 | | | | | -0.283654872 | | | | -8.666884087 | | | | | 3.27320530205451e-17 | | | | 2.45371107505302e-15 | | | | | 28.4047952649524 | | | | |
| -1.239787929 | | | | | 0.328075644170418 | | | | -8.660372424 | | | | | 3.28348821483327e-17 | | | | 2.46098200417481e-15 | | | | | 28.3994452068618 | | | | |
| 1.26877624256693 | | | | | 2.31865105666265 | | | | 8.6408076 | | | | | 3.25360701308194e-17 | | | | 2.44075514891703e-15 | | | | | 28.3955080949822 | | | | |
| -1.067115461 | | | | | 0.188580440175241 | | | | -8.640045794 | | | | | 3.30213855287262e-17 | | | | 2.47364143469497e-15 | | | | | 28.3820506351571 | | | | |
| 1.14007892175869 | | | | | -1.581446713 | | | | 8.63901700560115 | | | | | 3.30038447155304e-17 | | | | 2.47276673795268e-15 | | | | | 28.3815595706155 | | | | |
| -1.991045091 | | | | | 0.369090377899998 | | | | -8.812404115 | | | | | 3.49981308924331e-17 | | | | 2.60643974804173e-15 | | | | | 28.3572647825681 | | | | |
| 1.0189791247346 | | | | | -0.073411234 | | | | 8.63079421184017 | | | | | 3.52387538740853e-17 | | | | 2.62302248316474e-15 | | | | | 28.3175353960815 | | | | |
| -1.409956582 | | | | | -0.19199278 | | | | -8.73093205 | | | | | 3.75154611761597e-17 | | | | 2.77870095101901e-15 | | | | | 28.2895932121181 | | | | |
| 1.03831095114162 | | | | | 3.81123426944623 | | | | 8.62644328184946 | | | | | 3.6481168835155e-17 | | | | 2.70827719414081e-15 | | | | | 28.2836784852189 | | | | |
| -1.081950699 | | | | | -0.274222217 | | | | -8.626370161 | | | | | 3.65024143101788e-17 | | | | 2.70937740576889e-15 | | | | | 28.2831096116933 | | | | |
| -1.192505188 | | | | | 0.460457163500737 | | | | -8.630440347 | | | | | 3.71951029421569e-17 | | | | 2.75642514193661e-15 | | | | | 28.2747769337254 | | | | |
| -1.284300068 | | | | | -0.155144484 | | | | -8.653554957 | | | | | 3.89258983949787e-17 | | | | 2.87559277789255e-15 | | | | | 28.2407155461181 | | | | |
| -1.073807461 | | | | | -0.624267168 | | | | -8.621712246 | | | | | 3.8294290215442e-17 | | | | 2.83091748862998e-15 | | | | | 28.2367810733113 | | | | |
| -1.074371151 | | | | | -0.605005954 | | | | -8.921365324 | | | | | 3.60430936030239e-17 | | | | 2.67952949216979e-15 | | | | | 28.2268314421266 | | | | |
| -1.223902281 | | | | | 0.543059793625793 | | | | -8.621935433 | | | | | 3.93447230287201e-17 | | | | 2.90246489844653e-15 | | | | | 28.2146460881337 | | | | |
| 1.35057845503859 | | | | | 1.31524547798109 | | | | 8.65416683824967 | | | | | 4.02741872699982e-17 | | | | 2.9642898492128e-15 | | | | | 28.2064615093927 | | | | |
| -1.44912932 | | | | | 1.11238896283246 | | | | -8.678766195 | | | | | 4.09456234900936e-17 | | | | 3.01003156503971e-15 | | | | | 28.1905111031846 | | | | |
| -1.409950667 | | | | | 1.37751217784052 | | | | -8.682553477 | | | | | 4.08196024972659e-17 | | | | 3.00181405389294e-15 | | | | | 28.1904497204547 | | | | |
| 1.09269951264897 | | | | | 3.37578063561627 | | | | 8.61000440238448 | | | | | 4.15786066465924e-17 | | | | 3.05443398339174e-15 | | | | | 28.1558852102031 | | | | |
| -1.161428962 | | | | | 0.383187219226503 | | | | -8.613815939 | | | | | 4.20588815686581e-17 | | | | 3.08740595809453e-15 | | | | | 28.1484075142313 | | | | |
| 1.07888604216343 | | | | | 1.6653389459848 | | | | 8.60548164302539 | | | | | 4.31004937706566e-17 | | | | 3.1574331494567e-15 | | | | | 28.1207610783578 | | | | |
| -1.115546945 | | | | | -0.91723238 | | | | -8.62425858 | | | | | 4.39078279703405e-17 | | | | 3.21099788766103e-15 | | | | | 28.1159717450012 | | | | |
| -1.396023847 | | | | | -0.127415635 | | | | -8.604645651 | | | | | 4.33877697127122e-17 | | | | 3.17492599895863e-15 | | | | | 28.1142703541345 | | | | |
| -1.163690881 | | | | | 0.704463511524954 | | | | -8.601678047 | | | | | 4.44229078899942e-17 | | | | 3.24585125107847e-15 | | | | | 28.0912337551785 | | | | |
| -1.27162569 | | | | | -0.030311839 | | | | -8.601298344 | | | | | 4.45571018741769e-17 | | | | 3.25452852022937e-15 | | | | | 28.0882867139913 | | | | |
| -1.098673006 | | | | | -1.261083376 | | | | -8.601864809 | | | | | 4.4739732711616e-17 | | | | 3.26617089551716e-15 | | | | | 28.0846883693807 | | | | |
| -1.37686378 | | | | | -0.366316199 | | | | -8.632014544 | | | | | 4.58354642991014e-17 | | | | 3.33404178557911e-15 | | | | | 28.0814540629852 | | | | |
| -1.0254394 | | | | | -2.076761957 | | | | -8.600005212 | | | | | 4.50171319165933e-17 | | | | 3.28528453016094e-15 | | | | | 28.0782509323096 | | | | |
| -1.046356815 | | | | | 0.362359959645896 | | | | -8.600448307 | | | | | 4.54418436711894e-17 | | | | 3.31169412734712e-15 | | | | | 28.0703078993456 | | | | |
| -1.19715629 | | | | | -0.003342747 | | | | -8.596404084 | | | | | 4.65215421059379e-17 | | | | 3.37928314553158e-15 | | | | | 28.046970729186 | | | | |
| -1.065576324 | | | | | 0.198562520453975 | | | | -8.59521003 | | | | | 4.84194604074322e-17 | | | | 3.50747876138154e-15 | | | | | 28.0123413737105 | | | | |
| 1.01650471869305 | | | | | 0.721166319681049 | | | | 8.59148482878955 | | | | | 4.8167820981459e-17 | | | | 3.4910493422571e-15 | | | | | 28.0121566565541 | | | | |
| -1.038223647 | | | | | -0.165489653 | | | | -8.591342977 | | | | | 4.82220782090088e-17 | | | | 3.49438111675421e-15 | | | | | 28.0110567405364 | | | | |
| -1.097095829 | | | | | 0.0969252031476188 | | | | -8.590032948 | | | | | 4.87260177159068e-17 | | | | 3.52786742621332e-15 | | | | | 28.0008995035428 | | | | |
| 1.1167146258525 | | | | | 0.343214243844358 | | | | 8.58963789199782 | | | | | 4.88790062204853e-17 | | | | 3.53772924012909e-15 | | | | | 27.9978367043248 | | | | |
| -1.048019855 | | | | | -1.006505756 | | | | -8.599397865 | | | | | 5.01979156176589e-17 | | | | 3.62820621846044e-15 | | | | | 27.9806612320429 | | | | |
| -1.001828539 | | | | | -0.142811753 | | | | -8.587116419 | | | | | 4.98667107307742e-17 | | | | 3.60550341458257e-15 | | | | | 27.9782909212009 | | | | |
| -1.406109497 | | | | | 0.349916438420132 | | | | -8.662317303 | | | | | 5.20582807265474e-17 | | | | 3.74834645538179e-15 | | | | | 27.9688366030437 | | | | |
| -1.009554975 | | | | | 0.0967226510525449 | | | | -8.586107663 | | | | | 5.037429712871e-17 | | | | 3.64008713387509e-15 | | | | | 27.9684958384075 | | | | |
| 1.03942152093426 | | | | | 4.55635154823601 | | | | 8.58582659027603 | | | | | 5.03795586911448e-17 | | | | 3.64008713387509e-15 | | | | | 27.9682943337248 | | | | |
| 1.22085543670425 | | | | | 1.62350966367334 | | | | 8.58340121627091 | | | | | 5.13580657398043e-17 | | | | 3.70253984963297e-15 | | | | | 27.9495002512219 | | | | |
| -1.294049867 | | | | | 1.16679901728288 | | | | -8.708915942 | | | | | 5.23654347361028e-17 | | | | 3.76614921800656e-15 | | | | | 27.9460979576328 | | | | |
| 1.37502659243329 | | | | | 2.51876158697515 | | | | 8.58410165569711 | | | | | 5.17324830554657e-17 | | | | 3.72825775593885e-15 | | | | | 27.9436449176884 | | | | |
| -1.250487224 | | | | | -0.23570313 | | | | -8.589685649 | | | | | 5.20864601860338e-17 | | | | 3.74864271490781e-15 | | | | | 27.9428966261224 | | | | |
| 1.00765144142837 | | | | | 1.02892970633204 | | | | 8.58002148954239 | | | | | 5.27529952175176e-17 | | | | 3.79271655099254e-15 | | | | | 27.923318221951 | | | | |
| 1.5804324688829 | | | | | 0.0947751700437491 | | | | 8.57899614673022 | | | | | 5.36378489288825e-17 | | | | 3.85240534114152e-15 | | | | | 27.9093843798938 | | | | |
| 1.02581022795292 | | | | | 1.17713879765265 | | | | 8.57657628360138 | | | | | 5.42134824117514e-17 | | | | 3.89043552221974e-15 | | | | | 27.8966376621779 | | | | |
| 1.46823105331576 | | | | | -0.363073813 | | | | 8.58065072213986 | | | | | 5.49422467611611e-17 | | | | 3.93804133805521e-15 | | | | | 27.8894080203627 | | | | |
| 1.93923674865834 | | | | | -1.733602127 | | | | 8.62898708804474 | | | | | 5.85385799592957e-17 | | | | 4.17805738175969e-15 | | | | | 27.8515458547793 | | | | |
| -1.398256849 | | | | | 1.00935153662539 | | | | -8.5682265 | | | | | 5.82894624859642e-17 | | | | 4.16239079533557e-15 | | | | | 27.8274131499027 | | | | |
| -1.562204621 | | | | | 1.47936605650959 | | | | -8.564788863 | | | | | 5.95191787073608e-17 | | | | 4.24301810406303e-15 | | | | | 27.8054194886795 | | | | |
| -1.061349737 | | | | | -0.779805454 | | | | -8.564667618 | | | | | 5.97019055454965e-17 | | | | 4.25460579877802e-15 | | | | | 27.802524621096 | | | | |
| -1.038272385 | | | | | 1.60328756206672 | | | | -8.564469317 | | | | | 6.03045264145492e-17 | | | | 4.29319764531875e-15 | | | | | 27.7944122603502 | | | | |
| -1.028283111 | | | | | -2.058991615 | | | | -8.562258172 | | | | | 6.0723513244227e-17 | | | | 4.32083763579028e-15 | | | | | 27.785848927165 | | | | |
| -1.286687607 | | | | | 0.319372093597774 | | | | -8.572506827 | | | | | 6.14758110918078e-17 | | | | 4.37068036808002e-15 | | | | | 27.781793392885 | | | | |
| -1.529351344 | | | | | 0.82871573612267 | | | | -8.560976381 | | | | | 6.23921431067429e-17 | | | | 4.42463756875552e-15 | | | | | 27.7621058161971 | | | | |
| 1.05388930600727 | | | | | -0.088943124 | | | | 8.57088513950292 | | | | | 6.28566083466409e-17 | | | | 4.45233429221032e-15 | | | | | 27.7616465147661 | | | | |
| 1.52482777128869 | | | | | 2.39127498021939 | | | | 8.55826783286818 | | | | | 6.267157743117e-17 | | | | 4.44071988832154e-15 | | | | | 27.75500015 | | | | |
| 1.08258017598186 | | | | | 0.684825191501203 | | | | 8.55768658094926 | | | | | 6.29604477914299e-17 | | | | 4.45819177501465e-15 | | | | | 27.7505075617091 | | | | |
| -1.002019683 | | | | | -0.162682354 | | | | -8.55718641 | | | | | 6.32100755535271e-17 | | | | 4.47361405818325e-15 | | | | | 27.7466418577723 | | | | |
| -1.19711196 | | | | | 0.778229214216491 | | | | -8.557766078 | | | | | 6.38592847831443e-17 | | | | 4.51501418754333e-15 | | | | | 27.7399408543003 | | | | |
| 1.20940919316783 | | | | | 3.35795301254722 | | | | 8.55503407368237 | | | | | 6.42954810264529e-17 | | | | 4.54324538214534e-15 | | | | | 27.7300090777248 | | | | |
| 1.0704021286816 | | | | | 2.91759853139786 | | | | 8.55609252278415 | | | | | 6.44350430469218e-17 | | | | 4.55190561259768e-15 | | | | | 27.7283929757518 | | | | |
| -1.229935324 | | | | | 0.475890147991927 | | | | -8.552299033 | | | | | 6.65373161116378e-17 | | | | 4.68941836616987e-15 | | | | | 27.6977872243142 | | | | |
| 1.07885551435496 | | | | | 3.02348993781317 | | | | 8.54998118881541 | | | | | 6.69165124232692e-17 | | | | 4.71220537633934e-15 | | | | | 27.6909750281105 | | | | |
| -1.101242844 | | | | | 1.46454412603426 | | | | -8.548417763 | | | | | 6.77486781074859e-17 | | | | 4.76523521227416e-15 | | | | | 27.6789012507777 | | | | |
| -1.259630221 | | | | | 0.419701431412831 | | | | -8.560795741 | | | | | 6.88663632937097e-17 | | | | 4.83578341549685e-15 | | | | | 27.6741086609875 | | | | |
| -1.092343593 | | | | | 0.257859228993909 | | | | -8.547890836 | | | | | 6.86047670562347e-17 | | | | 4.81982208007372e-15 | | | | | 27.6676969882142 | | | | |
| -1.506795113 | | | | | 0.267280219433347 | | | | -8.568765562 | | | | | 6.9714439502961e-17 | | | | 4.88557216307355e-15 | | | | | 27.6667484644785 | | | | |
| 1.00604137153687 | | | | | 0.642652341524483 | | | | 8.54602217692957 | | | | | 6.9043657742323e-17 | | | | 4.84581211055348e-15 | | | | | 27.6604045209317 | | | | |
| -1.258079973 | | | | | -0.272386202 | | | | -8.564984106 | | | | | 7.0350760962146e-17 | | | | 4.92328687117711e-15 | | | | | 27.6555615503945 | | | | |
| -1.151341982 | | | | | 0.138616321964043 | | | | -8.545143919 | | | | | 6.95245189533025e-17 | | | | 4.87469985732115e-15 | | | | | 27.6536244095601 | | | | |
| -1.270207916 | | | | | -0.326313441 | | | | -8.60834445 | | | | | 7.19748183381786e-17 | | | | 5.02976825466685e-15 | | | | | 27.6492633817603 | | | | |
| -1.378311934 | | | | | 0.217013799007749 | | | | -8.598954412 | | | | | 7.23429896467959e-17 | | | | 5.05400370036568e-15 | | | | | 27.6441132465695 | | | | |
| -1.427895862 | | | | | -0.572886729 | | | | -8.643950886 | | | | | 7.36745166001668e-17 | | | | 5.13385288514687e-15 | | | | | 27.6368849517792 | | | | |
| -1.273466504 | | | | | 0.0379621601713903 | | | | -8.544107974 | | | | | 7.12863887819929e-17 | | | | 4.98413721732764e-15 | | | | | 27.630643813583 | | | | |
| -1.547940979 | | | | | 2.17051491690784 | | | | -8.541497202 | | | | | 7.15568079149714e-17 | | | | 5.00221472040882e-15 | | | | | 27.6254780592924 | | | | |
| -1.121835537 | | | | | -1.617031555 | | | | -8.53955709 | | | | | 7.26618271590092e-17 | | | | 5.07105472660368e-15 | | | | | 27.6105077861901 | | | | |
| -1.224920719 | | | | | 0.00179435140201623 | | | | -8.536429148 | | | | | 7.51035311304882e-17 | | | | 5.22502985840505e-15 | | | | | 27.5792899533202 | | | | |
| 1.03244095806353 | | | | | -0.710716774 | | | | 8.53444135031647 | | | | | 7.58144563360273e-17 | | | | 5.27014207108682e-15 | | | | | 27.5691175275682 | | | | |
| -1.229002015 | | | | | -1.917131227 | | | | -8.535070249 | | | | | 7.5913269289607e-17 | | | | 5.27527163173181e-15 | | | | | 27.5681474728604 | | | | |
| -1.303256468 | | | | | -0.548728312 | | | | -8.595691177 | | | | | 8.26666275017806e-17 | | | | 5.70601355683022e-15 | | | | | 27.518129061151 | | | | |
| -1.021740876 | | | | | -1.702805325 | | | | -8.525996689 | | | | | 8.08704999956126e-17 | | | | 5.59302341039035e-15 | | | | | 27.5059513265733 | | | | |
| -1.160466187 | | | | | -0.072691829 | | | | -8.52492295 | | | | | 8.17272773533047e-17 | | | | 5.64949853140459e-15 | | | | | 27.4957571417561 | | | | |
| 1.50637881939601 | | | | | -0.337762217 | | | | 8.52391513531478 | | | | | 8.2379400114308e-17 | | | | 5.68805004850176e-15 | | | | | 27.4879936431764 | | | | |
| 1.05745913801596 | | | | | 0.776839749080724 | | | | 8.52267669510033 | | | | | 8.3360386514404e-17 | | | | 5.75107561870562e-15 | | | | | 27.4765308142749 | | | | |
| -1.014228496 | | | | | 0.788260101168758 | | | | -8.522860688 | | | | | 8.41132136706796e-17 | | | | 5.80111527780757e-15 | | | | | 27.4695918072097 | | | | |
| -1.147419263 | | | | | -0.581272531 | | | | -8.542368481 | | | | | 8.61604270212706e-17 | | | | 5.92969924303235e-15 | | | | | 27.4621450752095 | | | | |
| 1.19060430733117 | | | | | 2.88515783153332 | | | | 8.51987162766387 | | | | | 8.50479147839824e-17 | | | | 5.86174472490387e-15 | | | | | 27.4568528916902 | | | | |
| -1.034724856 | | | | | -0.496672176 | | | | -8.519331317 | | | | | 8.59441556510704e-17 | | | | 5.91867909740109e-15 | | | | | 27.4482610417752 | | | | |
| -1.197928324 | | | | | -0.084494735 | | | | -8.538549812 | | | | | 8.76399528802012e-17 | | | | 6.02267583621161e-15 | | | | | 27.4481591585884 | | | | |
| -1.044594757 | | | | | -0.988428738 | | | | -8.51874741 | | | | | 8.65206349648033e-17 | | | | 5.95157522766862e-15 | | | | | 27.4418310758683 | | | | |
| -1.108054654 | | | | | 0.87738821727787 | | | | -8.516412577 | | | | | 8.77608574775713e-17 | | | | 6.02901935036942e-15 | | | | | 27.4270595569166 | | | | |
| -1.059096804 | | | | | 0.274100248336869 | | | | -8.513995355 | | | | | 9.01962445546837e-17 | | | | 6.18122061455769e-15 | | | | | 27.4020601661276 | | | | |
| -1.086975587 | | | | | -0.977949545 | | | | -8.514067503 | | | | | 9.12929426942391e-17 | | | | 6.25028334890076e-15 | | | | | 27.392153175182 | | | | |
| -1.144976049 | | | | | -1.755664181 | | | | -8.510857676 | | | | | 9.11203603935728e-17 | | | | 6.23948073516691e-15 | | | | | 27.3893848730198 | | | | |
| -1.011701593 | | | | | 1.27357330464936 | | | | -8.516759204 | | | | | 9.17266321480384e-17 | | | | 6.27691804249649e-15 | | | | | 27.3887200922986 | | | | |
| -1.113347533 | | | | | -0.446876633 | | | | -8.517615561 | | | | | 9.315863663797e-17 | | | | 6.36561372729425e-15 | | | | | 27.378759669929 | | | | |
| -1.27897117 | | | | | 0.503650094723984 | | | | -8.550806171 | | | | | 9.49922521508278e-17 | | | | 6.4802003167657e-15 | | | | | 27.3762862367126 | | | | |
| -1.678233099 | | | | | 0.162115953300781 | | | | -8.519950634 | | | | | 9.33624832193164e-17 | | | | 6.37747583008086e-15 | | | | | 27.3740263226127 | | | | |
| -1.356329844 | | | | | 1.31266022767755 | | | | -8.508628784 | | | | | 9.27340769675871e-17 | | | | 6.34071318873398e-15 | | | | | 27.372237412682 | | | | |
| 1.02804860284597 | | | | | 3.86895225751827 | | | | 8.50800789599423 | | | | | 9.31886065271397e-17 | | | | 6.36662989538221e-15 | | | | | 27.367461411845 | | | | |
| -1.260181771 | | | | | -0.757824086 | | | | -8.499267005 | | | | | 9.98260437962673e-17 | | | | 6.78273411014696e-15 | | | | | 27.300255186344 | | | | |
| -1.143403755 | | | | | -0.878228441 | | | | -8.515131857 | | | | | 1.04441560828283e-16 | | | | 7.07013532741403e-15 | | | | | 27.2741334259646 | | | | |
| -1.261019375 | | | | | -0.326754952 | | | | -8.496030636 | | | | | 1.03886746340818e-16 | | | | 7.03596600217357e-15 | | | | | 27.263383851853 | | | | |
| 1.11087203088868 | | | | | 2.29464817638497 | | | | 8.49407048441033 | | | | | 1.03991222721588e-16 | | | | 7.04191085354293e-15 | | | | | 27.2603276314517 | | | | |
| -1.318714552 | | | | | 1.97139858223776 | | | | -8.493904002 | | | | | 1.05418879558564e-16 | | | | 7.13285926563858e-15 | | | | | 27.2496607441677 | | | | |
| 1.15260843370312 | | | | | 0.730208990897016 | | | | 8.49104586150613 | | | | | 1.06494130189654e-16 | | | | 7.1940691888932e-15 | | | | | 27.2370971660187 | | | | |
| -1.154298669 | | | | | -0.038609059 | | | | -8.490457001 | | | | | 1.06988295227946e-16 | | | | 7.22397989571947e-15 | | | | | 27.232575244967 | | | | |
| 1.27722426985094 | | | | | 0.33181588894366 | | | | 8.50444420891935 | | | | | 1.089261141867e-16 | | | | 7.34776428877072e-15 | | | | | 27.2284270644346 | | | | |
| 1.14754578047325 | | | | | -0.454327055 | | | | 8.48866826146599 | | | | | 1.0850331974795e-16 | | | | 7.32158662795935e-15 | | | | | 27.2188409207305 | | | | |
| 1.03973265510628 | | | | | 1.09563959979527 | | | | 8.48798148076061 | | | | | 1.09312994703704e-16 | | | | 7.37150335466352e-15 | | | | | 27.2123740380909 | | | | |
| -1.021727352 | | | | | -2.199268206 | | | | -8.487338318 | | | | | 1.09643473792294e-16 | | | | 7.39024354123986e-15 | | | | | 27.2086308771872 | | | | |
| 1.00782137710758 | | | | | 3.92515747815016 | | | | 8.48716767612402 | | | | | 1.09790618323841e-16 | | | | 7.39765198951674e-15 | | | | | 27.2073209471215 | | | | |
| -1.196699565 | | | | | 1.03912998174656 | | | | -8.487222699 | | | | | 1.10191416731758e-16 | | | | 7.41887509073611e-15 | | | | | 27.2046572821082 | | | | |
| 1.20404356184511 | | | | | 1.80696324508731 | | | | 8.48621390645205 | | | | | 1.10616654406834e-16 | | | | 7.44512763986105e-15 | | | | | 27.1999997439085 | | | | |
| 1.02310731986179 | | | | | 1.66184550041305 | | | | 8.48674811 | | | | | 1.12443861529422e-16 | | | | 7.551234765267e-15 | | | | | 27.1863857039803 | | | | |
| 1.02056014017423 | | | | | 0.987564628390288 | | | | 8.4812737839691 | | | | | 1.1499443892355e-16 | | | | 7.70534007006092e-15 | | | | | 27.1620898860115 | | | | |
| 1.18366245806707 | | | | | 0.388089216528391 | | | | 8.4760606270458 | | | | | 1.19799844074351e-16 | | | | 8.00316860754618e-15 | | | | | 27.1221045633279 | | | | |
| -1.180968471 | | | | | 0.867276409074579 | | | | -8.490930742 | | | | | 1.21917756167561e-16 | | | | 8.12534538377988e-15 | | | | | 27.1214098394272 | | | | |
| -1.158428657 | | | | | -1.38000907 | | | | -8.475352788 | | | | | 1.20467432193194e-16 | | | | 8.04139657003388e-15 | | | | | 27.1166769499027 | | | | |
| -1.142921492 | | | | | 0.632967055157088 | | | | -8.476247468 | | | | | 1.21345623818895e-16 | | | | 8.09361107176742e-15 | | | | | 27.1116792021841 | | | | |
| 1.6505319744448 | | | | | -0.589748461 | | | | 8.47408279691386 | | | | | 1.21674431906219e-16 | | | | 8.11169291729569e-15 | | | | | 27.1069397635968 | | | | |
| 1.21476189622373 | | | | | -1.972097063 | | | | 8.47380501737875 | | | | | 1.21940021735723e-16 | | | | 8.12554503244345e-15 | | | | | 27.104810152979 | | | | |
| -1.217176722 | | | | | 0.526373622301543 | | | | -8.475369461 | | | | | 1.22689514836029e-16 | | | | 8.16645425744412e-15 | | | | | 27.1011251856069 | | | | |
| -1.481416366 | | | | | -0.251728129 | | | | -8.471987259 | | | | | 1.23692203981491e-16 | | | | 8.2254047709944e-15 | | | | | 27.0908756446888 | | | | |
| -1.12918808 | | | | | -0.692176445 | | | | -8.471560454 | | | | | 1.24610670129919e-16 | | | | 8.27864838091744e-15 | | | | | 27.0838529862459 | | | | |
| -1.216877774 | | | | | -0.453714157 | | | | -8.564920075 | | | | | 1.32436881405125e-16 | | | | 8.73938337748026e-15 | | | | | 27.0634058365095 | | | | |
| -1.038583053 | | | | | -0.17328769 | | | | -8.468108643 | | | | | 1.28030989164415e-16 | | | | 8.48582634406774e-15 | | | | | 27.0581088087457 | | | | |
| -1.148119384 | | | | | -0.406541187 | | | | -8.549852393 | | | | | 1.33237314186045e-16 | | | | 8.78807731469258e-15 | | | | | 27.0557877353094 | | | | |
| -1.228933075 | | | | | 0.314046997471385 | | | | -8.467382457 | | | | | 1.28762272389456e-16 | | | | 8.5275934346571e-15 | | | | | 27.0518435494491 | | | | |
| 1.0066076114645 | | | | | 3.37213102484071 | | | | 8.46574942526991 | | | | | 1.29896268567895e-16 | | | | 8.59324750031761e-15 | | | | | 27.0430766406701 | | | | |
| -1.222696444 | | | | | -1.026012169 | | | | -8.464865095 | | | | | 1.30800336732644e-16 | | | | 8.64356338975927e-15 | | | | | 27.0363025888883 | | | | |
| 1.07743049182324 | | | | | 0.963250851254544 | | | | 8.466151377 | | | | | 1.31612250783915e-16 | | | | 8.69312935276524e-15 | | | | | 27.0317748941296 | | | | |
| -1.037737293 | | | | | -0.817287204 | | | | -8.463874858 | | | | | 1.31820054407628e-16 | | | | 8.70276535808117e-15 | | | | | 27.0287179746654 | | | | |
| 1.32087133738474 | | | | | 0.817826671535703 | | | | 8.46362672103644 | | | | | 1.32076807192919e-16 | | | | 8.71835117236037e-15 | | | | | 27.0268175086022 | | | | |
| -1.212051374 | | | | | 0.860985508197755 | | | | -8.463046842 | | | | | 1.32678748432014e-16 | | | | 8.75397400271119e-15 | | | | | 27.0223764316856 | | | | |
| -1.23793568 | | | | | 1.22550764114293 | | | | -8.477888019 | | | | | 1.3593776935116e-16 | | | | 8.95358937867908e-15 | | | | | 27.0155348892404 | | | | |
| -1.293165219 | | | | | 1.45595581422228 | | | | -8.482771311 | | | | | 1.37111732812935e-16 | | | | 9.02245698739549e-15 | | | | | 27.0120409604936 | | | | |
| 1.21451745919498 | | | | | 0.7949760336132 | | | | 8.45924514315761 | | | | | 1.36967907933439e-16 | | | | 9.01439952213098e-15 | | | | | 26.9914045171519 | | | | |
| -1.074430852 | | | | | -0.538866637 | | | | -8.472779165 | | | | | 1.39138342817966e-16 | | | | 9.15010371429378e-15 | | | | | 26.9911189193879 | | | | |
| 1.15080973334423 | | | | | 2.96979252092036 | | | | 8.4584755000751 | | | | | 1.3751987376108e-16 | | | | 9.04790219516842e-15 | | | | | 26.9873750641466 | | | | |
| -1.120772265 | | | | | 0.53256727209394 | | | | -8.458981054 | | | | | 1.39502472057959e-16 | | | | 9.16975953120493e-15 | | | | | 26.9764041523765 | | | | |
| -1.206355924 | | | | | 2.94517160307187 | | | | -8.457261008 | | | | | 1.39394796078544e-16 | | | | 9.16411033090736e-15 | | | | | 26.9750607798259 | | | | |
| 1.05096906781913 | | | | | 3.00686112935349 | | | | 8.45662915706613 | | | | | 1.39524288770053e-16 | | | | 9.16976415923756e-15 | | | | | 26.9732426102135 | | | | |
| 1.02367449539841 | | | | | 0.0695125567779968 | | | | 8.45583235536642 | | | | | 1.40398197355515e-16 | | | | 9.22001368617038e-15 | | | | | 26.967144442733 | | | | |
| -1.081647625 | | | | | 1.57974533456532 | | | | -8.511226738 | | | | | 1.44710782796406e-16 | | | | 9.47960164389628e-15 | | | | | 26.9651125755508 | | | | |
| 1.32222641617491 | | | | | -0.840611192 | | | | 8.45536085379935 | | | | | 1.40917870685648e-16 | | | | 9.24981919863281e-15 | | | | | 26.9635361204997 | | | | |
| -1.373651406 | | | | | 1.38996228919084 | | | | -8.454717993 | | | | | 1.43355903486118e-16 | | | | 9.40253287027397e-15 | | | | | 26.9508710747646 | | | | |
| -1.181336368 | | | | | -0.276792115 | | | | -8.455299955 | | | | | 1.43581291503422e-16 | | | | 9.41146015673062e-15 | | | | | 26.9482641821603 | | | | |
| 1.08033448226515 | | | | | 1.55065679067448 | | | | 8.45299992663987 | | | | | 1.43548722257269e-16 | | | | 9.4107882018381e-15 | | | | | 26.945470843387 | | | | |
| 1.02958362700653 | | | | | 0.742011003104003 | | | | 8.45565553495346 | | | | | 1.44968204084691e-16 | | | | 9.4949895334563e-15 | | | | | 26.939484372968 | | | | |
| -1.192597081 | | | | | 0.434634500388163 | | | | -8.448947507 | | | | | 1.48178039116719e-16 | | | | 9.69468328228811e-15 | | | | | 26.9144723721043 | | | | |
| -1.368881845 | | | | | 0.217606599773283 | | | | -8.449536315 | | | | | 1.48387228421499e-16 | | | | 9.70686357562727e-15 | | | | | 26.9141112187217 | | | | |
| -1.424607364 | | | | | 0.0573536879037924 | | | | -8.551091167 | | | | | 1.54706832719423e-16 | | | | 1.00874022193906e-14 | | | | | 26.9060002078028 | | | | |
| -1.25661207 | | | | | 2.38425644300162 | | | | -8.44770402 | | | | | 1.49627893655067e-16 | | | | 9.78195254535379e-15 | | | | | 26.9049629439087 | | | | |
| -1.116114414 | | | | | 0.497325356797596 | | | | -8.447287212 | | | | | 1.5041758313776e-16 | | | | 9.83205429882178e-15 | | | | | 26.8999238893537 | | | | |
| -1.088728465 | | | | | 0.312507617597443 | | | | -8.447138105 | | | | | 1.50593269460401e-16 | | | | 9.84201238892859e-15 | | | | | 26.8994977491939 | | | | |
| -1.046831915 | | | | | 3.18167207781099 | | | | -8.444466977 | | | | | 1.53468205775266e-16 | | | | 1.00112837761844e-14 | | | | | 26.8802134392938 | | | | |
| -1.045161711 | | | | | -1.160890171 | | | | -8.455534582 | | | | | 1.60630018128938e-16 | | | | 1.04445427516468e-14 | | | | | 26.8479796714409 | | | | |
| -1.14847755 | | | | | -0.982897036 | | | | -8.464094318 | | | | | 1.65133967006564e-16 | | | | 1.07027420638884e-14 | | | | | 26.8288559606891 | | | | |
| -1.060542283 | | | | | 0.055394852264156 | | | | -8.444067483 | | | | | 1.63469642022762e-16 | | | | 1.06111828743439e-14 | | | | | 26.8282341297615 | | | | |
| -1.022221695 | | | | | -1.495397764 | | | | -8.43752849 | | | | | 1.62030832655305e-16 | | | | 1.05242669756258e-14 | | | | | 26.8271901838431 | | | | |
| -1.263786703 | | | | | 0.145411256362862 | | | | -8.436261357 | | | | | 1.63970842825231e-16 | | | | 1.06371669683254e-14 | | | | | 26.8156686423437 | | | | |
| -1.267458965 | | | | | 0.82744930518689 | | | | -8.436143098 | | | | | 1.6412256105184e-16 | | | | 1.06453715303383e-14 | | | | | 26.8147654425812 | | | | |
| -1.183496347 | | | | | 2.25003318614671 | | | | -8.435447316 | | | | | 1.64689966386795e-16 | | | | 1.06788894550699e-14 | | | | | 26.8112930770128 | | | | |
| 1.74120508798742 | | | | | 0.895325177821674 | | | | 8.43601352812685 | | | | | 1.64946697230187e-16 | | | | 1.0692248089324e-14 | | | | | 26.8100783142141 | | | | |
| -1.036310448 | | | | | 0.804442551894505 | | | | -8.450043666 | | | | | 1.68030053839306e-16 | | | | 1.08703947226788e-14 | | | | | 26.8091554678182 | | | | |
| 1.01381963825396 | | | | | 2.46794903474319 | | | | 8.43439652073881 | | | | | 1.67045147558076e-16 | | | | 1.08116542395723e-14 | | | | | 26.7977325812625 | | | | |
| 1.39871342421266 | | | | | -1.846281036 | | | | 8.44348527636388 | | | | | 1.70071361222603e-16 | | | | 1.09839165089194e-14 | | | | | 26.78931783 | | | | |
| -1.222411178 | | | | | 1.60459404094659 | | | | -8.432470087 | | | | | 1.68569097680934e-16 | | | | 1.09002501792853e-14 | | | | | 26.7885570768137 | | | | |
| -1.004606509 | | | | | -0.584520519 | | | | -8.430879724 | | | | | 1.70678078447594e-16 | | | | 1.10180381719224e-14 | | | | | 26.776414774222 | | | | |
| 1.13114749161885 | | | | | -2.637881061 | | | | 8.4304850965086 | | | | | 1.71545691624437e-16 | | | | 1.10672692452993e-14 | | | | | 26.7715650639048 | | | | |
| -1.071158629 | | | | | -1.014157935 | | | | -8.430105432 | | | | | 1.7205550797405e-16 | | | | 1.10933709552626e-14 | | | | | 26.7693893801524 | | | | |
| -1.152212146 | | | | | 1.02596673214673 | | | | -8.432934484 | | | | | 1.73132329989127e-16 | | | | 1.11542719001551e-14 | | | | | 26.7688685865856 | | | | |
| -1.213799794 | | | | | -0.385524951 | | | | -8.427921365 | | | | | 1.75017493729039e-16 | | | | 1.12705599542951e-14 | | | | | 26.751998173134 | | | | |
| -1.56706371 | | | | | 1.1141195286055 | | | | -8.425629507 | | | | | 1.7817982770508e-16 | | | | 1.14462352145645e-14 | | | | | 26.7352349406978 | | | | |
| -1.280718037 | | | | | -0.254782205 | | | | -8.482029058 | | | | | 1.86589157342555e-16 | | | | 1.19373463778843e-14 | | | | | 26.7239334571798 | | | | |
| 1.39427650478947 | | | | | -1.095830948 | | | | 8.42177327767012 | | | | | 1.84359126847816e-16 | | | | 1.18054233892055e-14 | | | | | 26.7014223222257 | | | | |
| -1.321248757 | | | | | -3.791319083 | | | | -8.420891414 | | | | | 1.84532334003384e-16 | | | | 1.18147205100527e-14 | | | | | 26.7001981541532 | | | | |
| -1.384828331 | | | | | 0.893587316417997 | | | | -8.472541108 | | | | | 1.92098724381406e-16 | | | | 1.22545036474898e-14 | | | | | 26.6943166901831 | | | | |
| -1.270355772 | | | | | 0.461456832501011 | | | | -8.423864506 | | | | | 1.88148706507887e-16 | | | | 1.20230203585535e-14 | | | | | 26.6862370074712 | | | | |
| -1.060152415 | | | | | -0.501689398 | | | | -8.417925046 | | | | | 1.88856722285794e-16 | | | | 1.20622862899501e-14 | | | | | 26.6775774467496 | | | | |
| -1.678731768 | | | | | 0.581586274414675 | | | | -8.533952741 | | | | | 1.86436184366317e-16 | | | | 1.19293696300068e-14 | | | | | 26.6731738003296 | | | | |
| -1.511967814 | | | | | 0.747135138428988 | | | | -8.470528132 | | | | | 2.00985887628546e-16 | | | | 1.27750556581744e-14 | | | | | 26.6504276332425 | | | | |
| -1.36288797 | | | | | -0.089426907 | | | | -8.415975028 | | | | | 1.94831204178022e-16 | | | | 1.24194215972406e-14 | | | | | 26.6501416068312 | | | | |
| -1.003343359 | | | | | 0.585205048247794 | | | | -8.413074445 | | | | | 1.96920041141456e-16 | | | | 1.25393044017091e-14 | | | | | 26.6369541000336 | | | | |
| -1.197465296 | | | | | -0.17595779 | | | | -8.426806488 | | | | | 2.0125315156402e-16 | | | | 1.27901155116805e-14 | | | | | 26.629879474367 | | | | |
| -1.194373742 | | | | | 1.28209106212084 | | | | -8.449456205 | | | | | 2.10133989511903e-16 | | | | 1.33003901767464e-14 | | | | | 26.604752510492 | | | | |
| 1.00900845544704 | | | | | 0.469911207905671 | | | | 8.40691800921675 | | | | | 2.05795653786955e-16 | | | | 1.30532270355513e-14 | | | | | 26.5936984846385 | | | | |
| 1.10561014443478 | | | | | -0.574374553 | | | | 8.40788950295024 | | | | | 2.10035884332603e-16 | | | | 1.32961764519634e-14 | | | | | 26.5766754828463 | | | | |
| -1.094882963 | | | | | -1.082292049 | | | | -8.40691978 | | | | | 2.09923134452251e-16 | | | | 1.3291034249922e-14 | | | | | 26.5760671629227 | | | | |
| -1.131416434 | | | | | 2.05451597864111 | | | | -8.40332415 | | | | | 2.11644790954655e-16 | | | | 1.33872882079515e-14 | | | | | 26.5663312237508 | | | | |
| -1.345766367 | | | | | 0.726272836240107 | | | | -8.436201475 | | | | | 2.19060537907281e-16 | | | | 1.38218250814549e-14 | | | | | 26.5594319754187 | | | | |
| -1.245495643 | | | | | 2.42540342156203 | | | | -8.402206827 | | | | | 2.15610571260873e-16 | | | | 1.36224916966695e-14 | | | | | 26.5523301153264 | | | | |
| 1.17603131233515 | | | | | 1.24853714637106 | | | | 8.40069031222068 | | | | | 2.16035435430858e-16 | | | | 1.36452459643694e-14 | | | | | 26.5462806852805 | | | | |
| -1.140783377 | | | | | 0.649931154859636 | | | | -8.403021115 | | | | | 2.1770868044468e-16 | | | | 1.37407412579315e-14 | | | | | 26.5429659459104 | | | | |
| -1.07114131 | | | | | -0.099298163 | | | | -8.400607803 | | | | | 2.17024336426439e-16 | | | | 1.37015499237815e-14 | | | | | 26.5420261617787 | | | | |
| 1.29513227948144 | | | | | 2.31838147933141 | | | | 8.3990593076894 | | | | | 2.19658965356964e-16 | | | | 1.38575098626677e-14 | | | | | 26.5302431957602 | | | | |
| 1.26972020847194 | | | | | 0.603297460241776 | | | | 8.39437323438597 | | | | | 2.28723576596812e-16 | | | | 1.43970548654393e-14 | | | | | 26.4909627329486 | | | | |
| 1.07901390665018 | | | | | 0.415217562520599 | | | | 8.39317827397858 | | | | | 2.29506204304632e-16 | | | | 1.44398509652543e-14 | | | | | 26.4873180841604 | | | | |
| -1.377963123 | | | | | 0.703121196551879 | | | | -8.393413245 | | | | | 2.30894705663882e-16 | | | | 1.45207114895286e-14 | | | | | 26.4833097696048 | | | | |
| 1.03869186726731 | | | | | 1.96684938751075 | | | | 8.39126183838535 | | | | | 2.32503036859643e-16 | | | | 1.46066083957994e-14 | | | | | 26.4745475326824 | | | | |
| -1.225051916 | | | | | 1.09015325926948 | | | | -8.390755156 | | | | | 2.33422277779218e-16 | | | | 1.46556241618765e-14 | | | | | 26.4706945122276 | | | | |
| -1.072166976 | | | | | 0.901611953327692 | | | | -8.390116079 | | | | | 2.34586834590252e-16 | | | | 1.47243570321036e-14 | | | | | 26.4658349824528 | | | | |
| -1.121601834 | | | | | 0.18085569872419 | | | | -8.390116222 | | | | | 2.35966664721005e-16 | | | | 1.47999499498596e-14 | | | | | 26.4604169114543 | | | | |
| -1.239503702 | | | | | 0.312962643860405 | | | | -8.451155758 | | | | | 2.45266085338844e-16 | | | | 1.53466909340897e-14 | | | | | 26.4591110919138 | | | | |
| -1.260546436 | | | | | -0.054982387 | | | | -8.388744668 | | | | | 2.38499348672242e-16 | | | | 1.49410221890692e-14 | | | | | 26.4499922477587 | | | | |
| -1.345046085 | | | | | 3.19776873746084 | | | | -8.384311942 | | | | | 2.47354079638114e-16 | | | | 1.54612799823604e-14 | | | | | 26.4152421216207 | | | | |
| 1.1029200518286 | | | | | 1.12163801195897 | | | | 8.38554548503611 | | | | | 2.47916915255007e-16 | | | | 1.54918680580289e-14 | | | | | 26.4143735905668 | | | | |
| -1.042010013 | | | | | -0.344408685 | | | | -8.390357731 | | | | | 2.50378302442717e-16 | | | | 1.56364068114663e-14 | | | | | 26.4085571762534 | | | | |
| -1.55835699 | | | | | 1.89305776490159 | | | | -8.382664256 | | | | | 2.50544740143784e-16 | | | | 1.56444840184258e-14 | | | | | 26.4034666889995 | | | | |
| -1.023357165 | | | | | -0.506321766 | | | | -8.385884182 | | | | | 2.5230718147706e-16 | | | | 1.57428779540444e-14 | | | | | 26.3989937586053 | | | | |
| -1.175471228 | | | | | 1.3631036240267 | | | | -8.379944588 | | | | | 2.55900392884909e-16 | | | | 1.59364227836035e-14 | | | | | 26.3820773993516 | | | | |
| 1.0148651405373 | | | | | -0.28950469 | | | | 8.37544380360804 | | | | | 2.62959109485156e-16 | | | | 1.63253780472034e-14 | | | | | 26.3543517392334 | | | | |
| -1.004997663 | | | | | -0.062340101 | | | | -8.373578852 | | | | | 2.67836402750749e-16 | | | | 1.66110569124737e-14 | | | | | 26.33736248 | | | | |
| 1.10411947812068 | | | | | 3.39137866563416 | | | | 8.37295365446605 | | | | | 2.68099919387354e-16 | | | | 1.66249548541273e-14 | | | | | 26.3354470928691 | | | | |
| -1.593626447 | | | | | -0.155359131 | | | | -8.509929629 | | | | | 2.78422206483384e-16 | | | | 1.72043217772346e-14 | | | | | 26.3343961567044 | | | | |
| -1.10164851 | | | | | -0.390412847 | | | | -8.380088234 | | | | | 2.73438278251939e-16 | | | | 1.69324443305472e-14 | | | | | 26.3257226472065 | | | | |
| -1.238822284 | | | | | 0.783831863564048 | | | | -8.37123448 | | | | | 2.74360469763344e-16 | | | | 1.69807103016453e-14 | | | | | 26.3141731058903 | | | | |
| -1.082930026 | | | | | 0.992837347186689 | | | | -8.377045167 | | | | | 2.79959430548578e-16 | | | | 1.72942415879606e-14 | | | | | 26.3053568661183 | | | | |
| 1.03658521507193 | | | | | -4.879900802 | | | | 8.36882991466203 | | | | | 2.76832356675904e-16 | | | | 1.71186244082018e-14 | | | | | 26.3041508554831 | | | | |
| -1.123052483 | | | | | -0.924613749 | | | | -8.406174926 | | | | | 2.86095325596116e-16 | | | | 1.76242243892059e-14 | | | | | 26.3013946171504 | | | | |
| 1.27157233192855 | | | | | 2.12826994936156 | | | | 8.36891029646724 | | | | | 2.80455189120475e-16 | | | | 1.73174073067121e-14 | | | | | 26.2929284984303 | | | | |
| 1.04635502657202 | | | | | 1.08550513735704 | | | | 8.38032814132474 | | | | | 2.84494650496273e-16 | | | | 1.75461253875623e-14 | | | | | 26.2870612759885 | | | | |
| -1.337076471 | | | | | 1.18564394138376 | | | | -8.3720555 | | | | | 2.87332196871957e-16 | | | | 1.76952486424709e-14 | | | | | 26.278095153316 | | | | |
| -1.449876397 | | | | | 1.10785610567249 | | | | -8.367948659 | | | | | 2.89395126656079e-16 | | | | 1.78118877619426e-14 | | | | | 26.2657325813922 | | | | |
| -1.380039829 | | | | | 0.0758527772922518 | | | | -8.369662911 | | | | | 2.92096645788651e-16 | | | | 1.79545761850875e-14 | | | | | 26.2592168231131 | | | | |
| 1.00345263958031 | | | | | 1.07427083712976 | | | | 8.36217610413024 | | | | | 2.91518367948586e-16 | | | | 1.79242563739983e-14 | | | | | 26.2536801691702 | | | | |
| -1.139799131 | | | | | -3.435426267 | | | | -8.360988571 | | | | | 2.94219186782594e-16 | | | | 1.8074505316232e-14 | | | | | 26.2446759614724 | | | | |
| -1.310915352 | | | | | 0.341136767366988 | | | | -8.363741212 | | | | | 2.96596871777851e-16 | | | | 1.82099596567511e-14 | | | | | 26.2413286359241 | | | | |
| -1.043142155 | | | | | -3.374416894 | | | | -8.359854093 | | | | | 2.9682241624165e-16 | | | | 1.82211542082714e-14 | | | | | 26.2360750247042 | | | | |
| -1.258128618 | | | | | 1.54511150694759 | | | | -8.358898456 | | | | | 3.00186990014638e-16 | | | | 1.84116142661051e-14 | | | | | 26.2252762458264 | | | | |
| 1.30599200003631 | | | | | -0.003148663 | | | | 8.35814160316613 | | | | | 3.00795124045533e-16 | | | | 1.84453821335742e-14 | | | | | 26.2230937781267 | | | | |
| -1.238174564 | | | | | 2.16334859934975 | | | | -8.378704912 | | | | | 3.09701016945646e-16 | | | | 1.89469028206306e-14 | | | | | 26.2196712956267 | | | | |
| -1.707550053 | | | | | -0.447862016 | | | | -8.381922135 | | | | | 3.14421840138206e-16 | | | | 1.92064692642442e-14 | | | | | 26.2028959699762 | | | | |
| -1.081737312 | | | | | -0.599920893 | | | | -8.394838688 | | | | | 3.18334029648479e-16 | | | | 1.94313709151526e-14 | | | | | 26.1990577830619 | | | | |
| -1.408368088 | | | | | 0.122187367794145 | | | | -8.383110993 | | | | | 3.23125802406428e-16 | | | | 1.96982011133032e-14 | | | | | 26.1762985073638 | | | | |
| -1.253282989 | | | | | 1.2757064134014 | | | | -8.351854954 | | | | | 3.15834565576839e-16 | | | | 1.9289971214772e-14 | | | | | 26.1754578126484 | | | | |
| -1.272196505 | | | | | -0.4621951 | | | | -8.348946469 | | | | | 3.25534845784224e-16 | | | | 1.98249968835693e-14 | | | | | 26.1463368979466 | | | | |
| -1.268143116 | | | | | 0.234017995707889 | | | | -8.355760489 | | | | | 3.32878594752671e-16 | | | | 2.02284071262947e-14 | | | | | 26.1355510276984 | | | | |
| -1.009439401 | | | | | -2.72081411 | | | | -8.346127797 | | | | | 3.30812771452616e-16 | | | | 2.01173667923889e-14 | | | | | 26.1303231056918 | | | | |
| 1.07879360394553 | | | | | 3.49126901521516 | | | | 8.34346741274663 | | | | | 3.37061760776672e-16 | | | | 2.04619684230779e-14 | | | | | 26.111949063656 | | | | |
| -1.03363301 | | | | | 0.209534690546156 | | | | -8.342352636 | | | | | 3.39987075381758e-16 | | | | 2.06276762699605e-14 | | | | | 26.1035121965515 | | | | |
| -1.288876107 | | | | | 0.2567635610168 | | | | -8.354867162 | | | | | 3.45512646055224e-16 | | | | 2.09267907874327e-14 | | | | | 26.1025845777155 | | | | |
| -1.01230474 | | | | | -2.028949475 | | | | -8.339665386 | | | | | 3.47142224668855e-16 | | | | 2.10194517340776e-14 | | | | | 26.0831783572773 | | | | |
| -1.077899125 | | | | | 0.64116748933125 | | | | -8.353988152 | | | | | 3.57353162966813e-16 | | | | 2.1554156519556e-14 | | | | | 26.0766026980952 | | | | |
| 1.50796946016474 | | | | | 0.172439186766177 | | | | 8.33673192151131 | | | | | 3.55799817282222e-16 | | | | 2.14850506878697e-14 | | | | | 26.0592315895887 | | | | |
| 1.13183359784957 | | | | | -1.720030585 | | | | 8.33669948082192 | | | | | 3.56569415102873e-16 | | | | 2.15222767343871e-14 | | | | | 26.0572258156721 | | | | |
| -1.187792929 | | | | | 0.241786123462461 | | | | -8.332170958 | | | | | 3.685921542904e-16 | | | | 2.21876165167213e-14 | | | | | 26.0247461991656 | | | | |
| -1.086985895 | | | | | 1.17542734440932 | | | | -8.386913641 | | | | | 3.86089975846595e-16 | | | | 2.31385105337171e-14 | | | | | 26.0155960941358 | | | | |
| -1.743114865 | | | | | -0.082283018 | | | | -8.330631624 | | | | | 3.72303418118709e-16 | | | | 2.23886455102847e-14 | | | | | 26.0148617559671 | | | | |
| -1.026042185 | | | | | 0.349843243215193 | | | | -8.33019359 | | | | | 3.73568193708469e-16 | | | | 2.245728739726e-14 | | | | | 26.0115507535659 | | | | |
| 1.19819903533439 | | | | | -0.585423083 | | | | 8.33018098753431 | | | | | 3.73604646030732e-16 | | | | 2.245728739726e-14 | | | | | 26.0114554929217 | | | | |
| -1.242631165 | | | | | 1.10490936922675 | | | | -8.330946773 | | | | | 3.75680398595445e-16 | | | | 2.25788417439768e-14 | | | | | 26.0074375018442 | | | | |
| -1.198860761 | | | | | 0.241726847987045 | | | | -8.342163797 | | | | | 3.81883908177175e-16 | | | | 2.29222758094049e-14 | | | | | 26.0044344327286 | | | | |
| -1.383232452 | | | | | 1.04140960749058 | | | | -8.330023623 | | | | | 3.8355235664379e-16 | | | | 2.30060486807947e-14 | | | | | 25.9894341654134 | | | | |
| -1.034516266 | | | | | -1.685444529 | | | | -8.325830731 | | | | | 3.8713246903791e-16 | | | | 2.31976905242597e-14 | | | | | 25.9776073156273 | | | | |
| 1.03910642516375 | | | | | -0.754554099 | | | | 8.32539998956393 | | | | | 3.87689554419253e-16 | | | | 2.3224471432301e-14 | | | | | 25.975326377358 | | | | |
| -1.19536287 | | | | | 0.702718298600597 | | | | -8.365119565 | | | | | 4.03964959775585e-16 | | | | 2.41089636929449e-14 | | | | | 25.9683325747876 | | | | |
| -1.545074904 | | | | | 2.18869719606525 | | | | -8.329325277 | | | | | 3.9494180486531e-16 | | | | 2.36253526539304e-14 | | | | | 25.9664648384794 | | | | |
| -1.217891826 | | | | | 1.99388262755711 | | | | -8.325858981 | | | | | 3.93028793689855e-16 | | | | 2.35242656401989e-14 | | | | | 25.9644423070279 | | | | |
| -1.341314052 | | | | | 1.23386927430902 | | | | -8.322160679 | | | | | 3.99038542722038e-16 | | | | 2.38433586948564e-14 | | | | | 25.9481398256173 | | | | |
| 1.03151822197592 | | | | | 3.50733752747019 | | | | 8.3197021913762 | | | | | 4.05161465869981e-16 | | | | 2.41715386691277e-14 | | | | | 25.932291697839 | | | | |
| -1.037830355 | | | | | -0.242062681 | | | | -8.343986653 | | | | | 4.18447710461266e-16 | | | | 2.49007685676266e-14 | | | | | 25.9270156709018 | | | | |
| -1.373344299 | | | | | 1.82196285947933 | | | | -8.33841549 | | | | | 4.25067899419914e-16 | | | | 2.52590728788606e-14 | | | | | 25.9127368424982 | | | | |
| -1.258305199 | | | | | 0.269888266583234 | | | | -8.340346057 | | | | | 4.29202684993656e-16 | | | | 2.54832295383378e-14 | | | | | 25.9021984341373 | | | | |
| -1.020789344 | | | | | -0.101206903 | | | | -8.336607343 | | | | | 4.32038904419166e-16 | | | | 2.56371861562665e-14 | | | | | 25.8884777646053 | | | | |
| -1.098724393 | | | | | 0.0020354242625276 | | | | -8.313777809 | | | | | 4.25756988904288e-16 | | | | 2.52964561802552e-14 | | | | | 25.8848724411382 | | | | |
| -1.109040883 | | | | | 0.48387517271328 | | | | -8.312814256 | | | | | 4.27323515974841e-16 | | | | 2.53788038001565e-14 | | | | | 25.8803007963404 | | | | |
| -1.229229795 | | | | | 0.32363226565546 | | | | -8.312409743 | | | | | 4.29470022993342e-16 | | | | 2.54955124025908e-14 | | | | | 25.8755131197026 | | | | |
| -1.22132697 | | | | | -2.419382516 | | | | -8.31156703 | | | | | 4.31462584783745e-16 | | | | 2.56065908692135e-14 | | | | | 25.8708904227022 | | | | |
| 1.14769442705914 | | | | | 2.52782885090224 | | | | 8.31127598787818 | | | | | 4.32434123959847e-16 | | | | 2.56570278669127e-14 | | | | | 25.8686946694353 | | | | |
| -1.416272936 | | | | | 1.45189342098978 | | | | -8.35838066 | | | | | 4.4849877619087e-16 | | | | 2.6535495854694e-14 | | | | | 25.8684092432293 | | | | |
| -1.215767407 | | | | | -0.206433258 | | | | -8.349002843 | | | | | 4.54062376528527e-16 | | | | 2.68307850771839e-14 | | | | | 25.8538154674463 | | | | |
| -1.023445391 | | | | | -3.886955116 | | | | -8.308779315 | | | | | 4.4085761329042e-16 | | | | 2.61200547697305e-14 | | | | | 25.8498612501396 | | | | |
| -1.055952485 | | | | | -0.510956456 | | | | -8.309880141 | | | | | 4.46383225316659e-16 | | | | 2.64214506764845e-14 | | | | | 25.8411611800355 | | | | |
| -1.009872575 | | | | | -0.471866382 | | | | -8.316829378 | | | | | 4.48474822840683e-16 | | | | 2.6535495854694e-14 | | | | | 25.8411459099762 | | | | |
| -1.236113206 | | | | | 1.16033070794379 | | | | -8.307354801 | | | | | 4.4573614078114e-16 | | | | 2.6386853640767e-14 | | | | | 25.8391176725441 | | | | |
| -1.59470652 | | | | | -0.581581188 | | | | -8.30402875 | | | | | 4.66099490884371e-16 | | | | 2.74853837677155e-14 | | | | | 25.7973346890408 | | | | |
| -1.139677042 | | | | | -0.294363033 | | | | -8.328211913 | | | | | 4.79237133894423e-16 | | | | 2.81687112461993e-14 | | | | | 25.7930753747233 | | | | |
| -1.145615976 | | | | | -0.303200129 | | | | -8.300347383 | | | | | 4.71403954883198e-16 | | | | 2.77543850398768e-14 | | | | | 25.7845652496483 | | | | |
| -1.08450499 | | | | | -0.081842506 | | | | -8.299955927 | | | | | 4.71945421619703e-16 | | | | 2.77746302769547e-14 | | | | | 25.7833405796436 | | | | |
| 1.79875726395812 | | | | | -1.040259405 | | | | 8.30208226923808 | | | | | 4.74975547619905e-16 | | | | 2.79412582540298e-14 | | | | | 25.7799027361593 | | | | |
| -1.050249049 | | | | | 0.60992491778321 | | | | -8.299647305 | | | | | 4.78452013971008e-16 | | | | 2.81300698175063e-14 | | | | | 25.7721609807204 | | | | |
| -1.337150334 | | | | | 1.3215289734921 | | | | -8.296615502 | | | | | 4.84271443262231e-16 | | | | 2.84286147125693e-14 | | | | | 25.7581720316011 | | | | |
| -1.459138838 | | | | | 1.58913002865426 | | | | -8.298179237 | | | | | 4.87607020666892e-16 | | | | 2.8608494838543e-14 | | | | | 25.7556126509498 | | | | |
| 1.22605920159369 | | | | | 0.514570838143363 | | | | 8.29617415321213 | | | | | 4.85923595286541e-16 | | | | 2.8513693629902e-14 | | | | | 25.7548473009879 | | | | |
| -1.047655877 | | | | | -2.900102519 | | | | -8.293987261 | | | | | 4.94192501703657e-16 | | | | 2.89465414909544e-14 | | | | | 25.7383753815418 | | | | |
| -1.224157292 | | | | | -0.758680926 | | | | -8.295945362 | | | | | 5.02831293166151e-16 | | | | 2.93953793691073e-14 | | | | | 25.7248040905021 | | | | |
| -1.047692248 | | | | | -1.292506155 | | | | -8.295792404 | | | | | 5.05395219067366e-16 | | | | 2.95288904010165e-14 | | | | | 25.723820791226 | | | | |
| 1.23288920607722 | | | | | -1.877989501 | | | | 8.29921287637259 | | | | | 5.13539568144506e-16 | | | | 2.99507924895565e-14 | | | | | 25.7139099804579 | | | | |
| -1.104110148 | | | | | 2.04154258079245 | | | | -8.295722367 | | | | | 5.11692285586287e-16 | | | | 2.98513124049765e-14 | | | | | 25.7138079604934 | | | | |
| -1.192546171 | | | | | 2.08623767835572 | | | | -8.293049546 | | | | | 5.18210317856177e-16 | | | | 3.0189796177178e-14 | | | | | 25.7025038553478 | | | | |
| -1.092819693 | | | | | -0.708913452 | | | | -8.287590142 | | | | | 5.24070540064701e-16 | | | | 3.05017011220266e-14 | | | | | 25.6823847299603 | | | | |
| -1.014952136 | | | | | -1.135665646 | | | | -8.282136546 | | | | | 5.41477166209957e-16 | | | | 3.14107410477029e-14 | | | | | 25.6491773730211 | | | | |
| 1.10687501553355 | | | | | 0.015344315440762 | | | | 8.28034116394216 | | | | | 5.49020133185411e-16 | | | | 3.18132911310007e-14 | | | | | 25.6356731510805 | | | | |
| -1.125331603 | | | | | -0.386605316 | | | | -8.280004609 | | | | | 5.50445610951314e-16 | | | | 3.18871274584202e-14 | | | | | 25.6331419735762 | | | | |
| -1.256583973 | | | | | 0.921216093139802 | | | | -8.294061212 | | | | | 5.63570791598964e-16 | | | | 3.25982024270967e-14 | | | | | 25.6308434283607 | | | | |
| -1.222448918 | | | | | 0.539698085412886 | | | | -8.281027676 | | | | | 5.6952882788471e-16 | | | | 3.29202496030357e-14 | | | | | 25.6068172672949 | | | | |
| -1.311192776 | | | | | -0.048320004 | | | | -8.274789962 | | | | | 5.76206276144022e-16 | | | | 3.32788522752568e-14 | | | | | 25.5888099690194 | | | | |
| -1.365828333 | | | | | 1.12151208323745 | | | | -8.308193579 | | | | | 6.02111707427348e-16 | | | | 3.46847600643292e-14 | | | | | 25.5781125542879 | | | | |
| -1.407914174 | | | | | 0.642405037673281 | | | | -8.35390337 | | | | | 6.10032871674042e-16 | | | | 3.50883318781603e-14 | | | | | 25.5764963252416 | | | | |
| 1.16067833865564 | | | | | 3.58219211137441 | | | | 8.27211008206893 | | | | | 5.84951086916512e-16 | | | | 3.37423152968653e-14 | | | | | 25.573793125019 | | | | |
| -1.00078704 | | | | | 1.24476392976143 | | | | -8.297453755 | | | | | 6.08946775363867e-16 | | | | 3.5035419125076e-14 | | | | | 25.5672582593359 | | | | |
| 1.06870667566181 | | | | | 1.88821597444954 | | | | 8.26656292549311 | | | | | 6.10465567398772e-16 | | | | 3.5103643229925e-14 | | | | | 25.5321193748019 | | | | |
| -1.172657346 | | | | | -0.244911293 | | | | -8.26590599 | | | | | 6.2748810327782e-16 | | | | 3.60205201010742e-14 | | | | | 25.5096910704497 | | | | |
| -1.367408412 | | | | | 0.491970835886401 | | | | -8.266199143 | | | | | 6.29667108115824e-16 | | | | 3.61274433118922e-14 | | | | | 25.505841212764 | | | | |
| -1.567017439 | | | | | 1.11716721385123 | | | | -8.362059908 | | | | | 6.5957897585717e-16 | | | | 3.76446037661121e-14 | | | | | 25.5023737673684 | | | | |
| -1.443963264 | | | | | 1.64169072278474 | | | | -8.270406339 | | | | | 6.44527555318273e-16 | | | | 3.68612415910832e-14 | | | | | 25.4947128882205 | | | | |
| -1.026661615 | | | | | -0.674589657 | | | | -8.261513761 | | | | | 6.34642997759608e-16 | | | | 3.63699256408391e-14 | | | | | 25.4942071178374 | | | | |
| -1.082868439 | | | | | 1.02889917724623 | | | | -8.267915999 | | | | | 6.43727549642119e-16 | | | | 3.6825477663491e-14 | | | | | 25.4914268113533 | | | | |
| -1.014812359 | | | | | -0.084661221 | | | | -8.261116148 | | | | | 6.41309816585351e-16 | | | | 3.67141082131504e-14 | | | | | 25.4852290104676 | | | | |
| -1.35646857 | | | | | 1.15407042818109 | | | | -8.258966884 | | | | | 6.55645049738184e-16 | | | | 3.74259981214431e-14 | | | | | 25.464763725626 | | | | |
| -1.105858725 | | | | | -0.319299068 | | | | -8.257771461 | | | | | 6.65427432664289e-16 | | | | 3.79535757515962e-14 | | | | | 25.4522009800481 | | | | |
| -1.73964632 | | | | | 0.926661245904893 | | | | -8.253697891 | | | | | 6.85281222444404e-16 | | | | 3.90068214184843e-14 | | | | | 25.4218272320405 | | | | |
| 1.01920444057674 | | | | | 2.41773411933681 | | | | 8.2514057429958 | | | | | 6.85919994873164e-16 | | | | 3.903791122124e-14 | | | | | 25.4183679 | | | | |
| -1.12221848 | | | | | 0.144133964100693 | | | | -8.25703993 | | | | | 6.9551597311885e-16 | | | | 3.95573537071791e-14 | | | | | 25.4134276252315 | | | | |
| -1.089577806 | | | | | -0.14003654 | | | | -8.244950457 | | | | | 7.23428271686356e-16 | | | | 4.10010751776862e-14 | | | | | 25.3666127586344 | | | | |
| -1.140573148 | | | | | 0.35918685329565 | | | | -8.280422864 | | | | | 7.57406729346324e-16 | | | | 4.27773500620767e-14 | | | | | 25.3558366081528 | | | | |
| -1.147099121 | | | | | 2.7794503176571 | | | | -8.242767125 | | | | | 7.3836232769838e-16 | | | | 4.18025299034071e-14 | | | | | 25.3484987835161 | | | | |
| -1.030594647 | | | | | -0.829344657 | | | | -8.240543601 | | | | | 7.46947246032075e-16 | | | | 4.22488591863642e-14 | | | | | 25.3352814306334 | | | | |
| -1.343482342 | | | | | 1.57186883630954 | | | | -8.240292293 | | | | | 7.49758803691208e-16 | | | | 4.23908279367755e-14 | | | | | 25.3325336963097 | | | | |
| 1.17049019745028 | | | | | 0.782198853274974 | | | | 8.23729716715858 | | | | | 7.65786724733659e-16 | | | | 4.32216956523146e-14 | | | | | 25.3109696933742 | | | | |
| 1.13017975154692 | | | | | 3.94309587237299 | | | | 8.23630208108269 | | | | | 7.70251063102843e-16 | | | | 4.34486626997352e-14 | | | | | 25.3051913361173 | | | | |
| -1.205375723 | | | | | 0.0271072292059878 | | | | -8.237998114 | | | | | 7.74432579949273e-16 | | | | 4.36746002390277e-14 | | | | | 25.3033803361498 | | | | |
| -1.280331126 | | | | | 1.50463885797772 | | | | -8.24617408 | | | | | 8.0897442781751e-16 | | | | 4.54221367480439e-14 | | | | | 25.2790222491839 | | | | |
| -1.003734593 | | | | | 0.593979569751845 | | | | -8.243253457 | | | | | 8.04808563167464e-16 | | | | 4.52123137264621e-14 | | | | | 25.2775153110066 | | | | |
| -1.188422491 | | | | | -1.443948649 | | | | -8.23184522 | | | | | 7.97036425585759e-16 | | | | 4.48287385823033e-14 | | | | | 25.2718277277297 | | | | |
| -1.231684255 | | | | | 1.06945736790881 | | | | -8.231515957 | | | | | 8.01963578199549e-16 | | | | 4.50620602785635e-14 | | | | | 25.2660232261881 | | | | |
| -1.174368705 | | | | | -0.16120872 | | | | -8.308563967 | | | | | 8.42056302101766e-16 | | | | 4.71102402689733e-14 | | | | | 25.264531833875 | | | | |
| -1.291646835 | | | | | -0.451372672 | | | | -8.268185097 | | | | | 8.37639646383411e-16 | | | | 4.68818037815892e-14 | | | | | 25.2574487920518 | | | | |
| 1.24398178444933 | | | | | 0.605208285547788 | | | | 8.23148249375061 | | | | | 8.27979310274028e-16 | | | | 4.63903847679045e-14 | | | | | 25.2398811355151 | | | | |
| -1.173498011 | | | | | 0.765317610591041 | | | | -8.228210321 | | | | | 8.27065667537189e-16 | | | | 4.6345352827961e-14 | | | | | 25.2370770460855 | | | | |
| -1.661513412 | | | | | 3.61477902682402 | | | | -8.227803061 | | | | | 8.26620033027496e-16 | | | | 4.63265376563935e-14 | | | | | 25.2365741083229 | | | | |
| 1.08518229739658 | | | | | 0.774427430536936 | | | | 8.22619887381427 | | | | | 8.36843141461766e-16 | | | | 4.68496611072999e-14 | | | | | 25.2253977388842 | | | | |
| -1.076217641 | | | | | -0.225936136 | | | | -8.242342682 | | | | | 8.60757592087068e-16 | | | | 4.80417807882666e-14 | | | | | 25.2206277403898 | | | | |
| -1.116312109 | | | | | 0.412386608963938 | | | | -8.225084153 | | | | | 8.42485272264914e-16 | | | | 4.71279868341664e-14 | | | | | 25.2187354947949 | | | | |
| -1.102512742 | | | | | -0.911614702 | | | | -8.224414481 | | | | | 8.4375352877575e-16 | | | | 4.71913354733799e-14 | | | | | 25.2162354920633 | | | | |
| -1.277473676 | | | | | 1.72236349511182 | | | | -8.224303849 | | | | | 8.44469107751371e-16 | | | | 4.72201682357142e-14 | | | | | 25.2154081281142 | | | | |
| 1.06367567351603 | | | | | 1.00017266769264 | | | | 8.22221921126692 | | | | | 8.58065305784016e-16 | | | | 4.78978551085446e-14 | | | | | 25.1998198537754 | | | | |
| 1.21835387385483 | | | | | 1.74339039755926 | | | | 8.22048848676392 | | | | | 8.69517245082739e-16 | | | | 4.84985895150845e-14 | | | | | 25.1868805405571 | | | | |
| 1.00686563581951 | | | | | 0.816970565040837 | | | | 8.22044049008377 | | | | | 8.69836972469402e-16 | | | | 4.85035911374203e-14 | | | | | 25.1865217382421 | | | | |
| -1.070376709 | | | | | 0.185799642476959 | | | | -8.230818202 | | | | | 8.95994751296999e-16 | | | | 4.98501262408505e-14 | | | | | 25.1748788403718 | | | | |
| -1.082734771 | | | | | -0.362658365 | | | | -8.21986928 | | | | | 8.84857068610751e-16 | | | | 4.92824831754187e-14 | | | | | 25.171374081315 | | | | |
| 1.03451589642409 | | | | | 1.06134264285642 | | | | 8.21654826044558 | | | | | 8.96154877886507e-16 | | | | 4.98524574351455e-14 | | | | | 25.1574309471717 | | | | |
| 1.07725003827319 | | | | | -1.080737395 | | | | 8.21676522642601 | | | | | 8.96283364502e-16 | | | | 4.98530281373906e-14 | | | | | 25.1573963152123 | | | | |
| -1.116290302 | | | | | 0.390101999024349 | | | | -8.216377142 | | | | | 8.97329788804399e-16 | | | | 4.98980683166492e-14 | | | | | 25.1561522570157 | | | | |
| -1.064056079 | | | | | -0.068039131 | | | | -8.214206482 | | | | | 9.12366284097910e-16 | | | | 5.06607178055788e-14 | | | | | 25.1399338808919 | | | | |
| -1.125778244 | | | | | 2.5515336706864 | | | | -8.217168304 | | | | | 9.23752321450365e-16 | | | | 5.12322295654315e-14 | | | | | 25.1377822039762 | | | | |
| -1.255135906 | | | | | 0.694572180339961 | | | | -8.234092374 | | | | | 9.37398554039405e-16 | | | | 5.19207734509124e-14 | | | | | 25.1367578560367 | | | | |
| 1.18067225728055 | | | | | -0.844110684 | | | | 8.21210236426842 | | | | | 9.27179144083801e-16 | | | | 5.14155220523167e-14 | | | | | 25.1242160984858 | | | | |
| -1.147986878 | | | | | 0.224692326470404 | | | | -8.21188905 | | | | | 9.38845319512542e-16 | | | | 5.19940774729254e-14 | | | | | 25.1126490382641 | | | | |
| 1.42835713650886 | | | | | 0.899060528615066 | | | | 8.20939182150975 | | | | | 9.46611542464044e-16 | | | | 5.23966512353391e-14 | | | | | 25.1039732742902 | | | | |
| -1.118341116 | | | | | 0.178601423185677 | | | | -8.220021283 | | | | | 9.58804798249076e-16 | | | | 5.30158955255295e-14 | | | | | 25.1023129682122 | | | | |
| -1.091220515 | | | | | -2.48310626 | | | | -8.208210844 | | | | | 9.5692208093058e-16 | | | | 5.29256733822138e-14 | | | | | 25.0935063153912 | | | | |
| -1.008100988 | | | | | 0.14664444050016 | | | | -8.219751312 | | | | | 9.85048289434113e-16 | | | | 5.43885442196782e-14 | | | | | 25.0863430965329 | | | | |
| 1.08729376611861 | | | | | 1.82297258259292 | | | | 8.20575616247445 | | | | | 9.73308823585540e-16 | | | | 5.3789663386804e-14 | | | | | 25.0768302841953 | | | | |
| 1.07394608273133 | | | | | 0.399972384701199 | | | | 8.20246608259874 | | | | | 9.98108285343555e-16 | | | | 5.50591732968101e-14 | | | | | 25.0522759711506 | | | | |
| -1.0262801 | | | | | 0.676651123261473 | | | | -8.254179441 | | | | | 1.04587779012467e-15 | | | | 5.747625280358e-14 | | | | | 25.0482398922538 | | | | |
| -1.22364527 | | | | | 0.668179765162808 | | | | -8.205675674 | | | | | 1.0298794839135e-15 | | | | 5.66783192354221e-14 | | | | | 25.0305209960003 | | | | |
| -1.146649079 | | | | | 1.38262053422967 | | | | -8.197178206 | | | | | 1.03927934548816e-15 | | | | 5.71359741345491e-14 | | | | | 25.0128290794323 | | | | |
| -1.230913464 | | | | | 1.58920665157149 | | | | -8.216064708 | | | | | 1.06985178969425e-15 | | | | 5.8656144085343e-14 | | | | | 25.0101796687539 | | | | |
| 1.1250354651537 | | | | | 3.08167354422473 | | | | 8.19632824138921 | | | | | 1.04605168661591e-15 | | | | 5.74776112526788e-14 | | | | | 25.0064904345738 | | | | |
| -1.450393756 | | | | | 1.1793149252544 | | | | -8.195569341 | | | | | 1.06742147128567e-15 | | | | 5.85533513460427e-14 | | | | | 24.992534477228 | | | | |
| 1.02889076816196 | | | | | 0.73506401809671 | | | | 8.20639053129683 | | | | | 1.08577513939637e-15 | | | | 5.94469775934279e-14 | | | | | 24.9865178607484 | | | | |
| 1.31541110317913 | | | | | 3.11533383196224 | | | | 8.19270392907382 | | | | | 1.07542168033273e-15 | | | | 5.8953855947205e-14 | | | | | 24.9794681525077 | | | | |
| 1.14921249276726 | | | | | 0.865032171251279 | | | | 8.19238417115438 | | | | | 1.07997739789767e-15 | | | | 5.91728260617866e-14 | | | | | 24.9754485068055 | | | | |
| 1.78868140503147 | | | | | -2.126847357 | | | | 8.19277102347414 | | | | | 1.08065669149934e-15 | | | | 5.91946618738017e-14 | | | | | 24.9750469288065 | | | | |
| -1.303411045 | | | | | 0.201292912466109 | | | | -8.266039028 | | | | | 1.1419199474314e-15 | | | | 6.22995709966878e-14 | | | | | 24.9669904191386 | | | | |
| -1.365659782 | | | | | 2.32156090645831 | | | | -8.191175626 | | | | | 1.09194252815728e-15 | | | | 5.975852120027e-14 | | | | | 24.9664743709492 | | | | |
| 1.60111913886317 | | | | | 1.50649575280346 | | | | 8.19530904622789 | | | | | 1.10601714304905e-15 | | | | 6.04691981640948e-14 | | | | | 24.9573503899477 | | | | |
| -1.592108444 | | | | | -0.527711876 | | | | -8.198122148 | | | | | 1.12354988291957e-15 | | | | 6.13370376917006e-14 | | | | | 24.9487478941911 | | | | |
| -1.114576307 | | | | | 0.641059867474215 | | | | -8.185979672 | | | | | 1.14225316593818e-15 | | | | 6.23096885486611e-14 | | | | | 24.9220017954476 | | | | |
| -1.065904173 | | | | | 0.512085057370123 | | | | -8.18392974 | | | | | 1.1499371880842e-15 | | | | 6.26720969496529e-14 | | | | | 24.91409063 | | | | |
| -1.259837497 | | | | | 1.09139943047361 | | | | -8.189123474 | | | | | 1.18190881870596e-15 | | | | 6.42369051106923e-14 | | | | | 24.9036505569675 | | | | |
| -1.104416708 | | | | | -0.526501965 | | | | -8.181885125 | | | | | 1.16802048251909e-15 | | | | 6.35590652811747e-14 | | | | | 24.8988643768618 | | | | |
| -1.235844091 | | | | | 0.828690397903685 | | | | -8.188703452 | | | | | 1.21665939718732e-15 | | | | 6.59844074613475e-14 | | | | | 24.8780846536668 | | | | |
| -1.087726924 | | | | | 0.271650353768597 | | | | -8.17849043 | | | | | 1.20079391627112e-15 | | | | 6.51910352354892e-14 | | | | | 24.8719663138494 | | | | |
| -1.103210551 | | | | | 0.292596088718459 | | | | -8.180231787 | | | | | 1.22436237043619e-15 | | | | 6.63680268340181e-14 | | | | | 24.8630460672935 | | | | |
| -1.363148924 | | | | | 0.467020156209064 | | | | -8.195727371 | | | | | 1.24237299103715e-15 | | | | 6.7275127665421e-14 | | | | | 24.8606104230669 | | | | |
| 1.24779397095513 | | | | | 0.273095925635353 | | | | 8.17671720748986 | | | | | 1.22148070760676e-15 | | | | 6.6220335558825e-14 | | | | | 24.8555108325376 | | | | |
| -1.306873203 | | | | | -1.336868629 | | | | -8.175600077 | | | | | 1.22538343328217e-15 | | | | 6.64063015437723e-14 | | | | | 24.8520795569133 | | | | |
| -1.304818658 | | | | | 1.323518779 | | | | -8.17813744 | | | | | 1.26046903111085e-15 | | | | 6.81762411940115e-14 | | | | | 24.8399741798464 | | | | |
| -1.0551098 | | | | | -0.1049044 | | | | -8.173459846 | | | | | 1.25442500648846e-15 | | | | 6.79015908197678e-14 | | | | | 24.8296476765352 | | | | |
| -1.046229981 | | | | | -0.041465243 | | | | -8.172216666 | | | | | 1.25963134232902e-15 | | | | 6.81407102555488e-14 | | | | | 24.8252867563047 | | | | |
| -1.10417579 | | | | | 1.00617736162871 | | | | -8.172031338 | | | | | 1.26141199989722e-15 | | | | 6.82184940357395e-14 | | | | | 24.8239082900412 | | | | |
| -1.427659023 | | | | | 1.32726880308779 | | | | -8.200323532 | | | | | 1.30890522833169e-15 | | | | 7.05607348031991e-14 | | | | | 24.8232014816048 | | | | |
| 1.12255666857301 | | | | | 0.867472199786637 | | | | 8.17645812880175 | | | | | 1.27908865766599e-15 | | | | 6.90681667662974e-14 | | | | | 24.8164363695817 | | | | |
| -1.760202089 | | | | | -0.025113478 | | | | -8.185320289 | | | | | 1.31504826156233e-15 | | | | 7.08289034581491e-14 | | | | | 24.8048241069404 | | | | |
| 1.18356023989347 | | | | | 0.310826827575176 | | | | 8.17973814093889 | | | | | 1.31289823611283e-15 | | | | 7.07488561305681e-14 | | | | | 24.7962176396177 | | | | |
| -1.246896601 | | | | | 1.37801662144269 | | | | -8.171722462 | | | | | 1.31358139216575e-15 | | | | 7.07766247935768e-14 | | | | | 24.7923714785421 | | | | |
| -1.279770399 | | | | | 0.705427094273545 | | | | -8.165745191 | | | | | 1.32330390642415e-15 | | | | 7.12276689075657e-14 | | | | | 24.7771675523044 | | | | |
| 1.53059892292416 | | | | | 0.023616936435675 | | | | 8.16452100213223 | | | | | 1.34520890269394e-15 | | | | 7.23328536086399e-14 | | | | | 24.7632663281525 | | | | |
| 1.03314573391032 | | | | | 4.27401488603453 | | | | 8.16355360559755 | | | | | 1.34321062574868e-15 | | | | 7.22346160642068e-14 | | | | | 24.7624918166265 | | | | |
| -1.061722499 | | | | | -0.575056638 | | | | -8.163586503 | | | | | 1.34761929661741e-15 | | | | 7.24532231039989e-14 | | | | | 24.7595067467937 | | | | |
| -1.146289103 | | | | | -1.247561692 | | | | -8.162789782 | | | | | 1.35104762089979e-15 | | | | 7.26097693480517e-14 | | | | | 24.7568151116293 | | | | |
| 1.29366142741136 | | | | | -1.75521305 | | | | 8.16768047522604 | | | | | 1.37774815347729e-15 | | | | 7.38752624461811e-14 | | | | | 24.7514881809629 | | | | |
| -1.096595504 | | | | | 0.387899947364514 | | | | -8.16555945 | | | | | 1.3895446649463e-15 | | | | 7.44415320630044e-14 | | | | | 24.7364517043098 | | | | |
| 1.07437099890518 | | | | | 1.62774627778757 | | | | 8.15782205800881 | | | | | 1.40313023236927e-15 | | | | 7.51311650899238e-14 | | | | | 24.7199060241009 | | | | |
| -1.213988922 | | | | | 1.83264129425835 | | | | -8.154347527 | | | | | 1.44325996308247e-15 | | | | 7.70743957235288e-14 | | | | | 24.6933530572813 | | | | |
| -1.684757163 | | | | | 0.728439912356566 | | | | -8.159704086 | | | | | 1.46097108354218e-15 | | | | 7.7921537667244e-14 | | | | | 24.6910919488204 | | | | |
| -1.037379879 | | | | | -0.861203972 | | | | -8.177231011 | | | | | 1.49164484125616e-15 | | | | 7.93868332970449e-14 | | | | | 24.6888227781556 | | | | |
| 1.43558349882936 | | | | | -1.74943425 | | | | 8.15281042777479 | | | | | 1.46023759318109e-15 | | | | 7.78989614357012e-14 | | | | | 24.6819426054883 | | | | |
| -1.46059184 | | | | | 1.15176196330017 | | | | -8.151916036 | | | | | 1.46763317145798e-15 | | | | 7.8257066187239e-14 | | | | | 24.6760500865601 | | | | |
| -1.194564175 | | | | | 2.80894096882092 | | | | -8.157784581 | | | | | 1.48521886321493e-15 | | | | 7.91047414490137e-14 | | | | | 24.6735500854286 | | | | |
| -1.008544546 | | | | | -1.928459918 | | | | -8.151495768 | | | | | 1.4723331086266e-15 | | | | 7.848782577934e-14 | | | | | 24.6729303432611 | | | | |
| 1.59348246724372 | | | | | 0.749423117096546 | | | | 8.15267936808826 | | | | | 1.47992069433423e-15 | | | | 7.88524335582404e-14 | | | | | 24.6721556752803 | | | | |
| -1.343811677 | | | | | 0.895437908665623 | | | | -8.151119357 | | | | | 1.47914363698075e-15 | | | | 7.88209904468181e-14 | | | | | 24.6685335127475 | | | | |
| -1.037369897 | | | | | 0.22788891320862 | | | | -8.150601148 | | | | | 1.48238732010679e-15 | | | | 7.89738801351146e-14 | | | | | 24.6662898373435 | | | | |
| -1.309332854 | | | | | 0.398032799971923 | | | | -8.189133963 | | | | | 1.56797844824082e-15 | | | | 8.30823664576159e-14 | | | | | 24.6523941202842 | | | | |
| -1.053625149 | | | | | -0.19809717 | | | | -8.15458398 | | | | | 1.53629996644722e-15 | | | | 8.15472818292799e-14 | | | | | 24.6403072678017 | | | | |
| -1.202214157 | | | | | 1.8681247525398 | | | | -8.146982159 | | | | | 1.54816401802894e-15 | | | | 8.21150089914797e-14 | | | | | 24.6274413068936 | | | | |
| -1.195260416 | | | | | -0.493796011 | | | | -8.14330937 | | | | | 1.56690331657461e-15 | | | | 8.30358327887416e-14 | | | | | 24.6121878151062 | | | | |
| -1.064101208 | | | | | 0.548508490069473 | | | | -8.14208541 | | | | | 1.5843095542001e-15 | | | | 8.38423455973341e-14 | | | | | 24.602377018777 | | | | |
| 1.49222645338305 | | | | | 0.256447646688644 | | | | 8.14188558543993 | | | | | 1.58395227113222e-15 | | | | 8.38339593533729e-14 | | | | | 24.6016286506215 | | | | |
| 1.53322453145285 | | | | | -1.833935348 | | | | 8.14153051273401 | | | | | 1.58823249227796e-15 | | | | 8.40077765954402e-14 | | | | | 24.5989955781402 | | | | |
| -1.120836704 | | | | | 0.14262724285482 | | | | -8.22781813 | | | | | 1.67377675772175e-15 | | | | 8.81125400035616e-14 | | | | | 24.5978184789576 | | | | |
| -1.01346803 | | | | | -0.423864183 | | | | -8.145420226 | | | | | 1.6129593088882e-15 | | | | 8.52087887470419e-14 | | | | | 24.5924532697212 | | | | |
| 1.16484491407974 | | | | | 1.54481920017128 | | | | 8.1403905730937 | | | | | 1.60205117893704e-15 | | | | 8.46962036888642e-14 | | | | | 24.5905429073478 | | | | |
| -1.259983602 | | | | | 1.71685781246093 | | | | -8.171123803 | | | | | 1.66283896510578e-15 | | | | 8.7637568932821e-14 | | | | | 24.5892803931861 | | | | |
| -1.359312333 | | | | | 0.58210075570746 | | | | -8.139225481 | | | | | 1.61629737421571e-15 | | | | 8.53744348973491e-14 | | | | | 24.581904756008 | | | | |
| -1.010029387 | | | | | 0.643430221882878 | | | | -8.137490031 | | | | | 1.64060289385014e-15 | | | | 8.65606884696935e-14 | | | | | 24.5674479552939 | | | | |
| -1.017599209 | | | | | -2.454480034 | | | | -8.136750264 | | | | | 1.64697939053451e-15 | | | | 8.68536517767244e-14 | | | | | 24.5635566014089 | | | | |
| 1.11082080515022 | | | | | -3.723132304 | | | | 8.13660735815423 | | | | | 1.6487682533074e-15 | | | | 8.69275619163565e-14 | | | | | 24.5624974186865 | | | | |
| 1.15166157601114 | | | | | 0.302270036436853 | | | | 8.13569919429202 | | | | | 1.6630711903408e-15 | | | | 8.7637568932821e-14 | | | | | 24.5550413551609 | | | | |
| -1.12864801 | | | | | 0.020320506497268 | | | | -8.133779255 | | | | | 1.68456684176575e-15 | | | | 8.86141841526151e-14 | | | | | 24.5415394440536 | | | | |
| -1.327137807 | | | | | 1.30889628910915 | | | | -8.138138951 | | | | | 1.70764361301261e-15 | | | | 8.96603265221052e-14 | | | | | 24.5401615618716 | | | | |
| -1.189849116 | | | | | 0.871897267637953 | | | | -8.136451293 | | | | | 1.71374467292145e-15 | | | | 8.99134896392669e-14 | | | | | 24.5312733923562 | | | | |
| 1.63755128179328 | | | | | 2.95705578921585 | | | | 8.13159648101852 | | | | | 1.71272023930173e-15 | | | | 8.98821086877861e-14 | | | | | 24.5253679303321 | | | | |
| -1.236421818 | | | | | 0.820492288528402 | | | | -8.136467813 | | | | | 1.73584531959736e-15 | | | | 9.09485457150371e-14 | | | | | 24.5251882136324 | | | | |
| -1.284870605 | | | | | 0.898705767425082 | | | | -8.131513495 | | | | | 1.71677717705756e-15 | | | | 9.00474566491037e-14 | | | | | 24.5231660861526 | | | | |
| -1.281200035 | | | | | 3.70649516778692 | | | | -8.129885152 | | | | | 1.74115448693435e-15 | | | | 9.11813974795839e-14 | | | | | 24.509516196164 | | | | |
| 1.22863858021177 | | | | | -1.375429973 | | | | 8.12877262761899 | | | | | 1.7498321224643e-15 | | | | 9.16130756244751e-14 | | | | | 24.504452253576 | | | | |
| 1.00791006289733 | | | | | 0.184144702484041 | | | | 8.12869483491682 | | | | | 1.75086564899094e-15 | | | | 9.165580610925e-14 | | | | | 24.503876145943 | | | | |
| 1.24787911477959 | | | | | 1.0745711460075 | | | | 8.12788645427644 | | | | | 1.76469672978149e-15 | | | | 9.23110867196331e-14 | | | | | 24.4971744092654 | | | | |
| 1.24599084089872 | | | | | 0.326482523044143 | | | | 8.17017814765129 | | | | | 1.90021665180396e-15 | | | | 9.87751917611166e-14 | | | | | 24.4673540862738 | | | | |
| -1.199838758 | | | | | 1.2827003494242 | | | | -8.122908206 | | | | | 1.84865538327372e-15 | | | | 9.63920508798106e-14 | | | | | 24.4532245542795 | | | | |
| -1.126283299 | | | | | 1.22106014393841 | | | | -8.120919865 | | | | | 1.85725838266138e-15 | | | | 9.6756874501708e-14 | | | | | 24.446320556624 | | | | |
| -1.45153853 | | | | | 1.24517949626148 | | | | -8.123041639 | | | | | 1.89349888823741e-15 | | | | 9.84381304651795e-14 | | | | | 24.4380298544496 | | | | |
| -1.368910054 | | | | | -0.092401891 | | | | -8.120093186 | | | | | 1.87867025319018e-15 | | | | 9.77636536668768e-14 | | | | | 24.4363283655612 | | | | |
| -1.123120518 | | | | | 0.907828010667091 | | | | -8.118883051 | | | | | 1.88616895645646e-15 | | | | 9.81175504402905e-14 | | | | | 24.4312503191305 | | | | |
| -1.129783311 | | | | | 0.508096485802368 | | | | -8.118813568 | | | | | 1.89042258933875e-15 | | | | 9.8290319759121e-14 | | | | | 24.4291592734597 | | | | |
| 1.2337710161419 | | | | | 2.331609793 | | | | 8.116402744 | | | | | 1.92197465732236e-15 | | | | 9.98323544904067e-14 | | | | | 24.412902999484 | | | | |
| -1.093864576 | | | | | -0.865026133 | | | | -8.150200344 | | | | | 1.9895240514916e-15 | | | | 1.02960555570995e-13 | | | | | 24.4105232014613 | | | | |
| -1.158470676 | | | | | 1.01718066774695 | | | | -8.118426822 | | | | | 1.97152774396336e-15 | | | | 1.02154596190345e-13 | | | | | 24.3956581567317 | | | | |
| -1.092051625 | | | | | 0.156912921897859 | | | | -8.113561031 | | | | | 1.96720644217612e-15 | | | | 1.01968141029111e-13 | | | | | 24.3903152202419 | | | | |
| -1.092079019 | | | | | -3.412507596 | | | | -8.112973717 | | | | | 1.97258304578455e-15 | | | | 1.02196718628329e-13 | | | | | 24.387545548042 | | | | |
| -1.075201083 | | | | | -1.054426571 | | | | -8.11153952 | | | | | 1.99413805252274e-15 | | | | 1.03174011362855e-13 | | | | | 24.3769424172078 | | | | |
| -1.113499592 | | | | | 1.59080685682874 | | | | -8.11488767 | | | | | 2.03242361299337e-15 | | | | 1.0484610532064e-13 | | | | | 24.3695102488452 | | | | |
| 1.01867758025807 | | | | | 2.45335852719486 | | | | 8.1103827213369 | | | | | 2.01169315850582e-15 | | | | 1.03967478140354e-13 | | | | | 24.3683912558031 | | | | |
| -1.025141626 | | | | | 0.00137201391514119 | | | | -8.110781696 | | | | | 2.01601939167166e-15 | | | | 1.04131649297976e-13 | | | | | 24.3666167038086 | | | | |
| 1.07370396378742 | | | | | -0.894126051 | | | | 8.1103156696467 | | | | | 2.01965577931488e-15 | | | | 1.04258131094169e-13 | | | | | 24.3647511714914 | | | | |
| 1.19685649843951 | | | | | 4.31409076430873 | | | | 8.10978603276595 | | | | | 2.02080778715935e-15 | | | | 1.04297921617073e-13 | | | | | 24.3639808808543 | | | | |
| -1.287973052 | | | | | -0.061649486 | | | | -8.136121915 | | | | | 2.08922539991412e-15 | | | | 1.07513262677787e-13 | | | | | 24.3620707666319 | | | | |
| -1.319297935 | | | | | -1.261521678 | | | | -8.122729434 | | | | | 2.07833122319124e-15 | | | | 1.07017941981078e-13 | | | | | 24.3545277374204 | | | | |
| -1.164056949 | | | | | -0.18929008 | | | | -8.109744034 | | | | | 2.05327930128054e-15 | | | | 1.05857229853401e-13 | | | | | 24.3511369432202 | | | | |
| -1.39342182 | | | | | 1.89223019887719 | | | | -8.107511579 | | | | | 2.05592611506632e-15 | | | | 1.05967774714585e-13 | | | | | 24.3471719363275 | | | | |
| -1.00193301 | | | | | -1.327199599 | | | | -8.111708516 | | | | | 2.08185157486053e-15 | | | | 1.07173037916181e-13 | | | | | 24.3394003280816 | | | | |
| 1.26213720407389 | | | | | 0.58370126320359 | | | | 8.10610012617085 | | | | | 2.07802151394725e-15 | | | | 1.07015062504413e-13 | | | | | 24.3367428410871 | | | | |
| -1.031277586 | | | | | 0.648183974968435 | | | | -8.1053645 | | | | | 2.09681920907575e-15 | | | | 1.07877715457659e-13 | | | | | 24.3290498995425 | | | | |
| -1.007649979 | | | | | -1.451344043 | | | | -8.101326952 | | | | | 2.15449281573131e-15 | | | | 1.1053817122356e-13 | | | | | 24.3014855825029 | | | | |
| 1.0760457043983 | | | | | 2.99712459911662 | | | | 8.10069827373406 | | | | | 2.16847771571525e-15 | | | | 1.11225154894252e-13 | | | | | 24.2952804424263 | | | | |
| -1.049808418 | | | | | 0.286614332890238 | | | | -8.098574939 | | | | | 2.19983811881564e-15 | | | | 1.12628185959324e-13 | | | | | 24.2811656407648 | | | | |
| -1.050524948 | | | | | -0.652677794 | | | | -8.110301304 | | | | | 2.25899871203902e-15 | | | | 1.15335026868324e-13 | | | | | 24.2719820390515 | | | | |
| 1.67567193113631 | | | | | -1.468832432 | | | | 8.09778294307967 | | | | | 2.27965105371387e-15 | | | | 1.16276818654836e-13 | | | | | 24.2525650657066 | | | | |
| -1.0666845 | | | | | -0.18706214 | | | | -8.094288126 | | | | | 2.27235506075706e-15 | | | | 1.15946751992913e-13 | | | | | 24.2495248109454 | | | | |
| -1.323030647 | | | | | 1.32149797677874 | | | | -8.093558815 | | | | | 2.33284152552619e-15 | | | | 1.18760024878502e-13 | | | | | 24.2295459289692 | | | | |
| -1.155904036 | | | | | 0.4795687637998 | | | | -8.09069907 | | | | | 2.35495227631703e-15 | | | | 1.1979885708223e-13 | | | | | 24.2161145731279 | | | | |
| -1.133807016 | | | | | -3.506868177 | | | | -8.086151811 | | | | | 2.41653755548581e-15 | | | | 1.22621106019459e-13 | | | | | 24.1895096192342 | | | | |
| -1.139026078 | | | | | 0.867704852825308 | | | | -8.085106212 | | | | | 2.4398513268048e-15 | | | | 1.23699904890439e-13 | | | | | 24.1811394396336 | | | | |
| -1.315890362 | | | | | 0.434556345742277 | | | | -8.14533303 | | | | | 2.59923829111546e-15 | | | | 1.31275698911804e-13 | | | | | 24.1670425875135 | | | | |
| 1.53806429191013 | | | | | 0.397355817428349 | | | | 8.10213985352006 | | | | | 2.59732317598476e-15 | | | | 1.3120023117296e-13 | | | | | 24.1402233050229 | | | | |
| -1.237481766 | | | | | 1.46004732673801 | | | | -8.082609783 | | | | | 2.57910462637869e-15 | | | | 1.30400776909838e-13 | | | | | 24.1352989068592 | | | | |
| -1.378842497 | | | | | 4.34099412180593 | | | | -8.077726545 | | | | | 2.57971741156099e-15 | | | | 1.30414751340717e-13 | | | | | 24.1267644980837 | | | | |
| -1.009559768 | | | | | 0.159947982546403 | | | | -8.075460282 | | | | | 2.61980072149068e-15 | | | | 1.32266687048728e-13 | | | | | 24.110723754823 | | | | |
| -1.13751545 | | | | | 0.170136420265173 | | | | -8.08590633 | | | | | 2.69737516571109e-15 | | | | 1.35857881763664e-13 | | | | | 24.1011373563835 | | | | |
| -1.15682208 | | | | | 1.50116064194351 | | | | -8.080336825 | | | | | 2.71672294553294e-15 | | | | 1.36750694096081e-13 | | | | | 24.0923186932718 | | | | |
| -1.027966957 | | | | | 0.814101119987409 | | | | -8.073274094 | | | | | 2.69532903158066e-15 | | | | 1.35771042019665e-13 | | | | | 24.0864153597075 | | | | |
| -1.386880867 | | | | | 0.814588475201844 | | | | -8.070049072 | | | | | 2.72901496291768e-15 | | | | 1.37353036454225e-13 | | | | | 24.0708819513106 | | | | |
| 1.55628663353362 | | | | | -5.139800754 | | | | 8.0701924232381 | | | | | 2.73066882288308e-15 | | | | 1.37403475244046e-13 | | | | | 24.0703983820261 | | | | |
| 1.105052462 | | | | | -0.024346329 | | | | 8.07212784549615 | | | | | 2.75189196879753e-15 | | | | 1.38372321307281e-13 | | | | | 24.0651489270568 | | | | |
| -1.158996971 | | | | | -0.117333756 | | | | -8.071639499 | | | | | 2.76687316100964e-15 | | | | 1.39015627829583e-13 | | | | | 24.0634671116118 | | | | |
| 1.12392212301132 | | | | | -3.08112925 | | | | 8.06651758014791 | | | | | 2.80270082299553e-15 | | | | 1.40675497682719e-13 | | | | | 24.0448922949818 | | | | |
| -1.629162075 | | | | | 1.51953322880846 | | | | -8.072537439 | | | | | 2.859364313462e-15 | | | | 1.43229763608639e-13 | | | | | 24.0388113018248 | | | | |
| -1.098179258 | | | | | -1.833884401 | | | | -8.124349789 | | | | | 2.9471602446405e-15 | | | | 1.47295135754126e-13 | | | | | 24.0382664314902 | | | | |
| -1.34575165 | | | | | 2.78649740407073 | | | | -8.064550495 | | | | | 2.84459239414218e-15 | | | | 1.42523678093861e-13 | | | | | 24.0304198847412 | | | | |
| -1.214615843 | | | | | 0.418767839110166 | | | | -8.062991399 | | | | | 2.97387408006539e-15 | | | | 1.48507044450636e-13 | | | | | 23.9935402244368 | | | | |
| -1.113236101 | | | | | -3.919466804 | | | | -8.059411906 | | | | | 2.95696213460068e-15 | | | | 1.47728019137582e-13 | | | | | 23.99262781 | | | | |
| -1.022210552 | | | | | 0.663468368782003 | | | | -8.057754471 | | | | | 2.99413225345309e-15 | | | | 1.49465579177645e-13 | | | | | 23.9804424196773 | | | | |
| -1.328068387 | | | | | 1.41956134647622 | | | | -8.108957629 | | | | | 3.14355429363466e-15 | | | | 1.56185052314944e-13 | | | | | 23.9786794856352 | | | | |
| -1.079833435 | | | | | -0.939606322 | | | | -8.108254866 | | | | | 3.17876585002955e-15 | | | | 1.57674414302077e-13 | | | | | 23.9670943749582 | | | | |
| -1.17358956 | | | | | 1.71879172200627 | | | | -8.055323965 | | | | | 3.04947456860836e-15 | | | | 1.51904541210288e-13 | | | | | 23.9625772619212 | | | | |
| -1.277189743 | | | | | 0.515880441970839 | | | | -8.092281765 | | | | | 3.24143729733646e-15 | | | | 1.60607398952135e-13 | | | | | 23.9465966759321 | | | | |
| 1.2144858323363 | | | | | 3.36937249570017 | | | | 8.05303978878229 | | | | | 3.10240431263851e-15 | | | | 1.54294305821305e-13 | | | | | 23.9457918146822 | | | | |
| 1.1361987867107 | | | | | 3.4853067674282 | | | | 8.05198756437356 | | | | | 3.12709076687658e-15 | | | | 1.55458661125778e-13 | | | | | 23.9380608104887 | | | | |
| -1.006870236 | | | | | -0.529941086 | | | | -8.051811472 | | | | | 3.1312410028869e-15 | | | | 1.55646633701204e-13 | | | | | 23.9367670879267 | | | | |
| 1.13777160473621 | | | | | 0.926475141779062 | | | | 8.05080419210466 | | | | | 3.15508569130286e-15 | | | | 1.56628794849503e-13 | | | | | 23.9293672398015 | | | | |
| 1.35446842262586 | | | | | 1.43431369183579 | | | | 8.05135622610279 | | | | | 3.16317029534342e-15 | | | | 1.57008723148738e-13 | | | | | 23.9291057369533 | | | | |
| 1.37560418881216 | | | | | 0.898748192156063 | | | | 8.04859523527901 | | | | | 3.20800589402454e-15 | | | | 1.5903125386002e-13 | | | | | 23.9131421531431 | | | | |
| -1.150895216 | | | | | 0.593940143827827 | | | | -8.058893534 | | | | | 3.25854081748909e-15 | | | | 1.61345865101649e-13 | | | | | 23.9122325917097 | | | | |
| -1.059810903 | | | | | 2.20594153049798 | | | | -8.04577299 | | | | | 3.27689447320894e-15 | | | | 1.62160320680438e-13 | | | | | 23.8924178330278 | | | | |
| -1.025735597 | | | | | -1.614717267 | | | | -8.04246596 | | | | | 3.35947487258906e-15 | | | | 1.66032556203074e-13 | | | | | 23.8681414248956 | | | | |
| -1.24818264 | | | | | 1.13796451450564 | | | | -8.048266766 | | | | | 3.44356070428454e-15 | | | | 1.69929343730329e-13 | | | | | 23.8627362758945 | | | | |
| -1.189695621 | | | | | -0.905474619 | | | | -8.039855064 | | | | | 3.43182509830729e-15 | | | | 1.69429538602416e-13 | | | | | 23.8483764625526 | | | | |
| -1.002624313 | | | | | -0.261505888 | | | | -8.038907202 | | | | | 3.45063529795877e-15 | | | | 1.7019878185639e-13 | | | | | 23.8420265027567 | | | | |
| -1.037896573 | | | | | -0.286007192 | | | | -8.040433991 | | | | | 3.46913732244482e-15 | | | | 1.71051348778684e-13 | | | | | 23.8397020311762 | | | | |
| 1.71693985509507 | | | | | 0.318001774598345 | | | | 8.04484863051096 | | | | | 3.50716425136181e-15 | | | | 1.72684020302631e-13 | | | | | 23.8370374453836 | | | | |
| 1.35999265151399 | | | | | -1.663995721 | | | | 8.04525503471038 | | | | | 3.54834869072081e-15 | | | | 1.74426695315487e-13 | | | | | 23.8355063986807 | | | | |
| -1.015008995 | | | | | 1.91456587587662 | | | | -8.038962302 | | | | | 3.48401790545849e-15 | | | | 1.71618510670291e-13 | | | | | 23.8341928887507 | | | | |
| -1.400214073 | | | | | 0.326881935240771 | | | | -8.039293916 | | | | | 3.51698331005267e-15 | | | | 1.72985693730306e-13 | | | | | 23.8302342161252 | | | | |
| -1.345887491 | | | | | 0.239450742949737 | | | | -8.03728394 | | | | | 3.50466741398355e-15 | | | | 1.72581234199982e-13 | | | | | 23.8279984508298 | | | | |
| -1.408063983 | | | | | 0.49044158515619 | | | | -8.035376171 | | | | | 3.55529877832524e-15 | | | | 1.74686883693358e-13 | | | | | 23.8130974421326 | | | | |
| -1.416535182 | | | | | 0.859711729454745 | | | | -8.108702085 | | | | | 3.78186113525072e-15 | | | | 1.84828162366849e-13 | | | | | 23.8023171099082 | | | | |
| -1.222388094 | | | | | 3.29780745838665 | | | | -8.032849625 | | | | | 3.61144961584451e-15 | | | | 1.77239287652835e-13 | | | | | 23.7975971033009 | | | | |
| -1.423652078 | | | | | 0.137635276909829 | | | | -8.032151992 | | | | | 3.64251232701953e-15 | | | | 1.78659785149974e-13 | | | | | 23.791284323727 | | | | |
| -1.070851873 | | | | | -0.558579713 | | | | -8.081354571 | | | | | 3.86378134919443e-15 | | | | 1.88525883072763e-13 | | | | | 23.7776385069519 | | | | |
| 1.01687501223615 | | | | | 1.43491090606794 | | | | 8.03026279647102 | | | | | 3.68846127258273e-15 | | | | 1.80745318397439e-13 | | | | | 23.7771247763727 | | | | |
| -1.231101329 | | | | | 0.139805805645383 | | | | -8.043885091 | | | | | 3.76885608433615e-15 | | | | 1.84342134912658e-13 | | | | | 23.7755477384297 | | | | |
| -1.218910878 | | | | | -0.475735558 | | | | -8.038750289 | | | | | 3.81066071616094e-15 | | | | 1.86149363320619e-13 | | | | | 23.7648864061919 | | | | |
| -1.216673702 | | | | | -1.454429402 | | | | -8.030823466 | | | | | 3.75436431084286e-15 | | | | 1.83697238216884e-13 | | | | | 23.7639801798961 | | | | |
| -1.089663638 | | | | | -2.404692539 | | | | -8.028300588 | | | | | 3.75570374384864e-15 | | | | 1.83741454654676e-13 | | | | | 23.7597205475825 | | | | |
| -1.052122544 | | | | | -0.658474909 | | | | -8.058458633 | | | | | 3.89016636888905e-15 | | | | 1.8970353333751e-13 | | | | | 23.755093837509 | | | | |
| -1.236363129 | | | | | 1.39383679926865 | | | | -8.026795041 | | | | | 3.81749854466388e-15 | | | | 1.86461787481573e-13 | | | | | 23.7459523239929 | | | | |
| 1.06103569603681 | | | | | 0.890300826404175 | | | | 8.02518966289747 | | | | | 3.82542448847592e-15 | | | | 1.86827280988608e-13 | | | | | 23.7414553726493 | | | | |
| -1.154881623 | | | | | 0.612763903293532 | | | | -8.033190889 | | | | | 3.97994076601081e-15 | | | | 1.93790025727919e-13 | | | | | 23.7218532177552 | | | | |
| 1.07692333677945 | | | | | 2.67989355618298 | | | | 8.01966461475298 | | | | | 3.9874789376262e-15 | | | | 1.94112242395386e-13 | | | | | 23.7009889598127 | | | | |
| -1.164555105 | | | | | 0.562951617361666 | | | | -8.023474314 | | | | | 4.13850909942363e-15 | | | | 2.01000360741069e-13 | | | | | 23.6768755168255 | | | | |
| 1.07599807056778 | | | | | 3.23708777850324 | | | | 8.01575446539433 | | | | | 4.10624072266839e-15 | | | | 1.99548061026574e-13 | | | | | 23.6723645382375 | | | | |
| -1.177890105 | | | | | 1.42146668496052 | | | | -8.027934807 | | | | | 4.23681953637579e-15 | | | | 2.05491108109452e-13 | | | | | 23.6660827870286 | | | | |
| -1.35851114 | | | | | 1.02071203233012 | | | | -8.02056967 | | | | | 4.22173405300033e-15 | | | | 2.04806557538961e-13 | | | | | 23.6599236817043 | | | | |
| 1.14617640338449 | | | | | 1.85995949262808 | | | | 8.01163618449177 | | | | | 4.23509715581943e-15 | | | | 2.05431199550716e-13 | | | | | 23.6422292027683 | | | | |
| -1.390927575 | | | | | 1.85380312017919 | | | | -8.029740019 | | | | | 4.38071401475386e-15 | | | | 2.12006849374642e-13 | | | | | 23.6359907868454 | | | | |
| -1.263607215 | | | | | 0.501629699170635 | | | | -8.011528913 | | | | | 4.28074032868547e-15 | | | | 2.07501985791103e-13 | | | | | 23.6324278396937 | | | | |
| -1.265215888 | | | | | -0.580770822 | | | | -8.034309605 | | | | | 4.41215272491536e-15 | | | | 2.13308007282167e-13 | | | | | 23.6282725725054 | | | | |
| -1.050958395 | | | | | 0.643178199709182 | | | | -8.009711619 | | | | | 4.31792988047155e-15 | | | | 2.09160424204301e-13 | | | | | 23.623663546223 | | | | |
| -1.150275691 | | | | | -0.198111771 | | | | -8.009171277 | | | | | 4.32120375480849e-15 | | | | 2.09294967527004e-13 | | | | | 23.6227070035442 | | | | |
| 1.05490052230418 | | | | | -0.581167261 | | | | 8.00822030064256 | | | | | 4.34499615609889e-15 | | | | 2.10374845463569e-13 | | | | | 23.6172435313175 | | | | |
| -1.187526801 | | | | | 0.351491176531674 | | | | -8.007721647 | | | | | 4.36842818697274e-15 | | | | 2.11436537373829e-13 | | | | | 23.6130319493323 | | | | |
| -1.17262846 | | | | | -1.614183468 | | | | -8.008241064 | | | | | 4.38754153564083e-15 | | | | 2.12312903297414e-13 | | | | | 23.6102333152936 | | | | |
| 1.01420281548752 | | | | | 0.602495289310068 | | | | 8.00921664125113 | | | | | 4.45237018416623e-15 | | | | 2.1507972683668e-13 | | | | | 23.5973515529661 | | | | |
| -1.020578638 | | | | | -0.445033846 | | | | -8.008915791 | | | | | 4.4700914709002e-15 | | | | 2.15720483114988e-13 | | | | | 23.597186554843 | | | | |
| -1.185065256 | | | | | 0.78885099671909 | | | | -8.004194478 | | | | | 4.47813418027865e-15 | | | | 2.16027324081695e-13 | | | | | 23.587807962328 | | | | |
| -1.218414264 | | | | | 0.629186854510664 | | | | -7.999877463 | | | | | 4.67121285541277e-15 | | | | 2.24570781523418e-13 | | | | | 23.5491415410831 | | | | |
| -1.306776898 | | | | | 0.107626118232682 | | | | -7.998955279 | | | | | 4.66505140174635e-15 | | | | 2.24325718391422e-13 | | | | | 23.5480354591771 | | | | |
| -1.193529652 | | | | | 0.47596362727643 | | | | -8.002721486 | | | | | 4.6901707642483e-15 | | | | 2.25302381654201e-13 | | | | | 23.5477421764635 | | | | |
| -1.359740964 | | | | | 0.280589074859327 | | | | -8.10022668 | | | | | 4.94101935566662e-15 | | | | 2.36329359313061e-13 | | | | | 23.5443354702154 | | | | |
| -1.352106964 | | | | | 0.13381885304752 | | | | -8.08826687 | | | | | 4.97373036508702e-15 | | | | 2.37678250571877e-13 | | | | | 23.5374335475267 | | | | |
| -1.516412104 | | | | | 0.373642218692789 | | | | -8.21422595 | | | | | 4.75737495011515e-15 | | | | 2.28270629860611e-13 | | | | | 23.5347988297613 | | | | |
| -1.140783525 | | | | | -0.572806149 | | | | -8.003627124 | | | | | 4.85264564726398e-15 | | | | 2.32445199373146e-13 | | | | | 23.5215213532902 | | | | |
| -1.258470869 | | | | | 1.7682994802243 | | | | -7.995801262 | | | | | 4.80800643457338e-15 | | | | 2.30542651042365e-13 | | | | | 23.5199627265036 | | | | |
| -1.279209808 | | | | | 0.680304980470967 | | | | -8.028373296 | | | | | 5.05345177447132e-15 | | | | 2.41241821548887e-13 | | | | | 23.5080676 | | | | |
| 1.07404399875812 | | | | | -4.140483628 | | | | 7.99018676549778 | | | | | 4.97350996947572e-15 | | | | 2.37678250571877e-13 | | | | | 23.4854851938108 | | | | |
| 1.20330771247949 | | | | | 1.6648177330436 | | | | 7.99369676592965 | | | | | 5.03474609209932e-15 | | | | 2.4040328214874e-13 | | | | | 23.4806497096499 | | | | |
| 1.08429060040597 | | | | | 3.33503707584818 | | | | 7.98880098583594 | | | | | 5.02536110065757e-15 | | | | 2.40063896602954e-13 | | | | | 23.4753706458774 | | | | |
| -1.001552646 | | | | | -0.812011775 | | | | -7.989416891 | | | | | 5.05158085766918e-15 | | | | 2.41179812075788e-13 | | | | | 23.4718803693891 | | | | |
| -1.236111364 | | | | | 0.510978795625872 | | | | -7.987050745 | | | | | 5.09161167298001e-15 | | | | 2.42816092981846e-13 | | | | | 23.4625980771338 | | | | |
| -1.127589435 | | | | | 0.279485393925461 | | | | -7.988375942 | | | | | 5.16796632187309e-15 | | | | 2.46095579779133e-13 | | | | | 23.4552372506414 | | | | |
| -1.074684943 | | | | | 0.5991348989849 | | | | -7.984466481 | | | | | 5.19100984064611e-15 | | | | 2.47081286771108e-13 | | | | | 23.4437434554882 | | | | |
| -1.217454648 | | | | | 0.148864549360336 | | | | -7.985565888 | | | | | 5.22498939848104e-15 | | | | 2.48586401856877e-13 | | | | | 23.4392960912869 | | | | |
| 1.00665074138523 | | | | | 1.78551064358951 | | | | 7.98302124667925 | | | | | 5.24742958931506e-15 | | | | 2.49569550521823e-13 | | | | | 23.4332013691359 | | | | |
| -1.155875855 | | | | | 0.739174095859046 | | | | -7.98765596 | | | | | 5.35013595930011e-15 | | | | 2.53938747181233e-13 | | | | | 23.4277287981745 | | | | |
| -1.179561334 | | | | | 0.346895424497436 | | | | -7.981218244 | | | | | 5.33593755827389e-15 | | | | 2.53350387365989e-13 | | | | | 23.4180445656921 | | | | |
| 1.01254103906005 | | | | | -3.985705301 | | | | 7.97678287409879 | | | | | 5.49798672575319e-15 | | | | 2.60165645005987e-13 | | | | | 23.3877147870145 | | | | |
| -1.127792488 | | | | | -2.723439864 | | | | -7.975939101 | | | | | 5.53276939704767e-15 | | | | 2.61547407135997e-13 | | | | | 23.3815647907324 | | | | |
| -1.020640081 | | | | | 0.0164795991387824 | | | | -7.975297735 | | | | | 5.55935339404501e-15 | | | | 2.62656867861844e-13 | | | | | 23.3768904421322 | | | | |
| 1.20876599427317 | | | | | -1.448527595 | | | | 7.97498340264539 | | | | | 5.5724281321173e-15 | | | | 2.631566547732e-13 | | | | | 23.3745996669735 | | | | |
| -1.03637686 | | | | | -0.10429206 | | | | -7.983013134 | | | | | 5.71019655351684e-15 | | | | 2.68987657153062e-13 | | | | | 23.3669426836591 | | | | |
| -1.044666342 | | | | | -1.268636359 | | | | -7.996761468 | | | | | 5.81418557014743e-15 | | | | 2.73471012756728e-13 | | | | | 23.3665933482689 | | | | |
| -1.034770051 | | | | | -0.506890791 | | | | -7.981015711 | | | | | 5.73275832681902e-15 | | | | 2.69942177964241e-13 | | | | | 23.3624573029928 | | | | |
| -1.193475961 | | | | | 1.34141244149165 | | | | -7.973194573 | | | | | 5.64741437941155e-15 | | | | 2.66339919624927e-13 | | | | | 23.3615645871036 | | | | |
| -1.166254729 | | | | | 0.694241435037207 | | | | -7.977135697 | | | | | 5.72654793033128e-15 | | | | 2.69679859926602e-13 | | | | | 23.3572782048645 | | | | |
| -1.09879927 | | | | | -0.862999716 | | | | -8.023655432 | | | | | 6.02079834444498e-15 | | | | 2.82527268851782e-13 | | | | | 23.3484875225174 | | | | |
| 1.04667079993376 | | | | | 2.22803829110534 | | | | 7.97096037556184 | | | | | 5.74246911732745e-15 | | | | 2.70308880632224e-13 | | | | | 23.3452876031699 | | | | |
| -1.193753649 | | | | | 0.214384701179727 | | | | -7.972736972 | | | | | 5.84719269364878e-15 | | | | 2.74808932582577e-13 | | | | | 23.3352973350724 | | | | |
| -1.076966783 | | | | | -0.41285906 | | | | -7.98175094 | | | | | 5.92817753569452e-15 | | | | 2.7839788634328e-13 | | | | | 23.3345927786233 | | | | |
| 1.54491134430393 | | | | | 0.506474760917109 | | | | 7.99560667241662 | | | | | 6.09097670378191e-15 | | | | 2.85381653578527e-13 | | | | | 23.3238319686362 | | | | |
| 1.17603118596185 | | | | | 0.379779843348839 | | | | 7.96827720828119 | | | | | 5.89669211462944e-15 | | | | 2.76980969586253e-13 | | | | | 23.3208218818861 | | | | |
| -1.339020625 | | | | | 1.7352256375444 | | | | -7.99913002 | | | | | 6.25236374238418e-15 | | | | 2.92320015439754e-13 | | | | | 23.3052257988311 | | | | |
| -1.246145535 | | | | | 0.568086273156124 | | | | -7.964899987 | | | | | 6.00832849516542e-15 | | | | 2.81981023899509e-13 | | | | | 23.3011547591334 | | | | |
| -1.246202231 | | | | | 0.683673516511705 | | | | -7.9637494 | | | | | 6.07967723739395e-15 | | | | 2.84941369422241e-13 | | | | | 23.2908064906896 | | | | |
| -1.075791493 | | | | | 1.09883764821393 | | | | -7.963257215 | | | | | 6.08246178993149e-15 | | | | 2.85040193705313e-13 | | | | | 23.2891966794478 | | | | |
| 1.34556789677735 | | | | | 0.0453078656599619 | | | | 7.96299034530963 | | | | | 6.10438172081053e-15 | | | | 2.85908546063998e-13 | | | | | 23.2857977372582 | | | | |
| -1.223900097 | | | | | 0.0178697349005123 | | | | -8.009400181 | | | | | 6.45614347393342e-15 | | | | 3.00846725456239e-13 | | | | | 23.2785341403359 | | | | |
| -1.117736054 | | | | | -0.599536646 | | | | -7.96401344 | | | | | 6.2087069773913e-15 | | | | 2.90536618705648e-13 | | | | | 23.2728008481226 | | | | |
| -1.186607074 | | | | | 0.801301413177117 | | | | -8.02447746 | | | | | 6.54417189038263e-15 | | | | 3.04309766323075e-13 | | | | | 23.2725147513977 | | | | |
| -1.224567675 | | | | | 2.17916938078462 | | | | -7.960221675 | | | | | 6.22182765899775e-15 | | | | 2.91085994560034e-13 | | | | | 23.2671058339391 | | | | |
| -1.110542999 | | | | | -0.800953451 | | | | -8.028811548 | | | | | 6.71200625671845e-15 | | | | 3.11393077155954e-13 | | | | | 23.2498144971217 | | | | |
| -1.167391478 | | | | | -0.104409763 | | | | -7.968072136 | | | | | 6.48784577426929e-15 | | | | 3.0218509054263e-13 | | | | | 23.246917199553 | | | | |
| -1.069076979 | | | | | 0.693385587826191 | | | | -7.956826304 | | | | | 6.39167066775708e-15 | | | | 2.98369951342093e-13 | | | | | 23.2409528854485 | | | | |
| 1.13862261490755 | | | | | 0.0264237865062062 | | | | 7.95637833618172 | | | | | 6.40280606112668e-15 | | | | 2.98823600641427e-13 | | | | | 23.2391465255086 | | | | |
| -1.135298966 | | | | | 0.919783932133503 | | | | -7.956162403 | | | | | 6.41312671946367e-15 | | | | 2.99205924296995e-13 | | | | | 23.2375760074581 | | | | |
| 1.3503509698632 | | | | | -2.306119905 | | | | 7.95572729567522 | | | | | 6.43397284960453e-15 | | | | 3.00012532510532e-13 | | | | | 23.2344115015982 | | | | |
| -1.051984418 | | | | | 0.642767131279282 | | | | -7.957886236 | | | | | 6.50953736048654e-15 | | | | 3.03033409008209e-13 | | | | | 23.2304906831734 | | | | |
| -1.27662603 | | | | | 0.651073903086912 | | | | -7.958712746 | | | | | 6.62544863835155e-15 | | | | 3.07715929874843e-13 | | | | | 23.22627971 | | | | |
| -1.332286766 | | | | | 0.884830708132781 | | | | -7.960757025 | | | | | 6.60584554956616e-15 | | | | 3.06906885509648e-13 | | | | | 23.2246984805552 | | | | |
| -1.291858894 | | | | | 0.812653762033112 | | | | -7.955534735 | | | | | 6.5269467626932e-15 | | | | 3.03670655760803e-13 | | | | | 23.2231864192997 | | | | |
| -1.155801553 | | | | | 1.65176273416802 | | | | -7.952268214 | | | | | 6.62324485417076e-15 | | | | 3.07647461738069e-13 | | | | | 23.2082582863029 | | | | |
| -1.151556189 | | | | | -0.019470879 | | | | -7.975711089 | | | | | 6.89679804778899e-15 | | | | 3.18878600088946e-13 | | | | | 23.2050224458523 | | | | |
| 1.44245160206271 | | | | | -3.069866424 | | | | 7.95399921852929 | | | | | 6.79153853784563e-15 | | | | 3.14563714329236e-13 | | | | | 23.1927572659593 | | | | |
| -1.044717829 | | | | | 2.26188031304383 | | | | -7.950457613 | | | | | 6.73483088756041e-15 | | | | 3.12211536978628e-13 | | | | | 23.1921844986181 | | | | |
| -1.182038503 | | | | | 1.51915304640218 | | | | -7.948338228 | | | | | 6.798362208477e-15 | | | | 3.14845183135474e-13 | | | | | 23.180693692728 | | | | |
| -1.169858918 | | | | | 0.871547164341761 | | | | -7.9520315 | | | | | 6.87997010513561e-15 | | | | 3.18170321806595e-13 | | | | | 23.1782190847051 | | | | |
| -1.053386584 | | | | | 0.08598183714399 | | | | -7.94682704 | | | | | 6.87535521124702e-15 | | | | 3.1806155040335e-13 | | | | | 23.1697126947096 | | | | |
| -1.207404165 | | | | | 0.384912292494085 | | | | -7.947187501 | | | | | 6.90092377132173e-15 | | | | 3.19034374153408e-13 | | | | | 23.1674823827445 | | | | |
| -1.091582313 | | | | | 2.29990190656068 | | | | -7.946392008 | | | | | 6.90867472245009e-15 | | | | 3.1932268663987e-13 | | | | | 23.1660620379719 | | | | |
| -1.037873088 | | | | | -1.239134529 | | | | -7.945531553 | | | | | 6.99781085640975e-15 | | | | 3.23053087784355e-13 | | | | | 23.1549474520701 | | | | |
| -1.461353422 | | | | | -0.196410337 | | | | -7.955213949 | | | | | 7.1369626416614e-15 | | | | 3.28793077363636e-13 | | | | | 23.1480502399881 | | | | |
| -1.122106937 | | | | | -1.170734348 | | | | -7.978143862 | | | | | 7.36622910374836e-15 | | | | 3.38430793874218e-13 | | | | | 23.1442286503624 | | | | |
| -1.166293795 | | | | | 1.24846848200226 | | | | -7.953088023 | | | | | 7.26376081261814e-15 | | | | 3.34232870128404e-13 | | | | | 23.1386793029425 | | | | |
| -1.345504412 | | | | | 1.81281880053451 | | | | -7.942659175 | | | | | 7.12618518321966e-15 | | | | 3.28476006362946e-13 | | | | | 23.1351015469017 | | | | |
| 1.04651539560599 | | | | | 0.645395295155924 | | | | 7.94196438609368 | | | | | 7.12898290262942e-15 | | | | 3.28497237520681e-13 | | | | | 23.1343903664466 | | | | |
| -1.183353986 | | | | | 0.244841009935842 | | | | -7.941787013 | | | | | 7.13840676076843e-15 | | | | 3.28823681321086e-13 | | | | | 23.1331022704739 | | | | |
| -1.199242833 | | | | | -1.892828471 | | | | -7.967684732 | | | | | 7.38191477001153e-15 | | | | 3.39040627499266e-13 | | | | | 23.1260206423912 | | | | |
| -1.244918077 | | | | | 2.75688230077467 | | | | -7.947948607 | | | | | 7.36891213133141e-15 | | | | 3.38517178169574e-13 | | | | | 23.1250292462507 | | | | |
| -1.138377329 | | | | | 1.51989552874221 | | | | -7.952585813 | | | | | 7.38721882417186e-15 | | | | 3.39247283989169e-13 | | | | | 23.1246290216034 | | | | |
| -1.735829677 | | | | | 0.315595399579803 | | | | -8.064789033 | | | | | 7.75954402028326e-15 | | | | 3.54761674838213e-13 | | | | | 23.1092076268197 | | | | |
| -1.138886918 | | | | | 1.22847879362906 | | | | -7.936843893 | | | | | 7.40603385587226e-15 | | | | 3.40074299902609e-13 | | | | | 23.0972148580253 | | | | |
| -1.084024962 | | | | | 0.446316609443122 | | | | -7.937177996 | | | | | 7.42296078730047e-15 | | | | 3.40769499208223e-13 | | | | | 23.0962741193954 | | | | |
| -1.076598878 | | | | | -3.395360913 | | | | -7.936511043 | | | | | 7.42440626372742e-15 | | | | 3.40769499208223e-13 | | | | | 23.0947990171222 | | | | |
| -1.061678321 | | | | | 0.492057121161603 | | | | -7.938160381 | | | | | 7.55096067788219e-15 | | | | 3.46050819372088e-13 | | | | | 23.0887370875276 | | | | |
| 1.03658356949084 | | | | | 1.89994297890308 | | | | 7.93527701981385 | | | | | 7.49291389085538e-15 | | | | 3.43652055727321e-13 | | | | | 23.0858431828204 | | | | |
| 1.0159842810453 | | | | | 1.17760560220978 | | | | 7.93435384447556 | | | | | 7.54457185824735e-15 | | | | 3.45833200942185e-13 | | | | | 23.0791440738945 | | | | |
| -1.173525749 | | | | | 0.298911104642164 | | | | -7.934102637 | | | | | 7.59477814327581e-15 | | | | 3.47832095109711e-13 | | | | | 23.0730058533786 | | | | |
| -1.671847234 | | | | | 0.471679575328068 | | | | -8.121425238 | | | | | 7.76925345344514e-15 | | | | 3.55095808899436e-13 | | | | | 23.0728587247313 | | | | |
| 1.48066878502334 | | | | | -2.797758545 | | | | 7.93350187696534 | | | | | 7.61666761237368e-15 | | | | 3.48758848095299e-13 | | | | | 23.0700898175613 | | | | |
| 1.27500214748456 | | | | | 0.612757975279719 | | | | 7.93197224061074 | | | | | 7.69162594044778e-15 | | | | 3.51961791677549e-13 | | | | | 23.0604314875637 | | | | |
| 1.45953156940837 | | | | | 0.0828697413662526 | | | | 7.9325692227819 | | | | | 7.71883495827521e-15 | | | | 3.53091900288122e-13 | | | | | 23.0604030351188 | | | | |
| -1.425408971 | | | | | 1.36237962931219 | | | | -7.931410489 | | | | | 7.79813862213546e-15 | | | | 3.56255808536929e-13 | | | | | 23.0486392847174 | | | | |
| 1.1322309124623 | | | | | 0.869038200579308 | | | | 7.92938157832874 | | | | | 7.8289030274481e-15 | | | | 3.57467627459019e-13 | | | | | 23.0430736932544 | | | | |
| 1.09006680305222 | | | | | 0.0863059066631032 | | | | 7.92906566113793 | | | | | 7.88469901405368e-15 | | | | 3.5993732091112e-13 | | | | | 23.0364784432752 | | | | |
| -1.159406336 | | | | | 0.059176302691436 | | | | -7.928717626 | | | | | 7.91772165838021e-15 | | | | 3.61288355339161e-13 | | | | | 23.0325136315566 | | | | |
| -1.054548316 | | | | | -2.544056025 | | | | -7.928352761 | | | | | 7.95190417317099e-15 | | | | 3.6276960215309e-13 | | | | | 23.0284238440682 | | | | |
| 1.03803022275294 | | | | | 1.31515501030818 | | | | 7.92658366837085 | | | | | 7.99351615089578e-15 | | | | 3.64510214701874e-13 | | | | | 23.0227851854795 | | | | |
| 1.3585074618758 | | | | | -0.340578125 | | | | 7.92751332222731 | | | | | 8.00167776971066e-15 | | | | 3.6484293524588e-13 | | | | | 23.0223399833822 | | | | |
| -1.354488158 | | | | | 1.94233007802355 | | | | -7.933726999 | | | | | 8.10165247786997e-15 | | | | 3.69082087331831e-13 | | | | | 23.0215963032452 | | | | |
| -1.062663589 | | | | | 1.73806701897959 | | | | -7.926091968 | | | | | 8.21838778428363e-15 | | | | 3.73875021792953e-13 | | | | | 23.0040241010482 | | | | |
| 1.41402509623797 | | | | | 2.84805715350976 | | | | 7.92339967667614 | | | | | 8.18499892738931e-15 | | | | 3.7243644088834e-13 | | | | | 22.9997044495448 | | | | |
| -1.219827352 | | | | | 0.18025322494577 | | | | -7.925145866 | | | | | 8.3171822071333e-15 | | | | 3.78124659976485e-13 | | | | | 22.9889351502024 | | | | |
| -1.442547273 | | | | | 1.7486101262093 | | | | -7.921856978 | | | | | 8.27939532233723e-15 | | | | 3.76487935465323e-13 | | | | | 22.988524261101 | | | | |
| -1.14740417 | | | | | 0.143592230074251 | | | | -7.932153367 | | | | | 8.4499533750937e-15 | | | | 3.83705776402731e-13 | | | | | 22.9860415493101 | | | | |
| 1.30496852574944 | | | | | 0.353238670226698 | | | | 7.92415237256126 | | | | | 8.33757797508091e-15 | | | | 3.78929349509848e-13 | | | | | 22.9843179944837 | | | | |
| -1.134820754 | | | | | -0.344921535 | | | | -7.942404085 | | | | | 8.62618997818092e-15 | | | | 3.90614854821687e-13 | | | | | 22.9795762571147 | | | | |
| -1.61306883 | | | | | 2.39262869816022 | | | | -7.924988135 | | | | | 8.52484990204119e-15 | | | | 3.86524027762764e-13 | | | | | 22.9792166387872 | | | | |
| -1.15317984 | | | | | 1.0990346261925 | | | | -7.920911283 | | | | | 8.49899745045008e-15 | | | | 3.85434744561334e-13 | | | | | 22.9690966120975 | | | | |
| -1.022632743 | | | | | -0.472453634 | | | | -7.919388076 | | | | | 8.45930482595534e-15 | | | | 3.84047703053466e-13 | | | | | 22.9677841194985 | | | | |
| -1.185606799 | | | | | 0.138234387213518 | | | | -7.937712518 | | | | | 8.71029591765084e-15 | | | | 3.94000265993974e-13 | | | | | 22.9664760152142 | | | | |
| -1.066730802 | | | | | -0.773456729 | | | | -7.918764301 | | | | | 8.47187847688402e-15 | | | | 3.84577133959273e-13 | | | | | 22.9661166693027 | | | | |
| 1.06835175065114 | | | | | 1.13226797607578 | | | | 7.94496382588489 | | | | | 8.74351348720257e-15 | | | | 3.95269190690316e-13 | | | | | 22.9659229358047 | | | | |
| 1.12109607818192 | | | | | -1.397718273 | | | | 7.91718081837123 | | | | | 8.57213315514038e-15 | | | | 3.8854255482404e-13 | | | | | 22.9546466157779 | | | | |
| 1.0395679459054 | | | | | -1.174245274 | | | | 7.9169465951044 | | | | | 8.58706145982644e-15 | | | | 3.89093725098325e-13 | | | | | 22.952950170239 | | | | |
| 1.06260496320857 | | | | | -0.176244477 | | | | 7.91684526905916 | | | | | 8.62060323899123e-15 | | | | 3.90460828773385e-13 | | | | | 22.9503356251512 | | | | |
| -1.103391073 | | | | | -0.229724642 | | | | -7.926414582 | | | | | 8.76817973470547e-15 | | | | 3.96108672459101e-13 | | | | | 22.9470396615248 | | | | |
| -1.163896615 | | | | | 0.882749378030842 | | | | -7.915502996 | | | | | 8.67963732593703e-15 | | | | 3.92781999487859e-13 | | | | | 22.9424953258619 | | | | |
| -1.54950942 | | | | | 1.27914819219068 | | | | -8.030317661 | | | | | 8.74921204505088e-15 | | | | 3.95421247913891e-13 | | | | | 22.941157958919 | | | | |
| 1.09690745441348 | | | | | 0.554617565193498 | | | | 7.91549560865112 | | | | | 8.69375138267303e-15 | | | | 3.93336281709414e-13 | | | | | 22.9410207807371 | | | | |
| -1.203896827 | | | | | 3.41112177876048 | | | | -7.914871418 | | | | | 8.7204482918297e-15 | | | | 3.94374885372783e-13 | | | | | 22.9379218157989 | | | | |
| -1.213037331 | | | | | 1.77143911482233 | | | | -7.920302385 | | | | | 8.9174231259094e-15 | | | | 4.02402566581649e-13 | | | | | 22.9306254159038 | | | | |
| -1.325254849 | | | | | 1.23979687961805 | | | | -7.953903228 | | | | | 9.29791638199223e-15 | | | | 4.1802456560344e-13 | | | | | 22.9262898400294 | | | | |
| 1.25073247424619 | | | | | -0.341779275 | | | | 7.91241897646603 | | | | | 8.88072053061493e-15 | | | | 4.00935056332377e-13 | | | | | 22.920165625339 | | | | |
| -1.231556958 | | | | | 1.25566535425129 | | | | -7.916022656 | | | | | 8.95843427817633e-15 | | | | 4.04054228482042e-13 | | | | | 22.9188706501349 | | | | |
| -1.237694644 | | | | | 2.02470116728266 | | | | -7.913183785 | | | | | 9.00053330348457e-15 | | | | 4.05866204478702e-13 | | | | | 22.9141673326529 | | | | |
| -1.16082187 | | | | | -0.037442493 | | | | -7.91772446 | | | | | 9.26631945028013e-15 | | | | 4.1678175174396e-13 | | | | | 22.8971031391769 | | | | |
| -1.297588208 | | | | | 0.96458139869501 | | | | -7.929889617 | | | | | 9.47746467466067e-15 | | | | 4.25379533508666e-13 | | | | | 22.8963634191031 | | | | |
| 1.04362897358851 | | | | | 0.518052547765146 | | | | 7.90758226130433 | | | | | 9.20537015717622e-15 | | | | 4.14217098941041e-13 | | | | | 22.8851604488309 | | | | |
| 1.02978044543026 | | | | | -2.438092595 | | | | 7.90791040434132 | | | | | 9.31449996215182e-15 | | | | 4.18680865477088e-13 | | | | | 22.876595013738 | | | | |
| -1.464532498 | | | | | 1.12203902078527 | | | | -7.979896753 | | | | | 9.96147499091949e-15 | | | | 4.4501061123223e-13 | | | | | 22.8681464263142 | | | | |
| -1.139744658 | | | | | 0.772446770434926 | | | | -7.905787804 | | | | | 9.38748730854956e-15 | | | | 4.21826702194809e-13 | | | | | 22.8674713245586 | | | | |
| 1.43372772724481 | | | | | 0.173428319662458 | | | | 7.91232233963958 | | | | | 9.73023602273848e-15 | | | | 4.35187592671631e-13 | | | | | 22.8482960374169 | | | | |
| -1.284038629 | | | | | 2.08460621508245 | | | | -7.902016417 | | | | | 9.60843735704474e-15 | | | | 4.3083685456668e-13 | | | | | 22.8444644591643 | | | | |
| 1.09920637047079 | | | | | 0.791905601970784 | | | | 7.90009995714113 | | | | | 9.74596600991601e-15 | | | | 4.35844892077769e-13 | | | | | 22.8296341645111 | | | | |
| -1.055520251 | | | | | 0.221939598031911 | | | | -7.899324199 | | | | | 9.84833836770149e-15 | | | | 4.40189616229456e-13 | | | | | 22.8217234264906 | | | | |
| -1.358731582 | | | | | 1.85351111266386 | | | | -7.902502181 | | | | | 9.85328824745728e-15 | | | | 4.40364181802365e-13 | | | | | 22.8209799369153 | | | | |
| 1.02691617979668 | | | | | -0.642751943 | | | | 7.89660998508905 | | | | | 1.00484461867187e-14 | | | | 4.48658227822287e-13 | | | | | 22.8011419232905 | | | | |
| -1.315400928 | | | | | 0.803854501956795 | | | | -7.938266026 | | | | | 1.06403379707658e-14 | | | | 4.72733253833401e-13 | | | | | 22.7965492876256 | | | | |
| -1.218089466 | | | | | 1.41486711208097 | | | | -7.900106109 | | | | | 1.03893155902379e-14 | | | | 4.62360179940435e-13 | | | | | 22.7856499814779 | | | | |
| 1.06316892574353 | | | | | 1.85601954462237 | | | | 7.89349745006838 | | | | | 1.02189130615018e-14 | | | | 4.5554594255666e-13 | | | | | 22.7833264862548 | | | | |
| -1.38215235 | | | | | 0.44943274416373 | | | | -7.892009325 | | | | | 1.03322211253806e-14 | | | | 4.60204212934689e-13 | | | | | 22.7725762419972 | | | | |
| 1.33698171861842 | | | | | 1.06854591333523 | | | | 7.89164058852381 | | | | | 1.03604882210208e-14 | | | | 4.61174608693956e-13 | | | | | 22.7699127488954 | | | | |
| -1.117504993 | | | | | -1.306703479 | | | | -7.938295932 | | | | | 1.09885683977149e-14 | | | | 4.86670602901818e-13 | | | | | 22.7614836585221 | | | | |
| -1.236316582 | | | | | 0.693778176364591 | | | | -7.906527624 | | | | | 1.08685258221027e-14 | | | | 4.81906549254054e-13 | | | | | 22.7521737432358 | | | | |
| -1.350760895 | | | | | 1.86655216112295 | | | | -7.889127602 | | | | | 1.05551731401711e-14 | | | | 4.69246163206743e-13 | | | | | 22.7517635063638 | | | | |
| 1.04079992575544 | | | | | 2.3626980796327 | | | | 7.88732742715794 | | | | | 1.07134324445168e-14 | | | | 4.75680010411646e-13 | | | | | 22.7373650582645 | | | | |
| -1.086966202 | | | | | 0.821426192713368 | | | | -7.885804287 | | | | | 1.08181841194856e-14 | | | | 4.79775315278027e-13 | | | | | 22.7277694430546 | | | | |
| -1.06931231 | | | | | 1.83164826277996 | | | | -7.905532539 | | | | | 1.12854090767157e-14 | | | | 4.98399502029607e-13 | | | | | 22.7238680984388 | | | | |
| -1.159524675 | | | | | 0.57609758072449 | | | | -7.896473097 | | | | | 1.1116369826177e-14 | | | | 4.91861454837782e-13 | | | | | 22.7213592765437 | | | | |
| -1.427837671 | | | | | 0.513981112020034 | | | | -7.903943486 | | | | | 1.12327663951536e-14 | | | | 4.96416580794544e-13 | | | | | 22.7176418283165 | | | | |
| -1.259589962 | | | | | 2.34136680795067 | | | | -7.883584691 | | | | | 1.0997433719213e-14 | | | | 4.87007758494069e-13 | | | | | 22.7117489119246 | | | | |
| -1.13789878 | | | | | -0.601205576 | | | | -7.88836425 | | | | | 1.10658608708422e-14 | | | | 4.89729382179685e-13 | | | | | 22.71056276 | | | | |
| -1.350739252 | | | | | 0.86798876281737 | | | | -7.891555714 | | | | | 1.14634130578226e-14 | | | | 5.05836896619095e-13 | | | | | 22.6945927972514 | | | | |
| -1.060203225 | | | | | 0.136610803175002 | | | | -7.880568578 | | | | | 1.12457092460003e-14 | | | | 4.96906446375455e-13 | | | | | 22.6899854388455 | | | | |
| -1.123361575 | | | | | 0.588743110368593 | | | | -7.88174401 | | | | | 1.1601165071981e-14 | | | | 5.10952922070424e-13 | | | | | 22.6663193605798 | | | | |
| 1.81540244395325 | | | | | 0.912039894807346 | | | | 7.87779771226238 | | | | | 1.15679627636306e-14 | | | | 5.0986336976483e-13 | | | | | 22.6649599575963 | | | | |
| 1.0811018420858 | | | | | 2.93204922050649 | | | | 7.87617357814044 | | | | | 1.16174130946198e-14 | | | | 5.1156167289143e-13 | | | | | 22.658284914247 | | | | |
[truncated: 889,441 more chars]
